# μ-Oxo-bis[(octacosafluoro-*meso*-tetraphenylporphyrinato)iron(iii)] – synthesis, crystal structure, and catalytic activity in oxidation reactions†

**DOI:** 10.1039/d2sc06083c

**Published:** 2022-12-07

**Authors:** Tristan Schuh, Olga Kataeva, Hans-Joachim Knölker

**Affiliations:** a Fakultät Chemie, Technische Universität Dresden Bergstrasse 66 01069 Dresden Germany hans-joachim.knoelker@tu-dresden.de https://tu-dresden.de/mn/chemie/oc/oc2 +49 351-463-37030

## Abstract

We describe the synthesis and X-ray crystal structure of μ*-*oxo-bis[(octacosafluoro-*meso*-tetraphenylporphyrinato)iron(iii)] [(FeTPPF_28_)_2_O]. This novel iron complex is an efficient catalyst for oxidative biaryl coupling reactions of diarylamines and carbazoles. The asymmetric oxidative coupling in the presence of an axially chiral biaryl phosphoric acid as co-catalyst provides the 2,2′-bis(arylamino)-1,1′-biaryl in 96% *ee*. The Wacker-type oxidation of alkenes to the corresponding ketones with (FeTPPF_28_)_2_O as catalyst in the presence of phenylsilane proceeds at room temperature with air as the terminal oxidant. For internal and aliphatic alkenes increased ketone/alcohol product ratios were obtained.

## Abstract 2

We describe the synthesis and crystal structure of a novel perfluorinated μ-oxo-bis[(porphyrinato)iron(iii)] complex enabling useful applications in catalytic C–H activations including asymmetric biaryl coupling and Wacker-type oxidation of olefins.

## Graphical abstract

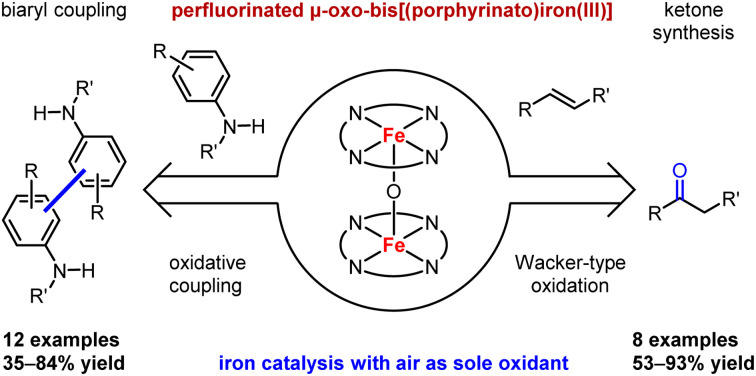

## Introduction

Limited resources and environmental issues have promoted the development of sustainable chemistry. In organometallic catalysis the classical noble metals like palladium and iridium, which are expensive and toxic, are being replaced by first row transition metals. Among those, iron is the prime candidate for environmentally benign organometallic catalysis because of its high abundance and low toxicity.^1,2^ In nature, porphyrin–iron complexes are essential biocatalysts with cytochrome P450 enzymes as the most important class. These oxidoreductases occur in nearly all organisms.^3^ Moreover, they also catalyze uncommon transformations like rearrangements, cyclizations, and intramolecular C–C and C–heteroatom coupling reactions.^4–10^ These reactions generally proceed either *via* a hydrogen atom transfer (HAT) or single-electron transfer (SET) process.^11,12^ Therefore, the design of novel electron-deficient porphyrin–iron complexes could open up the way to unprecedented biomimetic reactions for organic synthesis. In this respect, the properties of μ-oxo-bridged binuclear porphyrinoid complexes have recently attracted a lot of attention.^13,14^

## Results and discussion

In the present study,‡‡Part 153 of “Transition Metals in Organic Synthesis”; for part 152, see: ref. 30. we describe the synthesis, structural characterization, and applications in catalysis of the strongly electron-deficient complex μ*-*oxo-bis[(octacosafluoro-*meso*-tetraphenylporphyrinato)iron(iii)] [(FeTPPF_28_)_2_O] (5c) (Table 1). We focused our efforts on the fluorinated porphyrin ligands, since in addition to the electron-withdrawing effect of the fluorine atoms they improve considerably the solubility of the complexes.^15,16^

**Table 1:** Synthesis of the fluorinated μ*-*oxo-porphyrin–iron(iii) complexes 5^a^

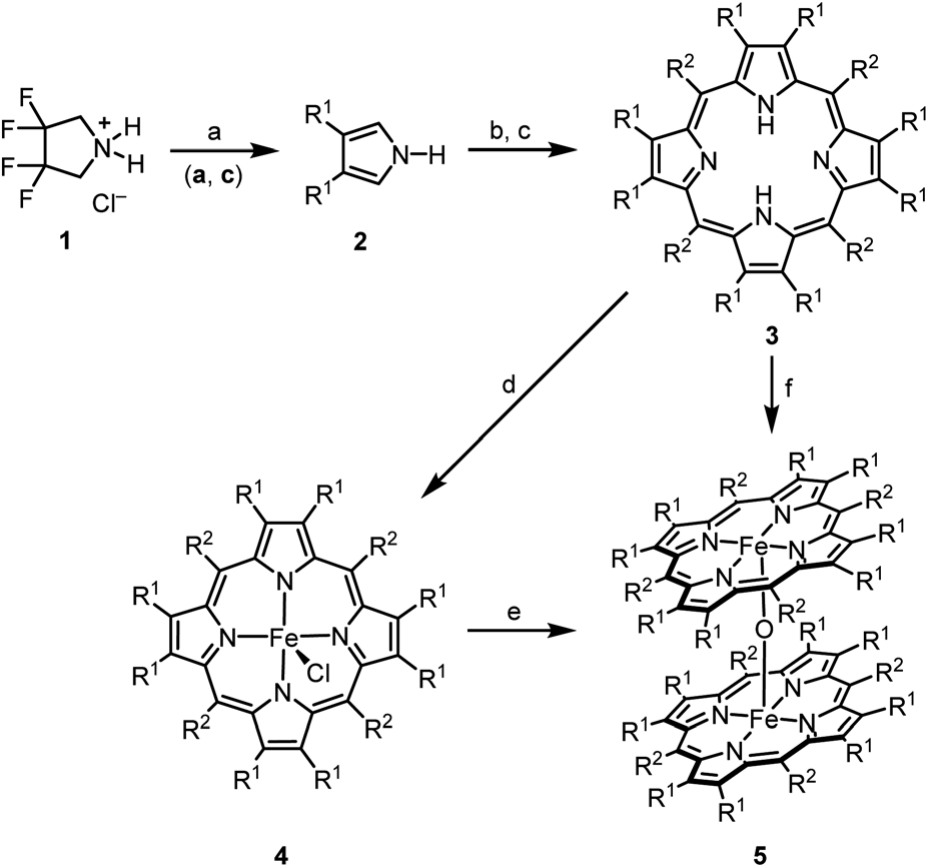					
	R^1^	R^2^	3	4, Yield [%]	5, Yield [%]
a	F	C_6_H_5_	H_2_TPPF_8_	FeTPPF_8_Cl, 59	—
b	H	C_6_F_5_	H_2_TPPF_20_	FeTPPF_20_Cl, 96	(FeTPPF_20_)_2_O, 98
c	F	C_6_F_5_	H_2_TPPF_28_	FeTPPF_28_Cl, 92	(FeTPPF_28_)_2_O, >99

a Reaction conditions: (a) 1 (1.0 equiv.), KO*t*-Bu (4.0 equiv.), DMSO, Ar, rt, 0.5 h; (b) 2 (1.0 equiv.), R^2^CHO (1.1 equiv.), BF_3_·OEt_2_ (4.0 equiv.), CH_2_Cl_2_, Ar, rt; (c) DDQ (1.0 equiv.), pyridine (8.0 equiv.), Ar, rt; (d) FeCl_2_ (20 equiv.), MeCN, air, 120 °C, 4 h, sealed tube; (e) activated alumina, CH_2_Cl_2_/MeOH (95 : 5), air, rt; (f) FeCl_2_ (20 equiv.), MeCN, air, 120 °C, 4 h, sealed tube; followed by elution of the crude product over activated alumina, CH_2_Cl_2_/MeOH (95 : 5), air, rt (see ESI for details).

Homogeneous catalysis sometimes suffers from low solubility. However, moderately fluorinated organometallic complexes generally allow a broader spectrum of solvents that can be used. The first syntheses of β-octafluoro-substituted *meso*-tetraphenylporphyrins and their zinc complexes were reported independently by two different groups in 1997.^17,18^ The direct introduction of fluorine substituents at the porphyrin ring is not possible and thus β-octafluoro-*meso*-tetraphenylporphyrins (3a and 3c) are synthesized by condensation of 3,4-difluoropyrrole (2a) with the corresponding benzaldehydes. The β-octafluorinated porphyrins 3a and 3c are accessible from 3,3,4,4-tetrafluoropyrrolidinium chloride (1) *via* a three-step sequence reported by DiMagno *et al.*^18,19^ According to ^1^H and ^19^F NMR analysis (see SI), the elimination of hydrogen fluoride proceeds nearly quantitatively. However, DiMagno *et al.* isolated 3,4-difluoropyrrole (2a) in only 53% yield due to the extremely high volatility of this compound.^19^ We found that the overall yield of the porphyrins 3a and 3c is considerably improved by avoiding the isolation of 2a and submitting the crude product directly to the cyclocondensation step. The formation of the fluorinated tetraphenylporphyrin–iron complexes 4a–4c from the corresponding porphyrins using the classical conditions described by Adler (DMF at reflux)^20^ led to a complex reaction mixture. This mixture is resulting from nucleophilic aromatic substitution at the fluorinated porphyrins by dimethylamine, formed by decarbonylation of the solvent. Adapting the conditions reported by Freire *et al.*, complexation of 3a–3c was achieved by reaction with iron(ii) chloride in acetonitrile at 120 °C.^21^

Several preparations of μ*-*oxo-porphyrinoid–iron complexes have been reported.^13,14,22,23^ The β-octafluoro-substituted μ-oxo-iron complex (FeTPPF_8_)_2_O (5a) could not be prepared due to the extremely low solubility of the corresponding chloro–iron complex 4a. Complex 5b was described previously.^24^ Elution of the chloro complex 4c over activated alumina (CH_2_Cl_2_/MeOH, 95 : 5) provided quantitatively the μ*-*oxo complex (FeTPPF_28_)_2_O (5c).

**Fig. 1:**
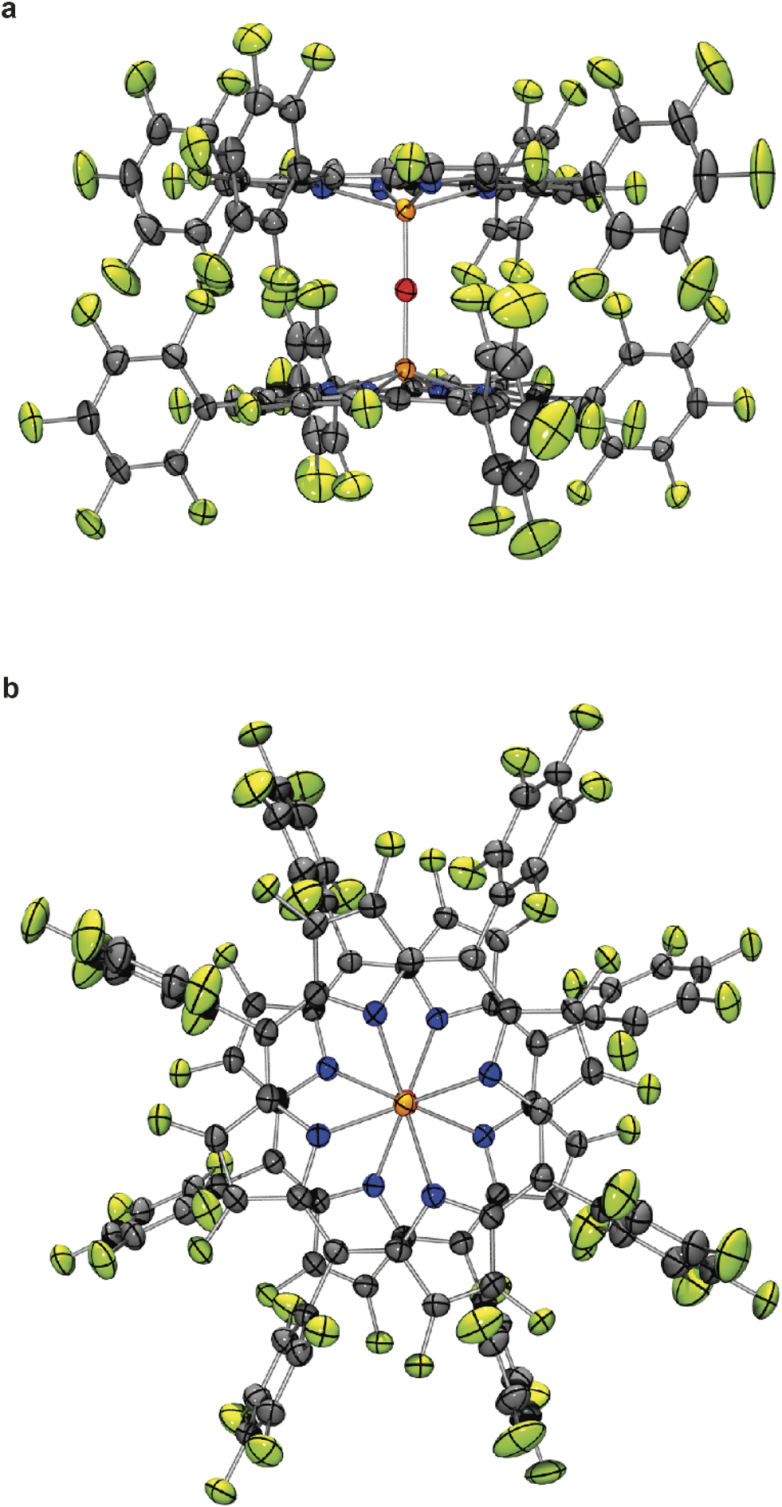
Molecular structure of μ*-*oxo-bis[(octacosafluoro-*meso*-tetra-phenylporphyrinato)iron(iii)] (5c) in the crystal (thermal ellipsoids are shown at the 50% probability level); (a) side view; (b) view along the Fe–O–Fe axis. Bond length Fe–O 1.7816(6) Å; bond angle Fe–O–Fe 178.34(19)°.

Deep red cubic crystals of complex 5c suitable for X-ray crystallography were obtained by recrystallization from dichloromethane (Fig. 1).^25^ The geometry of 5c is similar to the previously reported structures of the μ-oxo-bis[(tetraphenylporphyrinato)iron(iii)] complexes (FeTPPF_20_)_2_O (5b) and (FeTPP)_2_O.^24,26^ Remarkable is the central, nearly linear Fe–O–Fe axis with a bond angle of 178.34(19)°,^25^ which is more similar to the 178.4(5)° reported for the fluorinated complex (FeTPPF_20_)_2_O (5b)^24^ rather than to the 174.5(1)° of the non-fluorinated complex (FeTPP)_2_O.^26^ In contrast, the structurally related μ-oxo-porphyrinoid–iron complexes μ-oxo-bis[(phthalocyaninato)iron(iii)]^13b^ can adopt a tilted or nearly linear geometry and μ-oxo-bis[(octapropylporphyrazinato)iron(iii)]^13^ has a more bent Fe–O–Fe bond angle of 158.52(7)^13*a*,27^ as compared to the aforementioned porphyrin–iron complexes. The Fe–O bond length of complex 5c (1.7816(6) Å)^25^ is slightly longer than in the related porphyrinoid–iron complexes (FeTPPF_20_)_2_O (5b) (1.775(1) Å),^24^ (FeTPP)_2_O (1.763(1) Å),^26^ and μ-oxo-bis[(octapropylporphyrazinato)iron(iii)] (1.7601(12), 1.7501(12) Å).^13*a*^

Leroy *et al.* investigated the catalytic activity of the porphyrinato–iron(iii) chloride complexes 4a and 4c for epoxidation and hydroxylation reactions.^28*a*^ Very recently, Fujii *et al.* transformed 4b and 4c into the corresponding hypochlorite complexes and studied their catalytic reactivity in epoxidation and chlorination reactions.^28*b*^

**Fig. 2:**
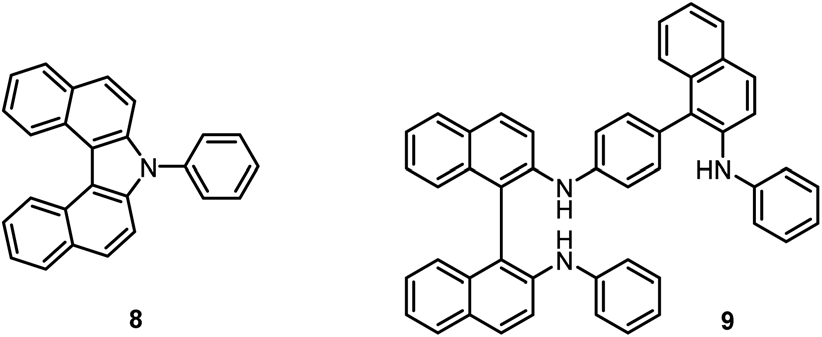
Isolated by-products: carbazole 8 and the twofold coupling product 9.

Previously, we described iron-catalyzed oxidative C–C and C–heteroatom coupling reactions using hexadecafluorophthalo-cyanine–iron(ii) (FePcF_16_) as well as the corresponding μ-oxo-iron(iii) complex ([FePcF_16_]_2_O) as catalysts and air as terminal oxidant.^29^ We have now studied in detail the catalytic activity of the fluorinated porphyrin–iron(iii) complexes 4a–4c, 5b, and 5c in oxidative coupling reactions. We postulated that the strong electron-withdrawing effect of the fluorine atom should increase the catalytic activity of (FeTPPF_28_)_2_O (5c) as compared to the unsubstituted FeTPP system in analogy to our observations with the perfluorinated phthalocyanine–iron complexes. The oxidative C–C homocoupling of *N-*phenyl-2-naphthylamine (6) was selected as model system. Using the previously reported catalyst FePcF_16_ and methanesulfonic acid (MsOH) as additive, the biaryl compound 7 was isolated in 62% yield along with 11% of the carbazole 8 (Table 2, entry 1; Fig. 2).^30^ Initial attempts with the unsubstituted *meso*-tetraphenylporphyrin–iron complex FeTPPCl and the corresponding β-octafluorinated complex 4a in the presence of methanesulfonic acid as additive gave no turnover (Table 2, entries 2 and 3). The perfluorinated complex FeTPPF_28_Cl (4c) gave only traces of the product 7 (entry 4). In conclusion, none of the chloro complexes 4a–4c showed significant catalytic activity in the C–C coupling of 6.

**Table 2:** Optimization of the reaction conditions for the tetraphenylporphyrin–iron-catalyzed oxidative C–C coupling of *N-*phenyl-2-naphthylamine (6)^a^

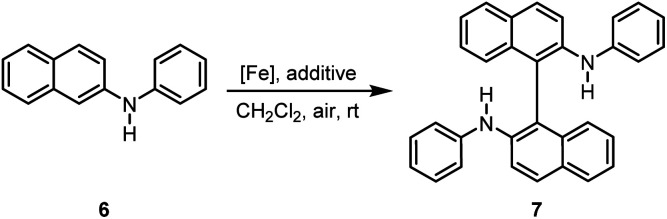				
Entry	[Fe] (mol%)	Additive (mol%)	Time [h]	Yield 7 [%]
1^30^	FePcF_16_ (3.0)	MsOH (10)	0.5	62^b^
2	FeTPPCl (3.0)	MsOH (20)	24	0
3	FeTPPF_8_Cl (4a) (3.0)	MsOH (20)	24	0
4	FeTPPF_28_Cl (4c) (3.0)	MsOH (20)	73	Traces
5	(FeTPPF_28_)_2_O (5c) (1.5)	MsOH (20)	49	6
6^c^	(FeTPPF_28_)_2_O (5c) (1.5)	MsOH (20)	62	12
7	(FeTPPF_28_)_2_O (5c) (1.5)	AcOH (20)	60	5
8	(FeTPPF_28_)_2_O (5c) (1.5)	TFA (20)	40	60^d^
9	(FeTPPF_28_)_2_O (5c) (1.5)	TfOH (20)	48	78^e^
10	(FeTPPF_28_)_2_O (5c) (1.5)	B(C_6_F_5_)_3_ (20)	18	80
11	(FeTPPF_28_)_2_O (5c) (1.5)	BF_3_·OEt_2_ (20)	14	76^f^
12	—	BF_3_·OEt_2_ (20)	24	0
13	(FeTPPF_28_)_2_O (5c) (1.5)	—	24	0
14^g^	(FeTPPF_28_)_2_O (5c) (1.5)	BF_3_·OEt_2_ (20)	5	84

a Reaction conditions: 6 (0.1 mmol), additive, CH_2_Cl_2_ (2 mL), air, rt.

b 8: 11% yield, reisolated 6: 7%.

c Molecular oxygen (1 atm).

d 8: 3% yield, reisolated 6: 37%.

e 9: 9% yield.

f 9: 7% yield.

g Water-free conditions, 3 Å MS, dried compressed air. Pc = phthalocyanine, TPP = tetraphenylporphyrin.

The μ-oxo-iron complexes are assumed to be intermediates in the catalytic cycle of oxidations with porphyrin and phthalocyanine–iron complexes.^13,14,29,31^ Thus, we tested the μ-oxo-iron complex (FeTPPF_28_)_2_O (5c) as catalyst under the same conditions used above for the complexes 4a–4c and obtained the biaryl 7 in 6% yield (entry 5). Performing the reaction under an atmosphere of pure oxygen improved the yield only slightly (entry 6). Variation of the additive improved the yield significantly and revealed that strong Brønsted acids (TFA and TfOH, entries 8 and 9) and the Lewis acids tris(pentafluorophenyl)borane (entry 10) and boron trifluoride diethyl etherate (entry 11) gave the best results. Control experiments confirmed that both the iron catalyst (entry 12) and the Lewis acid (entry 13) are required for the reaction to proceed. The iron-catalyzed oxidative coupling of 6 was generally performed using non-dried solvents under an ambient atmosphere. Finally, we have demonstrated that water-free conditions with dried air and anhydrous solvents led to a further slight increase of the yield of 7 and a decrease of the reaction time (entry 14).

Under the optimized reaction conditions identified above (Table 2, entry 9), the effect of the fluorine substitution and of the axial ligand at the iron atom was investigated using the porphyrin complexes FeTPPCl, 4a–4c, 5b, and 5c as catalysts (Table 3). Two general trends have been observed. The complex FeTPPCl had no catalytic activity at all and the complexes 4a–4c exhibited a very low catalytic activity providing 7 in yields below 10% (Table 3, entries 1–4). However, the μ*-*oxo-iron complexes (FeTPPF_20_)_2_O (5b) and (FeTPPF_28_)_2_O (5c) led to much higher turnover numbers and provided 7 in yields of 57 and 78%, respectively (entries 5 and 6). We concluded that the significant difference in catalytic activity is caused by the different axial ligand. Thus using the chloro–iron complex 4c, we added 3 mol% of silver triflate to the reaction mixture in order to generate *in situ* FeTPPF_28_OTf, which led to much shorter reaction times (4 h instead of 48 h) and afforded the biaryl compound 7 in 89% yield (entry 7).

**Table 3:** Variation of the tetraphenylporphyrin–iron(iii) complexes in the oxidative C–C coupling of *N*-phenyl-2-naphthylamine (6)^a^

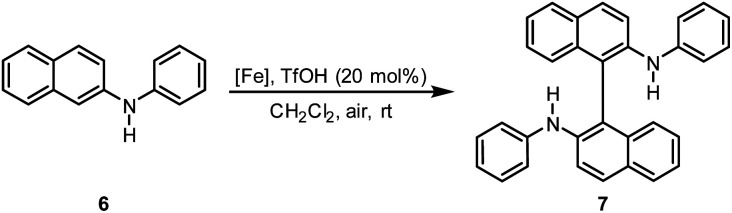			
Entry	[Fe] (mol%)	Yield 7 [%]	Reisolated 6 [%]
1	FeTPPCl (3.0)	0	99
2	FeTPPF_8_Cl (4a) (3.0)	6	93
3	FeTPPF_20_Cl (4b) (3.0)	5	89
4	FeTPPF_28_Cl (4c) (3.0)	7	90
5	(FeTPPF_20_)_2_O (5b) (1.5)	57	21^b^
6	(FeTPPF_28_)_2_O (5c) (1.5)	78	0^c^
7^d^	FeTPPF_28_Cl (4c) (3.0)	89	0^e^

a Reaction conditions: 6 (0.1 mmol), TfOH (20 mol%), CH_2_Cl_2_ (2 mL), air, rt, 48 h.

b 8: 11% yield.

c 9: 9% yield.

d AgOTf (3 mol%), 4 h.

e 9: 8% yield.

**Scheme 1:**
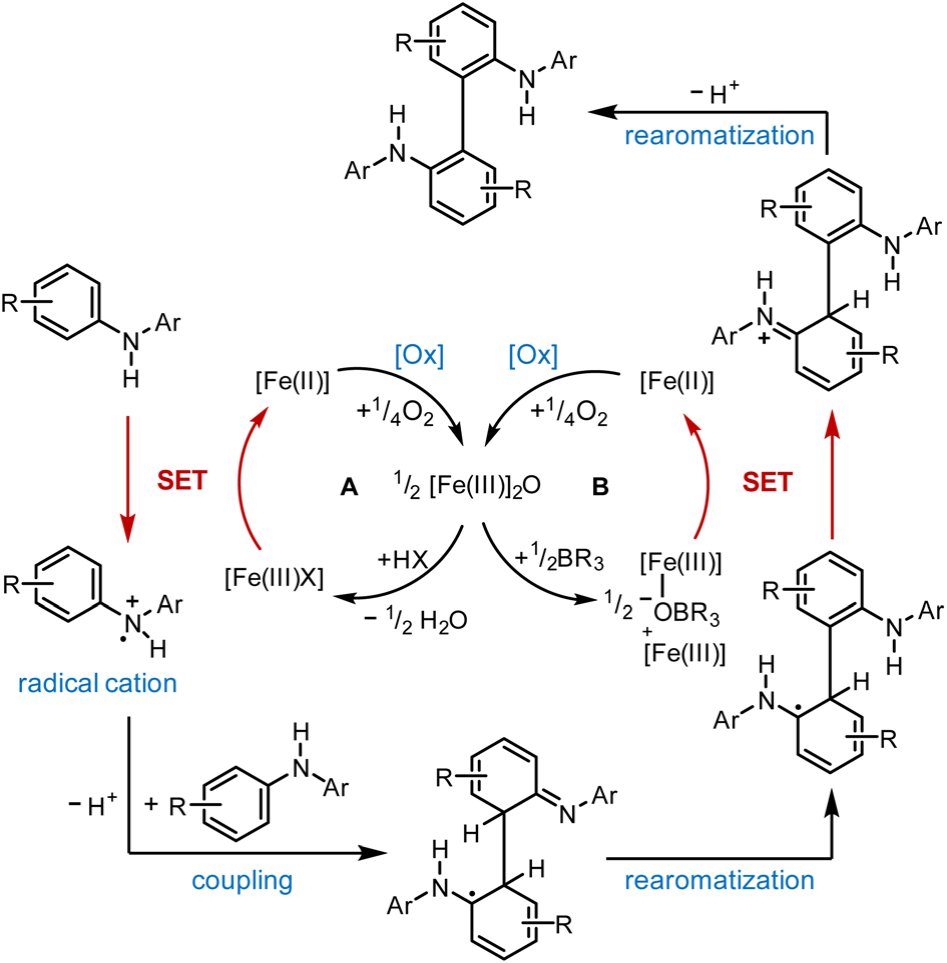
Proposed mechanism for the (FeTPPF_28_)_2_O-catalyzed biaryl coupling. Cycle A: Brønsted acid co-catalyst; cycle B: Lewis acid co-catalyst. X = OTf; [Fe(iii)]_2_O = 5c.

Based on our experimental findings, we postulate the following mechanism for the (FeTPPF_28_)_2_O-catalyzed oxidative coupling considering the strong influence of the additive (Scheme 1). The reaction is believed to be initiated by an SET oxidation followed by coupling and proton loss.^32^

**Fig. 3:**
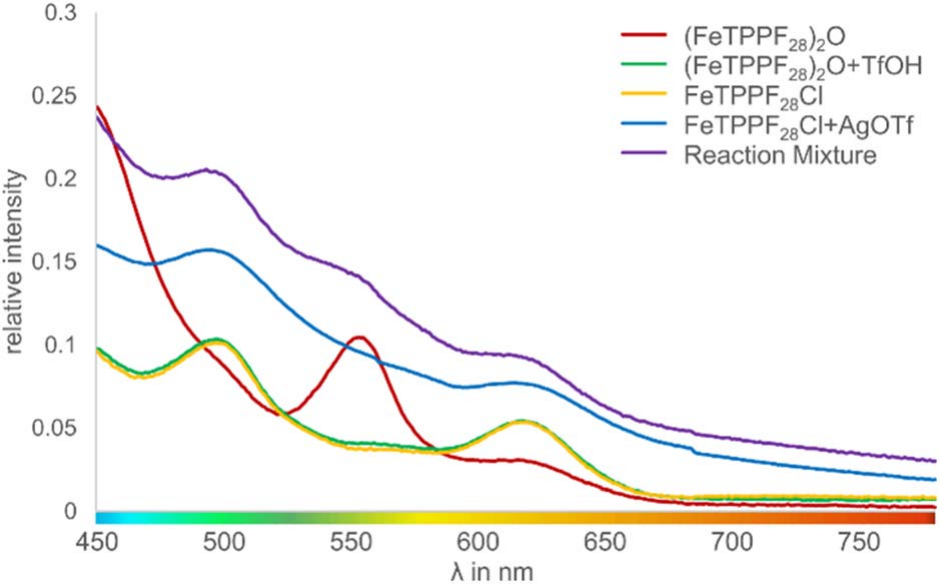
UV-vis spectra of different iron species involved in the oxidative coupling reaction (intensity of the Soret peak normalized to 1; Fig. S1†).

The key step is an SET from the substrate to the iron(iii) complex, as previously observed by Baciocchi *et al.* for the oxidation of *N*,*N*-dimethylanilines with FeTPPF_20_Cl (4b).^12*b*^ The SET process is much more efficient with strongly electron-deficient iron(iii) complexes. Chen *et al.* found that *in situ* exchange of the axial ligand from chloride to triflate enhances the reactivity of porphyrin–iron(iii) complexes for oxidation reactions significantly since triflate is a weaker donor than chloride.^33^ In order to rationalize our experimental findings described above, we followed the iron-catalyzed oxidative coupling by UV-vis experiments (Fig. 3 and S1†).

We observed that the μ-oxo complex (FeTPPF_28_)_2_O (5c) is rapidly hydrolyzed by strong Brønsted acids. After addition of TfOH to a solution of 5c, the characteristic peak at 553 nm disappeared whereas two peaks at 497 and 618 nm emerged, which are assigned to the complex FeTPPF_28_OTf. Alternatively, the latter complex can be generated *in situ* by reaction of FeTPPF_28_Cl (4c) with silver trifluoromethanesulfonate. The UV-vis spectrum of the reaction mixture with 5c as catalyst and TfOH as additive showed after 1 hour all three peaks, indicating that both porphyrin–iron(iii) triflate and the μ-oxo complex 5c are present. Thus, using the μ-oxo complex (FeTPPF_28_)_2_O (5c) in combination with a strong acid (TfOH) as catalyst generates a catalytic system more reactive than FeTPPF_28_Cl (4c) (Table 3, entries 4 *versus* 6). Additional support derives from the increase in catalytic activity observed by exchange of the chloro against the triflato ligand (Table 3, entries 4 and 7).

We have studied the catalytic activity of (FeTPPF_28_)_2_O (5c) for the oxidative coupling of a selection of diarylamines 10a–10c (Table 4). The variation of the additive showed that the Lewis acid BF_3_·OEt_2_ was more efficient than the previously used Brønsted acids (Table S1†). Using these modified conditions, the coupling of 10a–10c proceeded more slowly but gave improved yields compared to our previous results using FePcF_16_ as catalyst.^29*a*^

**Table 4:** (FeTPPF_28_)_2_O-catalyzed oxidative C–C coupling of the diarylamines 10a–10c^a^

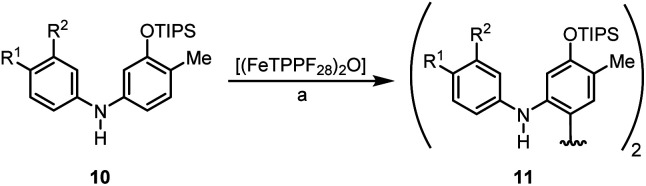					
Entry	Diarylamine	R^1^	R^2^	t [h]	Yield 11 [%]
1	10a	H	H	24	70^b^
2	10b	H	OPiv	20	75
3	10c	OPiv	H	22	78

a Reaction conditions: 10 (0.1 mmol), (FeTPPF_28_)_2_O (5c) (1.5 mol%), BF_3_·OEt_2_ (20 mol%), CH_2_Cl_2_ (2 mL), air, rt.

b Reisolated 10a: 8%.

We then explored the possibility to achieve an asymmetric catalytic oxidative coupling of 10d to the atropisomeric biaryl compound 11d using (FeTPPF_28_)_2_O (5c) as catalyst (Table 5). 1,1′-Biaryl-2,2′-phosphoric acids have been established as efficient chiral catalysts for asymmetric catalysis by Akiyama, Terada, and List.^34^ Recently, we have shown that oxidation of 10d with FePcF_16_ as catalyst in the presence of 10 mol% of the chiral phosphoric acid (*R*)-12 as co-catalyst afforded 11d in 71% yield and 90% *ee* (Table 5, entry 1).^30^ The chiral phosphate counter-ion was believed to direct the asymmetric coupling of the radical cation generated from 10d by an initial single-electron transfer. Using 5c as catalyst in the presence of 20 mol% of (*R*)-12 led to the biaryl compound 11d in 96% *ee* (Table 5, entry 3).

**Table 5:** Asymmetric (FeTPPF_28_)_2_O-catalyzed oxidative C–C coupling^a^

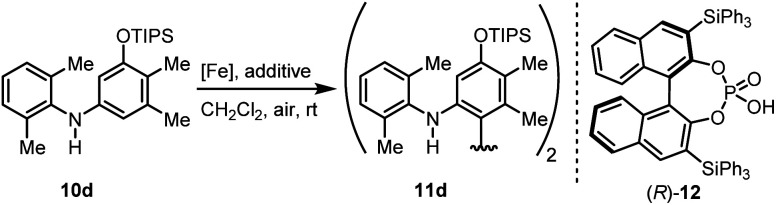					
Entry	[Fe] (mol%)	Additive (mol%)	t [h]	Yield 11d [%]	*ee* [%]^b^
1^30^	FePcF_16_ (3.0)	(*R*)-12 (10)	24	71^c^	90
2	(FeTPPF_28_)_2_O (1.5)	BF_3_·OEt_2_ (20)	23	71	0
3	(FeTPPF_28_)_2_O (1.5)	(*R*)-12 (20)	72	64	96

a Reaction conditions: 10d (0.1 mmol), [Fe], additive, CH_2_Cl_2_ (2 mL), air, rt.

b Determined by chiral HPLC (see: Fig. S2 and S3†).

c Reisolated 10d: 18%.

**Scheme 2:**
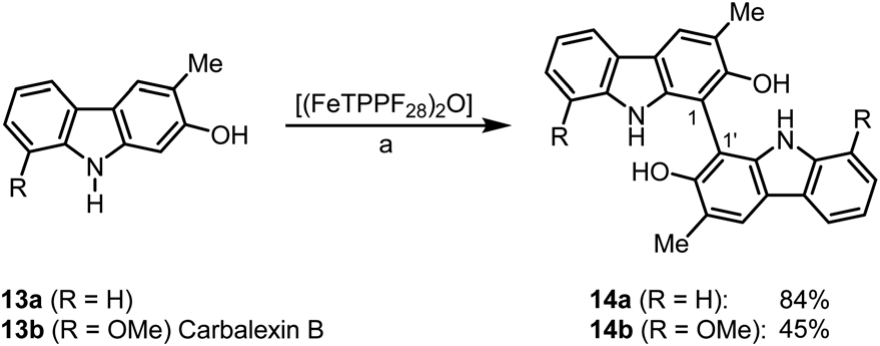
(FeTPPF_28_)_2_O-catalyzed synthesis of 1,1′-bicarbazoles. Reaction conditions: a) 13 (0.15 mmol), (FeTPPF_28_)_2_O (5c) (1.5 mol%), BF_3_·OEt_2_ (20 mol%), CH_2_Cl_2_ (5 mL), air, rt.

**Scheme 3:**
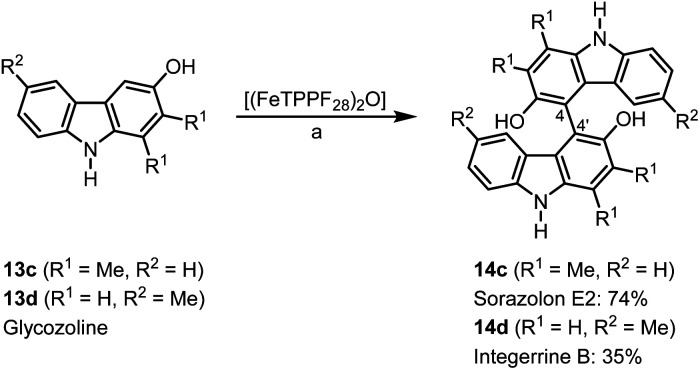
(FeTPPF_28_)_2_O-catalyzed synthesis of 4,4′-bicarbazoles. Reaction conditions: a) 13 (0.15 mmol), (FeTPPF_28_)_2_O (5c) (1.5 mol%), BF_3_·OEt_2_ (20 mol%), CH_2_Cl_2_ (15 mL), air, rt.

**Fig. 4:**
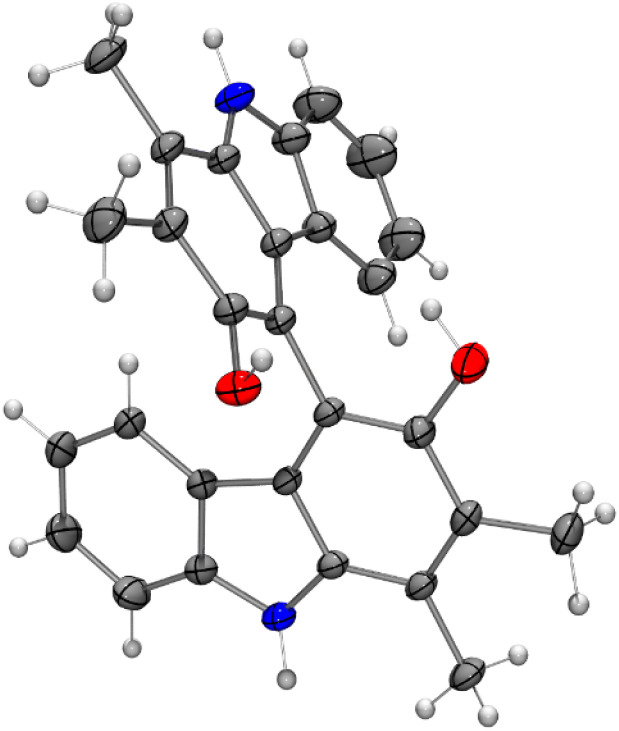
Molecular structure of sorazolon E2 (14c) in the crystal (thermal ellipsoids are shown at the 50% probability level).

Bicarbazoles are an important class of biologically active natural products which can be prepared by C–H/C–H coupling reactions.^35,36^ Recently, we described the synthesis of various bicarbazole alkaloids by FePcF_16_-catalyzed oxidative coupling.^37^ Using (FeTPPF_28_)_2_O (5c) as catalyst for the iron-catalyzed oxidative homocoupling of carbazoles offers a broad structural variety of 1,1′-, 3,3′-, and 4,4′-linked bicarbazoles. The oxidative coupling of 2-hydroxy-3-methylcarbazole (13a)^38^ and carbalexin B (13b)^39^ using 1.5 mol% of (FeTPPF_28_)_2_O (5c) as catalyst in the presence of 20 mol% of BF_3_·OEt_2_ provided regioselectively the naturally occurring 1,1′-bicarbazole alkaloid bis-2-hydroxy-3-methylcarbazole (14a)^40^ and biscarbalexin B (14b)^37^ (Scheme 2). 4,4′-Bicarbazoles as natural products have been isolated only recently. Oxidative coupling of 3-hydroxy-1,2-dimethyl-9*H*-carbazole (13c)^41^ in the presence of (FeTPPF_28_)_2_O as catalyst provided sorazolon E2 (14c)^42^ (Scheme 3). The structure of 14c was confirmed by an X-ray crystal structure determination (Fig. 4).^43^ Analogously, the oxidative homocoupling of glycozoline (13d)^44^ led to the first synthesis of integerrine B (14d).^45^ The ^1^H and ^13^C NMR data of synthetic integerrine B (14d) are in excellent agreement with those reported for the natural product (Table S2†).

Due to their physical properties, 3,3′-bicarbazoles represent promising candidates for hole-transporting materials in organic light-emitting diodes (OLEDs).^46^ Previous procedures for the synthesis of 3,3′-bicarbazoles by oxidative homocoupling required stochiometric amounts of iron(iii) chloride,^47^ DDQ,^48^ or rhodium as noble metal catalyst.^49^ Our method using oxygen as terminal oxidant in the presence of (FeTPPF_28_)_2_O (5c) as catalyst enables the first iron-catalyzed oxidative coupling of the carbazoles 15a–15c to the 3,3′-bicarbazoles 16a–16c (Table 6).

**Table 6:** (FeTPPF_28_)_2_O-catalyzed synthesis of 3,3′-bicarbazoles 16^a^

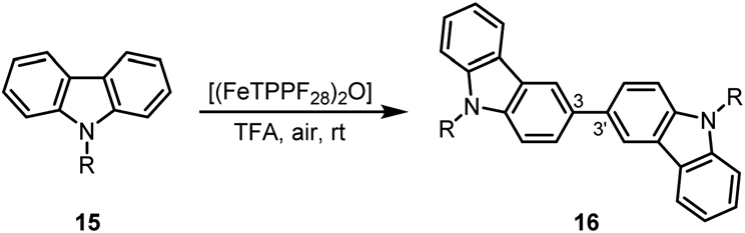					
Entry	Carbazole	R	[Fe] (mol%)	*t* [h]	Yield 16 [%]
1	15a	Me	1.5	1.5	82
2	15b	Bn	1.5	18	71
3	15c	Ph	2.6	4.5	82

a Reaction conditions: 15 (0.1 mmol), (FeTPPF_28_)_2_O (5c), TFA (2–4 mL), air, rt.

Another iron-catalyzed oxidation process recently investigated by our group is the Wacker-type oxidation of olefins to ketones.^50–53^ The oxidation of 2-vinylnaphthalene (17a) to 2-acetylnaphthalene (18a) served as a model system in order to test different phthalocyanine– and porphyrin–iron complexes under an atmosphere of pure oxygen (Table 7). Our previous results showed a much higher catalytic activity of the fluorinated phthalocyanine– and porphyrin–iron complexes as compared to their unsubstituted analogs (Table 7, entries 1–8).^50,51^ Moreover, the importance to use the appropriate silane reducing agent in combination with the corresponding iron catalyst was emphasized.^50–53^ The present results confirm this strong influence of the silane when using the iron complexes FeTPPF_20_Cl (4b) and (FeTPPF_20_)_2_O (5b) as catalysts for the Wacker-type reaction (Table 7, entries 8–11). Based on these previous results, we expected a high catalytic activity for the iron complexes 4c and 5c with the perfluorinated porphyrinato ligand octacosafluoro-*meso*-tetraphenylporphyrin. To our surprise, we had no turnover at all in the oxidation of the olefin 17a using FeTPPF_28_Cl (4c) as catalyst and either triethylsilane or triphenylsilane as reducing agent under otherwise identical reaction conditions (Table 7, entries 12 and 13). Phenylsilane was proven to be the best reducing agent for the iron-catalyzed Wacker-type reaction with tris(1,3-diketonato)iron(iii) complexes as catalysts.^53^ Indeed, using complex 4c as catalyst in combination with phenylsilane provided the ketone 18a in 76% yield along with 9% of the corresponding alcohol 19a (Table 7, entry 14). Basically the same results were obtained for the oxidation of 17a to 18a using the three reducing agents Et_3_SiH, Ph_3_SiH, and PhSiH_3_ in combination with (FeTPPF_28_)_2_O (5c) as catalyst (Table 7, entries 15–17). These results provide further evidence for our mechanistic hypothesis with μ-oxo[diiron(iii)] complexes as intermediates in the catalytic cycle of the Wacker-type oxidation which also applies to the present reaction using complex 5c as catalyst.^51^ Finally, we tested air instead of an atmosphere of pure oxygen as re-oxidant for our iron complex (Table 7, entry 18). Although the reaction time was prolonged, we were delighted that the yield of the desired product 18a increased to 87%, whereas only traces of the alcohol 19a could be detected.

**Table 7:** Porphyrinoid–iron complex-catalyzed Wacker-type oxidation of 2-vinylnaphthalene (17a)^a^

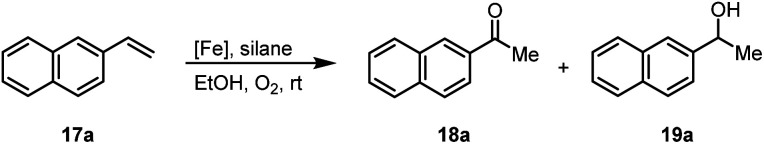					
Entry	[Fe] (mol%)	Silane	*t* [h]	Yield 18a [%]	Yield 19a [%]
1^50^	FePc (5.0)	Et_3_SiH^b^	23	17	8
2^51^	FePcF_16_ (5.0)	Et_3_SiH	6	82	12
3^51^	FePcF_16_ (5.0)	Ph_3_SiH	2.5	85	12
4^51^	FePcF_16_ (5.0)	PhSiH_3_	4	48	13
5^51^	(FePcF_16_)_2_O (2.5)	Et_3_SiH	10	84	13
6^51^	(FePcF_16_)_2_O (2.5)	Ph_3_SiH	4	85	12
7^50^	FeTPPCl (5.0)	Et_3_SiH	6	2	Traces
8^50^	FeTPPF_20_Cl (4b) (5.0)	Et_3_SiH	6	22	4
9	FeTPPF_20_Cl (4b) (5.0)	PhSiH_3_	24	74	Traces
10	(FeTPPF_20_)_2_O (5b) (2.5)	Et_3_SiH	24	0	0
11	(FeTPPF_20_)_2_O (5b) (2.5)	PhSiH_3_	24	78	Traces
12	FeTPPF_28_Cl (4c) (5.0)	Et_3_SiH	24	0	0
13	FeTPPF_28_Cl (4c) (5.0)	Ph_3_SiH	24	0	0
14	FeTPPF_28_Cl (4c) (5.0)	PhSiH_3_	24	76	9
15	(FeTPPF_28_)_2_O (5c) (2.5)	Et_3_SiH	24	0	0
16	(FeTPPF_28_)_2_O (5c) (2.5)	Ph_3_SiH	24	0	0
17	(FeTPPF_28_)_2_O (5c) (2.5)	PhSiH_3_	24	78	10
18^c^	(FeTPPF_28_)_2_O (5c) (2.5)	PhSiH_3_	40	87	Traces

a Reaction conditions: 17a (0.2 mmol), silane (2.0 equiv), EtOH (5 mL), O_2_ (1 atm), rt.

b Et_3_SiH (6.0 equiv.).

c Air instead of pure O_2_.

Using (FeTPPF_28_)_2_O (5c) as catalyst under the optimized reaction conditions, we have tested the Wacker-type oxidation for a range of different olefins 17a–17h (Table 8). A special focus was on those olefins which gave poor results in our previous study with FePcF_16_ or [FePcF_16_]_2_O as catalysts, the cyclic olefins 17e–17g and the aliphatic olefin 17h.^50–52^ For the simple styrene derivatives 17a–17d, the oxidation with 5c as catalyst proceeded smoothly affording the corresponding ketones 18a–18d in yields as high or even slightly better compared to those obtained with FePcF_16_ as catalyst,^50^ albeit longer reaction times were required. The differences between the results with (FeTPPF_28_)_2_O (5c) and the perfluorophthalocyanine–iron complex as catalyst were most pronounced for the oxidation of the more challenging substrates (cyclic olefins and aliphatic olefins). The results with the substrates 17e–17h show that the selectivity of the reaction is shifted significantly towards the ketone at the expense of the alcohol by-product. For example, the Wacker-type oxidation of the nitrochromene 17e catalyzed by (FeTPPF_28_)_2_O (5c) afforded the chroman-4-one 18e in 79% yield along with only 11% of the corresponding alcohol 19e (previous result with FePcF_16_ as catalyst under O_2_: 62% of 18e and 35% of 19e).^52^ Compound 18e represents a synthetic precursor for the pyrano[3,2-*a*]carbazole alkaloid euchrestifoline.^52–54^ Also for the (FeTPPF_28_)_2_O-catalyzed oxidation of the cyanochromene 17f and the dihydronaphthalene 17g to the ketones 18f (93% yield) and 18g (89% yield), we observed a much higher selectivity in favor of the ketones (previous yields with FePcF_16_ as catalyst under O_2_: 65% for 18f and 68% for 18g).^50^ Most strikingly, oxidation of the aliphatic alkene 1-octadecene (17h) using (FeTPPF_28_)_2_O (5c) as catalyst provided 2-octadecanone (18h) in 53% yield,^55^ whereas the corresponding reaction with FePcF_16_ required more of the catalyst (10 mol%), pure oxygen as reoxidant, and elevated temperature (78 °C) but still led preferentially to the alcohol 19h (42% yield) along with 18h (30% yield).^50^ Thus, we have shown that the Wacker-type oxidation of olefins using the new catalyst (FeTPPF_28_)_2_O (5c) proceeds smoothly with ambient air as final oxidant. The present reaction gives higher selectivities in favor of the desired ketones as compared to the corresponding reaction with FePcF_16_ as catalyst which needs pure oxygen as reoxidant to achieve the best turnover numbers.

**Table 8:** Substrate scope and selectivity of the (FeTPPF_28_)_2_O-catalyzed Wacker-type oxidation^a^

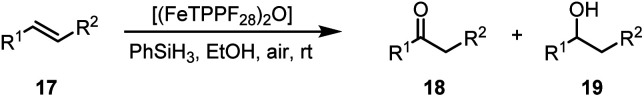				
	17	Time [h]	Yield 18 [%]	Yield 19 [%]
a	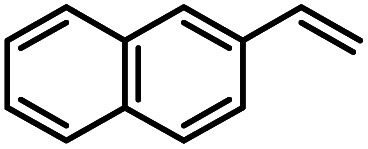	40	87	Traces
b	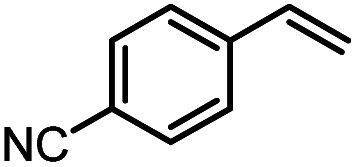	24	90	0
c	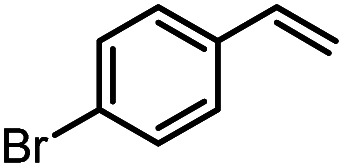	76	89	0
d	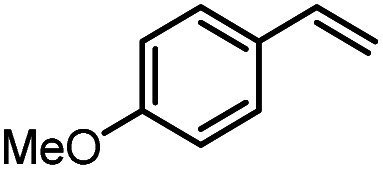	48	85	7
e	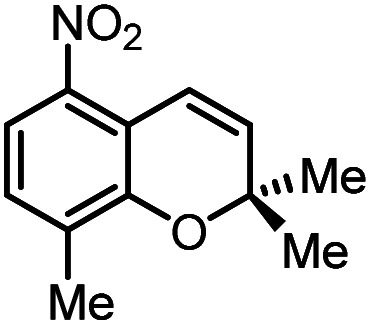	62	79	11
f	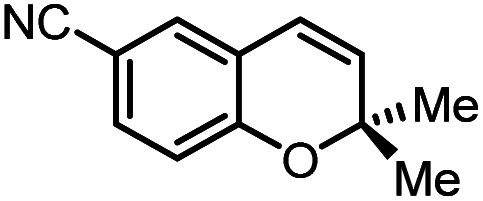	71	93	6
g	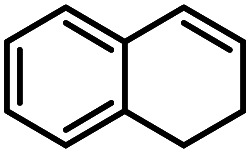	48	89	5
h	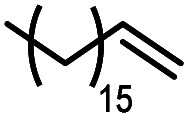	120	53	11

a Reaction conditions: 17 (0.2 mmol), (FeTPPF_28_)_2_O (2.5 mol%), PhSiH_3_ (2.0–5.0 equiv.), EtOH (3 mL), air (1 atm), rt; see SI for details.

## Conclusions

We have described the synthesis of the novel perfluorinated porphyrin–iron complex μ*-*oxo-bis[(octacosafluoro-*meso*-tetraphenylporphyrinato)iron(iii)] [(FeTPPF_28_)_2_O]. The high activity of this catalyst in oxidation reactions has been demonstrated for the biaryl coupling and the Wacker-type reaction. The twofold aryl C–H bond activation was exploited for the oxidative coupling of diarylamines leading to 2,2′-bis(arylamino)-1,1′-biaryls. In the presence of an axially chiral biaryl phosphoric acid as co-catalyst this coupling proceeds in up to 96% *ee*. The (FeTPPF_28_)_2_O-catalyzed oxidative coupling of 2-hydroxy-, 9-alkyl/aryl-, and 3-hydroxycarbazoles affords regioselectively 1,1′-, 3,3′-, and 4,4′-bicarbazoles and has been applied to the synthesis of a variety of bicarbazole natural products including the first synthesis of integerrine B. The Wacker-type oxidation of alkenes, including internal and aliphatic alkenes, previously considered as difficult substrates, with (FeTPPF_28_)_2_O as catalyst in the presence of phenylsilane proceeds at room temperature with air as terminal oxidant and provides the corresponding ketones in high yields. The present findings are paving the way for the development of mild and selective oxidation reactions under biomimetic conditions resembling those of the enzymatic oxidative processes in nature dependent on cytochrome P450 heme proteins.

## Data availability

The data supporting this article have been uploaded as part of the ESI.†

## Author contributions

H.-J. K. secured the funding and directed the project. T. S. and H.-J. K. conceived the project and designed the molecules. T. S. carried out the chemical syntheses, the catalytic experiments, and the structure characterizations. O. K. performed the X-ray crystal structure determinations and analyzed the data. T. S. and H.-J. K. wrote, reviewed, and edited the manuscript.

## Conflicts of interest

There are no conflicts to declare.

## Supplementary Material

SC-014-D2SC06083C-s001

SC-014-D2SC06083C-s002

## References

[cit1] Egorova K. S., Ananikov V. P. (2016). Angew. Chem. Int. Ed..

[cit2] Bauer I., Knölker H.-J. (2015). Chem. Rev..

[cit3] Rendic S., Guengerich F. P. (2015). Chem. Res. Toxicol..

[cit4] Chapple C. (1998). Annu. Rev. Plant Physiol. Plant Mol. Biol..

[cit5] Mizutani M., Sato F. (2011). Arch. Biochem. Biophys..

[cit6] Robins R. J., Chesters N. C. J. E., O′Hagan D., Parr A. J., Walton N. J., Woolley J. G. (1995). J. Chem. Soc., Perkin Trans. 1.

[cit7] Woithe K., Geib N., Zerbe K., Dong B. L., Heck M., Fournier-Rousset S., Meyer O., Vitali F., Matoba N., Abou-Hadeed K., Robinson J. A. (2007). J. Am. Chem. Soc..

[cit8] Bedewitz M. A., Jones A. D., D'Auria J. C., Barry C. S. (2018). Nat. Commun..

[cit9] Ikezawa N., Iwasa K., Sato F. (2008). J. Biol. Chem..

[cit10] Kraus P. F. X., Kutchan T. M. (1995). Proc. Natl. Acad. Sci. U. S. A..

[cit11] Guengerich F. P. (2018). ACS Catal..

[cit12] Augusto O., Beilan H. S., Ortiz De Montellano P. R. (1982). J. Biol. Chem..

[cit13] Colomban C., Kudrik E. V., Tyurin D. V., Albrieux F., Nefedov S. E., Afanasiev P., Sorokin A. B. (2015). Dalton Trans..

[cit14] Yusubov M. S., Celik C., Geraskina M. R., Yoshimura A., Zhdankin V. V., Nemykin V. N. (2014). Tetrahedron Lett..

[cit15] Berger R., Resnati G., Metrangolo P., Weber E., Hulliger J. (2011). Chem. Soc. Rev..

[cit16] Smart B. E. (2001). J. Fluorine Chem..

[cit17] Leroy J., Bondon A., Toupet L., Rolando C. (1997). Chem.–Eur. J..

[cit18] Woller E. K., DiMagno S. G. (1997). J. Org. Chem..

[cit19] Woller E. K., Smirnov V. V., DiMagno S. G. (1998). J. Org. Chem..

[cit20] Adler A. D., Longo F. R., Kampas F., Kim J. (1970). J. Inorg. Nucl. Chem..

[cit21] Rebelo S. L. H., Silva A. M. N., Medforth C. J., Freire C. (2016). Molecules.

[cit22] Tabor E., Połtowicz J., Pamin K., Basąg S., Kubiak W. (2016). Polyhedron.

[cit23] Guo C. C. (1998). J. Catal..

[cit24] Gold A., Jayaraj K., Doppelt P., Fischer J., Weiss R. (1988). Inorg. Chim. Acta.

[cit25] ESI† for μ-oxo-bis[(octacosafluoro-*meso*-tetraphenyl-porphyrinato)iron(III)] (5c) have been deposited with the Cambridge Crystallographic Data Centre (CCDC 2209883)

[cit26] Hoffman A. B., Collins D. M., Day V. W., Fleischer E. B., Srivastava T. S., Hoard J. L. (1972). J. Am. Chem. Soc..

[cit27] Cailler L. P., Clémancey M., Barilone J., Maldivi P., Latour J.-M., Sorokin A. B. (2020). Inorg. Chem..

[cit28] Porhiel E., Bondon A., Leroy J. (1998). Tetrahedron Lett..

[cit29] Fritsche R. F., Theumer G., Kataeva O., Knölker H.-J. (2017). Angew. Chem. Int. Ed..

[cit30] Fritsche R. F., Schuh T., Kataeva O., Knölker H.-J. (2022). Chem.–Eur. J..

[cit31] Ercolani C., Rossi G., Monacelli F. (1980). Inorg. Chim. Acta.

[cit32] Sarhan A. A. O., Bolm C. (2009). Chem. Soc. Rev..

[cit33] Chen G.-Q., Xu Z.-J., Zhou C.-Y., Che C.-M. (2011). Chem. Commun..

[cit34] Akiyama T., Itoh J., Yokota K., Fuchibe K. (2004). Angew. Chem. Int. Ed..

[cit35] (b) Knölker H.-J. and Reddy K. R. in The Alkaloids, ed.: G. A. Cordell, Academic Press, Amsterdam, 2008, vol. 65, pp. 1–430

[cit36] Bringmann G., Tasler S., Endress H., Kraus J., Messer K., Wohlfahrt M., Lobin W. (2001). J. Am. Chem. Soc..

[cit37] Brütting C., Fritsche R. F., Kutz S. K., Börger C., Schmidt A. W., Kataeva O., Knölker H.-J. (2018). Chem.–Eur. J..

[cit38] Bhattacharyya P., Jash S. S., Chowdhury B. K. (1986). Chem. Ind..

[cit39] Pacher T., Bacher M., Hofer O., Greger H. (2001). Phytochemistry.

[cit40] Ito C., Thoyama Y., Omura M., Kajiura I., Furukawa H. (1993). Chem. Pharm. Bull..

[cit41] Knölker H.-J., Bauermeister M., Bläser D., Boese R., Pannek J.-B. (1989). Angew. Chem. Int. Ed..

[cit42] Karwehl S., Jansen R., Huch V., Stadler M. (2016). J. Nat. Prod..

[cit43] ESI† for sorazolon E2 (14c) have been deposited with the Cambridge Crystallographic Data Centre (CCDC 2209872)

[cit44] Forke R., Krahl M. P., Krause T., Schlechtingen G., Knölker H.-J. (2007). Synlett.

[cit45] Cao N.-K., Chen Y.-M., Zhu S.-S., Zeng K.-W., Zhao M.-B., Li J., Tu P.-F., Jiang Y. (2020). Phytochemistry.

[cit46] Sasabe H., Toyota N., Nakanishi H., Ishizaka T., Pu Y.-J., Kido J. (2012). Adv. Mater..

[cit47] Kim Y. E., Kwon Y. S., Lee K. S., Park J. W., Seo H. J., Kim T. W. (2004). Mol. Cryst. Liq. Cryst..

[cit48] Mallick S., Maddala S., Kollimalayan K., Venkatakrishnan P. (2019). J. Org. Chem..

[cit49] Matsumoto K., Toubaru Y., Tachikawa S., Miki A., Sakai K., Koroki S., Hirokane T., Shindo M., Yoshida M. (2020). J. Org. Chem..

[cit50] Puls F., Knölker H.-J. (2018). Angew. Chem. Int. Ed..

[cit51] Puls F., Seewald F., Grinenko V., Klauß H.-H., Knölker H.-J. (2021). Chem.–Eur. J..

[cit52] Puls F., Kataeva O., Knölker H.-J. (2018). Eur. J. Org. Chem..

[cit53] Puls F., Linke P., Kataeva O., Knölker H.-J. (2021). Angew. Chem. Int. Ed..

[cit54] Wu T.-S., Wang M.-L., Wu P.-L. (1996). Phytochemistry.

[cit55] Pangborn M. C., Anderson R. J. (1936). J. Am. Chem. Soc..

