# Supplementary material for: μ-Oxo-bis[(octacosafluoro-meso-tetraphenylporphyrinato)iron(iii)] – synthesis, crystal structure, and catalytic activity in oxidation reactions
Source: Chem Sci. 2022 Dec 7;14(2):257–65. doi: 10.1039/d2sc06083c (PMC9811517; doi:10.1039/d2sc06083c)

Electronic Supplementary Material (ESI) for Chemical Science.
This journal is © The Royal Society of Chemistry 2022

Table of Content

General 2
Structure of the Iron Complexes 3
Synthesis of the Fluoro-Substituted Porphyrin Ligands 4
Synthesis of the Chloro- and μ-Oxo-porphyrin–Iron Complexes 7
UV-Vis Spectra of the Porphyrin−Iron Complexes and of the Reaction Mixture of the Oxidative Coupling 11
Substrate Synthesis 12
Oxidative C–C Coupling of Diarylamines 12
Asymmetric Oxidative C–C Coupling of Diarylamines 16
Oxidative C–C Coupling for the Synthesis of 1,1'- and 4,4'-Bicarbazoles 19
Oxidative C–C Coupling for the Synthesis of 3,3'-Bicarbazoles 24
Wacker-type Oxidation of Olefins 26
References 29
NMR Spectra 30

General

All reactions were carried out in oven-dried glassware. All iron–porphyrin catalyzed reactions were carried out with non-dried solvents in an atmosphere of air, unless stated otherwise. All other reactions were carried out with dry solvents in an atmosphere of argon. Dichloromethane, THF, and toluene were dried by using a solvent purification system (MBraun-SPS). All solvents used for flash chromatography were distilled prior to use. Isohexane was additionally filtrated through a silica gel column before use. All chemicals were used as received from commercial sources, unless stated otherwise. Automated flash chromatography was performed on a Büchi Sepacore system equipped with an UV monitor on silica gel (Acros Organics; 0.035–0.070 mm). TLC analysis was performed on TLC plates from Merck (60 F254) with UV light, Cer(IV), or anisaldehyde solution for visualization. Melting points were measured on a Gallenkamp MPD 350 4 melting-point apparatus. UV spectra were recorded on a PerkinElmer 25 UV/Vis spectrometer, shoulders are labelled with sh. Fluorescence spectra were measured on a Varian Cary Eclipse spectrophotometer. IR spectra were recorded on a Thermo Nicolet Avatar 360 FTIR spectrometer by using the attenuated total reflectance (ATR) technique. NMR spectra were recorded on Bruker DRX 500 and Avance III 600 spectrometers. Chemical shifts δ are reported in ppm with the solvent signal as an internal standard. The following abbreviations have been used: s=singlet, d=doublet, t=triplet, q=quartet, quin=quintet, spt=septet, m=multiplet, and br=broad. EI mass spectra were recorded by GC-MS coupling on an Agilent Technologies 6890 N GC system equipped with a 5973 mass-selective detector (electron impact=70 eV). ESI mass spectra were recorded on a Bruker Esquire LC with an ion-trap detector. Positive and negative ions were detected. High Resolution Mass Spectrometry (HRMS) was conducted on a Waters Xevo G2-XS QTOF equipped with a Waters Zspray ESI ionizer. Elemental analyses were measured on a EuroVector EuroEA3000 elemental analyzer. Weight portions are given in percent. High performance liquid chromatography (HPLC) was performed with an Agilent 1100 Series equipped with a Chiralpak IA column (250 mm x 4.6 mm, particle size 5 µm) and a flow rate of 0.6 mL/min at 25 °C. The detection was performed with a diode array UV-Vis detector.

Structure of the Iron Complexes

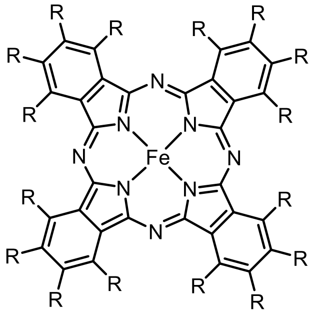

FePc R = H
FePcF$_{16}$ R = F

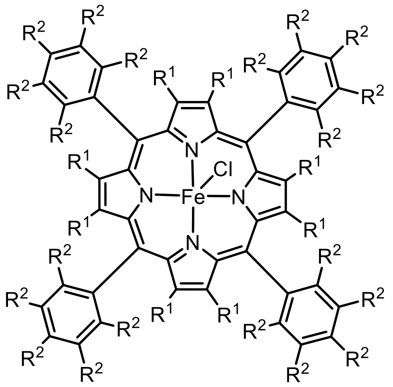

FeTPPCl $R^1 = R^2$ =H
4a FeTPPF$_8$Cl R^1 = F, R^2 = H
4b FeTPPF$_{20}$Cl R^1 = H, R^2 = F
4c FeTPPF$_{28}$Cl $R^1 = R^2$ = F

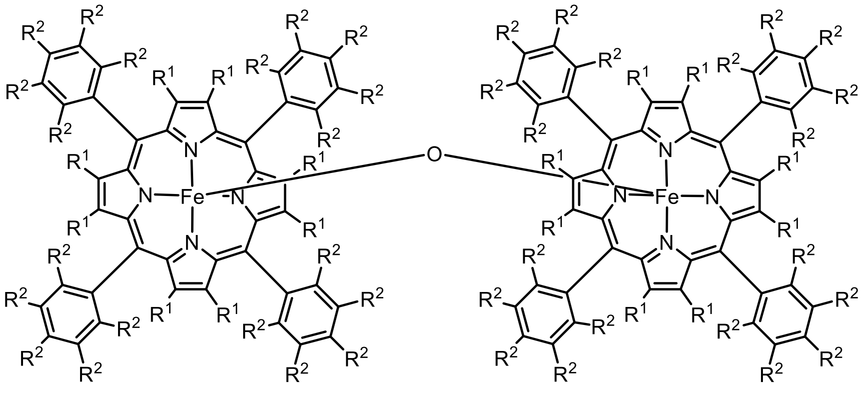

5b (FeTPPF$_{20}$)$_2$O R^1 = H, R^2 = F
5c (FeTPPF$_{28}$)$_2$O $R^1 = R^2$ = F

Synthesis of the Fluoro-Substituted Porphyrin Ligands

The following procedure for the synthesis of the β-fluoro-substituted porphyrins H_2TPPF_8 (**3a**) and H_2TPPF_{28} (**3c**) represents a modified version of the synthesis of 3,4-difluoropyrrole (**2a**) and subsequent porphyrin synthesis described by DiMagno et al.[1,2]

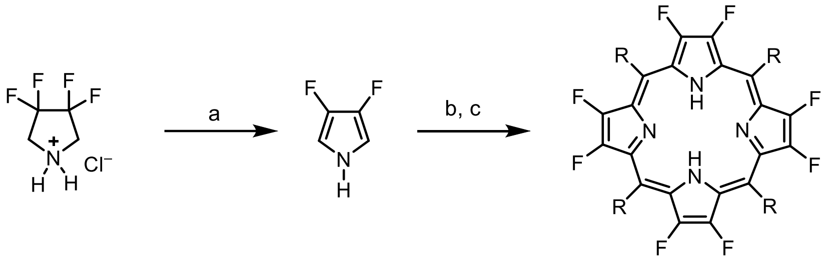

1 **2a** **3a** (H_2TPPF_8) R = Ph
3c (H_2TPPF_{28}) R = C_6F_5

General Procedure I: a) 3,3,4,4-Tetrafluoropyrrolidinium chloride (**1**) (1.0 equiv) was given in a round-bottom Schlenk flask equipped with a magnetic stirring bar and dissolved in dry DMSO (0.1 mL/mg 3,3,4,4-tetrafluoropyrrolidinium chloride) at room temperature. The mixture was cooled to 15 °C in a cold-water bath, then potassium *tert*-butoxide (4.0 equiv) was slowly added under an argon counter flow under constant stirring over a period of 5 minutes. The mixture was stirred for 30 minutes at room temperature, then cooled to 0 °C and quenched by addition of ice water. Due to the reactivity and volatility of the 3,4-difluoro-1*H*-pyrrole (**2a**), the work up and follow up reaction should be done in rapid succession to ensure reproducibility. After all solids were dissolved, the reaction mixture was neutralized by addition of aqueous HCl (1 M) and extracted six times with dichloromethane. The combined organic layers were washed four times with water and once with brine and dried over magnesium sulfate and diluted with dry dichloromethane until the concentration of the crude product was 0.01 M (assuming quantitative yield). The solution was filtered, transferred to a round-bottom Schlenk flask, equipped with a magnetic stirring bar, and then degassed in an argon stream for 15 minutes.

b) The corresponding benzaldehyde (1.1 equiv) and boron trifluoride diethyl etherate were added under vigorous stirring. The reaction mixture was stirred for the given time at room temperature under argon.

c) Pyridine and DDQ were added and the reaction mixture was stirred overnight at room temperature. The suspension was filtered through a short silica gel column and rinsed with dichloromethane until the eluent became colorless. The obtained crude product was further purified as described below to afford the porphyrins **3a** and **c**.

2,3,7,8,12,13,17,18-Octafluoro-5,10,15,20-tetraphenylporphyrin (H_2TPPF_8) (**3a**)

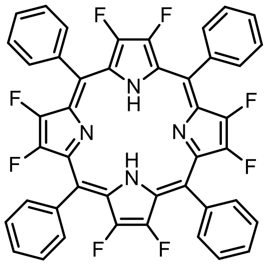

3a

General Procedure I: a) **1** (400 mg, 2.23 mmol, 1.0 equiv), reaction time: 1 h. b) Benzaldehyde (248 µL, 2.45 mmol, 1.1 equiv), $BF_3{\cdot}OEt_2$ (330 µL, 2.67 mmol, 1.2 equiv), reaction time: 2 h. After addition of $BF_3{\cdot}OEt_2$, the reaction mixture turns from yellow to orange and then deep red. c) Pyridine (3.6 mL, 44.6 mmol, 20 equiv), DDQ (658 mg, 2.90 mmol, 1.3 equiv), stirring overnight (black solution and a precipitate was formed). The crude product was washed first three times with isohexane (7 mL), then three times with ethanol (7 mL), and crystallized from toluene/isohexane to provide H_2TPPF_8 (**3a**) (232 mg, 0.310 mmol, 55%) as violet microcrystals. M.p. >300 °C; ^{1}H NMR ($CDCl_3$, 600 MHz): δ=−4.16 (s, 2H), 7.72 (t, J=7.5 Hz, 8H), 7.78 (t, J=7.7 Hz, 4H), 8.05 (d, J=7.1 Hz, 8H) ppm; ^{19}F NMR ($CDCl_3$, 471 MHz): δ=−146.21 (br s, 4F), −141.23 ppm (br s, 4F); IR (ATR): $\tilde{\nu}$=3329, 3056, 3021, 2920, 2850, 2635, 2539, 2056, 2030, 2008, 1917, 1889, 1844, 1771, 1735, 1717, 1678, 1630, 1600, 1577, 1554, 1519, 1458, 1434, 1351, 1268, 1224, 1208, 1167, 1132, 1087, 1032, 1002, 987, 906, 847, 772, 722, 741, 700, 681, 640 cm^{-1}; UV/Vis (CH_2Cl_2): λ_{max}=404, 500, 532, 582, 638 nm; fluorescence (CH_2Cl_2): λ_{ex}=404 nm; λ_{em}=666 nm; MS (ESI, +10 V): m/z =759.4 [M+H]$^+$, (ESI, −100 V): m/z=757.0 [M−H]$^-$; HRMS (ESI): m/z calcd for $C_{44}H_{23}F_8N_4^+$ ([M+H]$^+$): 759.1789; found: 759.1805. The physical and spectroscopic data are in agreement with those reported in the literature.[2]

2,3,7,8,12,13,17,18-Octafluoro-5,10,15,20-tetrakis(pentafluorophenyl)porphyrin (H_2TPPF_{28}) (**3c**)

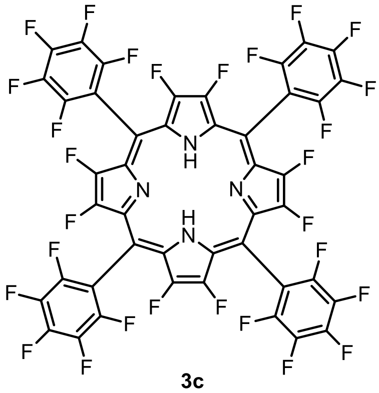

3c

General Procedure I: a) **1** (540 mg, 3.00 mmol, 1.0 equiv), reaction time: 50 min. b) Pentafluorobenzaldehyde (650 mg, 3.30 mmol, 1.1 equiv), $BF_3{\cdot}OEt_2$ (1.5 mL, 3.3 mmol, 4.0 equiv), reaction time: 1 h. After addition of $BF_3{\cdot}OEt_2$, the reaction mixture turns from colorless to deep red over a period of several minutes. c) Pyridine (4.0 mL, 49.6 mmol, 16.5 equiv), DDQ (690 mg, 3.04 mmol, 1.0 equiv), stirring overnight (black solution and a precipitate was formed), reaction time: 18 h. The reaction mixture was filtered through a pad of silica gel and eluted with

CH_2Cl_2/MeOH (95:5). The deep purple solution was concentrated under reduced pressure and purified by column chromatography on silica gel with pure pentane as eluent. The first fraction (deep yellow/orange on the column and in diluted solution) afforded H_2TPPF_{28} (**3c**) (179 mg, 0.160 mmol, 21%) as dark red/violet crystals after removing the solvent in vacuo. Sometimes, the product was contaminated with a green by-product. In this case, the solid product was washed with cold pentane (3×1.0 mL) until the supernatant turns from deep green to pale yellow affords pure **3c**. The product must be dried with care due to its tendency to sublime slowly under reduced pressure. M.p. >300 °C; ^{1}H NMR ($CDCl_3$, 500 MHz): δ=−4.23 ppm (s, 2H); ^{19}F NMR ($CDCl_3$, 471 MHz, 240 K): δ=−160.77 (t, *J*=18.8 Hz, 8F), −149.31 (t, *J*=20.9 Hz, 4F), −147.73 (s, 4 F), −142.72 (s, 4F), −138.24 ppm (m, 8F); IR (ATR): ṽ=3329, 2921, 2852, 2644, 1735, 1698, 1650, 1623, 1558, 1527, 1498, 1473, 1457, 1427, 1350, 1273, 1166, 1130, 1077, 1045, 1030, 984, 964, 943, 790, 754, 720, 676, 649, 624 cm^{-1}; UV/Vis (CH_2Cl_2): λ_{max}=392, 493, 578 nm; fluorescence (CH_2Cl_2): λ_{ex}=392 nm; λ_{em}=642, 703 nm; MS (ESI, +100 V): *m/z*=1119.2 $[M+H]^+$, (ESI, −10 V): m/z=1117.1 $[M-H]^-$; HRMS (ESI): *m/z* calcd for $C_{44}H_3F_{28}N_4^+$($[M+H]^+$): 1118.9905; found: 1118.9904. The physical and spectroscopic data are in agreement with those in the literature.[2]

5,10,15,20-Tetrakis(pentafluorophenyl)porphyrin (H_2TPPF_{20}) (**3b**)

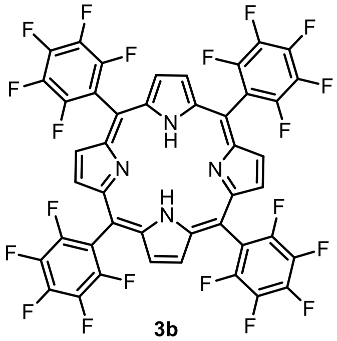

3b

Conditions: This synthesis follows a literature procedure.[3] a) 1*H*-Pyrrole (**2b**) (268 mg, 4.0 mmol, 1.0 equiv) and pentafluorobenzaldehyde (490 µL, 4.0 mmol, 1.0 equiv) were dissolved in dichloromethane (80 mL) in a round-bottom flask equipped with a magnetic stirring bar. Boron trifluoride diethyl etherate (100 µL, 0.8 mmol, 0.2 equiv) was added slowly. The reaction mixture was stirred at room temperature overnight (during the course of the reaction, the colorless solution changes to deep red). b) Then, pyridine (320 µL, 4.0 mmol, 1.0 equiv) and DDQ (908 mg, 4.0 mmol, 1.0 equiv) were added, and the solution was heated under reflux for 2 h. The reaction mixture was then filtered through a short silica gel column and rinsed with dichloromethane until the eluent became colorless. The solvent was removed in vacuo and the residue was adsorbed on silica gel. The crude product was then purified by column chromatography on silica gel with isohexane/dichloromethane (3:2). After removing the solvent in vacuo, the first fraction afforded H_2TPPF_{20} (**3b**) (279 mg, 0.310 mmol, 31%) as dark red/violet crystals. M.p. >300 °C; ^{1}H NMR ($CDCl_3$, 500 MHz): δ=−2.92 (s, 2H), 8.92 ppm (s, 8H); ^{19}F NMR: ($CDCl_3$, 282 MHz): δ=−161.93 (td, *J*=22.9, 7.7 Hz, 8F), −151.83 (t, *J*=21.4 Hz, 4F), −137.13 ppm(dd, *J*=23.2, 7.7 Hz, 8F); IR (ATR): ṽ=3318, 3100, 2919, 2717, 2625, 2539, 1676, 1648, 1558, 1539, 1498, 1482, 1435, 1400, 1342, 1323, 1262, 1246, 1147, 1078, 1044, 984, 917, 831, 806, 770, 754, 723, 698, 636 cm^{-1}; UV/Vis (CH_2Cl_2): λ_{max}=413, 506, 582 nm; fluorescence (CH_2Cl_2): λ_{ex}=392 nm; λ_{em}=640, 706 nm; MS (ESI, +10 V): *m/z*=975.3 $[M+H]^+$; MS (ESI, −50 V): *m/z*=973.2 $[M-H]^-$. HRMS

(ESI): *m/z* calcd for $C_{44}H_{11}F_{20}N_4^+$ ($[M+H]^+$): 975.0659; found: 975.0670. The physical and spectroscopic data are in agreement with those reported in the literature.[3]

Synthesis of the Chloro- and µ-Oxo-porphyrin–Iron Complexes

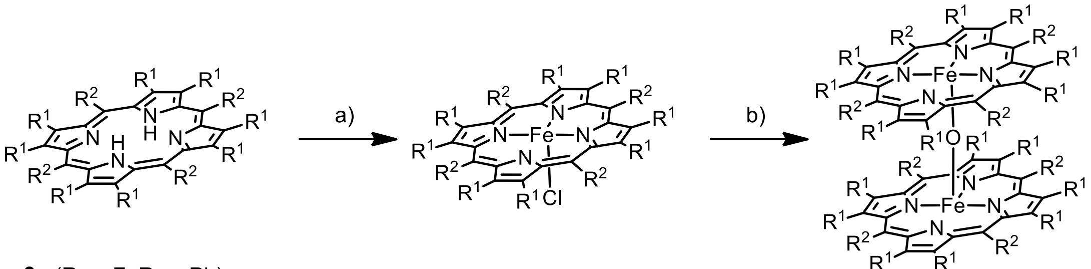

3a (R_1 = F, R_2 = Ph)
3b (R_1 = H, R_2 = C_6F_5)
3c (R_1 = F, R_2 = C_6F_5)

4a (R_1 = F, R_2 = Ph)
4b (R_1 = H, R_2 = C_6F_5)
4c (R_1 = F, R_2 = C_6F_5)

5b (R_1 = H, R_2 = C_6F_5)
5c (R_1 = F, R_2 = C_6F_5)

General Procedure II: a) The porphyrins **3a**–**c** were placed in a sealed tube equipped with a magnetic stirring bar and suspended in acetonitrile (c = 10 mg/mL). Then, iron(II) chloride (10 equiv) was added and the reaction mixture was stirred for 1.5 h at room temperature under air. Subsequently, the reaction mixture was heated to 120 °C and the suspension turned to a dark but clear solution. After 30 minutes, the mixture was cooled to room temperature. Then, additional iron(II) chloride (10 equiv) was added at room temperature. After 1.5 h, the solution was again heated to 120 °C for 30 minutes. After cooling to room temperature, the solution was diluted with dichloromethane and washed three times with 1 M aqueous HCl. The organic layer was dried over magnesium sulfate and the solvent was removed in vacuo. The crude products were purified by washing to provide the chloro-porphyrin–iron complexes **4a**–**c**.
b) The chloro-porphyrin–iron complexes **4a**–**c** were eluted through an activated alumina pad with CH_2Cl_2/MeOH (95:5) under ambient air until the eluent became colorless. The solvent was removed in vacuo to afford the µ-oxo-porphyrin–iron complexes **5b**–**c**.

(2,3,7,8,12,13,17,18-Octafluorotetraphenylporphyrinato)iron(III) chloride ($FeTPPF_8Cl$) (**4a**)

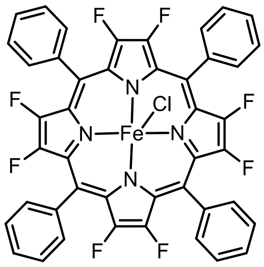

4a

General Procedure II: a) H_2TPPF_8 (**3a**) (50.0 mg, 66 µmol, 1.0 equiv). Workup: the crude product was washed three times with cold acetone. Yield: 59% (33.2 mg, 39.0 µmol) $FeTPPF_8Cl$ (**4a**), green solid. M.p. >300 °C; IR (ATR) ṽ=3068, 3023, 2162, 1991, 1914, 1871, 1717, 1658, 1474, 1442, 1376, 1334, 1233, 1183, 1155, 1075, 1009, 909,

873, 790, 753, 698, 666, 621 cm^{-1}; UV/Vis (CH_2Cl_2): λ_{max}=396 nm; MS (ESI, +50 V): *m/z*=812.2 [M−Cl]$^+$; elemental analysis calcd (%) for $C_{44}H_{20}ClF_8FeN_4$: C 62.32, H 2.38, N 6.61; found: C 62.52, H 2.47, N 6.55.

[5,10,15,20-Tetrakis(pentafluorophenyl)porphyrinato]iron(III) chloride ($FeTPPF_{20}Cl$) (**4b**)

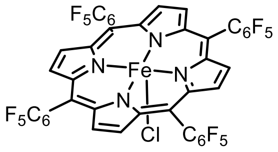

4b

General Procedure II: a) H_2TPPF_{20} (**3b**) (50.1 mg, 51 µmol, 1.0 equiv). Workup: the crude product was washed three times with isohexane. Yield: 96% (52.1 mg, 49 µmol) $FeTPPF_{20}Cl$ (**4b**), deep green powder. M.p. >300 °C; IR (ATR) ṽ=2021, 1649, 1623, 1558, 1512, 1483, 1459, 1421, 1363, 1336, 1209, 1161, 1082, 1052, 985, 936, 837, 806, 758, 725, 706 cm^{-1}; UV/Vis (CH_2Cl_2): λ_{max}=351, 410, 503, 630 nm; MS (ESI, +50 V): *m/z*=1028.4 [M−Cl]$^+$; MS (ESI, −25 V): *m/z*=1146.2 [M+2OAc]$^-$; elemental analysis calcd (%) for $C_{44}H_8ClF_{20}FeN_4$: C 49.68, H 0.76, N 5.27; found: C 49.35, H 0.50, N 5.55.

[2,3,7,8,12,13,17,18-Octafluoro-5,10,15,20-tetrakis(pentafluorophenyl)porphyrinato]iron(III) chloride ($FeTPPF_{28}Cl$) (**4c**)

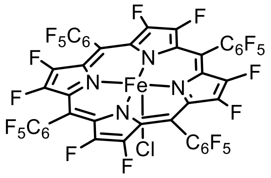

4c

General Procedure II: a) H_2TPPF_{28} (**3c**) (50.2 mg, 45 µmol, 1.0 equiv). Work-up: the crude product was washed three times with isohexane. Yield: 92% (49.7 mg, 45 µmol) $FeTPPF_{28}Cl$ (**4c**), deep green powder. M.p. >300 °C; IR (ATR) ṽ=2130, 2056, 2026, 1842, 1735, 1673, 1653, 1557, 1499, 1477, 1434, 1386, 1335, 1179, 1146, 1052, 986, 964, 803, 730, 679, 635 cm^{-1}; UV/Vis (CH_2Cl_2): λ_{max}=365, 403, 496, 618 nm; MS (ESI, +25 V): *m/z*=1172.1 [M−Cl]$^+$; MS (ESI, −25 V): *m/z*=1289.5 [M+2OAc]$^-$; MS (ESI, −100 V): *m/z*=1230.6 [M+OAc]$^-$; elemental analysis calcd (%) for $C_{44}ClF_{28}FeN_4$: C 43.76, N 4.64; found: C 43.40, N 4.78.

µ-Oxo-bis{[5,10,15,20-tetrakis(pentafluorophenyl)porphyrinato]iron(III)} $(FeTPPF_{20})_2O$ (**5b**)

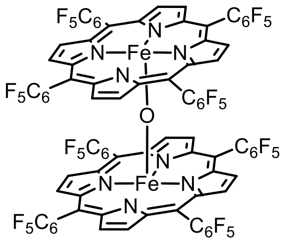

5b

General Procedure II: a) H_2TPPF_{28} (**3b**) (50.0 mg, 51 µmol, 1.0 equiv). b) The crude product **4b** from step a) was eluted through alumina. The color of the solution changes from deep green (chloro-porphyrin–iron complex) to deep red. The column was rinsed with dichloromethane/MeOH (95:5) until the eluent became colorless. Yield: 98% (51.8 mg, 50 µmol) $(FeTPPF_{20})_2O$ (**5b**), deep red crystals. R_f=0.85 (CH_2Cl_2/MeOH 95:5); M.p. >300 °C; IR (ATR) ṽ=3636, 2807, 1976, 1648, 1558, 1538, 1483, 1421, 1378, 1335, 1208, 1160, 1080, 1048, 984, 935, 837, 804, 758, 705 cm^{-1}; UV/Vis (CH_2Cl_2): λ_{max}=351, 410, 502, 630 nm; MS (ESI, +50 V): *m/z*=1028.4 $[0.5(M{-}O)]^+$; MS (ESI, −100 V): *m/z*=1187.0 $[0.5(M{-}O)+OAc]^-$; elemental analysis calcd (%) for $C_{88}H_{16}F_{40}Fe_2N_8O$: C 50.99, H 0.78, N 5.41; found: C 50.73, H 0.87, N 5.62.

µ-Oxo-bis{[2,3,7,8,12,13,17,18-octafluoro-5,10,15,20-tetrakis(pentafluorophenyl)porphyrinato]iron(III)]} $(FeTPPF_{28})_2O$ (**5c**)

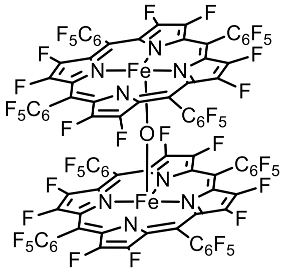

5c

General Procedure II: a) H_2TPPF_{28} (**3c**) (175 mg, 156 µmol, 1.0 equiv). b) The crude product **4c** from step a) was eluted through alumina. The color of the solution changes from deep green to deep red. The column was rinsed with dichloromethane/MeOH (95:5) until the eluent became colorless. Yield: >99% (184.6 mg, 78 µmol) $(FeTPPF_{28})_2O$ (**5c**), deep red crystals. R_f=0.9 (CH_2Cl_2/MeOH 95:5); M.p. >300 °C; IR (ATR) ṽ=2056, 2029, 2008, 1845, 1772, 1735, 1717, 1698, 1677, 1654, 1622, 1556, 1498, 1477, 1432, 1381, 1336, 1260, 1179, 1050, 988, 963, 851, 803, 731, 678, 634 cm^{-1}; UV/Vis (CH_2Cl_2): λ_{max}=367, 497, 550, 618 nm; MS (ESI, +75 V): *m/z*=1172.1 $[0.5((M{-}O)]^+$; MS (ESI, −100 V): *m/z*=1230.6 $[0.5(M{-}O)+OAc]^-$; elemental analysis calcd (%) for $C_{88}F_{56}Fe_2N_8O$: C 44.77, N 4.75; found: C 45.04, N 4.67.

Crystallographic data for μ-Oxo-bis{[2,3,7,8,12,13,17,18-octafluoro-5,10,15,20-tetrakis(pentafluorophenyl)-porphyrinato]iron(III)]} $(FeTPPF_{28})_2O$ (**5c**):

Crystallization of **5c** from dichloromethane afforded single crystals suitable for X-ray analysis.

$C_{88}F_{56}Fe_2N_8O_1$ + $2H_2O$, M = 2396.69 g mol^{-1}, crystal size: 0.255 × 0.274 × 0.291 mm, tetragonal, space group *P*4/*ncc*, a = 29.489(7), b = 29.489, c = 20.678(5) Å, V = 17982 (10) Å^3, Z = 8, ρ_{calcd} = 1.771 g/cm^3, μ = 0.495 mm^{-1}, λ = 0.71073 Å, T = 150(2) K, θ range: 2.09–26.50°, reflections collected: 116202, independent: 9318 (R_{int} = 0.0590), 716 parameters. The structure was solved by direct methods and refined by full-matrix least-squares on F^2; final R indices [I>2σ(I)]: R_1 = 0.0461, wR_2 = 0.1568; maximal residual electron density: 0.835 eÅ^{-3}; CCDC 2209883 contains the supplementary crystallographic data for this paper. These data can be obtained free of charge from The Cambridge Crystallographic Data Centre.

UV-Vis Spectra of the Porphyrin−Iron Complexes and of the Reaction Mixture of the Oxidative Coupling

All spectra were recorded in a solution of CH_2Cl_2 (Fig. S1). The reaction mixture was prepared using 50 mol% $(FeTPPF_{28})_2O$ (**5c**) (2.38 mg, 1.00 µmol), 1.0 eq. TfOH (300 µg, 2.00 µmol), and 1.0 eq. *N*-phenyl-2-naphthylamine (430 µg, 2.00 µmol). The relative intensity of the spectra was set to 1.0 (Soret peak).

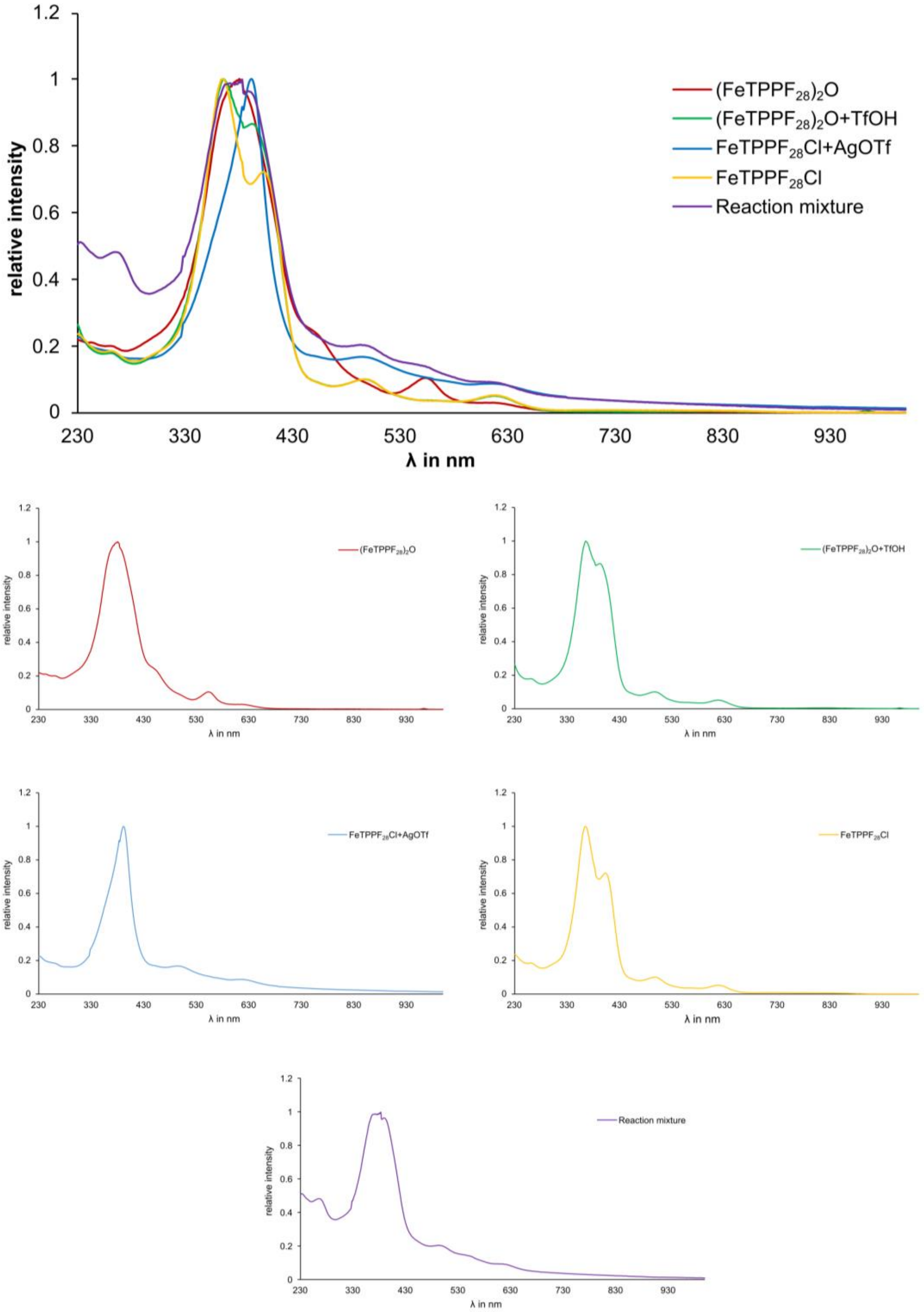

Fig. S1 UV-Vis spectra of different iron species proposed for the oxidative coupling reaction (overlayed and single spectra, 230–1000 nm).

Substrate Synthesis

The diarylamines **10a**–**d** were prepared according to our previous report.[4]

3-Hydroxy-1,2-dimethyl-9*H*-carbazole (**13c**) (sorazolon E) was published by us previously as an intermediate for the synthesis of 4-deoxycarbazomycin B.[5,6]

9-Methyl-9H-carbazole (**15a**) was synthesized according to a literature procedure.[7]

9-Benzyl-9*H*-carbazole (**15b**)

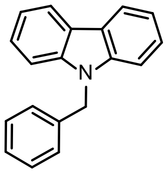

15b

Sodium hydride (96 mg, 60 wt% in mineral oil, 2.4 mmol) was given in a round-bottom flask equipped with a magnetic stirring bar and then dry DMF (5 mL) was added. A solution of 9*H*-carbazole (334 mg, 2.00 mmol, 1.0 equiv) in DMF (5 mL) was added slowly to the suspension at 0 °C and the reaction mixture was stirred for 15 min at 0 °C. Then, benzyl bromide (411 mg, 2.40 mmol) was added slowly at 0 °C, and the yellow solution became colorless. After 1 h, water (15 mL) was added to the reaction mixture. The product precipitated as a colorless microcrystalline powder, which was filtrated and washed thoroughly with water and cold ethanol. **15b**: Colorless powder, 496 mg (1.93 mmol, 96%) yield. ^{1}H NMR (600 MHz, $CDCl_3$) δ=5.53 (s, 2H), 7.15 (d, J=7.0 Hz, 2H), 7.21−7.29 (m, 5H), 7.38 (d, J=8.2 Hz, 2H), 7.44 (ddd, J=8.1, 7.1, 1.1 Hz, 2H), 8.14 (d, J=7.7 Hz, 2H). ^{13}C NMR and DEPT (151 MHz, $CDCl_3$): δ=46.81 (CH_2), 109.13 (2CH), 119.44 (2CH), 120.63 (2CH), 123.26 (2C), 126.08 (2CH), 126.65 (CH), 127.68 (2CH), 129.01 (2CH), 137.42 (C), 140.91 (2C); MS (EI): *m/z* (%)=257 (32, M$^+$), 180 (5), 166 (15), 152 (4), 140 (12), 91 (100), 65 (13).

Oxidative C–C Coupling of Diarylamines

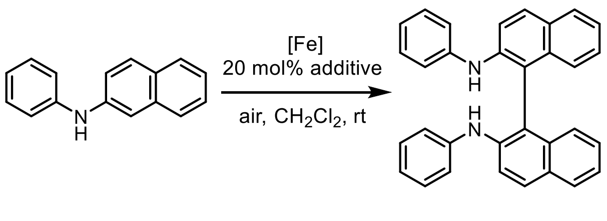

6 **7**

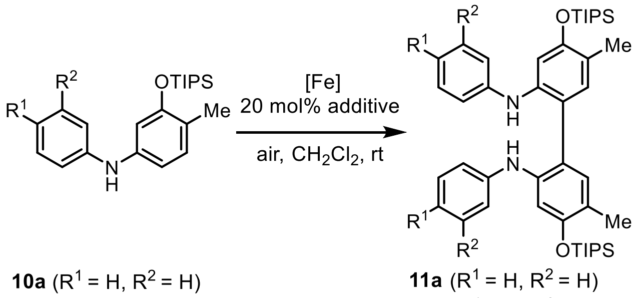

10a (R^1 = H, R^2 = H)
10b (R^1 = H, R^2 = OPiv)
10c (R^1 = OPiv, R^2 = H)

11a (R^1 = H, R^2 = H)
11b (R^1 = H, R^2 = OPiv)
11c (R^1 = OPiv, R^2 = H)

General Procedure III: The diarylamine **6** or **10** (1.0 equiv), iron catalyst (3.0 mol% chloro- or 1.5 mol% µ-oxo complex), and the additive (20 mol%) were placed in a round-bottom flask equipped with a magnetic stirring bar and dissolved in dichloromethane (c ~ 50 mM). To minimize solvent loss by evaporation a splash head was placed on top of the flask and the reaction mixture was stirred vigorously at room temperature under ambient air. After completion of the reaction (TLC analysis), triethylamine (about 10 equiv) was added. The reaction mixture was filtered through a short pad of silica with dichloromethane as eluent. The solvent was removed in vacuo, and the residue was adsorbed on silica gel. The crude product was purified by column chromatography on silica gel.

N^{2},*N*$^{2'}$-diphenyl-(1,1'-binaphthalene)-2,2'-diamine (**7**) and *N*^{2}-phenyl-*N*$^{2'}$-[4-(2-(phenylamino)naphthalen-1-yl]phenyl)-(1,1'-binaphthalene)-2,2'-diamine (**9**)

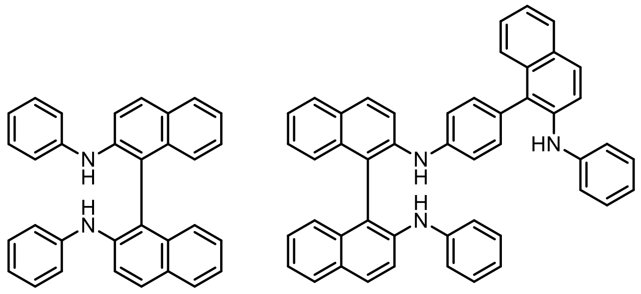

7 **9**

Method 1 (General Procedure III, Lewis acid additive): *N*-Phenylnaphthalen-2-amine (**6**) (21.7 mg, 99.0 µmol), $B(C_6F_5)_3$ (10.3 mg, 20.0 µmol, 20 mol%), $(FeTPPF_{28})_2O$ (**5c**) (3.6 mg, 1.5 µmol, 1.5 mol%), reaction time: 18 h. Column chromatography: isohexane/dichloromethane (3:1). **7**, yield: 80% (17.9 mg, 41.0 µmol); 7% of **6** were reisolated.

Method 2 (General Procedure III, Brønsted acid additive): **6** (21.8 mg, 99.0 µmol), TfOH (3.1 mg, 21 µmol, 21 mol%), $(FeTPPF_{28})_2O$ (**5c**) (3.5 mg, 1.5 µmol, 1.5 mol%), reaction time: 48 h. Column chromatography: isohexane/dichloromethane (3:1). **7**, yield: 78% (18.5 mg, 42 µmol); **9**, yield: 9% (2.1 mg, 3.0 µmol).

Method 3 (water-free conditions): **6** (21.7 mg, 99 µmol, 1.0 equiv), $(FeTPPF_{28})_2O$ (**5c**) (3.6 mg, 1.5 µmol, 1.5 mol%) were placed in a Schlenk flask equipped with a magnetic stir bar and a septum. The flask was evacuated and filled with dry air (compressed air dried with calcium chloride), in three repeated cycles. The starting materials were dissolved in dry CH_2Cl_2 (2.5 mL), then $BF_3{\cdot}OEt_2$ (2.5 µL, 0.02 mmol, 0.2 equiv) was added. The reaction mixture was stirred vigorously for 5 h. Column chromatography: isohexane/dichloromethane (3:1). **7**, yield: 84% (17.9 mg, 41 µmol).

7: Colorless solid; ^{1}H NMR (600 MHz, [D$_6$]acetone): δ=6.46 (br s, 2H), 6.86 (t, *J*=7.4, 2H), 7.04–7.09 (m, 6H), 7.13–7.17 (m, 4H), 7.20 (ddd, *J*=8.3, 6.9, 1.4 Hz, 2H), 7.28 (ddd, *J*=8.0, 6.9, 1.2 Hz, 2H), 7.68 (dd, *J*=9.0, 3.2 Hz, 2H), 7.88 (d, *J*=8.0 Hz, 2H), 7.93 ppm (d, *J*=9.1 Hz, 2H); ^{13}C NMR und DEPT (151 MHz, [D$_6$]acetone): δ=118.35 (C), 118.39 (C) 119.45 (CH), 119.47 (CH), 120.18 (2CH), 120.24 (2CH), 122.20 (2CH), 124.06 (2CH), 125.26 (2CH), 127.41 (2CH), 129.09 (2CH), 129.85 (4CH), 129.94 (2CH), 130.57 (2C), 135.22 (2C), 141.58 (C), 141.67 (C), 144.15 (C), 144.24 ppm (C); ^{1}H NMR (600 MHz, [D$_6$]DMSO): δ=6.69 (s, 2NH), 6.79 (tt, *J*=11.0, 1.0 Hz, 2H), 6.97 (d, *J*=9.1 Hz, 2H), 6.98–7.01 (m, 4H), 7.10–7.15 (m, 4H), 7.18 (ddd, *J*=8.4, 6.9, 1.4 Hz, 2H), 7.27 (ddd, *J*=8.1, 6.9,

1.2 Hz, 2H), 7.61 (d, *J*=9.0 Hz, 2H), 7.87 (d, *J*=7.9 Hz, 2H), 7.93 (d, *J*=9.0 Hz, 2H); ^{13}C NMR und DEPT (151 MHz, [D$_6$]DMSO): *δ*=118.29 (4CH), 118.87 (2C), 119.48 (2CH), 120.43 (2CH), 123.14 (2CH), 124.20 (2CH), 126.32 (2CH), 128.05 (2CH), 128.77 (2CH), 128. 84 (4CH), 129.36 (2C), 133.77 (2C), 140.23 (2C), 143.50 ppm (2C); MS (ESI, +10 V): *m/z*=437.0 [M+H]$^+$.

9: Colorless solid. ^{1}H NMR (CDCl$_3$, 600 MHz): *δ* (ppm)=5.66 (br s, 3H), 6.89–6.97 (m, 2H), 6.99 (d, *J*=8.3 Hz, 2H), 7.02 (d, *J*=8.3 Hz, 2H), 7.05–7.08 (m, 1H), 7.09–7.13 (m, 1H), 7.14–7.21 (m, 6H), 7.22–7.29 (m, 4H), 7.29–7.32 (m, 2H), 7.32–7.39 (m, 3H), 7.57 (d, *J*=9.0 Hz, 1H), 7.71 (d, *J*=9.0 Hz, 1H), 7.74 (d, *J*=8.7 Hz, 1H), 7.75–7.79 (m, 1H), 7.85 (d, *J*=9.0 Hz, 1H), 7.88 (dd, *J*=8.1, 3.6 Hz, 2H), 7.91 (d, *J*=9.0 Hz, 1H), 7.95 (d, *J*=9.0 Hz, 1H); ^{13}C NMR und DEPT (CDCl$_3$, 151 MHz): *δ* (ppm)=116.54 (C), 117.17 (C), 118.08 (CH), 118.27 (CH), 118.70 (2CH),118.77 (CH), 119.50 (CH), 119.79 (2CH), 119.82 (CH), 121.46 (CH), 122.22 (2CH), 123.28 (CH), 123.57 (CH), 123.75 (CH), 124.57 (CH), 124.65 (CH), 124.90 (C), 125.05 (CH), 126.23 (CH), 127.11 (CH), 127.15 (CH), 127.93 (CH), 128.13 (CH), 128.29 (2CH), 129.29 (C), 129.30 (2CH), 129.31 (2CH), 129.41 (C), 129.52 (2CH), 129.70 (C), 131.82 (2CH), 134.01 (C), 134.03 (C), 134.05 (C), 138.18 (C), 139.75 (C), 140.33 (C), 142.28 (C), 142.58 (C), 143.51 (C); MS (ESI, +10 V): *m/z*=654.4 [M+H]$^+$; MS (ESI, −25 V): *m/z*=652.1 [M−H]$^-$.

5,5'-Dimethyl-*N*^{2},*N*$^{2'}$-diphenyl-4,4'-bis[(triisopropylsilyl)oxy]-(1,1'-biphenyl)-2,2'-diamine (**11a**)

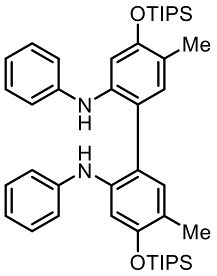

11a

General Procedure III: 35.9 mg (101 µmol) **10a**, additive: 2.8 mg (20 µmol, 20 mol%) $BF_3 \cdot OEt_2$, 3.5 mg (1.5 µmol, 1.5 mol%) $(FeTPPF_{28})_2O$ (**5c**), reaction time: 24 h. Column chromatography: isohexane/EtOAc (5:1). Colorless solid; **11a**, yield: 70% (24.9 mg, 35 µmol); 8% of starting material **10a** (2.9 mg, 8 µmol) were reisolated. **11a**: ^{1}H NMR (500 MHz, CDCl$_3$): *δ*=1.09 (d, *J*=7.4 Hz, 36H), 1.24 (spt, *J*=7.3 Hz, 6H), 2.19 (s, 6H), 5.58 (s, 2H), 6.77 (s, 2H), 6.82–6.90 (m, 6H), 6.97 (s, 2H), 7.13–7.19 ppm (m, 4H); ^{13}C NMR and DEPT (125 MHz, CDCl$_3$): *δ*=12.91 (6CH), 16.26 (2CH$_3$), 18.02 (12CH$_3$), 106.84 (2CH), 118.23 (4CH), 120.44 (2C), 120.84 (2CH), 121.27 (2C),129.12 (4CH), 133.78 (2CH), 139.75 (2C), 143.28 (2C), 154.12 ppm (2C); MS (ESI, +10 V): *m/z*=710.0 [M+H]$^+$.

{[5,5'-Dimethyl-4,4'-bis[(triisopropylsilyl)oxy]-(1,1'-biphenyl)-2,2'-diyl]bis(azanediyl)}bis(3,1-phenylene) bis(2,2-dimethylpropanoate) (**11b**)

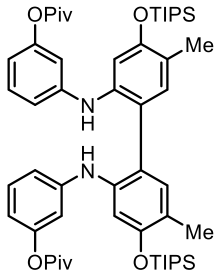

11b

General Procedure III: 32.3 mg (71 µmol) **10b**, additive: 2.0 mg (14 µmol, 20 mol%) $BF_3{\cdot}OEt_2$, 2.5 mg (1.1 µmol, 1.5 mol%) $(FeTPPF_{28})_2O$ (**5c**), reaction time: 20 h. Column chromatography: isohexane/EtOAc (30:1). Yellow oil; **11b**, yield: 75% (24.4 mg, 29 µmol). ^1H NMR (500 MHz, $CDCl_3$): *δ*=1.10 (d, *J*=7.3 Hz, 36H), 1.20–1.29 (m, 6H), 1.32 (s, 18H), 2.19 (s, 6H), 5.55 (s, 2H), 6.53 (dd, *J*=7.9, 1.7 Hz, 2H), 6.59 (t, *J*=2.1 Hz, 2H), 6.67 (dd, *J*=8.1, 1.7 Hz, 2H), 6.79 (s, 2H), 6.96 (s, 2H), 7.13 ppm (t, *J*=8.1 Hz, 2H); ^{13}C NMR and DEPT (125 MHz, $CDCl_3$): *δ*=13.07 (6CH), 16.48 ($2CH_3$), 18.22 ($12CH_3$), 27.30 ($6CH_3$), 39.13 (2C), 107.60 (2CH), 110.28 (2CH), 113.68 (2CH), 115.07 (2CH), 120.92 (2C), 122.20 (2C), 129.90 (2CH), 133.94 (2CH), 139.01 (2C), 144.73 (2C), 152.21 (2C), 154.36 (2C), 176.92 ppm (2C=O); MS (ESI, +10 V): *m/z*=909.9 $[M+H]^+$.

{[5,5'-Dimethyl-4,4'-bis[(triisopropylsilyl)oxy]-(1,1'-biphenyl)-2,2'-diyl]bis(azanediyl)}bis(4,1-phenylene) bis(2,2-dimethylpropanoate) (**11c**)

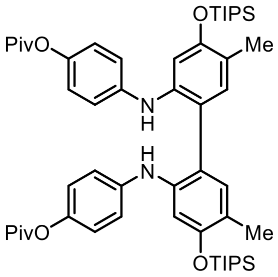

11c

General Procedure III: 32.0 mg (70 µmol) **10c**, additive: 2.1 mg(15 µmol, 21 mol%) $BF_3{\cdot}OEt_2$, 2.7 mg (1.1 µmol, 1.6 mol%) $(FeTPPF_{28})_2O$ (**5c**), reaction time: 22 h. Column chromatography: isohexane/EtOAc (30:1). Colorless foam; **11c**, yield: 78% (24.9 mg, 27 µmol). ^1H NMR (500 MHz, $CDCl_3$): *δ*=1.09 (d, *J*=7.3 Hz, 36H), 1.18–1.29 (m, 6H), 1.34 (s, 18H), 2.19 (s, 6H), 6.76 (s, 2H), 6.87 (s, 8H), 6.97 ppm (s, 2H); ^{13}C NMR and DEPT (125 MHz, $CDCl_3$): *δ*=13.07 (6CH), 16.45 ($2CH_3$), 18.18 ($12CH_3$), 27.31 ($6CH_3$), 39.13 (2C), 107.50 (2CH), 119.41 (4CH), 120.57 (2C),122.23 (4CH), 133.96 (2CH), 139.59 (2C), 140.59 (2C), 145.56 (2C), 154.50 (4C), 177.32 ppm (2C=O); MS (ESI, +10 V): *m/z*=909.9 $[M+H]^+$, (ESI, −50 V): *m/z*=907.4 $[M-H]^-$.

Table S1 Variation of Catalyst and Additive for the Oxidative C–C Coupling of Diarylamines[a]

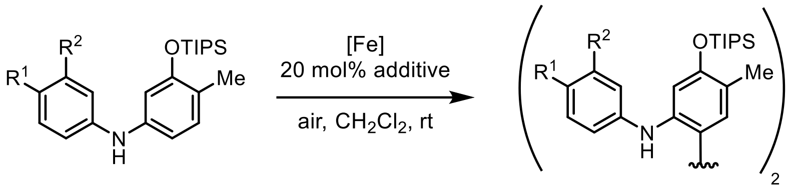

10a (R^1 = H, R^2 = H)
10b (R^1 = H, R^2 = OPiv)
10c (R^1 = OPiv, R^2 = H)

11a (R^1 = H, R^2 = H)
11b (R^1 = H, R^2 = OPiv)
11c (R^1 = OPiv, R^2 = H)

Entry	Diarylamine	[Fe] (mol%)	Additive (mol%)	time [h]	Yield **11** [%]
1[4]	**10a**	$FePcF_{16}$ (3.0)	MsOH (10)	0.2	57
2[b]	**10a**	$(FeTPPF_{28})_2O$ (1.5)	TfOH (20)	48	52
3[c]	**10a**	$(FeTPPF_{28})_2O$ (1.5)	$BF_3{\cdot}OEt_2$ (20)	24	70
4[4]	**10b**	$FePcF_{16}$ (3.0)	MsOH (10)	1.5	65
5[d]	**10b**	$(FeTPPF_{28})_2O$ (1.5)	TfOH (20)	48	55
6	**10b**	$(FeTPPF_{28})_2O$ (1.5)	$BF_3{\cdot}OEt_2$ (20)	20	76
7[4]	**10c**	$FePcF_{16}$ (3.0)	MsOH (10)	0.5	73
8[e]	**10c**	$(FeTPPF_{28})_2O$ (1.5)	TfOH (20)	48	63
9	**10c**	$(FeTPPF_{28})_2O$ (1.5)	$BF_3{\cdot}OEt_2$ (20)	22	78

[a] Reaction conditions: **10** (0.1 mmol), solvent (2 mL), additive, air, room temperature; all yields refer to isolated products. [b] Reisolated **10a**: 12%. [c] Reisolated **10a**: 8%. [d] Reisolated **10b**: 36%. [e] Reisolated **10c**: 23%. [4] Reference 4 (see page 29). Pc = phthalocyanine, TPP = tetraphenylporphyrin.

Asymmetric Oxidative C–C Coupling of Diarylamines

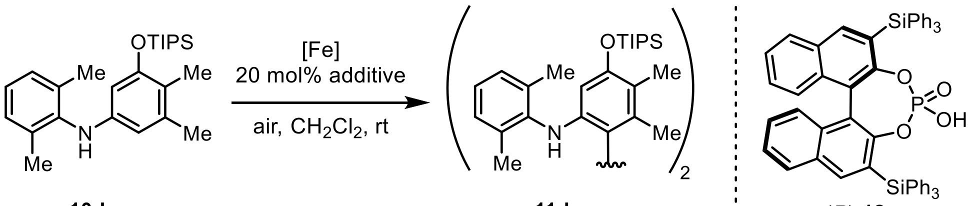

10d **11d** (*R*)-**12**

N^2,$N^{2'}$-Bis(2,6-dimethylphenyl)-5,5',6,6'-tetramethyl-4,4'-bis[(triisopropylsilyl)oxy]-(1,1'-biphenyl)-2,2'-diamine (**11d**)

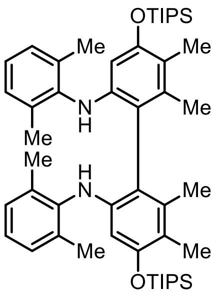

11d

General Procedure III (racemic synthesis): **10d** (39.6 mg, 100 µmol), $BF_3·OEt_2$ (3.0 mg, 21 µmol, 21 mol%), $(FeTPPF_{28})_2O$ (**5c**) (3.6 mg, 1.5 µmol, 1.5 mol%), reaction time: 24 h. Column chromatography: isohexane/dichloromethane 20:1. Colorless solid; **11d**, yield: 71% (28.3 mg, 36 µmol). ^{1}H NMR (600 MHz, $CDCl_3$): δ=0.94 (d, J=7.4 Hz, 18H), 0.95 (d, J=7.3 Hz, 18H), 1.01 (spt, J=7.3 Hz, 3H), 1.01 (spt, J=7.2 Hz, 3H), 1.99 (s, 6H), 2.14 (br s, 18H), 4.92 (s, 2H), 5.61 (s, 2H), 7.00–7.04 (m, 2H), 7.04–7.08 ppm (m, 4H); ^{13}C NMR and DEPT (151 MHz, $CDCl_3$): δ=12.50 ($2CH_3$), 12.88 (6CH), 17.33 ($2CH_3$), 18.07 ($12CH_3$), 18.54 ($4CH_3$), 99.85 (4CH), 115.34 (2C), 116.04 (2C), 125.92 (2CH), 128.46 (4CH), 138.22 (2C), 138.84 (2C), 143.37 (4C), 154.16 ppm (2C); MS (ESI, +10 V): m/z=793.7 $[M+H]^+$, (ESI, −50 V): m/z=791.2 $[M-H]^-$.

General Procedure III (asymmetric synthesis): **10d** (39.6 mg, 100 µmol), (R)-**12** (17.9 mg, 21 µmol, 21 mol%), $(FeTPPF_{28})_2O$ (**5c**) (3.6 mg, 1.5 µmol, 1.5 mol%), reaction time: 24 h. Column chromatography: isohexane/dichloromethane 20:1. White solid; **11d**, yield: 64% (25.1 mg, 32 µmol), 96% ee.

Chiral HPLC: For the determination of the enantiomeric excess by chiral HPLC, **11d** was converted into **11e**

11d (1 mg) was dissolved in THF (50 µL) under an argon atmosphere. A 1M solution of TBAF in THF (20 µL) was added and the mixture was stirred for 5 min at rt. Then, 1M aqueous HCl (0.5 mL) was added and the solution was stirred for another 5 min. The reaction mixture was extracted three times with Et_2O (0.2 mL) and the combined organic layers were dried in vacuo to afford the bisphenol **11e**, which was dissolved in iPrOH (1.0 mL).

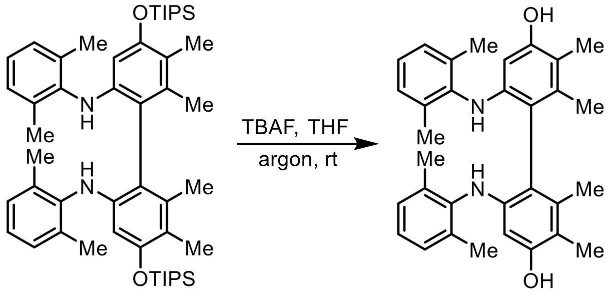

11d **11e**

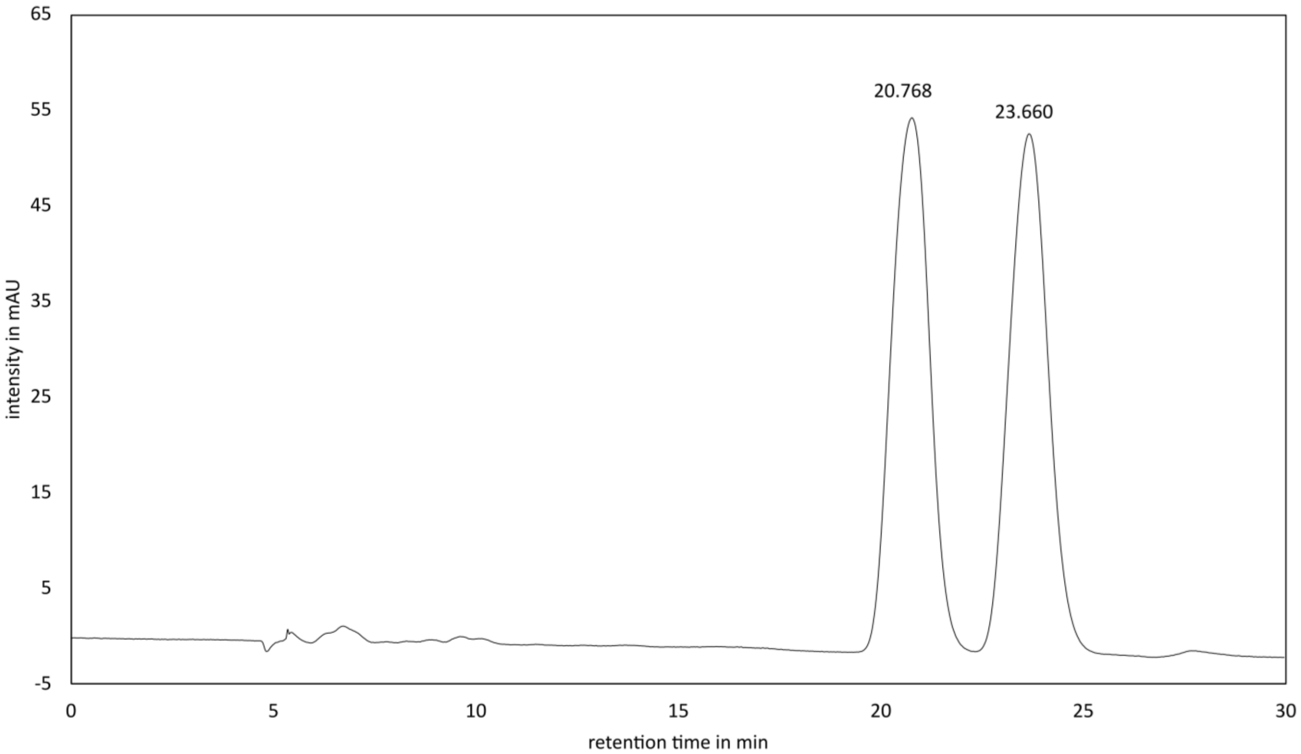

Fig. S2 Chiral HPLC of *rac*-**11e**, hexane/isopropanol, 95:5, 250 nm), t_1: 20.77 min (50.1%), t_2: 23.66 min (49.9%).

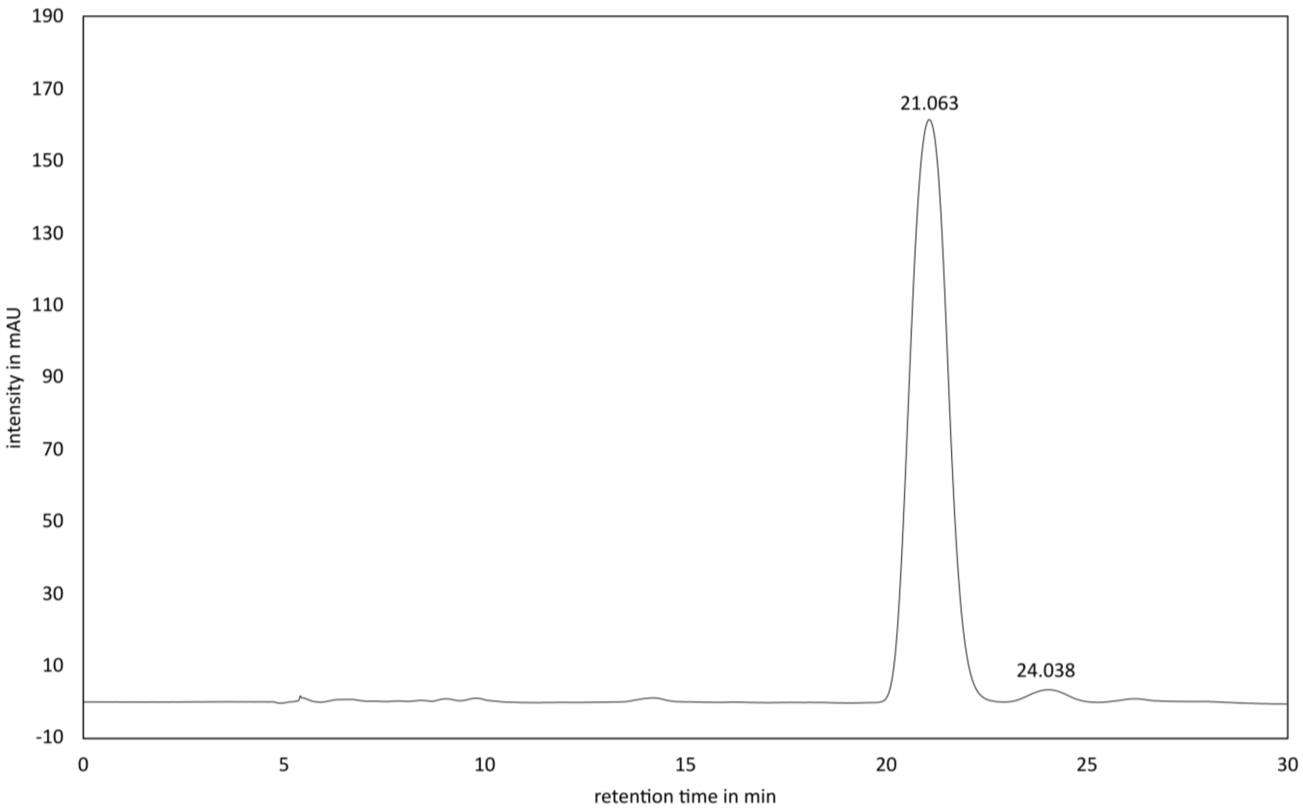

Fig. S3 Chiral HPLC of enantioenriched **11e**, hexane/isopropanol, 95:5, 250 nm), t_1: 21.06 min (98.0%), t_2: 24.03 min (2.0%).

Oxidative C–C Coupling for the Synthesis of 1,1'- and 4,4'-Bicarbazoles

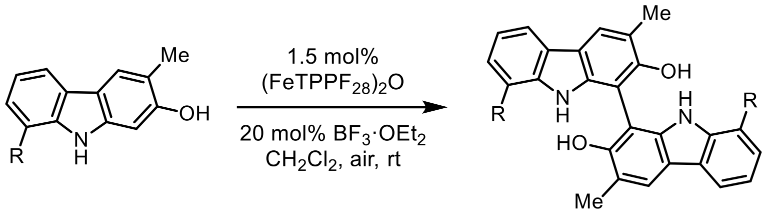

13a (R = H)
13b (R = OMe)

14a (R = H)
14b (R = OMe)

General Procedure IV (Synthesis of 1,1'-Bicarbazoles): Carbazole (1.0 equiv), µ-oxo iron catalyst $(FeTPPF_{28})_2O$ (**5c**) 1.5 mol%) and additive (20 mol%) were placed in a round-bottom flask equipped with a magnetic stirring bar and dissolved in dichloromethane (c ~ 50 mM). To minimize solvent loss by evaporation a splash head was placed on top of the flask. The reaction mixture was stirred vigorously at room temperature under ambient air. The reaction was monitored by TLC analysis. After completion of the reaction, triethylamine (10 equiv) was added. The reaction mixture was then filtered through a short silica pad and rinsed with dichloromethane. The solvent was removed in vacuo and the residue was adsorbed on silica gel. The crude product was purified by column chromatography on silica gel.

Bis-2-hydroxy-3-methylcarbazole (3,3'-Dimethyl-9*H*,9*H*'-[1,1'-bicarbazole]-2,2'-diol) (**14a**)

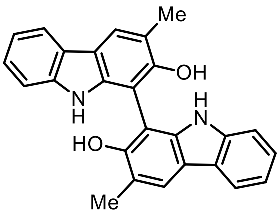

14a

General Procedure IV: **13a** (29.5 mg, 0.15 mmol), $BF_3·OEt_2$ (4.4 mg, 30 µmol, 20 mol%), $(FeTPPF_{28})_2O$ (**5c**) (5.4 mg, 2.3 µmol, 1.5 mol%), reaction time: 2 h. Column chromatography: isohexane/ dichloromethane/ethyl acetate (7:2:1). Pale yellow/gray solid; **14a**, yield: 84% (24.7 mg, 63 µmol); 7% of starting material **13a** were reisolated. **14a**: ^{1}H NMR (600 MHz, [D$_6$]acetone): δ=2.46 (d, *J*=0.7 Hz, 6H), 7.11 (ddd, *J*=8.0, 7.0, 1.0 Hz, 2H), 7.20 (ddd, *J*=8.1, 7.0, 1.1 Hz, 2H), 7.28 (dd, *J*=8.0, 1.0 Hz, 2H), 7.40 (s, 2H), 7.90 (s, 2H), 8.01 (d, *J*=7.7 Hz, 2H), 9.62 ppm (br s, 2H); ^{13}C NMR and DEPT (151 MHz, [D$_6$]acetone): δ=17.39 (CH_3), 17.41 (CH_3), 102.52 (2C), 111.54 (CH), 111.59 (CH), 116.97 (2C), 118.24 (2C), 119.36 (CH), 119.37 (CH) 119.60 (2CH), 122.47 (2CH), 124.50 (2CH), 124.70 (CH), 124.74 (CH), 140.13 (C), 140.25 (C), 140.89 (C), 141.03 (C), 153.36 (C), 153.47 ppm (C); MS (ESI, +10 V): m/z=393.1 [M+H]$^+$, 785.4 [2M+H]$^+$, (ESI, −50 V): *m/z*=391.1 [M−H]$^-$, (ESI, −50 V): *m/z*=805.2 [2(M−H)+Na]$^-$.

Biscarbalexine B (8,8'-Dimethoxy-3,3'-dimethyl-9*H*,9*H*'-[1,1'-bicarbazole]-2,2'-diol) (**14b**)

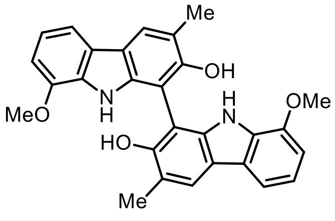

14b

General Procedure IV: **13b** (34.1 mg, 150 µmol), $BF_3{\cdot}OEt_2$ (4.3 mg, 30 µmol, 20 mol%), $(FeTPPF_{28})_2O$ (**5c**) (5.3 mg, 2.3 µmol, 1.5 mol%), reaction time: 5 h. Column chromatography: isohexane/ dichloromethane/ethyl acetate (7:2:1). Gray solid; **14b**, yield: 45% (15.5 mg, 34 µmol). ^{1}H NMR (500 MHz, [D$_6$]DMSO): δ=2.43 (s, 6H), 3.81 (s, 6H), 6.82 (d, *J*=7.8 Hz, 2H), 7.03 (t, *J*=7.8 Hz, 2H), 7.60 (d, *J*=7.8 Hz, 2H), 7.86 (s, 2H), 7.95 (s, 2H), 9.31 ppm (s, 2H); ^{13}C NMR and DEPT (125 MHz, [D$_6$]DMSO): δ=17.64 (2CH$_3$), 55.08 (2CH$_3$), 104.44 (2C), 104.73 (2CH), 111.63 (2CH), 115.96 (2C), 117.57 (2C), 118.94 (2CH), 120.83 (2CH), 124.78 (2C), 129.14 (2C), 139.03 (2C), 145.34 (2C), 152.22 ppm (2C); MS (ESI, +10 V): *m/z*=453.2 [M+H]$^+$; MS (ESI, −50 V): *m/z*=450.9 [M−H]$^-$.

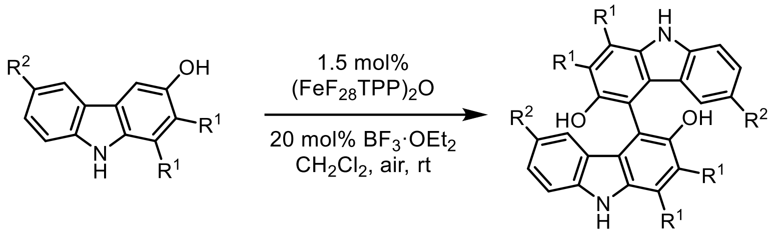

13c (R^1 = Me, R^2 = H)
13d (R^1 = H, R^2 = Me)

14c (R^1 = Me, R^2 = H)
14d (R^1 = H, R^2 = Me)

General Procedure V (Synthesis of 4,4'-Bicarbazoles): Carbazole (1.0 equiv), µ-oxo iron catalyst $(FeTPPF_{28})_2O$ (**5c**) (1.5 mol%) and additive (20 mol%) were placed in a round-bottom flask equipped with a magnetic stirring bar and suspended in dichloromethane (c ~ 12.5 mM). To minimize solvent loss by evaporation a splash head was placed on top of the flask and the reaction mixture was stirred vigorously at room temperature under ambient air. After completion of the reaction, the mixture was worked up as described below. The 4,4'-bicarbazoles are very sensitive to oxidation when heated, especially in the presence of traces of iron catalyst. Thus, high temperatures and exposure to air should be minimized during workup.

Sorazolon E2 (1,1',2,2'-Tetramethyl-9*H*,9'*H*-[4,4'-bicarbazole]-3,3'-diol) (**14c**)

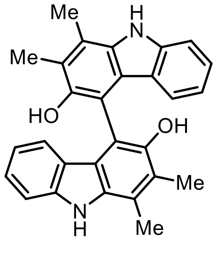

14c

General Procedure V: Sorazolon E (**13c**) (29.7 mg, 0.141 mmol), $BF_3{\cdot}OEt_2$ (4.3 mg, 30 µmol, 21 mol%), $(FeTPPF_{28})_2O$ (**5c**) (5.2 mg, 2.2 µmol, 1.6 mol%), CH_2Cl_2 (12 mL), reaction time: 24 h. Workup: The reaction mixture was filtered through a short silica gel pad and rinsed with dichloromethane. The first fraction (deep red, catalyst) was portioned off and the crude product was then collected. The solvent was degassed and removed under argon atmosphere and reduced pressure. The crude product was adsorbed on silica gel and purified by column chromatography: isohexane/ethyl acetate/MeOH 89:9:2. Brown solid; **14c**, yield: 74% (21.9 mg, 52 µmol). *(Remark: If the workup is not executed with the appropriate care, an oxidized quinone by-product can be observed as deep brown fraction eluting directly after the desired product. Reduction of this fraction with $NaBH_4$ (excess) in MeOH at rt (10 min affords the desired product **14c**.)* M.p. >300 °C (dec.); ^{1}H NMR (600 MHz, [D$_6$]acetone): δ=2.45 (s, 6H), 2.66 (s, 6H), 6.50 (s, 2OH), 6.53 (ddd, J=8.0, 7.1, 0.8 Hz, 2H), 6.64 (d, J=8.0 Hz, 2H), 7.06 (ddd, J=8.1, 7.1, 1.1 Hz, 2H), 7.34 (d, J=8.0 Hz, 2H), 10.05 ppm (s, 2NH); ^{13}C NMR and DEPT (151 MHz, [D$_6$]acetone): δ=13.07 ($2CH_3$), 14.30 ($2CH_3$), 111.14 (2CH), 111.66 (2C), 118.58 (2CH), 120.09 (2C), 120.27 (2C), 122.01 (2CH), 123.09 (2C), 124.62 (2C), 125.16 (2CH), 135.38 (2C), 141.27 (2C), 147.81 ppm (2C); IR (ATR): $\tilde{\nu}$=3772, 3526, 3494, 3443, 3375, 3051, 2920, 2850, 1704, 1612, 1583, 1500, 1452, 1389, 1345, 1308, 1257, 1216, 1166, 1147, 1108, 1084, 1060, 1015, 925, 889, 847, 801, 775, 731, 691, 664, 643 cm^{-1}; UV/Vis (MeOH): λ_{max}=216, 234, 265, 302, 341, 353 nm; fluorescence (MeOH): λ_{ex}=216 nm; λ_{em}=388 nm; HRMS (ESI): *m/z* calcd for $C_{28}H_{25}N_2O_2^+$ ($[M+H]^+$): 421.1911; found: 421.1910.

Crystallographic data for Sorazolon E2 (**14c**):

Crystallization of **14c** from dichloromethane/methanol afforded single crystals suitable for X-ray analysis.

$C_{28}H_{24}N_2O_2$ + CH_3OH, M = 452.53 gmol^{-1}, crystal size: 0.130 × 0.379 × 0.491 mm, triclinic, space group $P\bar{1}$, a = 8.7151(3), b = 9.8582(3), c = 14.9790(4) Å, V = 1135.83(6) Å^3, Z = 2, ρ_{calcd} = 1.323 g/cm^3, μ = 0.086 mm^{-1}, λ = 0.71073 Å, T = 150(2) K, θ range: 2.64–28.33°, reflections collected: 58937, independent: 5640 (R_{int} = 0.0936), 332 parameters. The structure was solved by direct methods and refined by full-matrix least-squares on F^2; final R indices [I>2σ(I)]: R_1 = 0.0573, wR_2 = 0.1551; maximal residual electron density: 0.570 eÅ^{-3}; CCDC 2209872 contains the supplementary crystallographic data for this paper. These data can be obtained free of charge from The Cambridge Crystallographic Data Centre.

Integerrine B (6,6'-Dimethyl-9*H*,9'*H*-[4,4'-bicarbazole]-3,3'-diol) (**14d**)

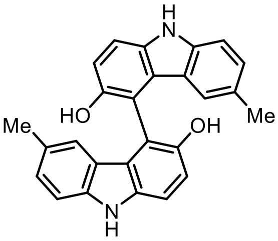

14d

General Procedure V: **13d** (29.4 mg, 0.149 mmol), BF_3·OEt_2 (4.5 mg, 32 µmol, 21 mol%), $(FeTPPF_{28})_2O$ (**5c**) (5.2 mg, 2.3 µmol, 1.5 mol%), reaction time: 23.5 h. Workup: The reaction mixture was filtered through a short pad of silica gel and rinsed with dichloromethane, the first fraction (deep red, catalyst) was portioned off and the crude product was then collected. The solvent was degassed and removed under argon atmosphere and reduced pressure. The crude product was adsorbed on silica gel and purified by column chromatography: isohexane/ethyl acetate/acetic acid 64:35:1. Gray solid, 35% yield **14d** (10.3 mg, 26 µmol). M.p. >300 °C (dec.); ^{1}H NMR (600 MHz, [D$_6$]acetone): δ=1.99 (s, 6H), 6.52 (s, 2H), 6.95 (dd, *J*=8.3, 1.1 Hz, 2H), 7.19 (d, *J*=8.6 Hz, 2H), 7.24 (d, *J*=8.2 Hz, 2H), 7.50 (d, *J*=8.6 Hz, 2H), 9.99 ppm (s, 2NH); ^{13}C NMR and DEPT (151 MHz, [D$_6$]acetone): δ=21.51 (2CH$_3$), 110.83 (2CH), 111.83 (2CH), 115.69 (2CH), 115.97 (2C), 122.44 (2CH), 123.35 (2C), 124.51 (2C), 127.05 (2CH), 127.05 (2C), 136.04 (2C), 140.06 (2C), 149.18 ppm (2C); IR (ATR): $\tilde{\nu}$=3403, 3028, 2949, 2918, 2853, 2198, 1733, 1627, 1580, 1492, 1437, 1374, 1339, 1288, 1256, 1149, 1062, 1031, 939, 876, 797, 638 cm^{-1}; UV/Vis (MeOH): λ_{max}=220, 236, 264, 302, 355 nm; fluorescence (MeOH): λ_{ex}=220 nm; λ_{em}=391 nm; HRMS (ESI): *m/z* calcd for $C_{26}H_{21}N_2O_2^+$ ([M+H]$^+$): 393.1598; found: 393.1599.

Table S2 Comparison of synthetic **14d** with the natural product (atom numbering according to isolation paper),[8] solvent: [D6]acetone

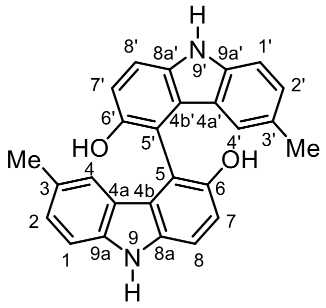

Integerrine B (**14d**)

position (H)	natural product[8]<br>400 MHz ^{1}H NMR<br>δ_H in ppm (*J* in Hz)	synthetic product<br>600 MHz ^{1}H NMR<br>δ_H in ppm (*J* in Hz)
1/1‘	7.24, d (8.2)	7.24, d (8.2)
2/2‘	6.95, dd (8.2, 1.6)	6.95, dd (8.3, 1.1)
4/4‘	6.52, d (1.6)	6.52, s
7/7‘	7.19, d (8.6)	7.19, d (8.6)
8/8‘	7.50, d (8.6)	7.50, d (8.6)
3-Me/3’-Me	1.99, s	1.99, s
9	10.0, br s	9.99, s

position (C)	natural product[8]<br>100 MHz ^{13}C NMR<br>δ_C in ppm	synthetic product<br>151 MHz ^{13}C NMR<br>δ_C in ppm
1/1‘	110.9, CH	110.8, CH
2/2‘	127.2, CH	127.1, CH
3/3‘	127.2, C	127.1 C
3-Me/3’-Me	21.6, CH_3	21.5, CH_3
4/4‘	122.5, CH	122.4, CH
4a/4a’	124.6, C	124.5, C
4b/4b’	123.4, C	123.4, C
5/5’	116.1, C	116.0, C
6/6’	149.3, C	149.2, C
7/7‘	115.8, CH	115.7, CH
8/8‘	111.9, CH	111.8, CH
8a/8a’	136.1, C	136.0, C
9a/9a’	140.1, C	140.1, C

Oxidative C–C Coupling for the Synthesis of 3,3'-Bicarbazoles

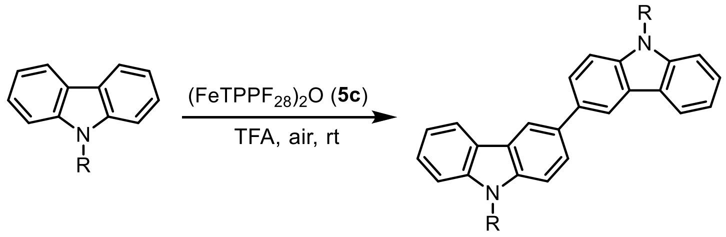

15a (R = Me) **16a** (R = Me)
15b (R = Bn) **16b** (R = Bn)
15c (R = Ph) **16c** (R = Ph)

General Procedure VI (Synthesis of 3,3'-Bicarbazoles): The *N*-substituted carbazole **15** (1.0 equiv) and iron catalyst $(FeTPPF_{28})_2O$ (**5c**) were placed in a round bottom flask equipped with a magnetic stir bar and suspended in trifluoroacetic acid (TFA). To minimize solvent loss by evaporation a splash head was placed on top of the flask and the reaction mixture was stirred vigorously. Small aliquots of the reaction mixture were neutralized with saturated aqueous K_2CO_3 and extracted with dichloromethane in a GC vial to monitor the reaction by TLC. After consumption of the starting material, the solution was transferred to a separatory funnel, diluted with water, and then neutralized with saturated K_2CO_3 solution. The aqueous layer was then extracted three times with dichloromethane. The combined organic layers were washed once with water and with brine and then dried over magnesium sulfate. The crude product obtained after evaporation of the solvent in vacuo was further purified by chromatography on silica gel.

9,9'-Dimethyl-9*H*,9'*H*-3,3'-bicarbazole (**16a**)

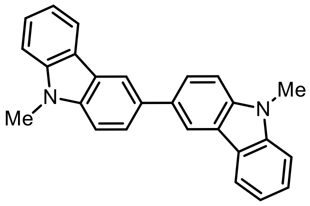

16a

General Procedure VI: 9-Methyl-9*H*-carbazole (**15a**) (18.4 mg, 102 µmol), $(FeTPPF_{28})_2O$ (**5c**) (3.5 mg, 1.5 µmol, 1.5 mol%), TFA (2.0 mL), reaction time: 1.5 h. The crude product was adsorbed on silica gel and purified by column chromatography: isohexane/ethyl acetate 9:1. Colorless solid, 82% yield **16a** (14.8 mg, 41 µmol). ^1H NMR (600 MHz, $[D_6]DMSO$): δ=3.93 (s, 6H), 7.24 (t, *J*=7.4 Hz, 2H), 7.46−7.52 (m, 2H), 7.61 (d, *J*=8.2 Hz, 2H), 7.70 (d, *J*=8.5 Hz, 2H), 7.92 (dd, *J*=8.5, 1.8 Hz, 2H), 8.29 (d, *J*=7.6 Hz, 2H), 8.58 (d, *J*=1.62 Hz, 2H); ^{13}C NMR and DEPT (151 MHz, $[D_6]DMSO$): δ=29.09 ($2CH_3$), 109.20 (2CH), 109.22 (2CH), 118.20 (2CH), 118.72 (2CH), 120.47 (2CH), 122.26 (2C), 122.67 (2C), 125.01 (2CH), 125.77 (2CH), 132.25 (2C), 139.71 (2C), 141.09 (2C); MS (ESI, +10 V): *m/z*=361.2 $[M+H]^+$. The physical and spectroscopic data are in agreement with those reported in the literature.[9]

9,9'-Dibenzyl-9*H*,9'*H*-3,3'-bicarbazole (**16b**)

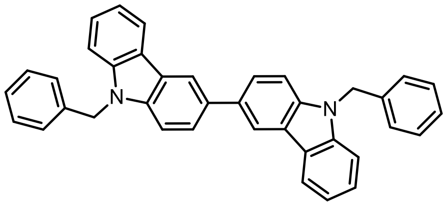

16b

General Procedure VI: 9-Benzyl-9*H*-carbazole (**15b**) (25.9 mg, 101 µmol), $(FeTPPF_{28})_2O$ (**5c**) 3.6 mg (1.5 µmol, 1.5 mol%), TFA (3.0 mL), reaction time: 18 h. The crude product was adsorbed on silica gel and purified by column chromatography: isohexane/ethyl acetate 9:1. Colorless solid; **16b**, yield: 71% (18.4 mg, 36 µmol). ^1H NMR (600 MHz, [D6]DMSO): δ=5.71 (s, 4H), 7.20−7.22 (m, 4H), 7.24 (t, *J*=7.9 Hz, 4H), 7.29 (t, *J*=7.4 Hz, 4H), 7.43−7.48 (m, 2H), 7.65 (d, *J*=8.2 Hz, 2H), 7.73 (d, *J*=8.5 Hz, 2H), 7.86 (dd, *J*=8.5, 1.8 Hz, 2H), 8.30 (d, *J*=7.7 Hz, 2H), 8.59 (d, *J*=1.6 Hz, 2H); ^{13}C NMR and DEPT (151 MHz, [D6]DMSO): δ=45.67 ($2CH_2$), 109.64 (2CH), 109.89 (2CH), 118.48 (2CH), 119.05 (2CH), 120.60 (2CH), 122.51 (2C), 122.94 (2C), 125.25 (2CH), 125.93 (2CH), 126.76 (4CH), 127.25 (2CH), 128.59 (4CH), 132.63 (2C), 137.88 (2C), 139.26 (2C), 140.63 (2C); MS (ESI, +25 V): *m/z*=513.4 $[M+H]^+$, 1025.7 $[2M+H]^+$. The physical and spectroscopic data are in agreement with those reported in literature.[10]

9,9'-Diphenyl-9*H*,9'*H*-3,3'-bicarbazole (**16c**)

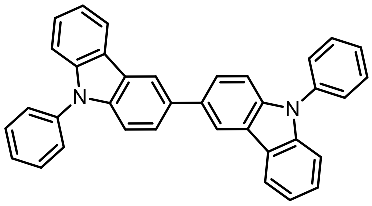

16c

General Procedure VI: 9-Phenyl-9*H*-carbazole (**15c**) (24.3 mg, 100 µmol), $(FeTPPF_{28})_2O$ (**5c**) (6.2 mg, 2.6 µmol, 2.6 mol%), TFA (4.0 mL), reaction time: 4.5 h. The crude product was adsorbed on silica gel and purified by column chromatography: pentane/ethyl acetate 30:1. Colorless solid; **16c**, yield: 82% (19.7 mg, 41 µmol). ^1H NMR ([D6]DMSO) δ=7.34 (ddd, *J*=7.8, 7.0, 0.9 Hz, 2H), 7.42 (d, *J*=8.2 Hz, 2H), 7.47 (ddd, *J*=8.2, 7.0, 1.2 Hz, 2H), 7.50 (d, *J*=8.5 Hz, 2H), 7.56 (tt, *J*=10.9, 1.4 Hz, 2H), 7.67−7.70 (m, 4H), 7.70−7.75 (m, 4H), 7.89 (dd, *J*=8.5, 1.9 Hz, 2H), 8.40 (td, *J*=7.8, 0.9 Hz, 2H), 8.70 (d, *J*=1.6 Hz, 2H). ^{13}C NMR and DEPT (151 MHz, [D6]DMSO): δ=109.71 (2CH), 110.01 (2CH), 118.55 (2CH), 120.12 (2CH), 120.83 (2CH), 122.99 (2C), 123.48 (2C), 125.54 (2CH), 126.42 (2CH), 126.65 (4CH), 127.68 (2CH), 130.24 (4CH), 133.28 (2C), 136.91 (2C), 139.29 (2C), 140.60 (2C); MS (ESI, +10 V): *m/z*=485.3 $[M+H]^+$. The physical and spectroscopic data are in agreement with those reported in literature.[10]

Wacker-type Oxidation of Olefins

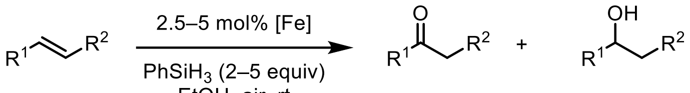

17a–g **18a–g** **19a–g**

General Procedure VII: Olefin **17a–h** (1.0 equiv), FeTPPF$_{28}$Cl (**4c**) (5.0 mol%) or (FeTPPF$_{28}$)$_2$O (**5c**) (2.5 mol%), and $PhSiH_3$ were given in a round-bottom flask equipped with a magnetic stirring bar and dissolved in ethanol (c ~ 50 mM). To minimize solvent loss by evaporation, a splash head was placed on top of the flask and the reaction mixture was stirred vigorously at room temperature under ambient air. After completion of the reaction, the mixture was filtered through a short pad of silica, and rinsed with ethyl acetate. The solvent was removed in vacuo and the residue was adsorbed on silica gel. The crude product was purified by column chromatography on silica gel. The ketones **18a**, **18d**, and **18f–h** are less polar than the corresponding alcohols **19a**, **19d**, and **19f–h**; only the ketone **18e** is more polar than the corresponding alcohol **19e**.

1-(Naphthalen-2-yl)ethanone (**18a**) and 1-(Naphthalen-2-yl)ethanol (**19a**)

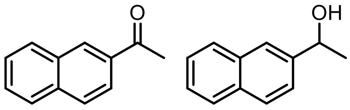

18a **19a**

General Procedure VII (under ambient air): 2-Vinylnaphthalene (**17a**) (31.2 mg, 202 µmol), $PhSiH_3$ (43.8 mg, 0.40 mmol, 2.0 equiv), (FeTPPF$_{28}$)$_2$O (**5c**) (11.8 mg, 5 µmol, 2.5 mol%), reaction time: 40 h. Column chromatography: isohexane/ethyl acetate (7:1, twice). **18a**: Colorless solid; yield: 87% (30.1 mg, 176 µmol). ^{1}H NMR (500 MHz, $CDCl_3$): δ=2.74 (s, 3H), 7.56 (ddd, J=8.1, 6.9, 1.2 Hz, 1H), 7.61 (ddd, J=8.1, 6.9, 1.3 Hz, 1H), 7.89 (t, J=8.3 Hz, 2H), 7.97 (d, J=8.1 Hz, 1H), 8.04 (dd, J=8.6, 1.8 Hz, 1H), 8.47 ppm (s, 1H); ^{13}C NMR and DEPT (125 MHz, $CDCl_3$): δ=26.70 (CH_3), 123.89 (CH), 126.77 (CH), 127.78 (CH), 128.41 (CH), 128.46 (CH), 129.54 (CH), 130.19 (CH), 132.50 (C), 134.48 (C), 135.58 (C), 198.12 ppm (C=O). MS (EI): *m/z*= 170 (34, M^+), 155 (70), 128 (12), 127 (100), 126 (33), 43 (31). **19a**: Traces detected by GC-MS, not isolated. MS (EI): *m/z*=172 (31, M^+), 157 (30), 129 (100), 128 (82), 127 (47), 126 (18), 43 (14).

General Procedure VII (under 1 atm of O_2): 2-Vinylnaphthalene (**17a**) (30.4 mg, 197 µmol), $PhSiH_3$ (44.5 mg, 0.41 mmol, 2.0 equiv), FeTPPF$_{28}$Cl (**4c**) (12.1 mg, 10 µmol, 5.0 mol%), reaction time: 24 h. Protocol according to the general procedure VII but with an oxygen balloon placed on top of the round-bottom flask. **18a**: Colorless solid; yield: 76% (25.6 mg, 150 µmol). **19a**: reddish solid; yield: 9% (3.0 mg, 17 µmol). ^{1}H NMR (600 MHz, $CDCl_3$): δ=1.59 (d, J=6.5 Hz, 3H), 5.08 (q, J=6.5 Hz, 1H), 7.48 (ddt, J=12.6, 7.2, 1.7 Hz, 2H), 7.51 (dd, J=8.5, 1.6 Hz, 1H), 7.80−7.86 ppm (m, 4H); ^{13}C NMR and DEPT (151 MHz, $CDCl_3$): δ=25.39 (CH_3), 70.81 (CH), 124.06 (2CH), 126.05 (CH), 126.40 (CH), 127.92 (CH), 128.18 (CH), 128.57 (CH), 133.17 (C), 133.57 (C), 143.42 (C); MS (EI): *m/z*=172 (31, M^+), 157 (30), 129 (100), 128 (82), 127 (47), 126 (18), 43 (14).

4-Acetylbenzonitrile (**18b**)

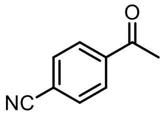

18b

General Procedure VII: 4-Vinylbenzonitrile (**17b**) (24.7 mg, 192 µmol), $PhSiH_3$ (44.1 mg, 2.0 equiv), $(FeTPPF_{28})_2O$ (**5c**) (11.9 mg, 5 µmol, 2.5 mol%), reaction time: 24 h. Column chromatography: isohexane/ethyl acetate 10:1. **18b**: Yellow solid; yield: 90% (25.7 mg, 177 µmol). ^1H NMR (600 MHz, $CDCl_3$): δ=2.65 (s, 3H), 7.78 (dt, *J*=8.4, 1.7 Hz, 2H), 8.04 ppm (dt, *J*=8.5, 1.6 Hz, 2H). ^{13}C NMR and DEPT (125 MHz, $CDCl_3$): δ=26.88 (CH_3), 116.61 (C), 118.06 (C), 128.64 (2CH), 132.67 (2CH), 140.10 (C), 196.62 ppm (C=O). MS (EI): *m/z* (%)=145 (18, M+), 130 (100), 116 (2), 102 (61), 89 (2),75 (23), 50 (15), 43 (20), 39 (3).

1-(4-Bromophenyl)ethanone (**18c**)

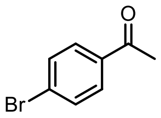

18c

General Procedure VII: 4-Bromostyrene (**17c**) (36.9 mg, 201 µmol), $PhSiH_3$ (3×21.6 mg, 3×1.0 equiv), $(FeTPPF_{28})_2O$ (**5c**) (11.9 mg, 5 µmol, 2.5 mol%), reaction time: 76 h. Column chromatography: isohexane/ethyl acetate 10:1. **18c**: Colorless solid; yield: 89% (33.9 mg, 170 µmol). ^1H NMR (500 MHz, $CDCl_3$): δ=2.59 (s, 3H), 7.61 (dt, *J*=8.7, 1.9 Hz, 2H), 7.82 ppm (dt, *J*=8.8, 1.9 Hz, 2H). ^{13}C NMR and DEPT (125 MHz, $CDCl_3$): δ=25.79 (CH_3), 127.55 (C), 129.08 (2CH), 131.14 (2CH), 135.05 (C), 196.28 ppm (C=O). MS (EI): *m/z* (%)=200 (28), 198 (29, M^+), 185 (99), 183 (100), 156 (46), 155 (46), 76 (33), 75 (32), 74 (22), 50 (30), 43 (21).

1-(4-Methoxyphenyl)ethanone (**18d**) and 1-(4-Methoxyphenyl)ethanol (**19d**)

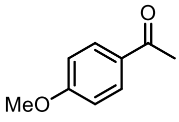

18d

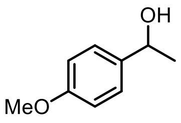

19d

General Procedure VII: 4-Methoxystyrene (**17d**) (26.9 mg, 200 µmol), $PhSiH_3$ (2×21.6 mg, 2×1.0 equiv), $(FeTPPF_{28})_2O$ (**5c**) 11.8 mg (5 µmol, 2.5 mol%), reaction time: 48 h. Column chromatography: gradient isohexane/ethyl acetate 10:1–6:1. **18d**: Colorless solid; yield: 85% (25.6 mg, 170 µmol). ^1H NMR (600 MHz, $CDCl_3$): *δ*=2.56 (s, 3H), 3.87 (s, 3H), 6.91–6.96 (m, 2H), 7.92–7.96 (m, 2H). ^{13}C NMR and DEPT (151 MHz, $CDCl_3$): *δ*=26.49 (CH_3), 55.61 (CH_3), 113.82 (2CH), 130.52 (C), 130.74 (2CH), 163.63 (C), 196.97 (C=O). MS (EI): *m/z* (%)=150 (29, M^+), 135 (100), 107 (14), 92 (21), 77 (29), 43 (9). **19d**: Reddish solid; yield: 7% (2.0 mg, 13 µmol). MS (EI): *m/z* (%)=152 (24, M^+), 137 (100), 135 (11), 109 (45), 94 (30), 92 (7), 91 (11), 77 (31), 43 (20).

2,2,8-Trimethyl-5-nitrochroman-4-one (**18e**) and 2,2,8-Trimethyl-5-nitrochroman-4-ol (**19e**)

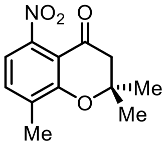

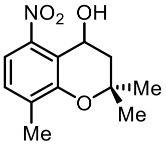

18e **19e**

General Procedure VII: 2,2,8-Trimethyl-5-nitro-2*H*-chromene (**17e**) (33.3 mg, 152 µmol), $PhSiH_3$ (32.5 mg, 2.0 equiv), $(FeTPPF_{28})_2O$ (**5c**) 8.8 mg (3.7 µmol, 2.5 mol%), reaction time: 62 h. Column chromatography: gradient isohexane/ethyl acetate 10:1–6:1. **18e**: Yellow solid; yield: 79% (28.1 mg, 119 µmol). ^1H NMR (500 MHz, $CDCl_3$): δ=1.50 (s, 6H), 2.26 (s, 3H), 2.78 (s, 2H), 6.92 (d, *J*=7.9 Hz, 1H), 7.36 (dd, *J*=7.9, 1.0 Hz, 1H); ^{13}C NMR and DEPT (125 MHz, $CDCl_3$): δ=16.35 (CH_3), 26.77 ($2CH_3$), 48.82 (CH_2), 80.48 (C), 111.53 (C), 114.42 (CH), 131.86 (C), 135.52 (CH), 147.19 (C) 158.31 (C), 189.12 ppm (C=O); MS (EI): *m/z* (%)=235 (50, M^+), 220 (52), 180 (82), 179 (53), 174 (18), 165 (12), 150 (25), 149 (93), 133 (14), 121 (100), 106 (12), 105 (33), 103 (10), 93 (12), 91 (15), 77 (52), 65 (34). **19e**: Yellow solid; yield: 11% (3.8 mg, 16 mmol). MS (EI): *m/z* (%)=237 (78, M^+), 219 (23), 204 (100), 182 (45), 181 (22), 174 (15), 163 (87), 158 (21), 151 (72), 134 (19), 133 (38), 118 (13), 106 (22), 105 (30), 91 (37), 79 (13), 78 (19), 77 (79), 65 (13).

2,2-Dimethyl-4-oxochromane-6-carbonitrile (**18f**) and 4-Hydroxy-2,2-dimethylchroman-6-carbonitrile (**19f**)

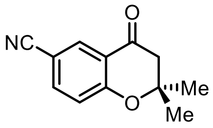

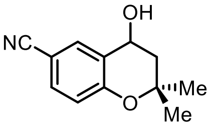

18f **19f**

General Procedure VII: 2,2-Dimethyl-2*H*-chromene-6-carbonitrile (**17f**) (37.2 mg, 201 µmol), $PhSiH_3$ (3×22 mg, 3×1.0 equiv), $(FeTPPF_{28})_2O$ (**5c**) 11.6 mg (5 µmol, 2.5 mol%), reaction time: 71 h. Column chromatography: gradient isohexane/ethyl acetate 6:1–4:1. **18f**: Colorless solid; yield: 93% (37.8 mg, 188 µmol). ^1H NMR (500 MHz, $CDCl_3$): δ=1.49 (s, 6H), 2.77 (s, 2H), 7.02 (d, *J*=8.7 Hz, 1H), 7.69 (dd, *J*=8.7, 2.2 Hz, 1H), 8.18 (d, *J*=2.2 Hz, 1H). ^{13}C NMR and DEPT (151 MHz, $CDCl_3$): δ=26.71 ($2CH_3$), 48.56 (CH_2), 80.99 (C), 104.70 (C), 118.33 (C), 120.05 (CH), 120.52 (C), 131.98 (CH), 138.58 (CH), 162.75 (CN), 190.52 (C=O). MS (EI): *m/z* (%)=201 (19, M^+), 186 (100), 146 (41), 145 (24), 117 (30). **19f**: Red solid; yield: 6% (2.5 mg, 12 µmol): MS (EI): *m/z* (%)=203 (62, M^+), 185 (8), 171 (20), 170 (100), 148 (98), 147 (75), 146 (90), 129 (18), 56 (24).

1-Tetralone (**18g**) and 1-Tetralol (**19g**)

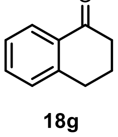

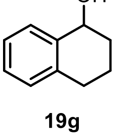

18g **19g**

General Procedure VII: 1,2-Dihydronaphthalene (**17g**) (26.1 mg, 200 µmol), $PhSiH_3$ (2×21.8 mg, 2×1.0 equiv), $(FeTPPF_{28})_2O$ (**5c**) (11.7 mg, 5 µmol, 2.5 mol%), reaction time: 48 h. Column chromatography: gradient isohexane/ethyl acetate 6:1–4:1. **18g**: Yellow oil; yield: 89% (26.5 mg, 178 µmol). ^1H NMR (500 MHz, $CDCl_3$): δ=2.15 (quin, *J*=6.4 Hz, 2H), 2.66 (t, *J*=6.5 Hz, 2H), 2.97 (t, *J*=6.1 Hz, 2H), 7.25 (d, *J*=8.5 Hz, 1H), 7.31 (ddd, *J*=7.9,

7.2, 0.7 Hz, 1H), 7.46 (td, J=7.5, 1.4 Hz, 1H), 8.04 (dd, J=7.9, 1.1 Hz, 1H). ^{13}C NMR and DEPT (125 MHz, $CDCl_3$): δ=23.44 (CH_2), 29.86 (CH_2), 39.33 (CH_2), 126.79 (CH), 127.33 (CH), 128.92 (CH), 132.78 (C), 133.54 (CH), 144.64 (C), 198.57 ppm (C=O). MS (EI): m/z (%)=146 (40, M^+), 118 (87), 117 (11), 115 (22), 91 (36), 90 (100), 89 (77), 87 (11), 77 (18), 76 (9), 75 (12), 74 (18), 65 (15), 64 (11), 63 (50). **19g**: Green oil; yield: 5% (1.5 mg, 10 µmol): MS (EI): m/z (%)=148 (61, M^+), 131 (13), 118(100), 90 (72), 77 (6), 63 (15), 50 (8), 39 (7).

Octadecan-2-one (**18h**) and Octadecan-2-ol (**19h**)

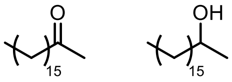

18h **19h**

General Procedure VII: Octadec-1-ene (**17h**) (46.2 mg, 183 µmol), $PhSiH_3$ (5×19.4 mg, 5×1.0 equiv), $(FeTPPF_{28})_2O$ (**5c**) (10.6 mg, 4.5 µmol, 2.5 mol%), reaction time: 120 h. Column chromatography: isohexane/ethyl acetate 9:1, the product fraction was again purified by chromatography with pentane/ethyl acetate 20:1. **18h**: Colorless solid; yield: 53% (25.9 mg, 96 µmol). ^1H NMR (600 MHz, $CDCl_3$): δ=0.88 (t, J=7.0 Hz, 3H), 1.26 (m, J=12.1 Hz, 26H), 1.56 (m, 2H), 2.13 (s, 3H), 2.41 (t, J=7.5 Hz, 2H). ^{13}C NMR and DEPT (151 MHz, $CDCl_3$): δ=14.27 (CH_3), 22.84 (CH_2), 24.04 (CH_2), 29.34 (CH_2), 29.51 (CH_2), 29.55 (CH_2), 29.62 (CH_2), 29.76 (CH_2), 29.80 (CH_2), 29.82 (CH_2), 29.84 ($4CH_2$), 29.99 (CH_3), 32.08 (CH_2), 43.99 (CH_2), 209.56 ppm (C); MS (EI): m/z (%)=268 (2, M^+), 253 (1), 210 (1), 95 (8), (84 (13), 70 (43), 68 (10), 57 (100), 54 (24), 42 (98), 40 (29). **19h**: Yellow oil, 11% (5.6 mg, 20 µmol). MS (EI): m/z (%)=252 (22, $[M–H_2O]^+$), 224 (2), 196 (2), 182 (2), 168 (4), 154 (4), 140 (5), 125 (18), 111 (42), 97 (82), 83 (77), 69 (62), 55 (100), 43 (48).

References

1. E. K. Woller, V. V Smirnov, S. G. DiMagno, *J. Org. Chem.* **1998**, *63*, 5706–5707.
2. E. K. Woller, S. G. DiMagno, *J. Org. Chem.* **1997**, *62*, 1588–1593.
3. M. K. Peters, F. Röhricht, C. Näther, R. Herges, *Org. Lett.* **2018**, *20*, 7879–7883.
4. R. F. Fritsche, G. Theumer, O. Kataeva, H.-J. Knölker, *Angew. Chem. Int. Ed.* **2017**, *56*, 549–553; *Angew. Chem.* ***2017***, *129*, 564–568.
5. H.-J. Knölker, M. Bauermeister, D. Bläser, R. Boese, J.-B. Pannek, *Angew. Chem. Int. Ed.* **1989**, *28*, 223–225; *Angew. Chem.* **1989**, *101*, 225–227.
6. H. J. Knölker, M. Bauermeister, D. Bläser, R. Boese, J.-B. Pannek, *Tetrahedron* **1993**, *49*, 841–862.
7. G. Shi, T. Yoon, S. Cha, S. Kim, M. Yousuf, N. Ahmed, D. Kim, H.-W. Kang, K. S. Kim, *ACS Sens.* **2018**, *3*, 1102–1108.
8. N.-K. Cao, Y.-M. Chen, S.-S. Zhu, K.-W. Zeng, M.-B. Zhao, J. Li, P.-F. Tu, Y. Jiang, *Phytochemistry* **2020**, *178*, 112463.
9. K. Matsumoto, Y. Toubaru, S. Tachikawa, A. Miki, K. Sakai, S. Koroki, T. Hirokane, M. Shindo, M. Yoshida, *J. Org. Chem.* **2020**, *85*, 15154–15166.
10. S. Mallick, S. Maddala, K. Kollimalayan, P. Venkatakrishnan, *J. Org. Chem.* **2019**, *84*, 73–93.

NMR Spectra

2a (Reaction Mixture) ^{1}H NMR (300 MHz) + ^{19}F NMR (282 MHz) (crude product after extraction with CH_2Cl_2)

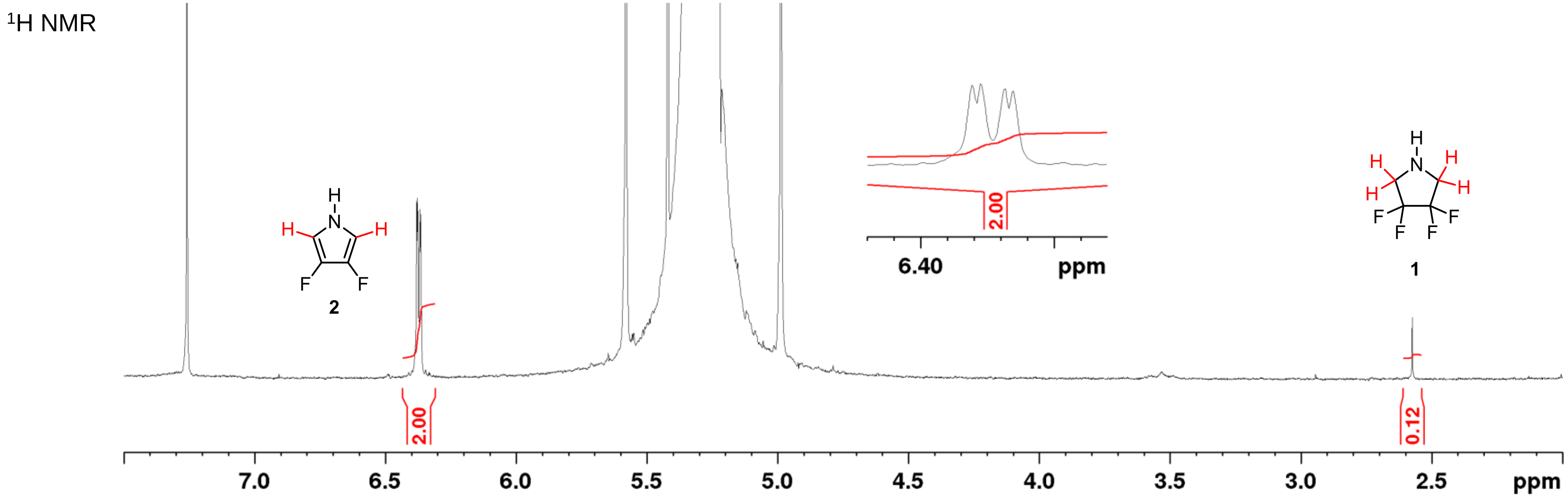

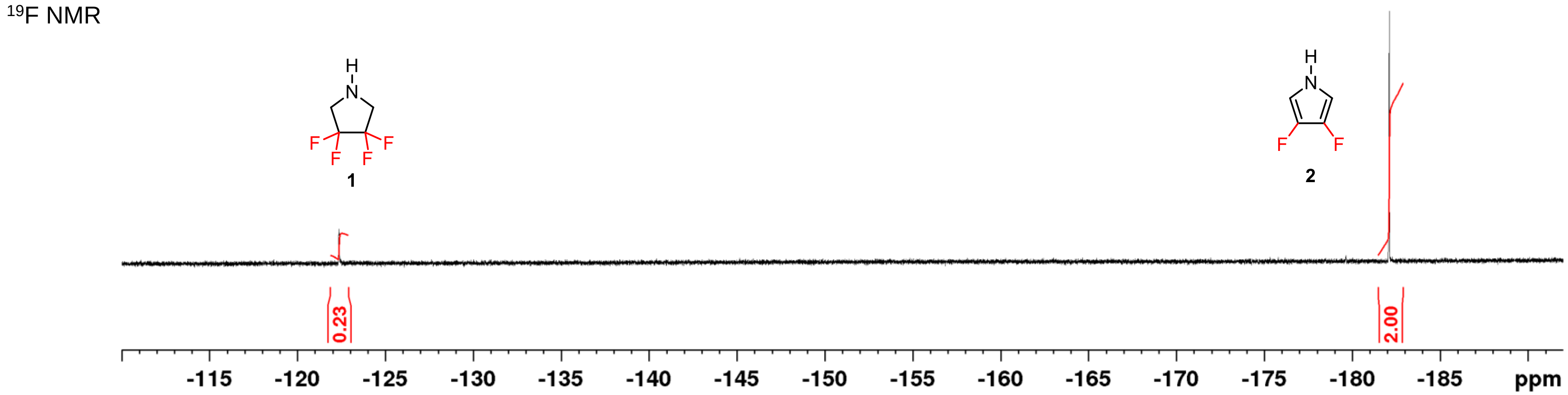

H$_2$TPPF$_8$ (**3a**), ^{1}H NMR, 600 MHz, CDCl$_3$

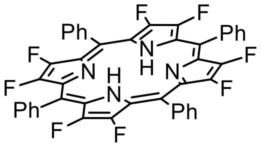

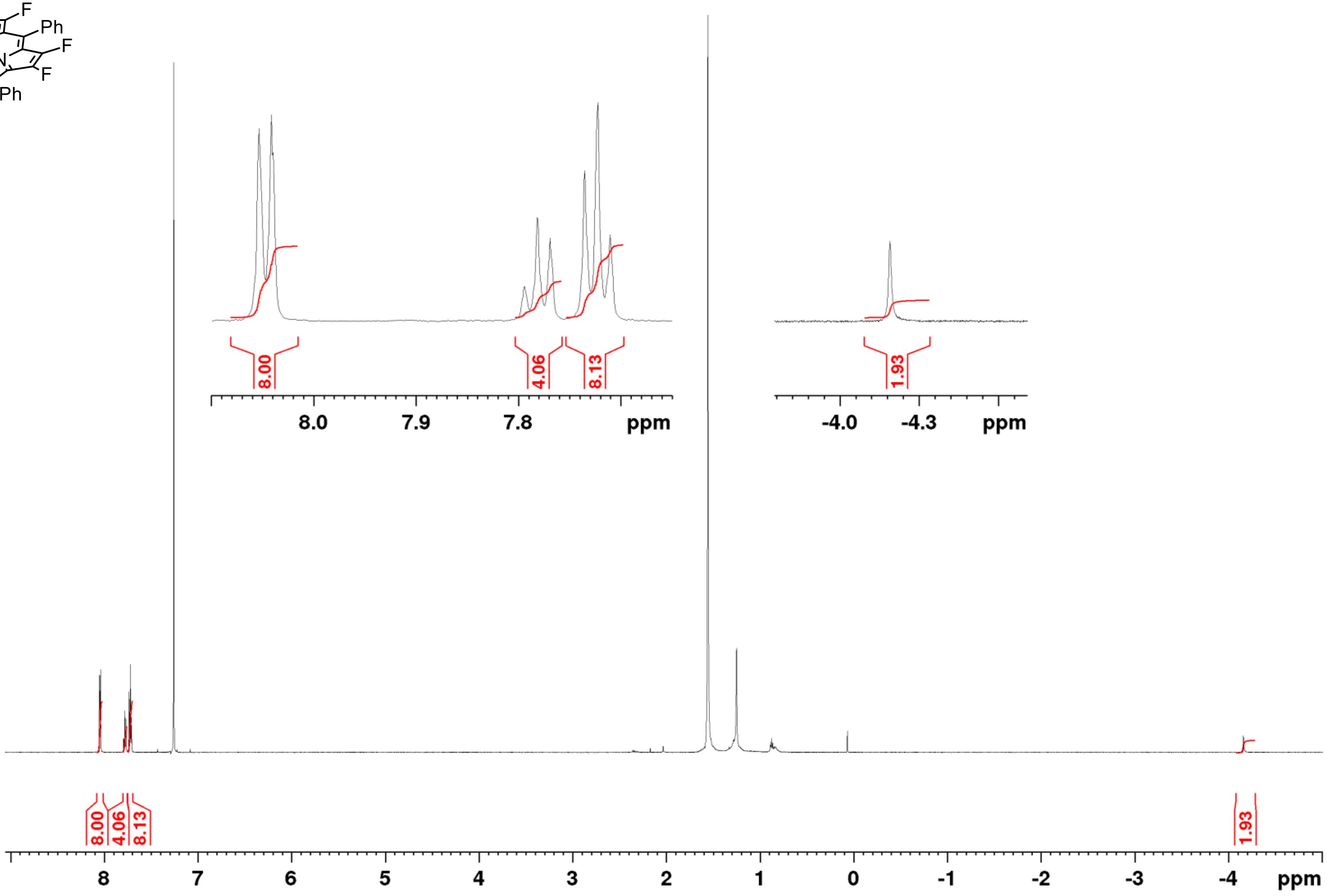

H$_2$TPPF$_8$ (**3a**), ^{19}F NMR, 282 MHz, CDCl$_3$

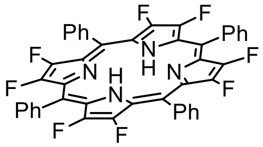

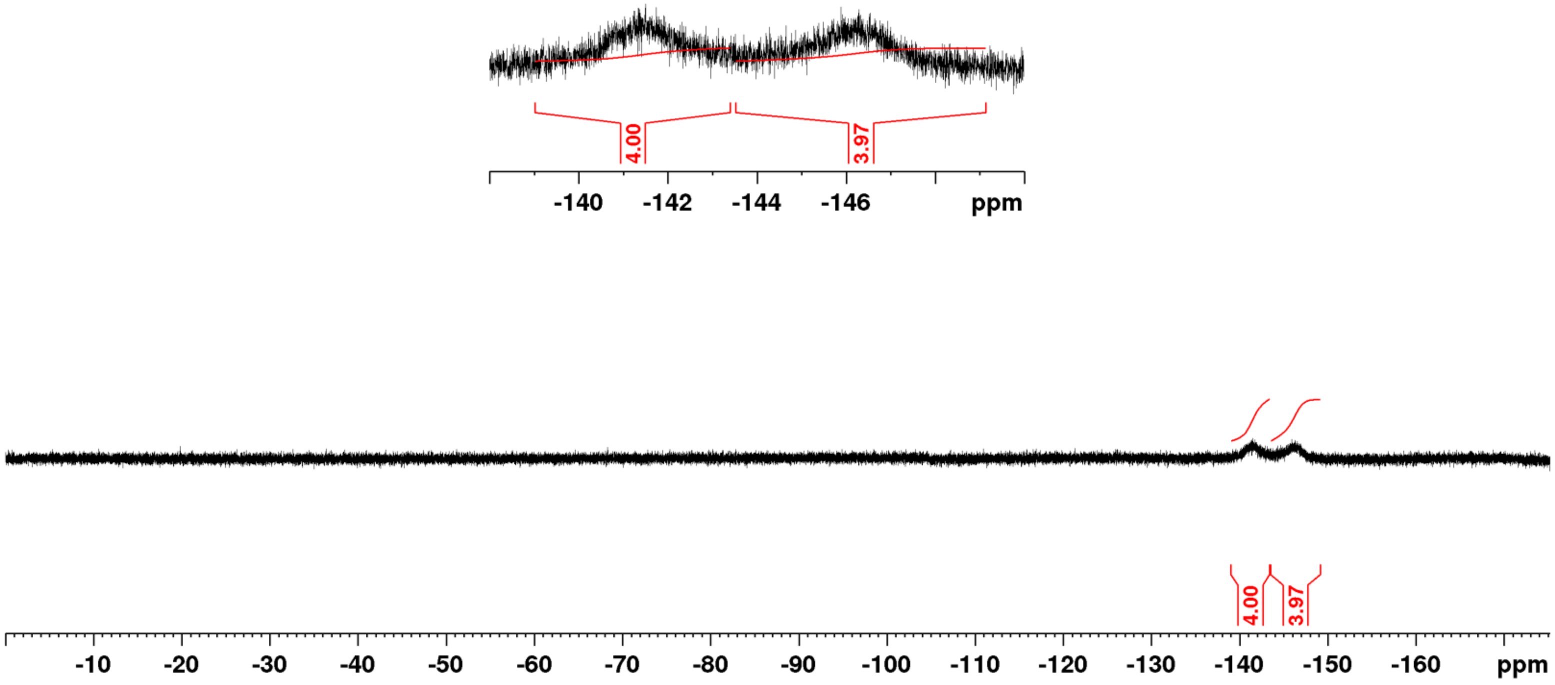

H_2TPPF_{20} (**3b**), ^1H NMR, 300 MHz, $CDCl_3$ (traces of CH_2Cl_2, 5.30 ppm)

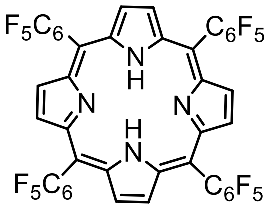

3b

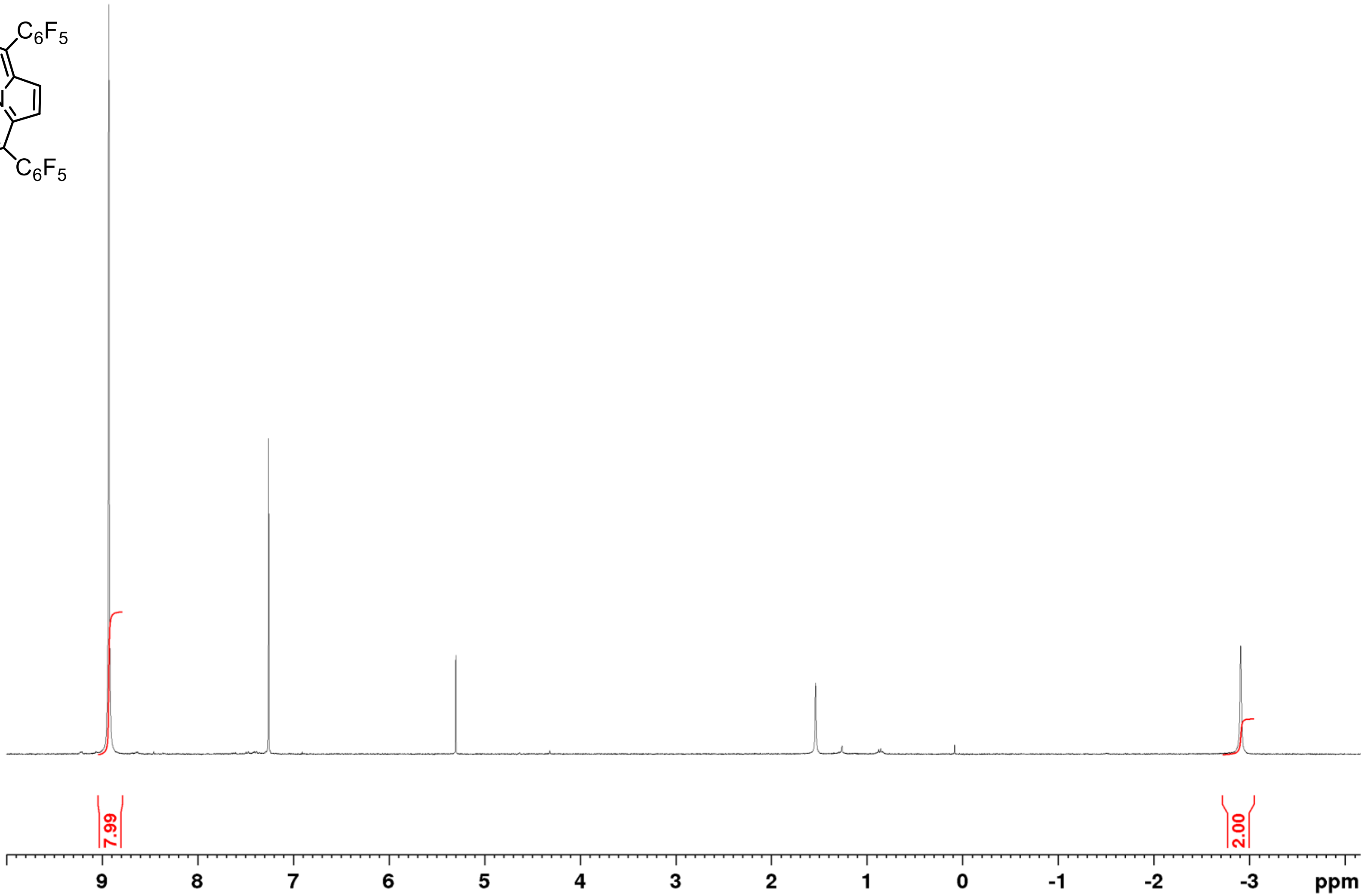

H_2TPPF_{20} (**3b**), ^{19}F NMR, 282.4 MHz, $CDCl_3$

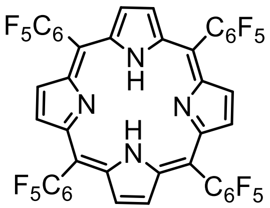

3b

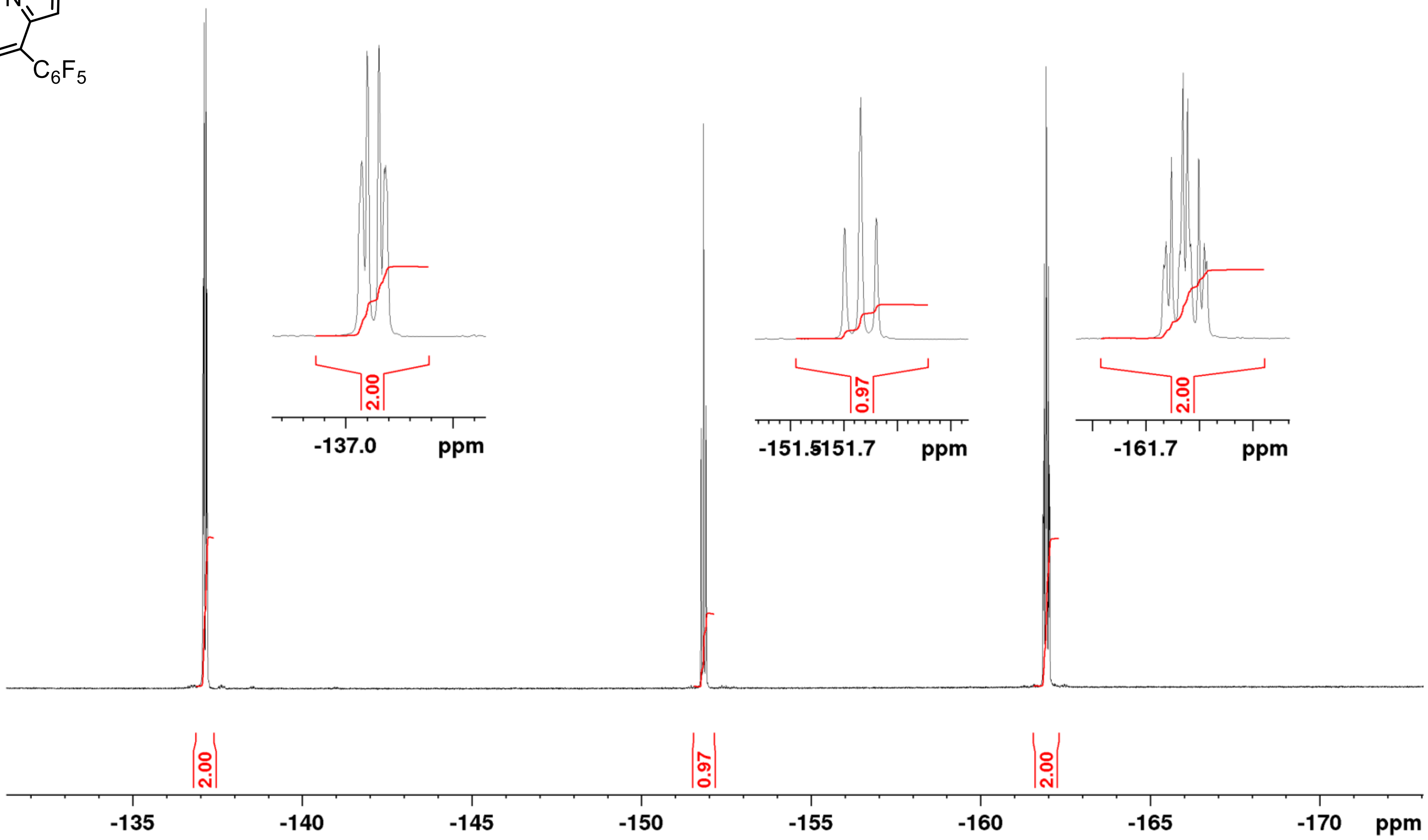

H_2TPPF_{28} (**3c**), ^1H NMR, 500 MHz, $CDCl_3$, traces of CH_2Cl_2 (5.30 ppm), water (1.56 ppm), silica gel (0.07 ppm)

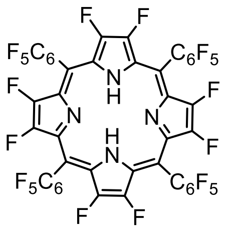

3c

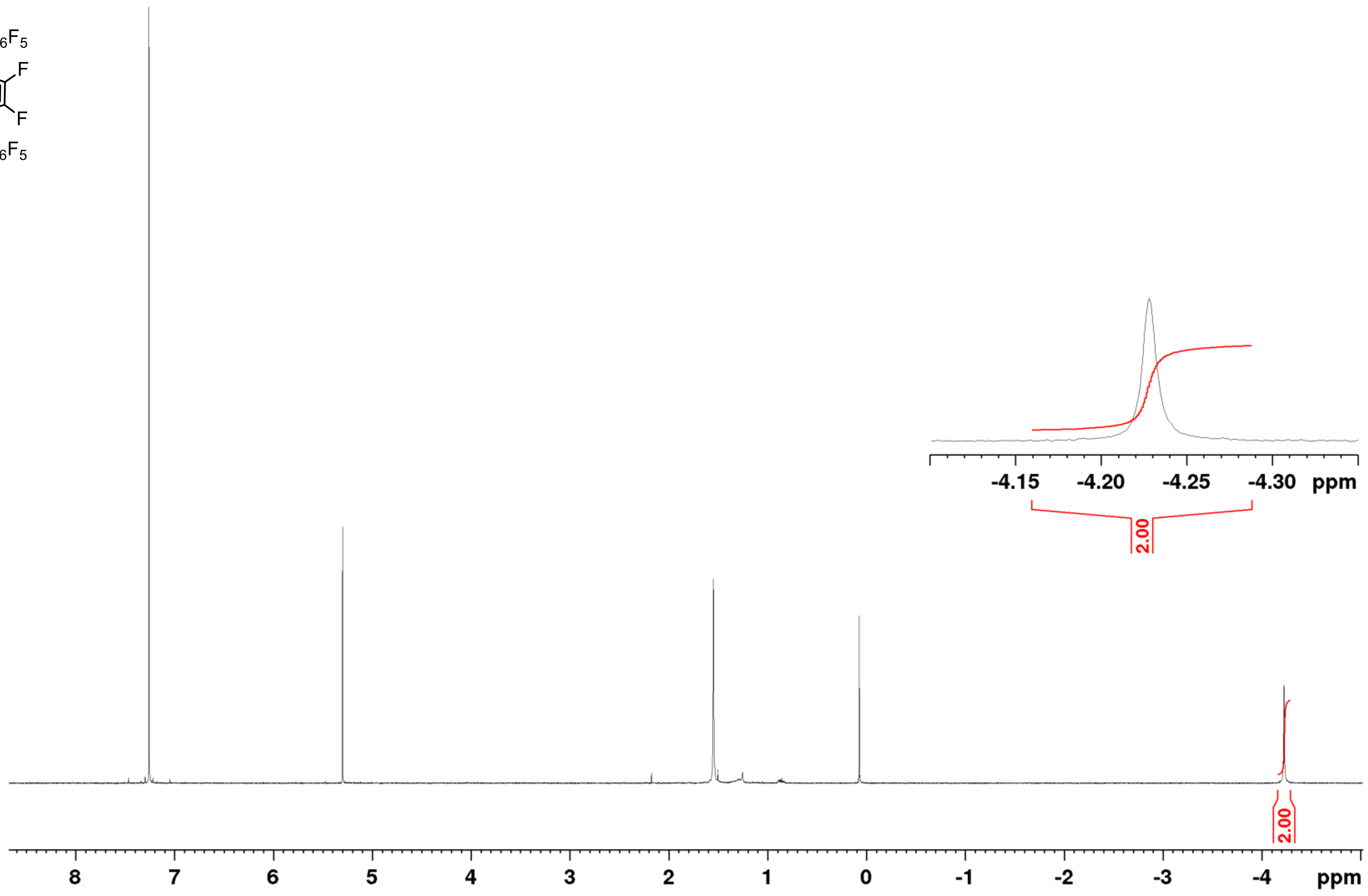

H_2TPPF_{28} (**3c**), ^{19}F NMR, 471 MHz, $CDCl_3$, 240 K

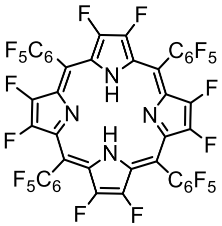

3c

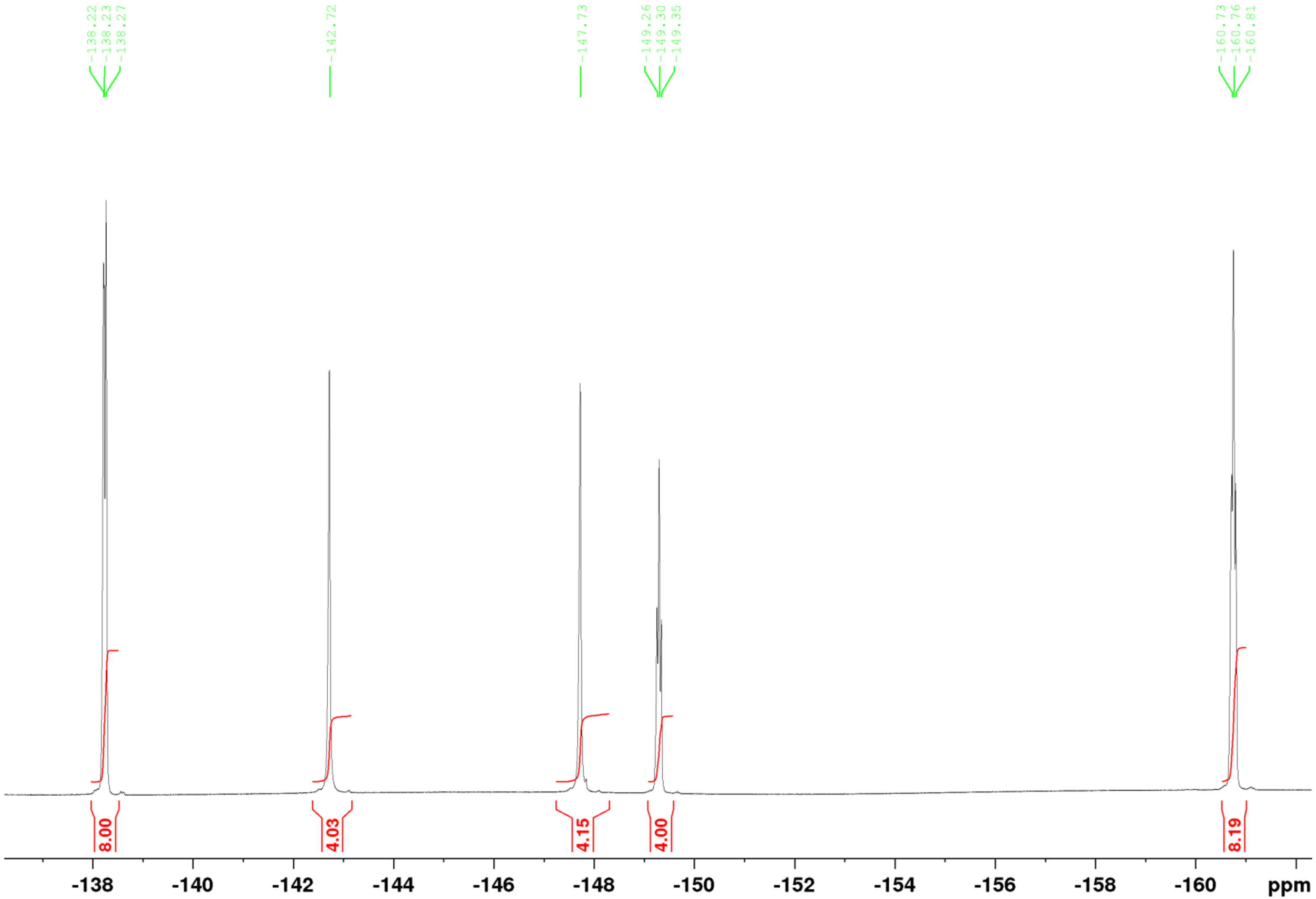

H_2TPPF_{28} (**3c**), ^{19}F NMR, 471 MHz, $CDCl_3$, 240–300 K

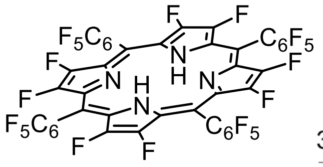

3c

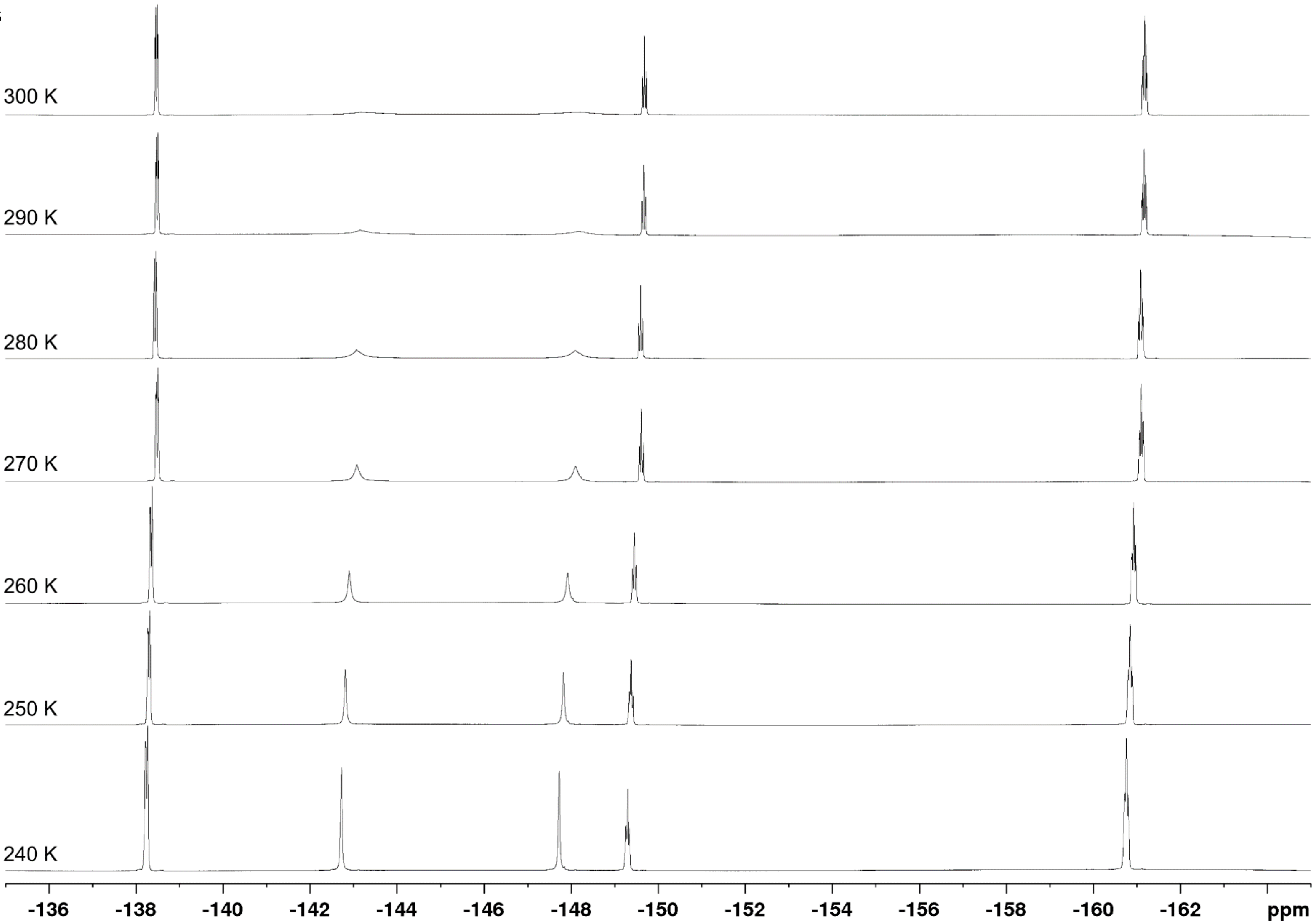

7 ^{1}H NMR, 600 MHz, [D$_6$]acetone

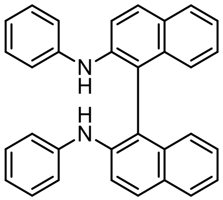

7

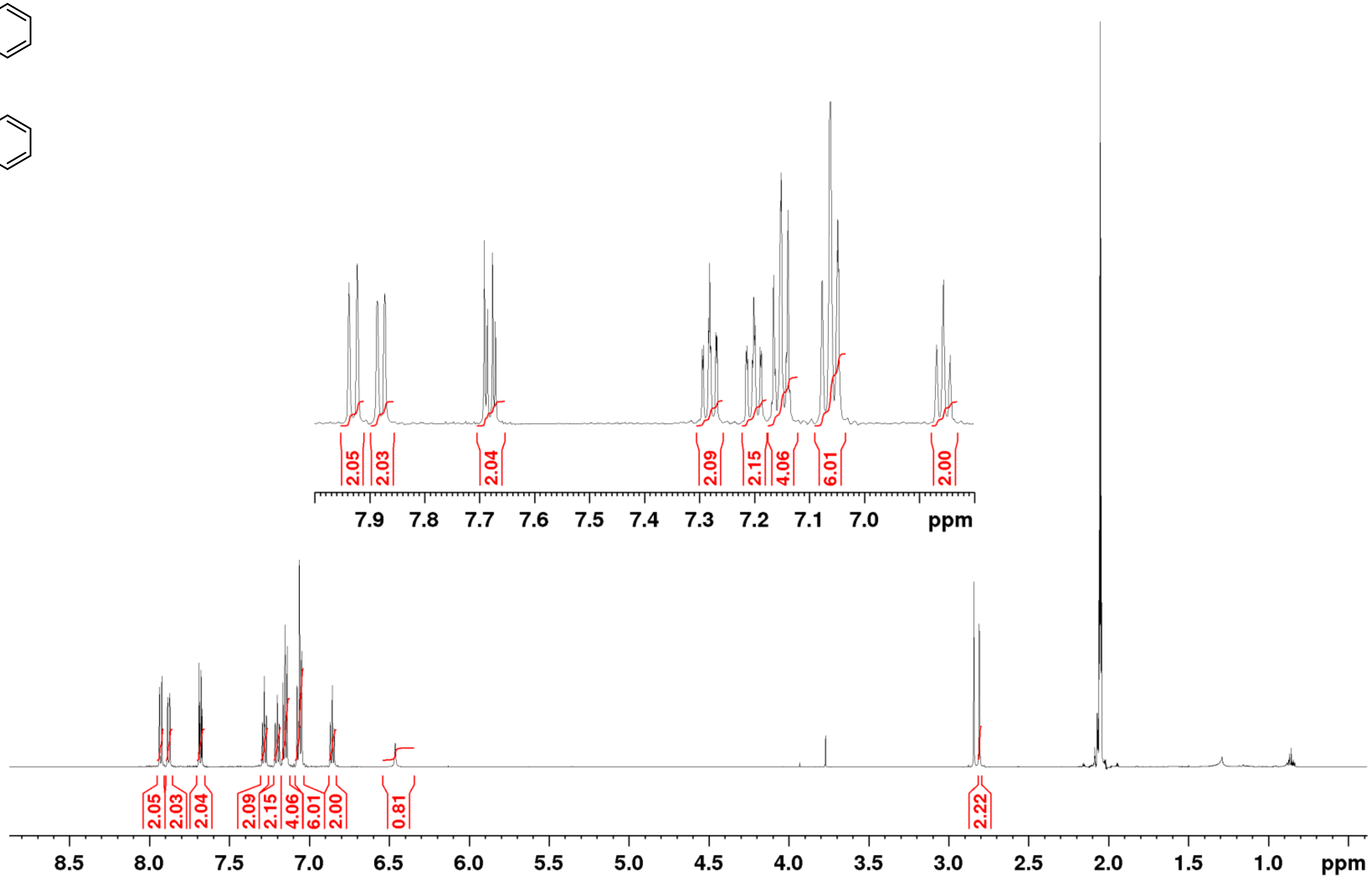

7 ^{13}C NMR, 151 MHz, [D$_6$]acetone

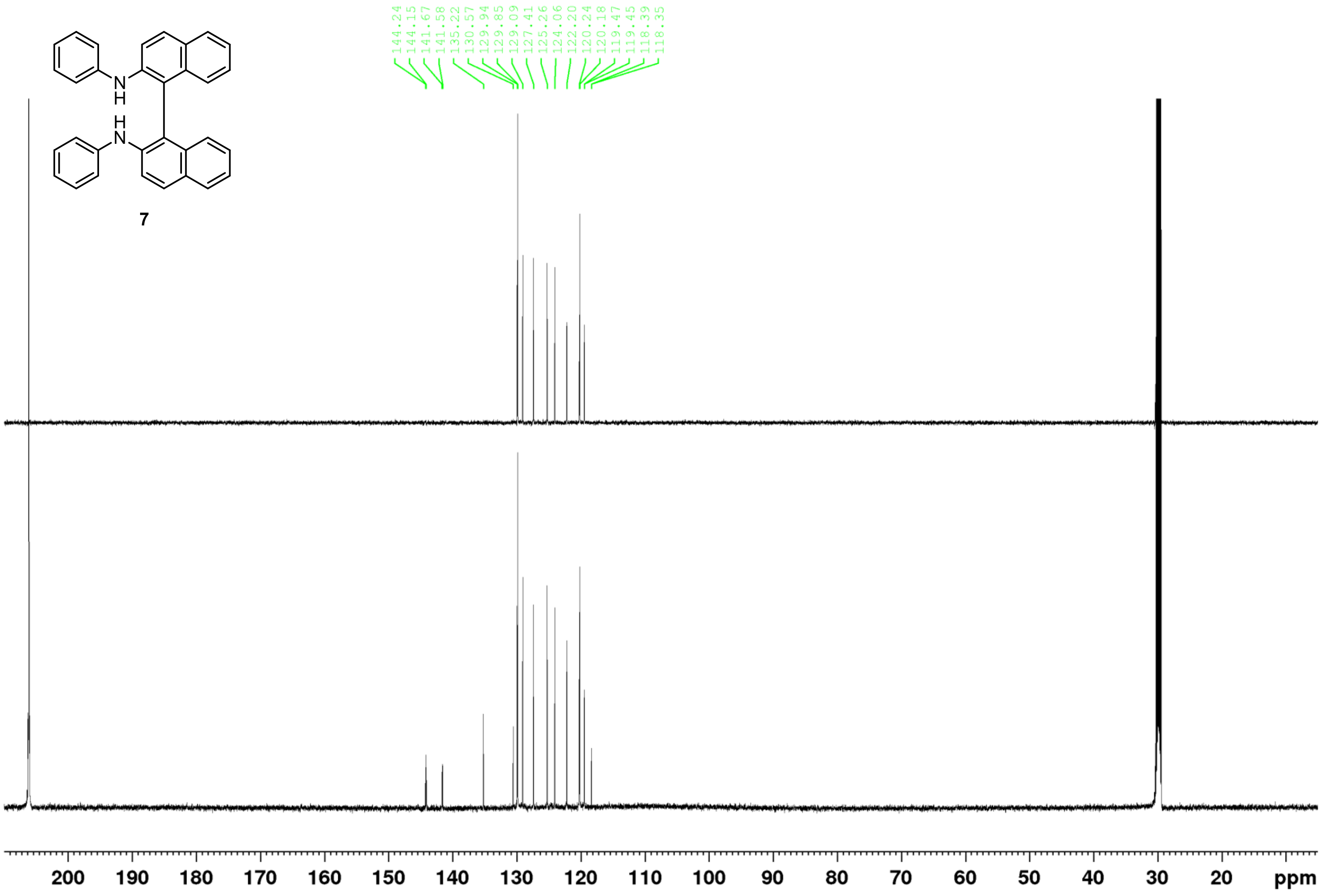

7 ^{13}C NMR, 151 MHz, [D$_6$]acetone, detail

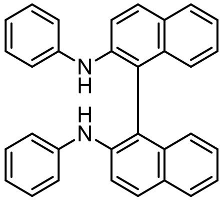

7

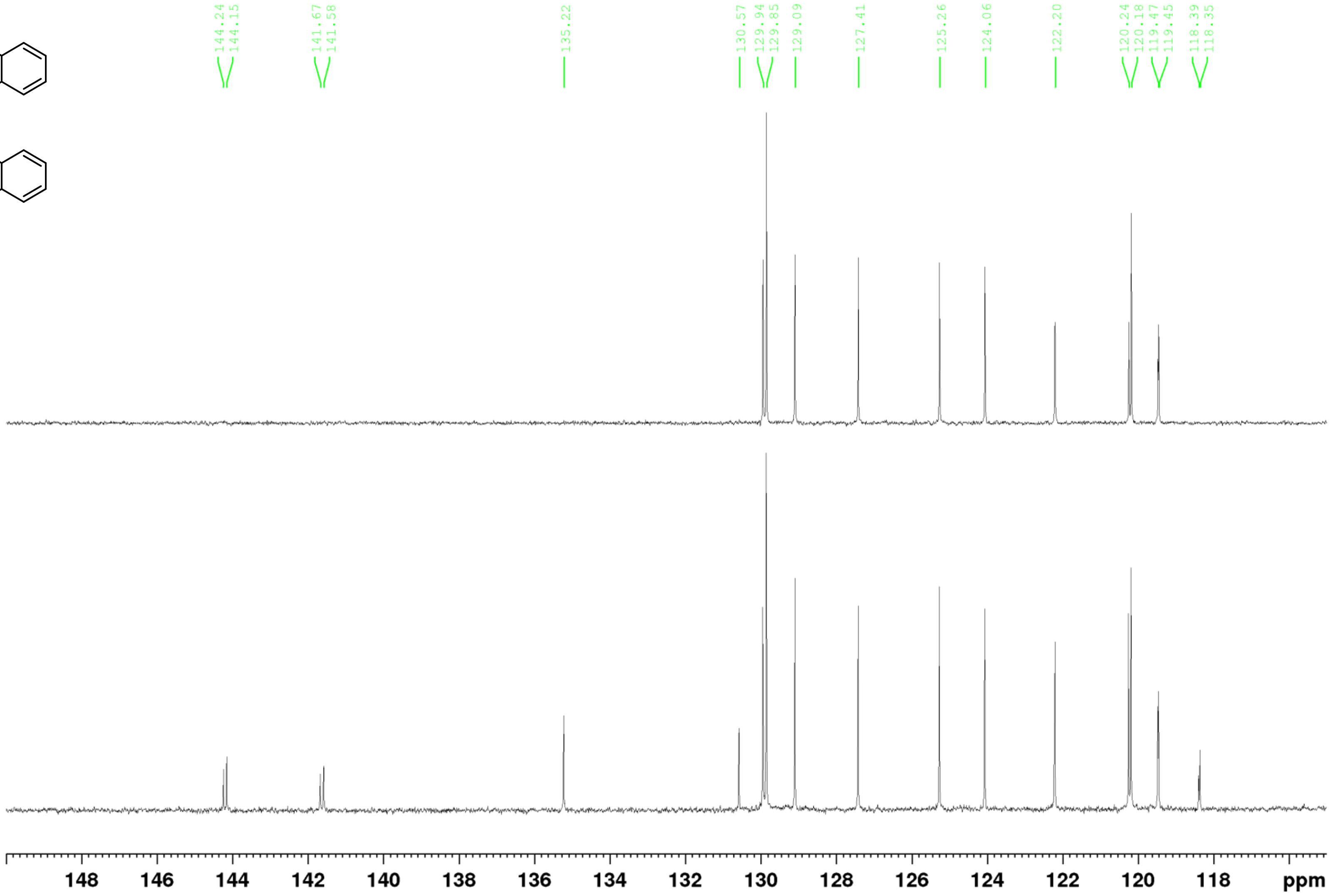

7 ^{1}H NMR, 600 MHz, [D$_6$]DMSO

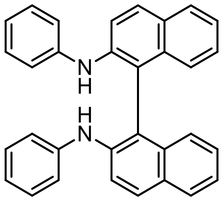

7

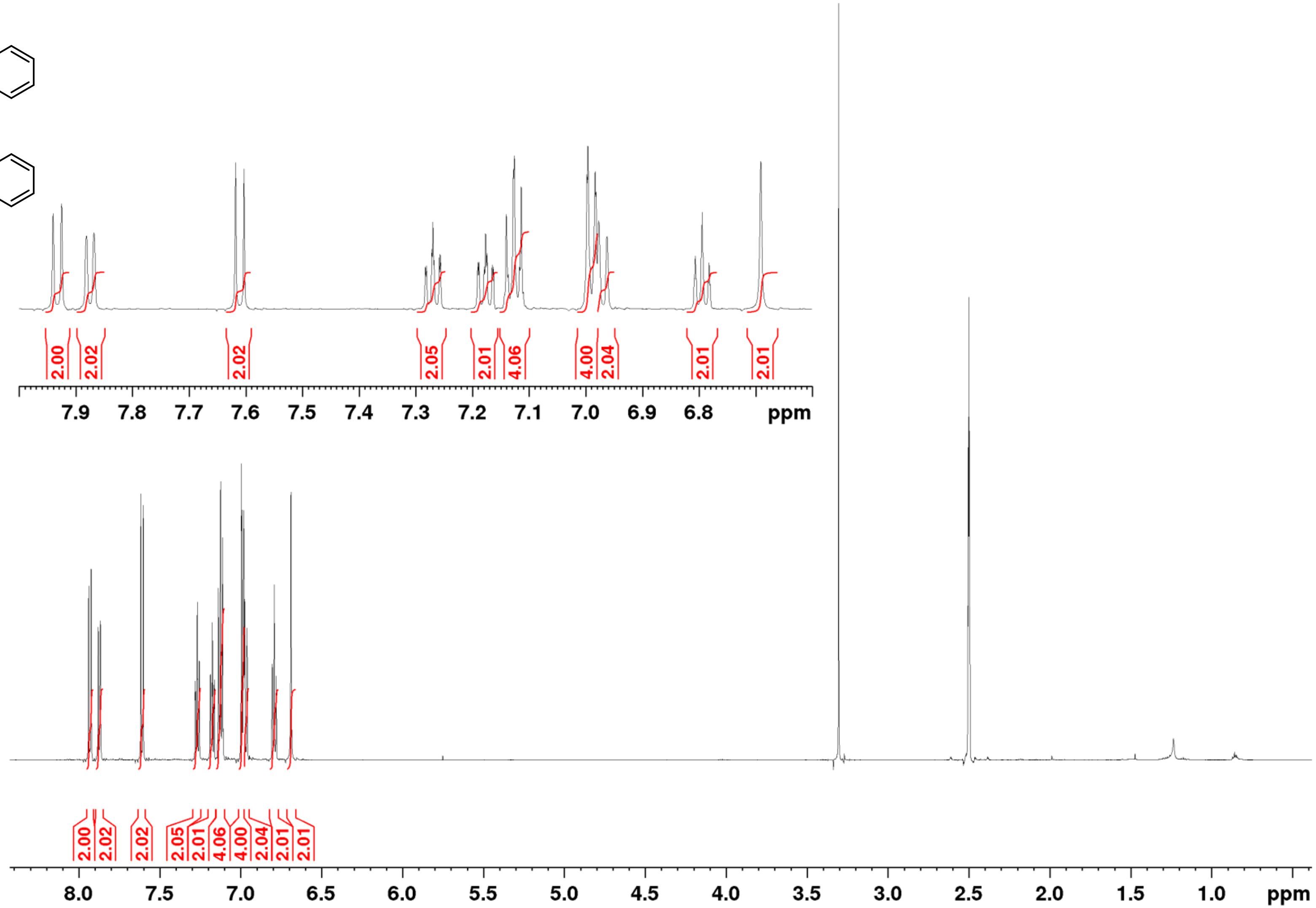

7 ^{13}C NMR, 600 MHz, [D$_6$]DMSO

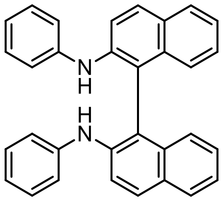

7

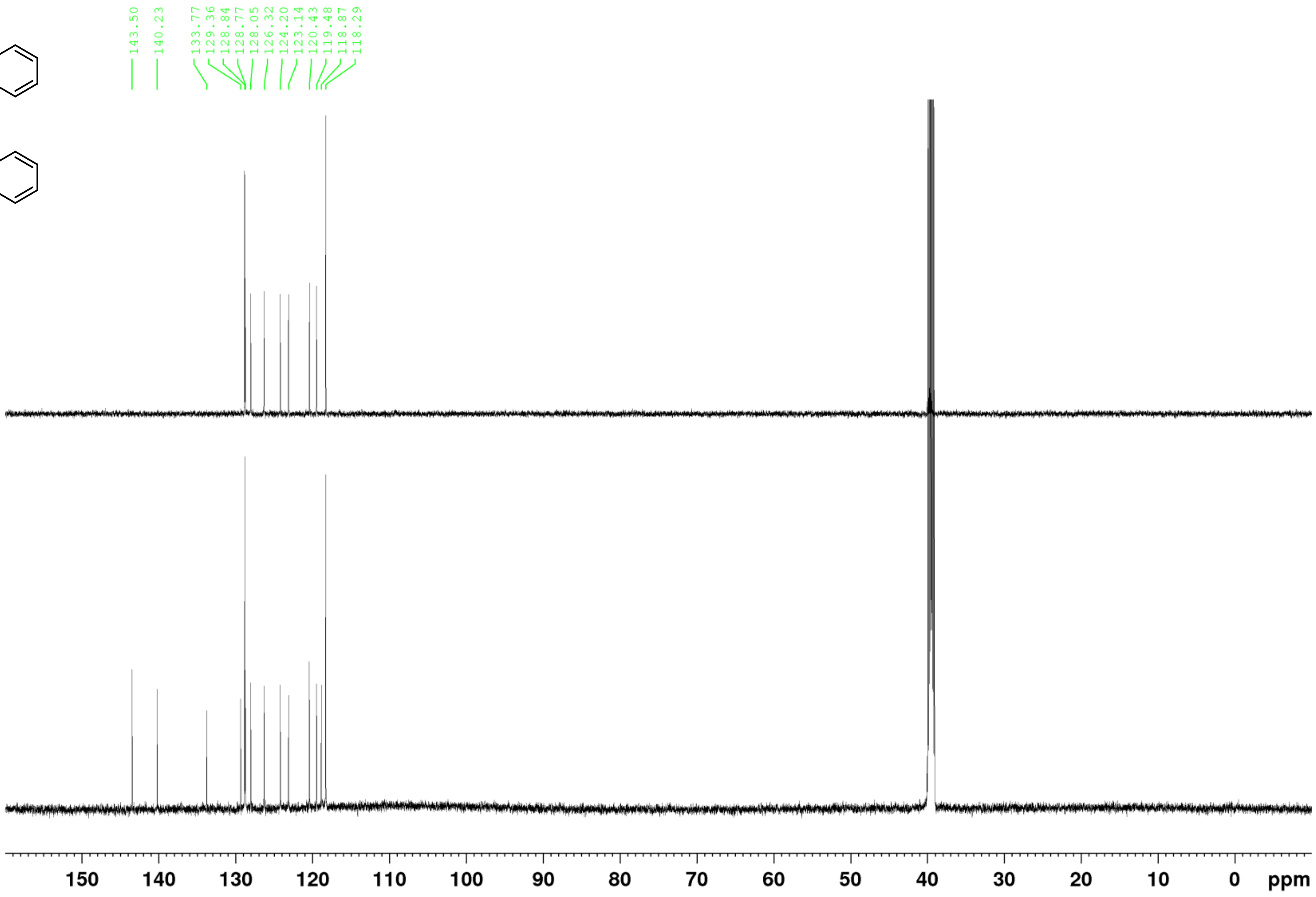

9 ^{1}H NMR, 600 MHz, $CDCl_3$

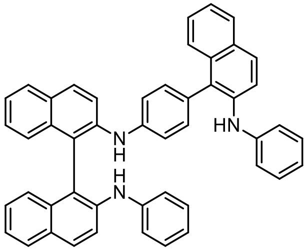

9

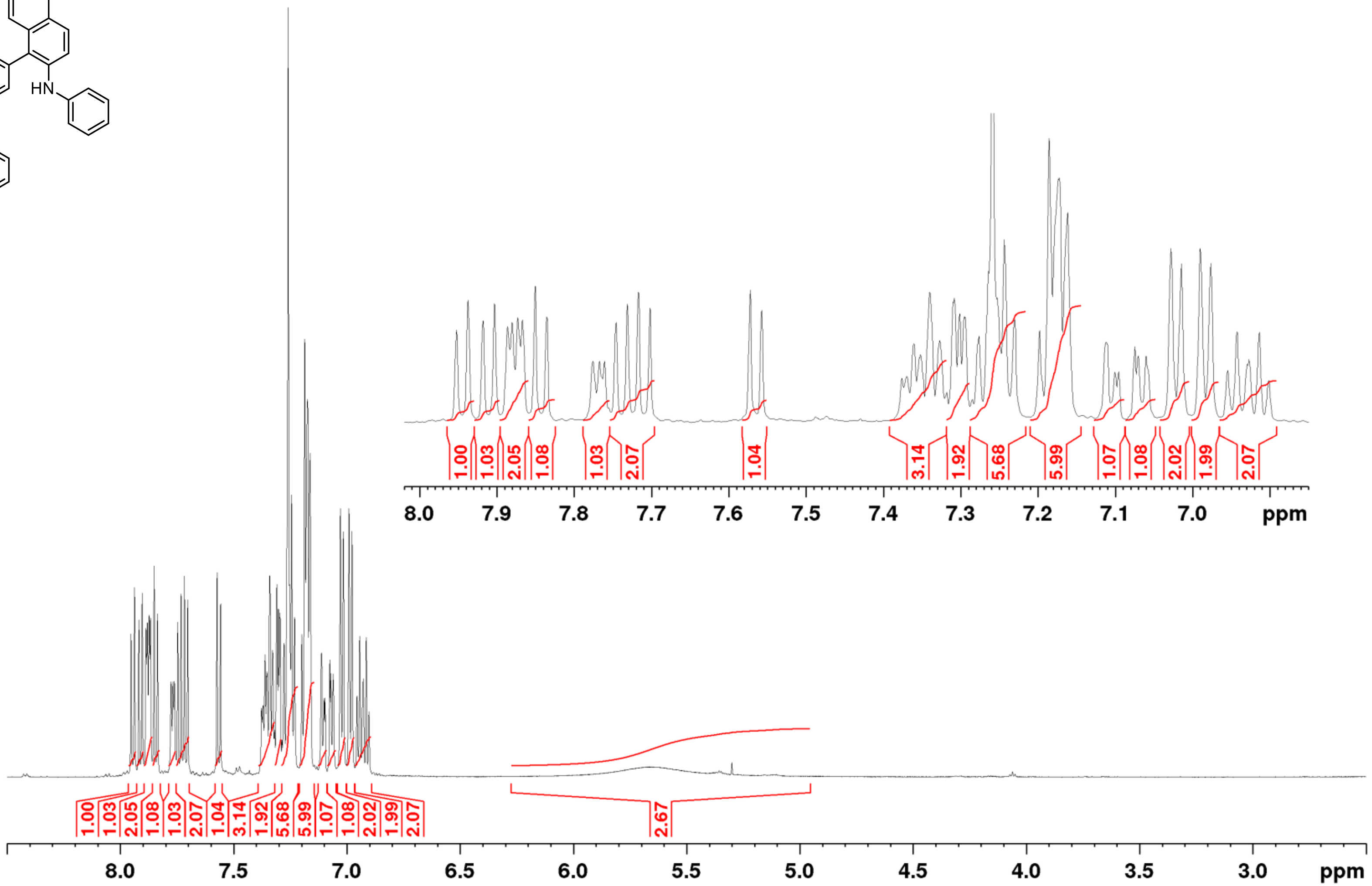

9 ^{13}C NMR, 151 MHz, $CDCl_3$

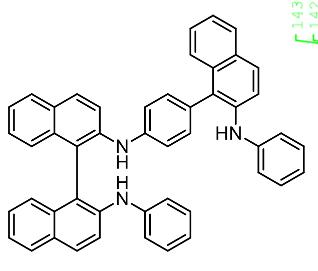

9

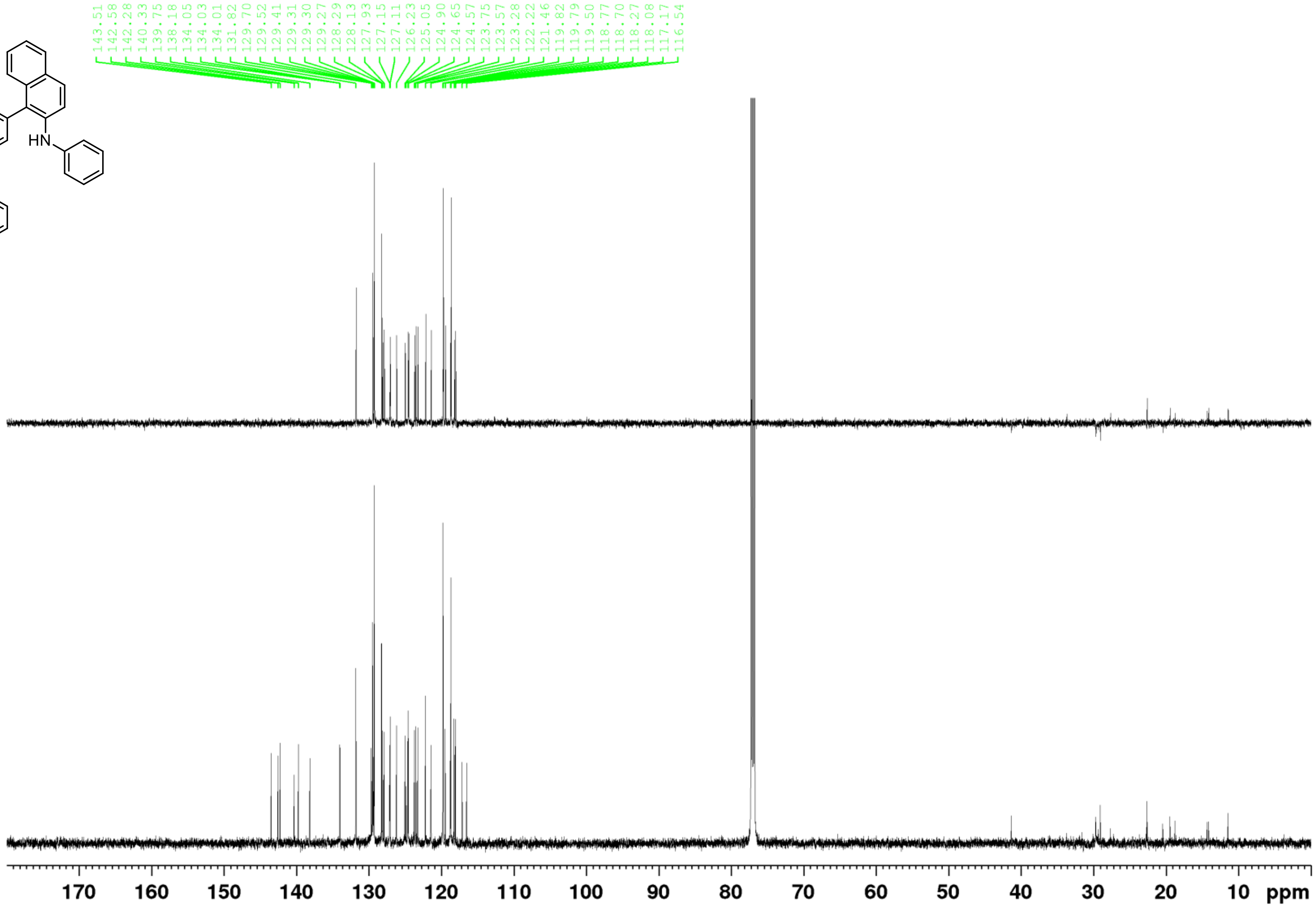

9 ^{13}C NMR, 151 MHz, $CDCl_3$, detail

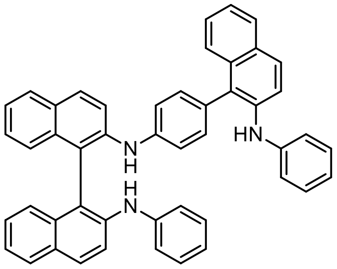

9

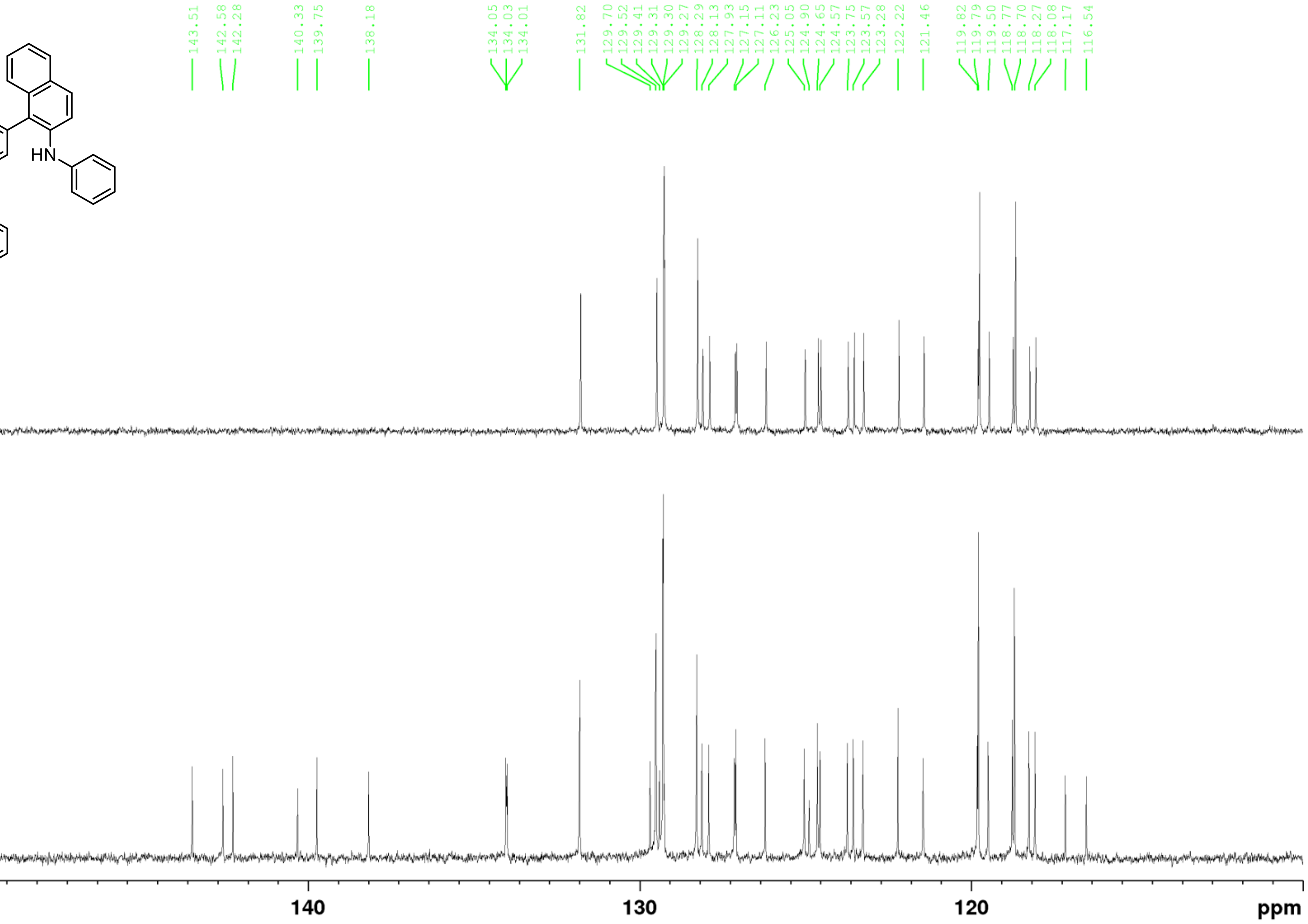

9 COSY, 600 MHz, $CDCl_3$

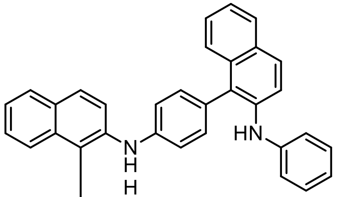

9

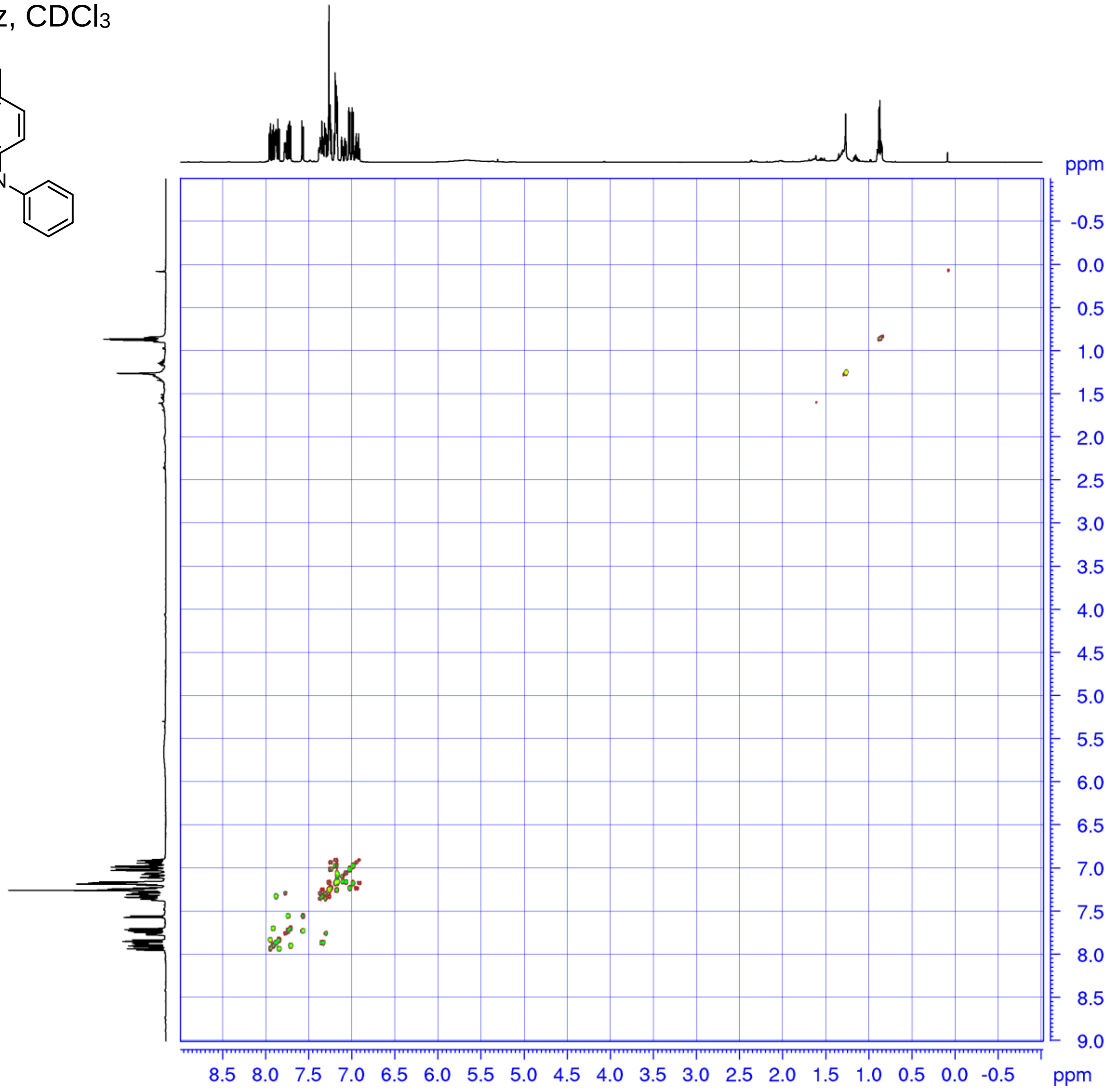

9 COSY, 600MHz, $CDCl_3$, detail

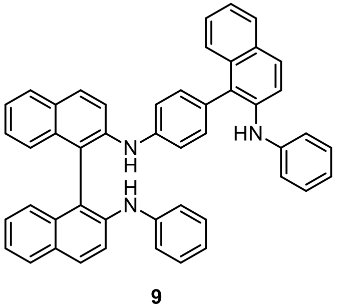

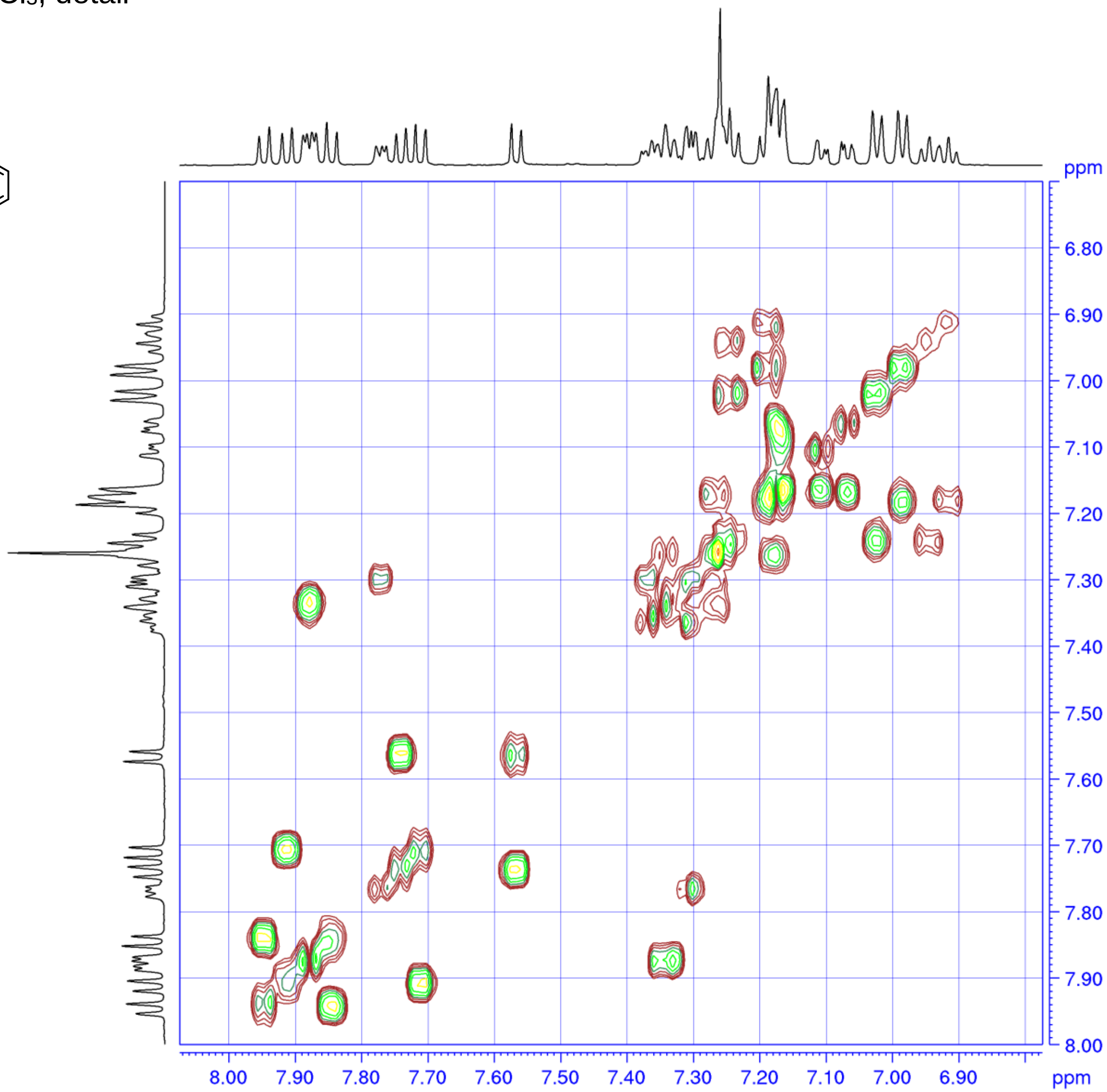

9 HMBC 600/151 MHz, $CDCl_3$

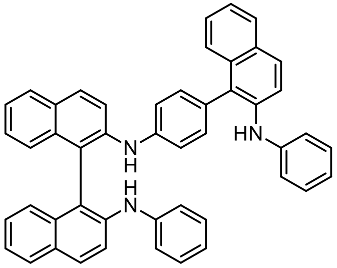

9

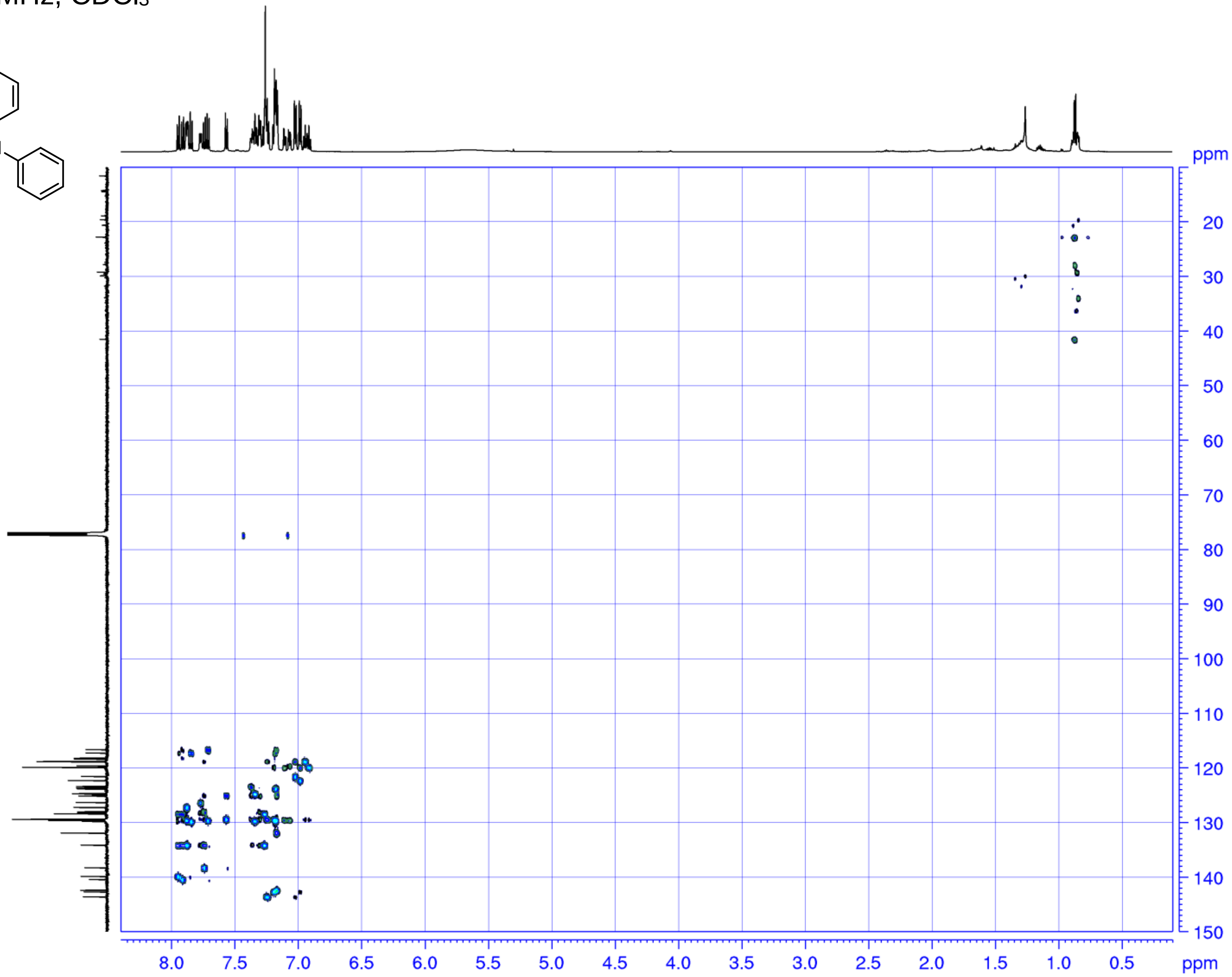

9 HMBC 600/151 MHz, $CDCl_3$, detail

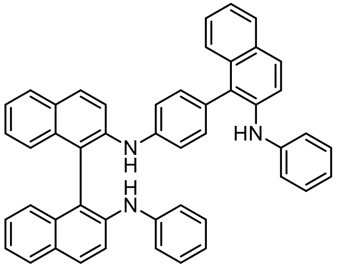

9

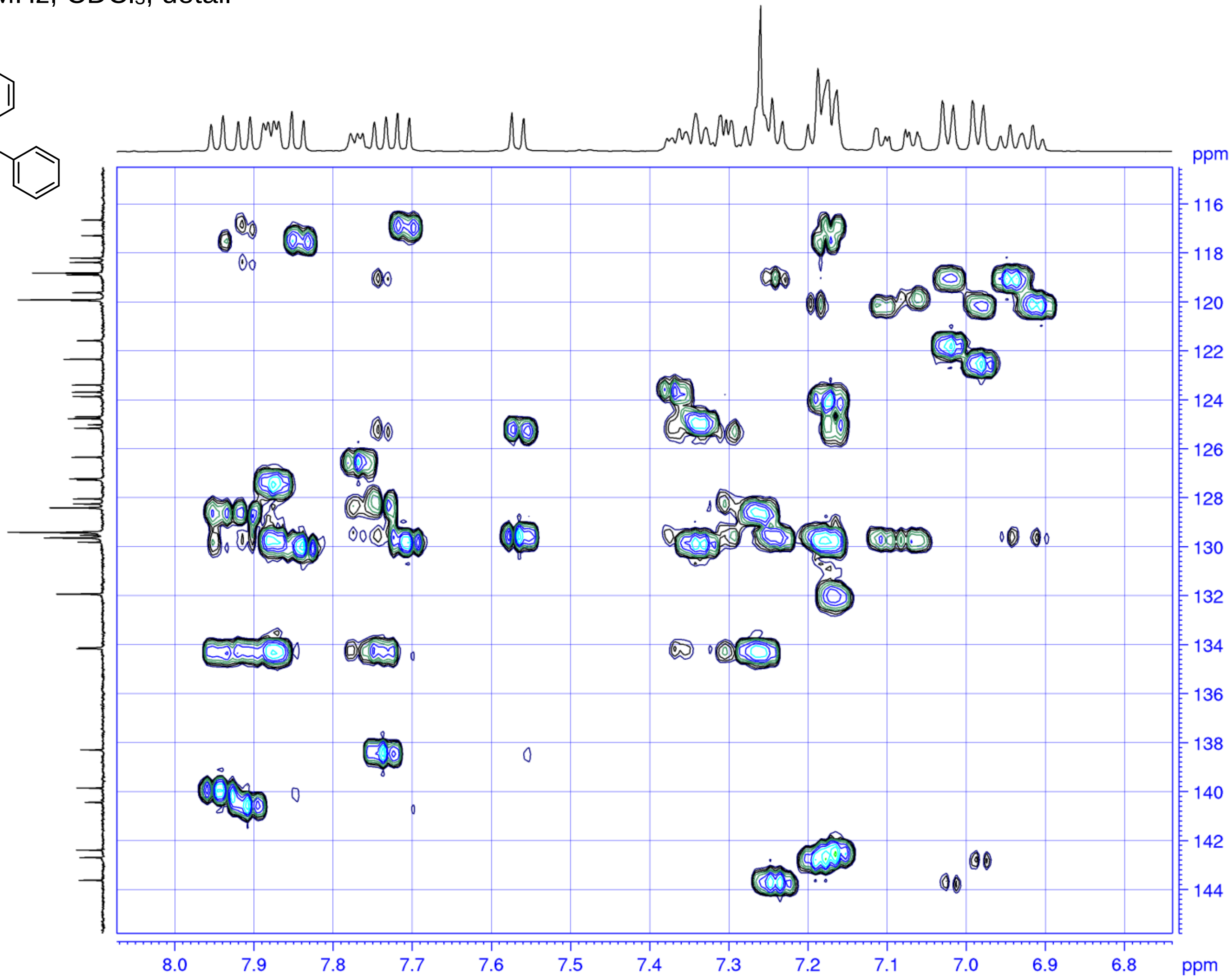

9 HSQC, 600/151 MHz, $CDCl_3$

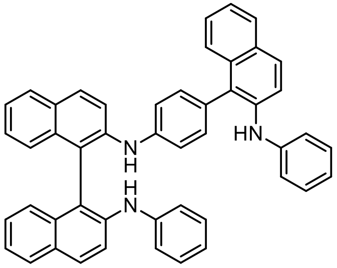

9

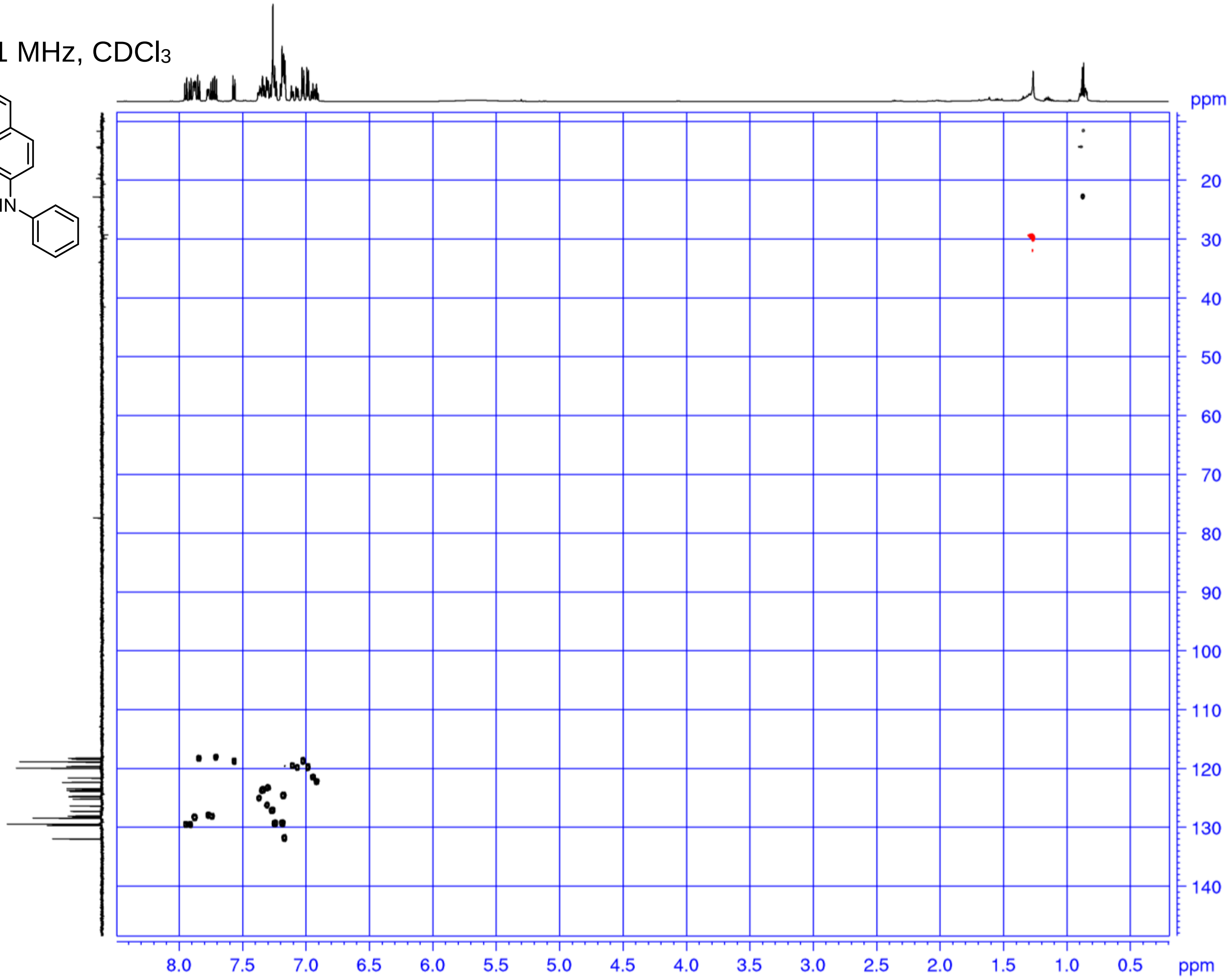

9 HSQC, 600/151 MHz, $CDCl_3$, detail

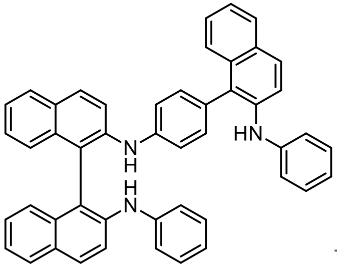

9

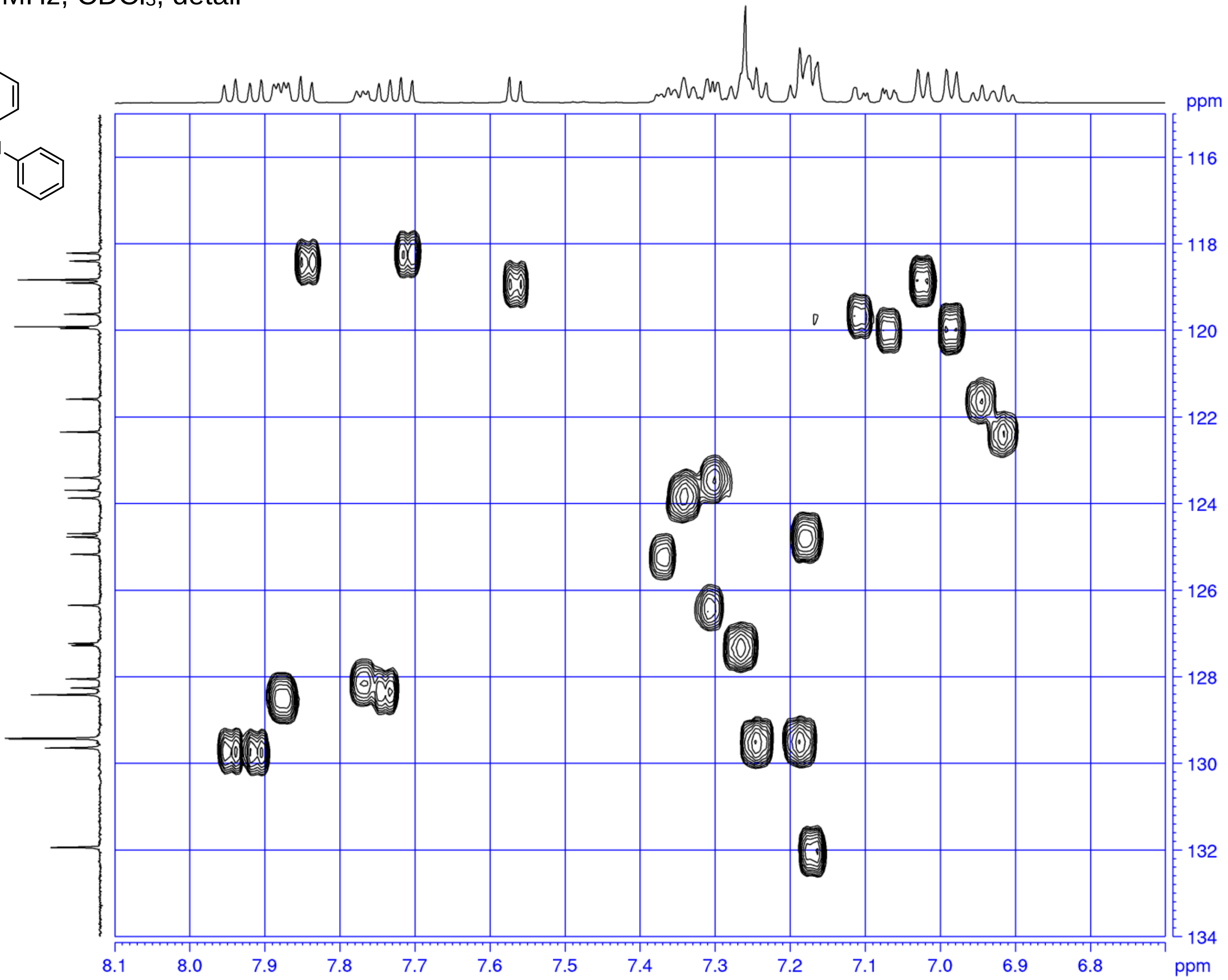

9 NOESY, 600 MHz, $CDCl_3$

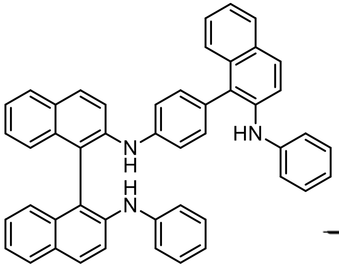

9

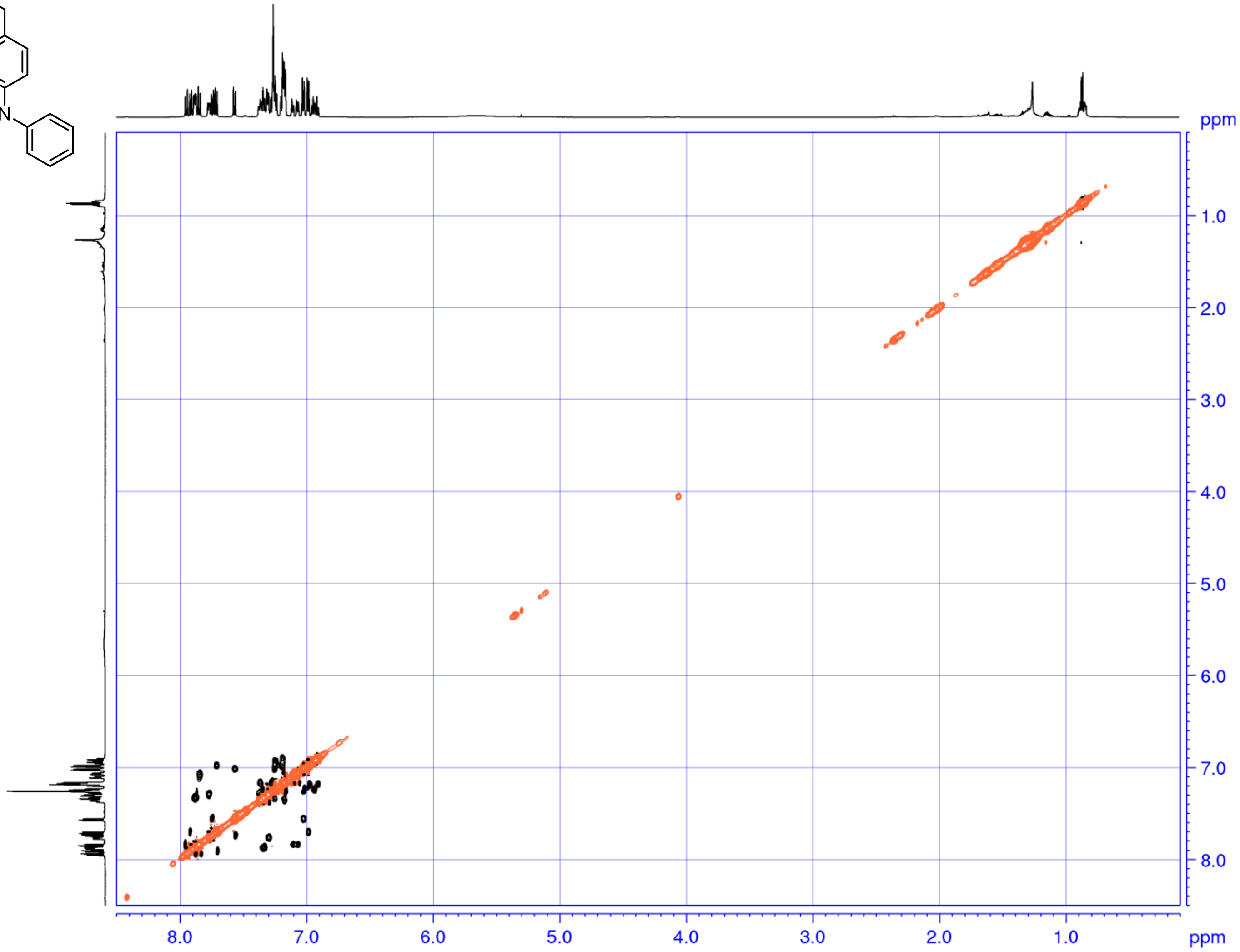

9 NOESY, 600 MHz, $CDCl_3$, detail

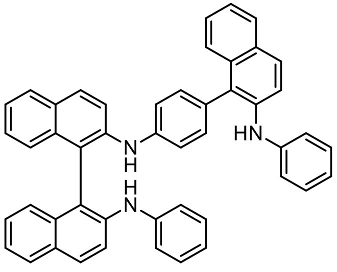

9

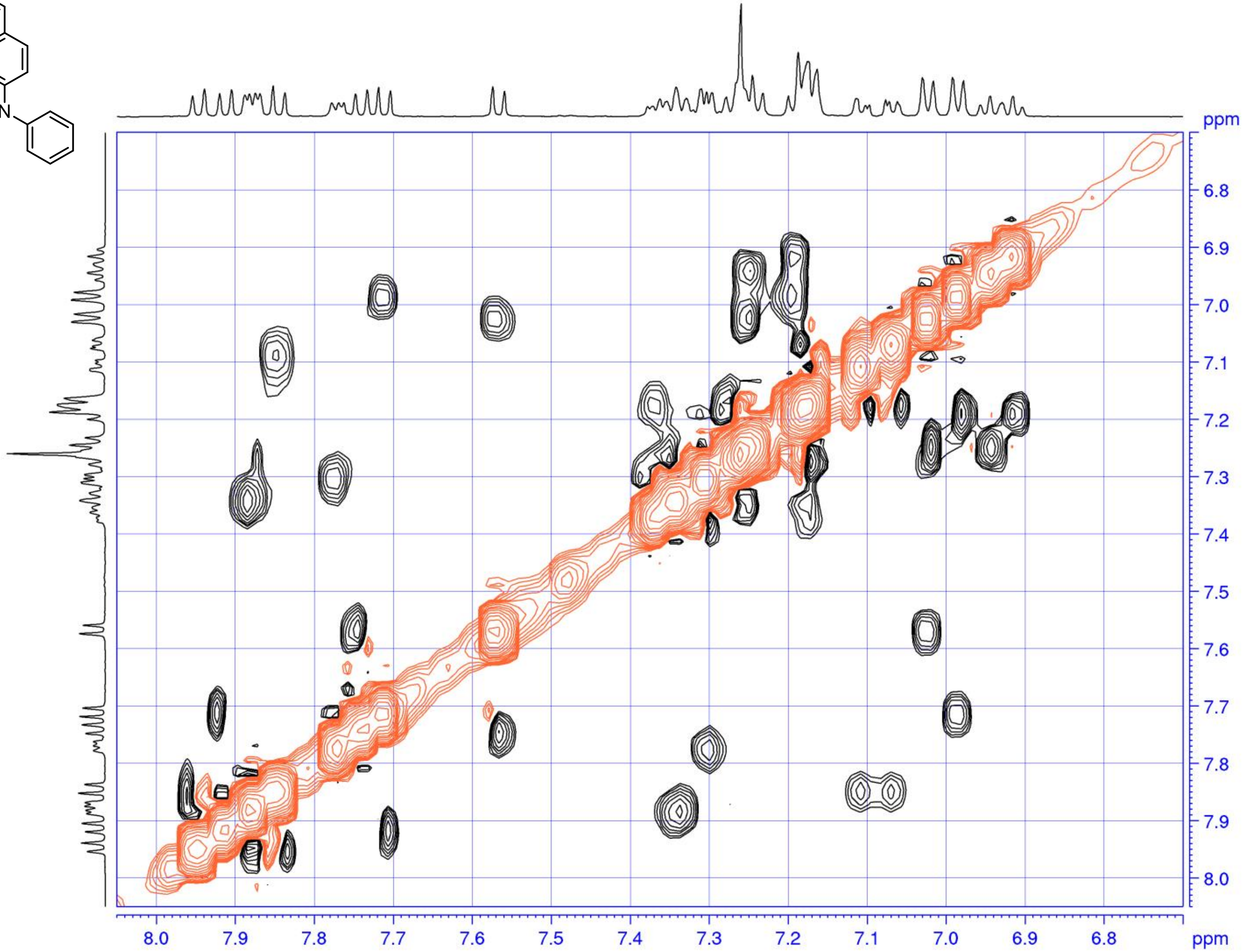

11a ^{1}H NMR, 500 MHz, $CDCl_3$

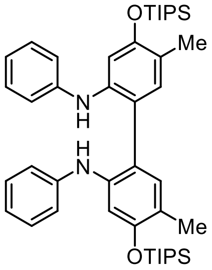

11a

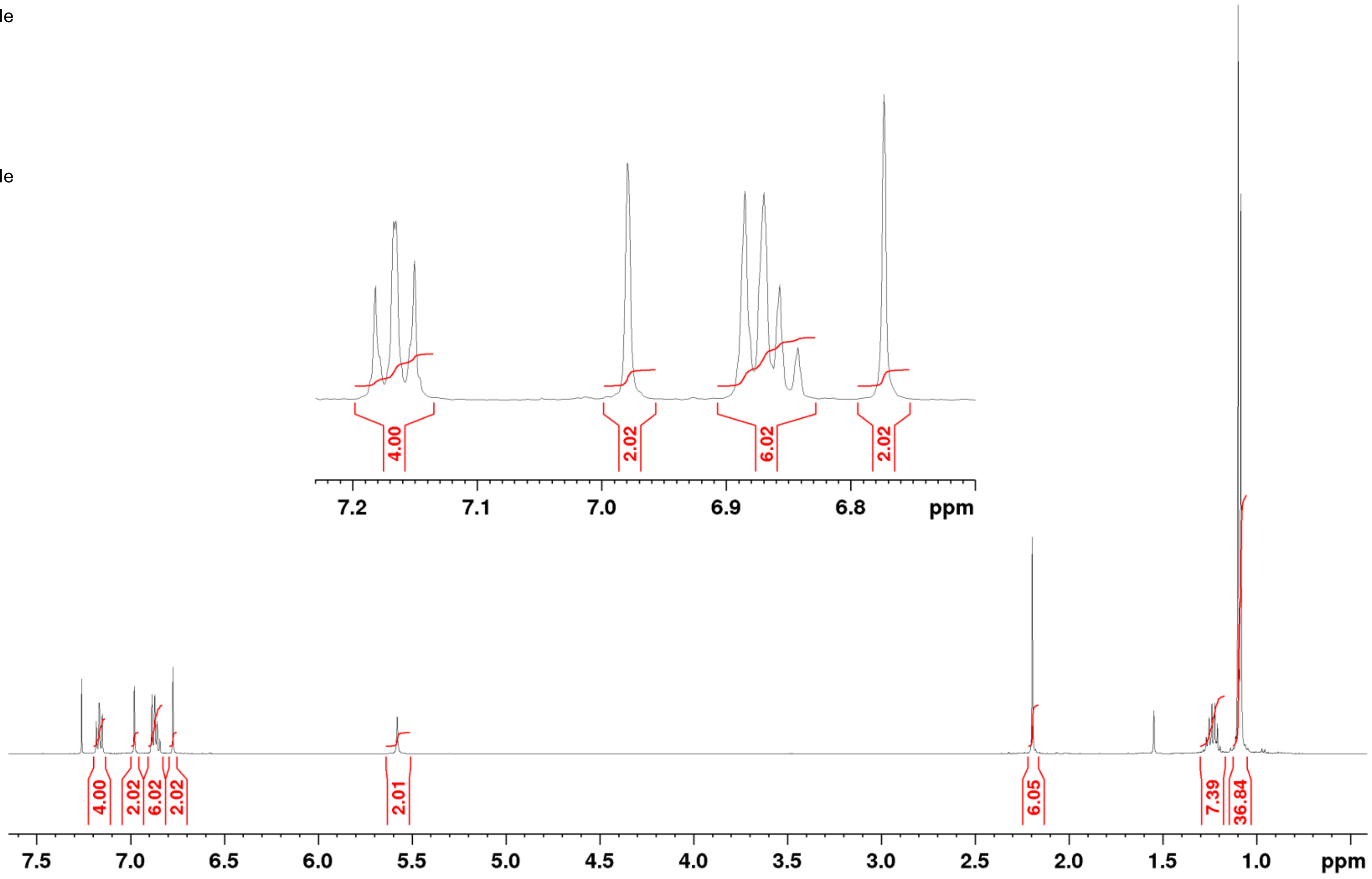

11a ^{13}C NMR, 125 MHz, $CDCl_3$

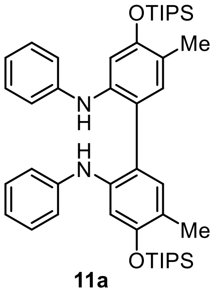

11a

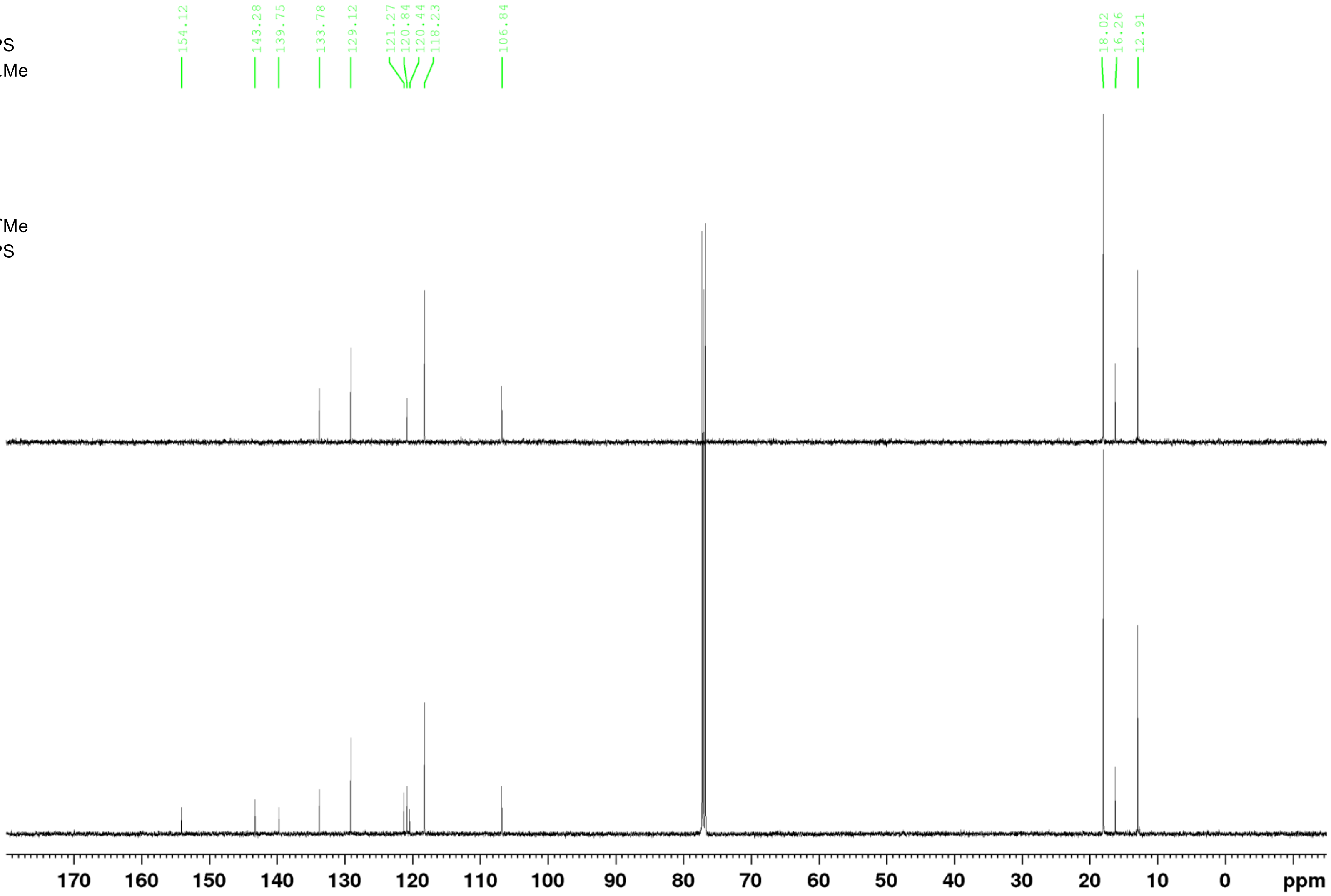

11b ^{1}H NMR, 500 MHz, $CDCl_3$

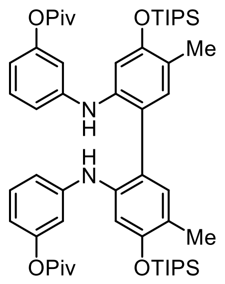

11b

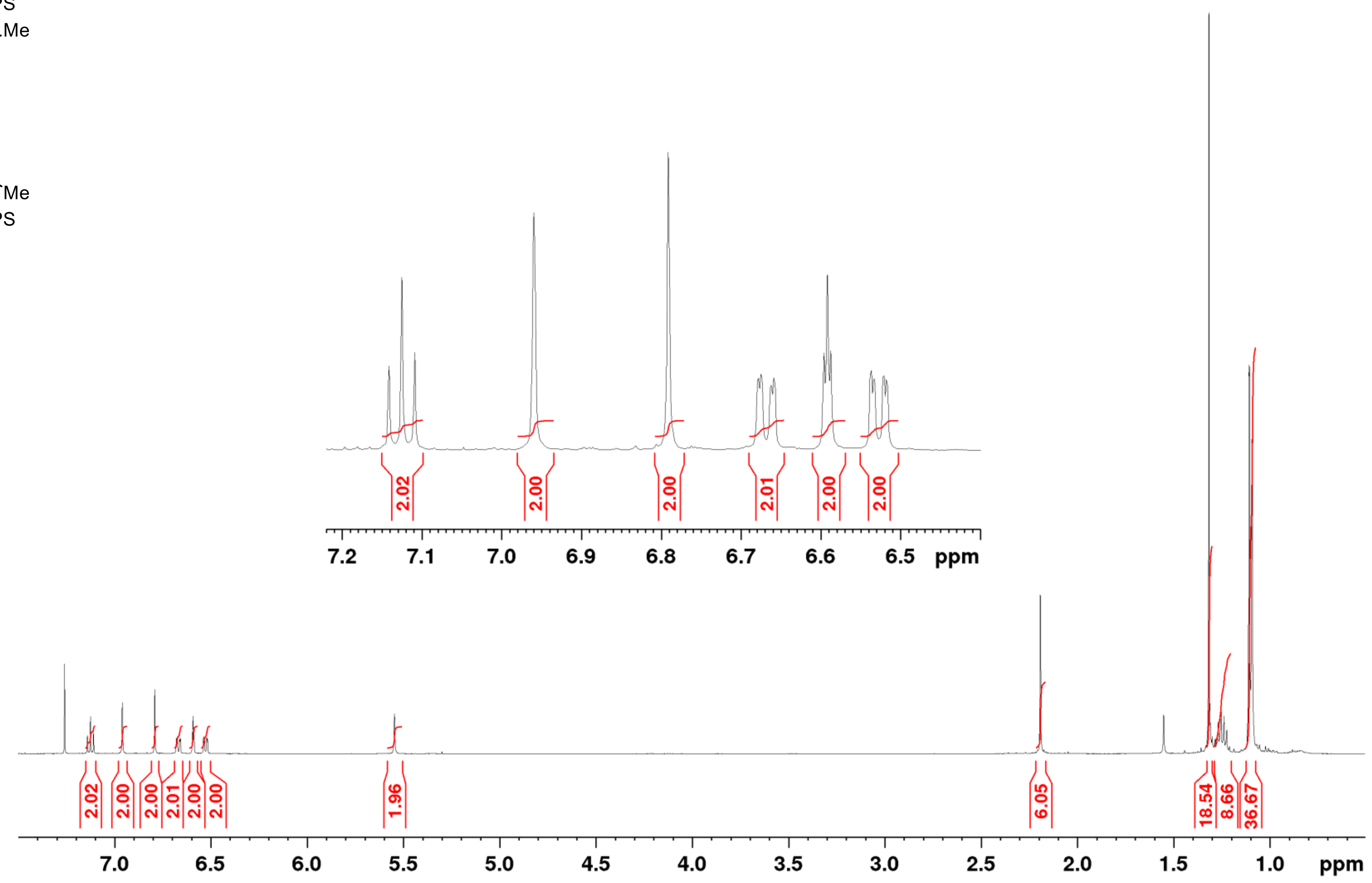

11b ^{13}C NMR, 125 MHz, $CDCl_3$

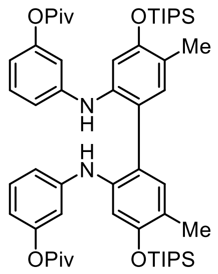

11b

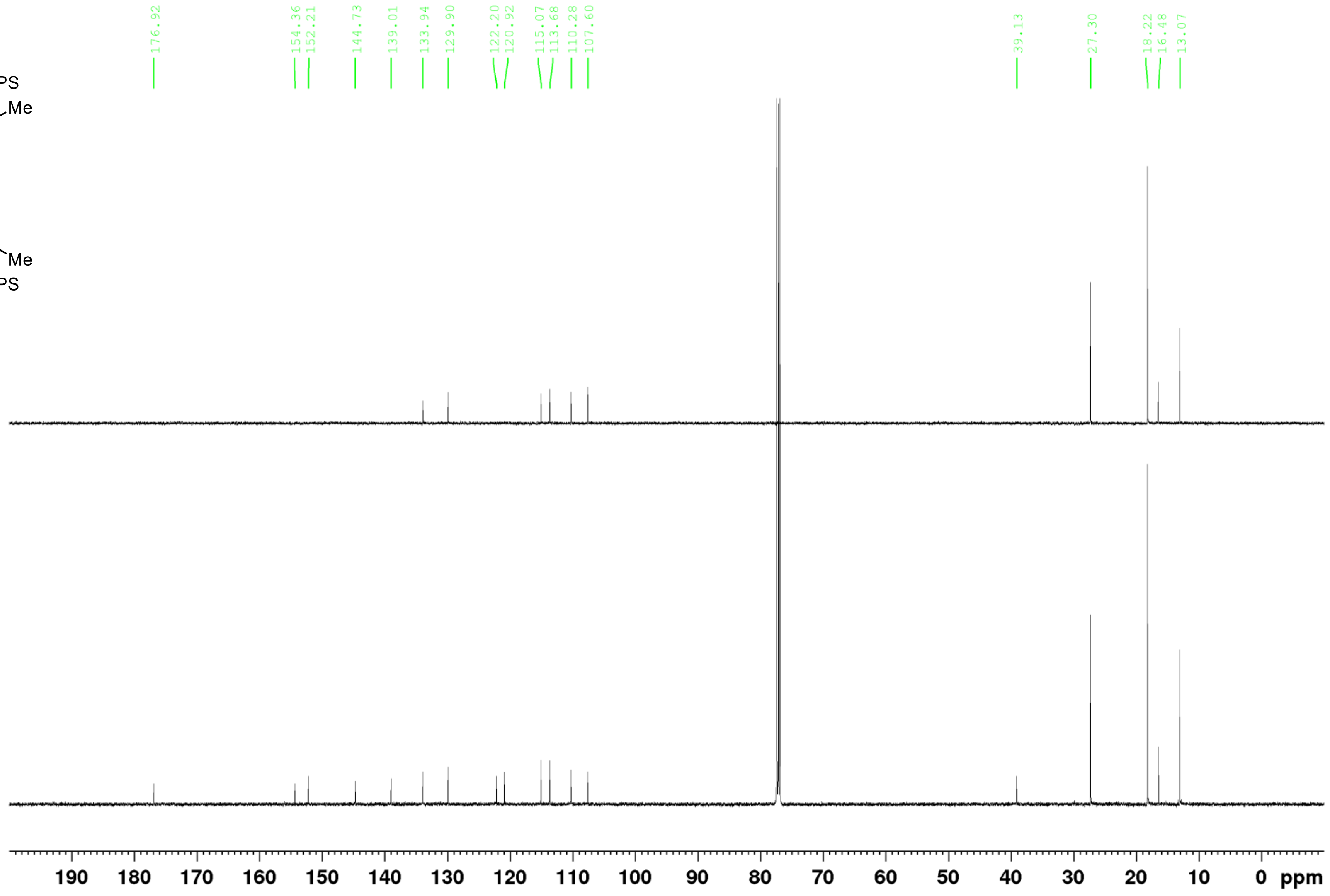

11c ^{1}H NMR, 500 MHz, $CDCl_3$

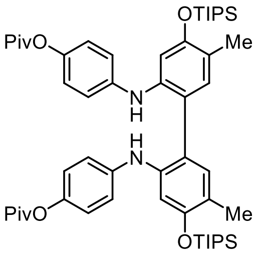

11c

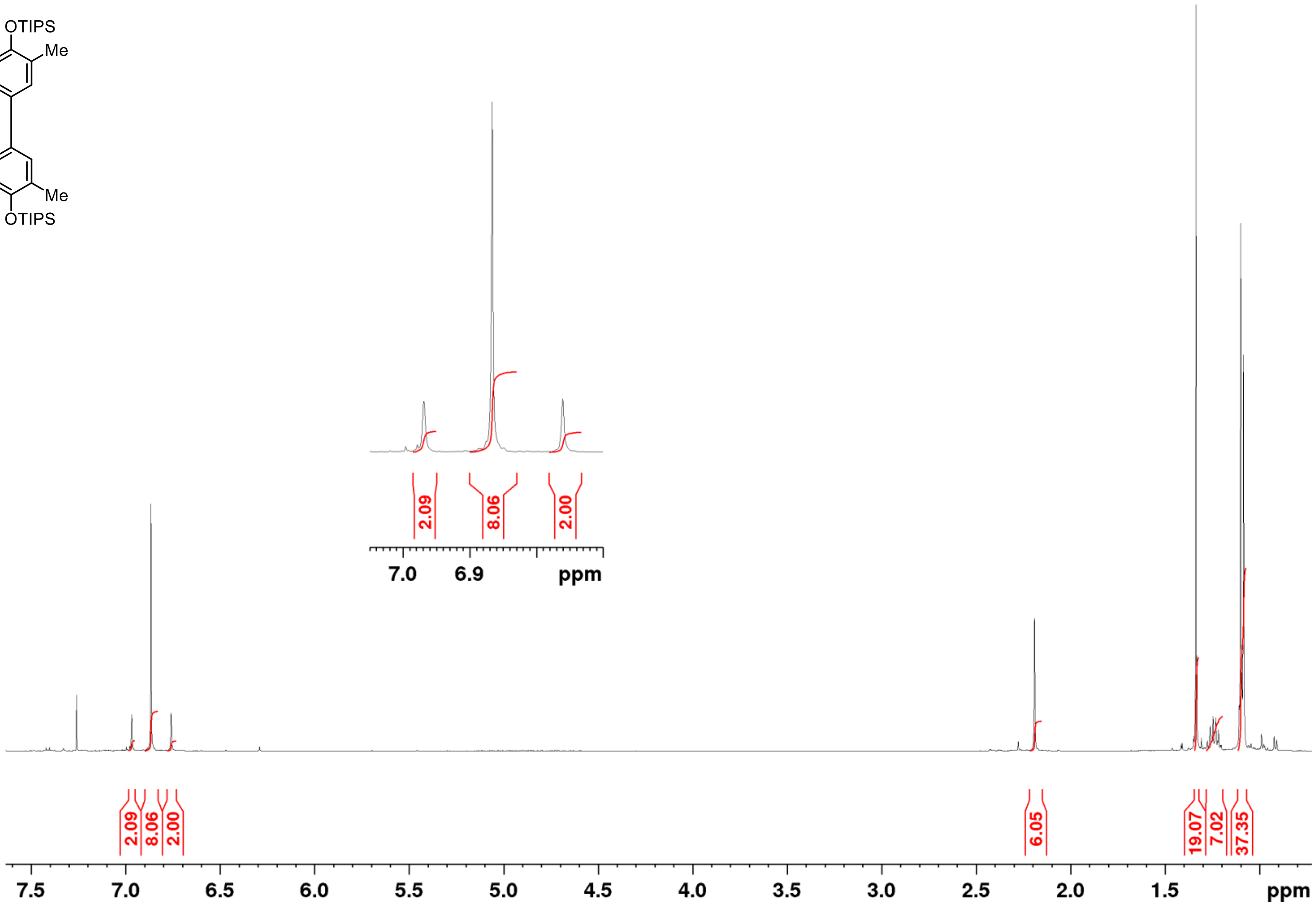

8c ^{13}C NMR, 125 MHz, $CDCl_3$

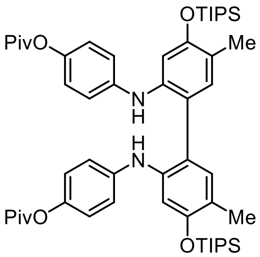

11c

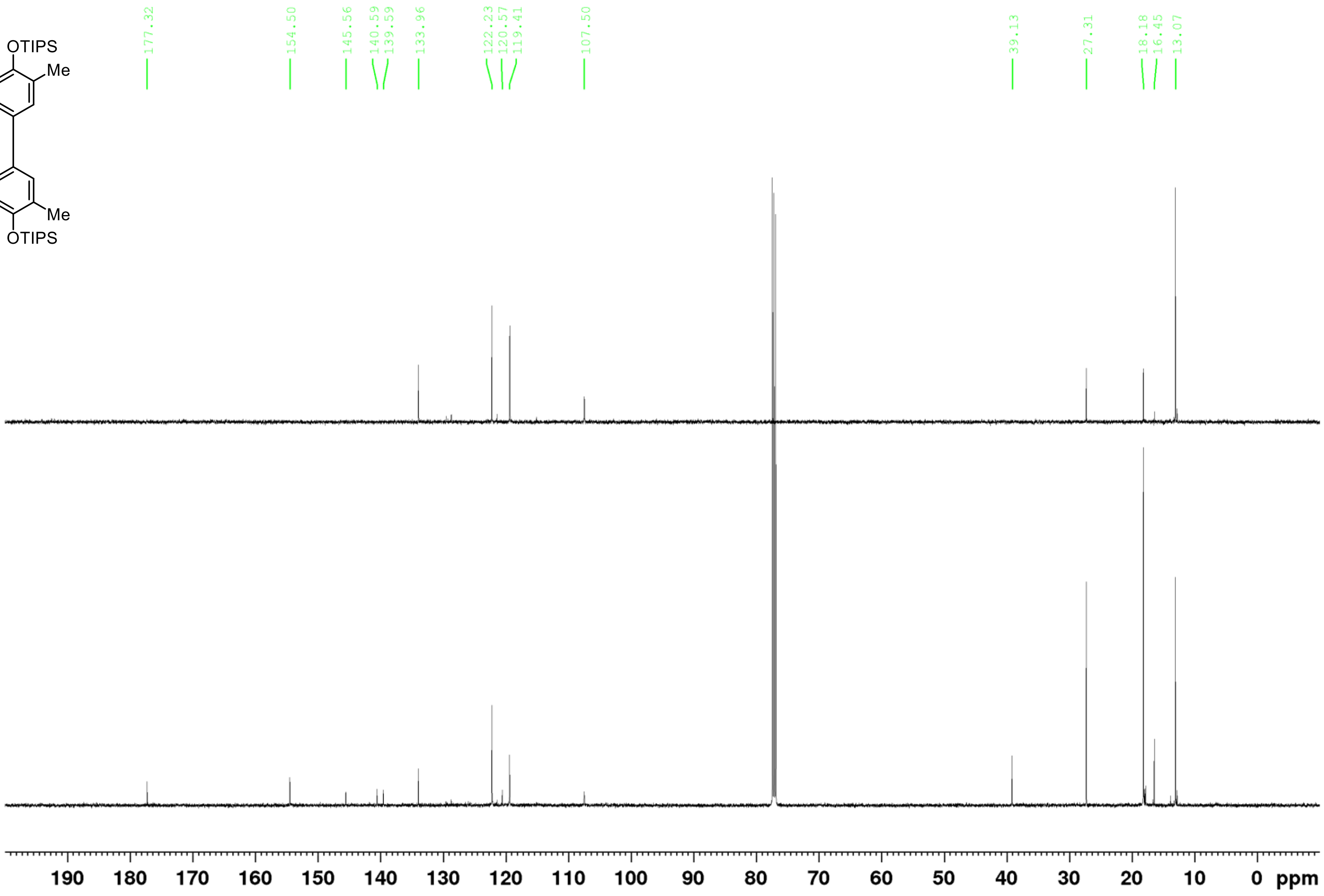

11d ^{1}H NMR, 600 MHz, $CDCl_3$

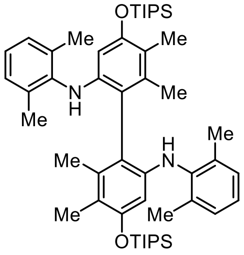

11d

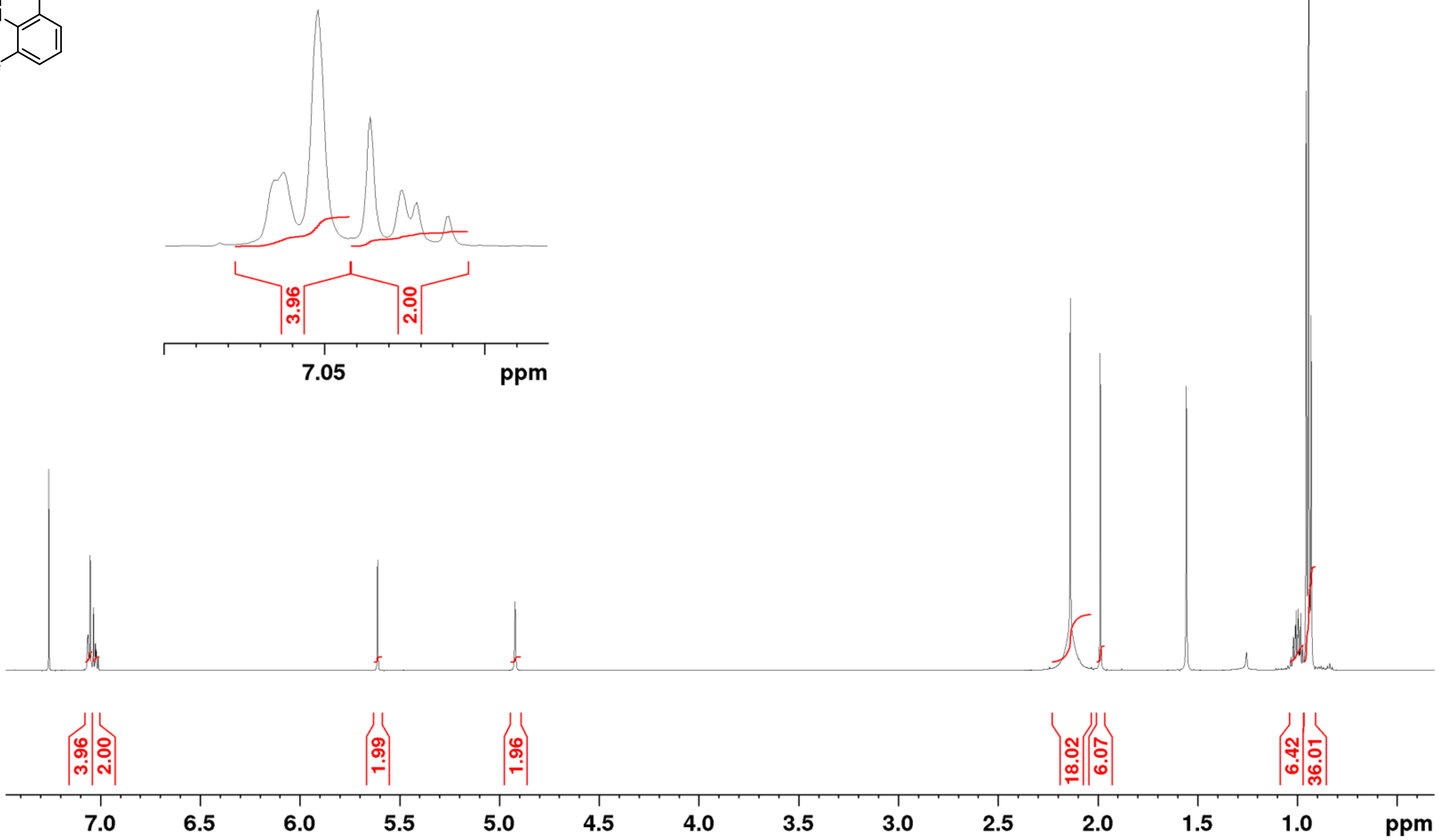

11d ^{13}C NMR, 151 MHz, $CDCl_3$

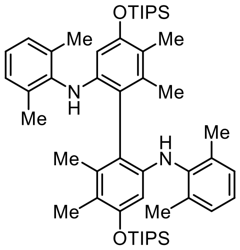

11d

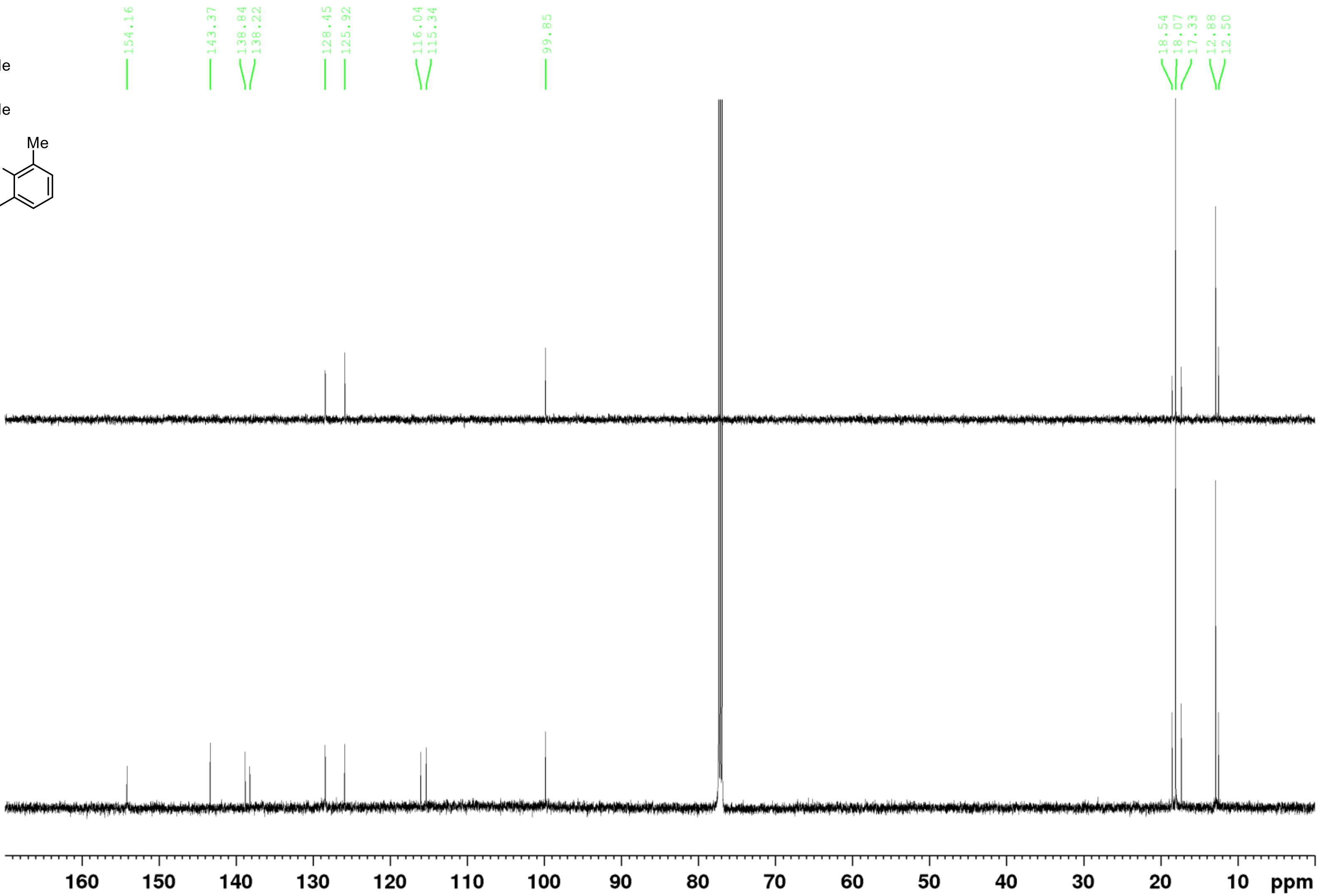

14a ^{1}H NMR, 600 MHz, [D6]acetone (traces of EtOAc)

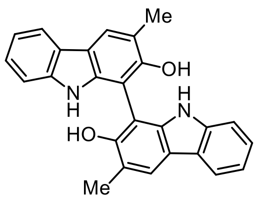

14a

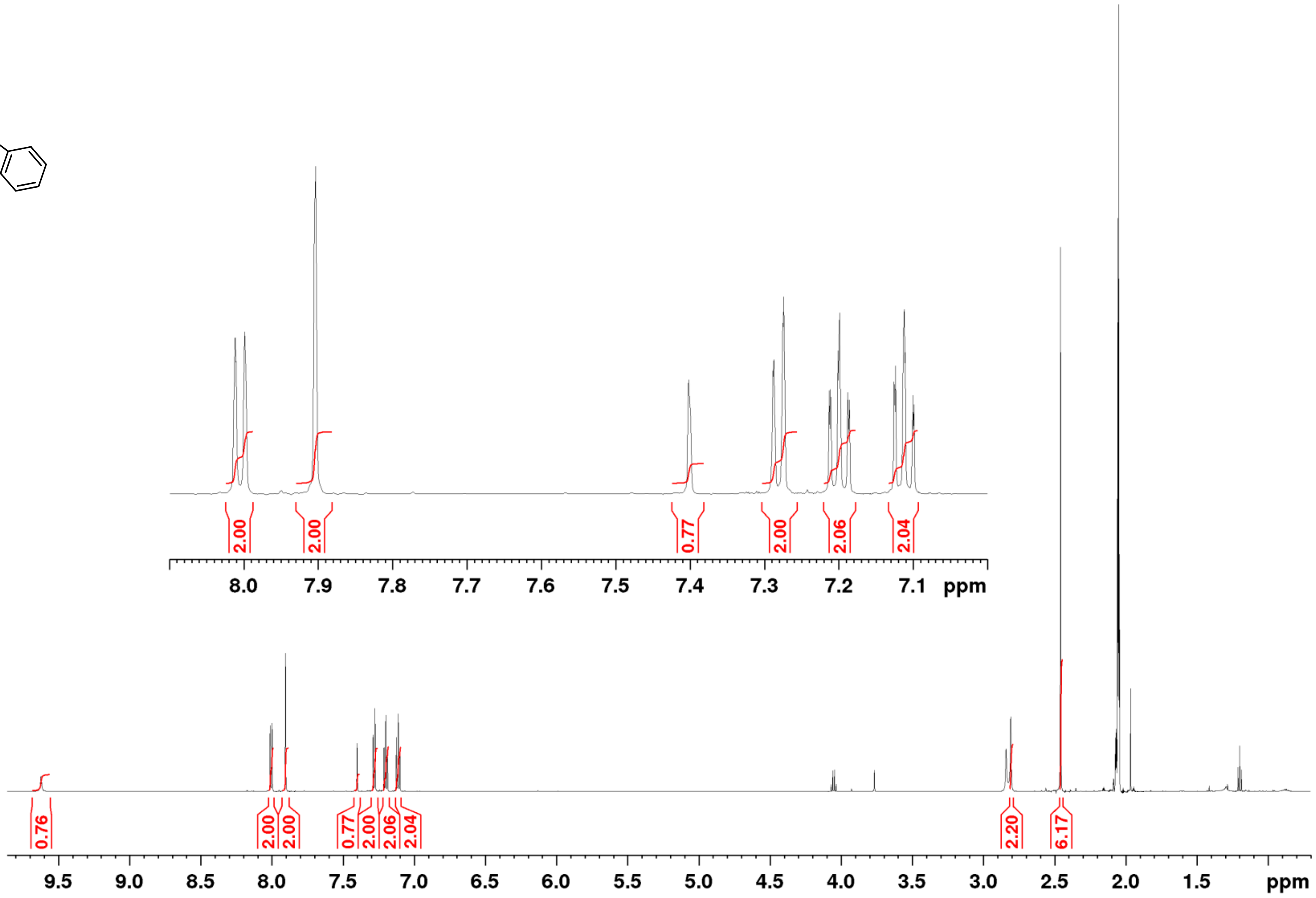

14a ^{13}C NMR, 151 MHz, [D$_6$]acetone

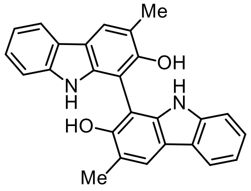

14a

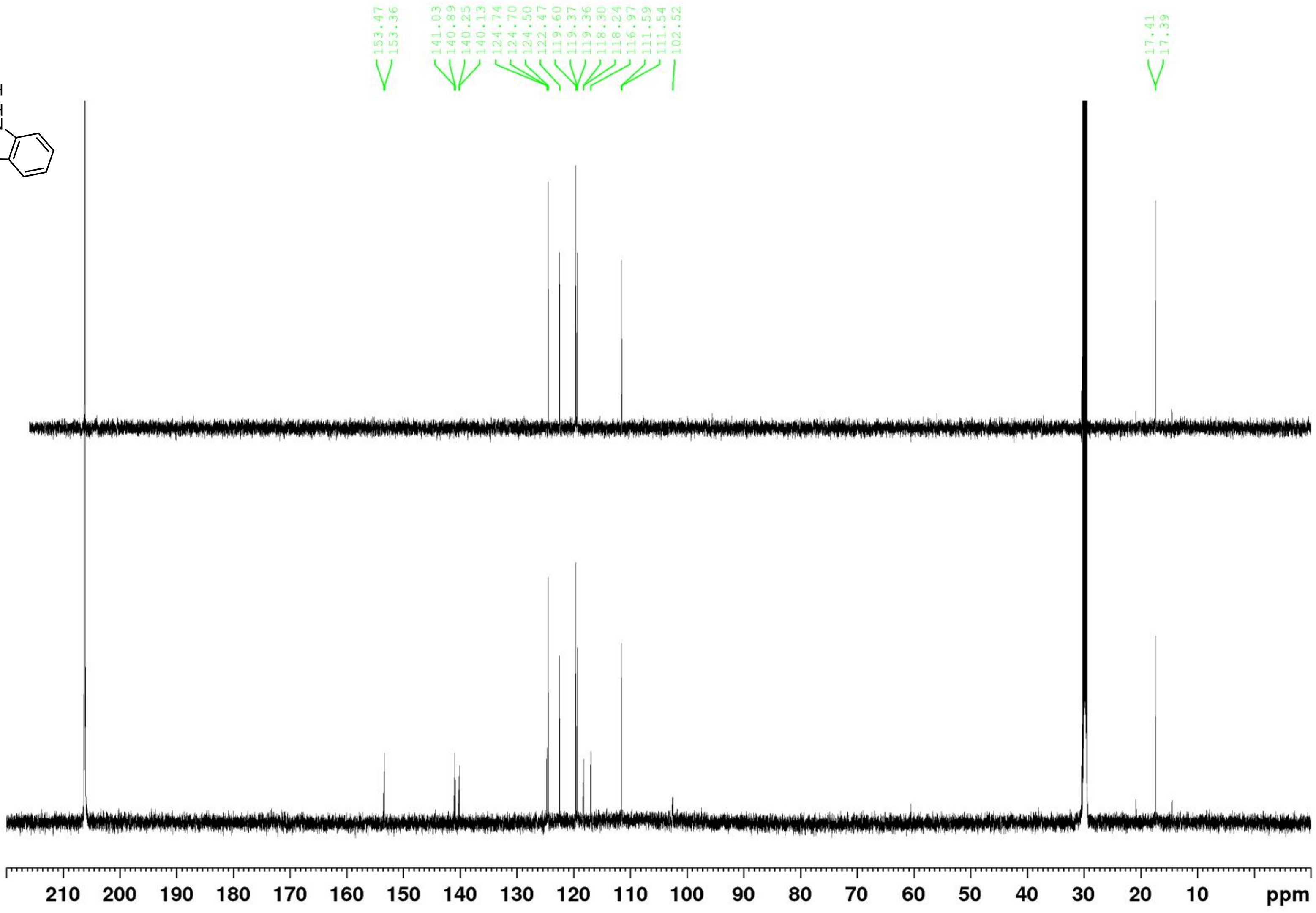

14b ^{1}H NMR, 500 MHz, [D$_6$]DMSO

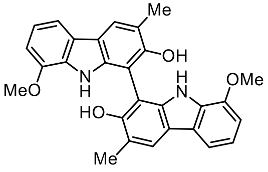

14b

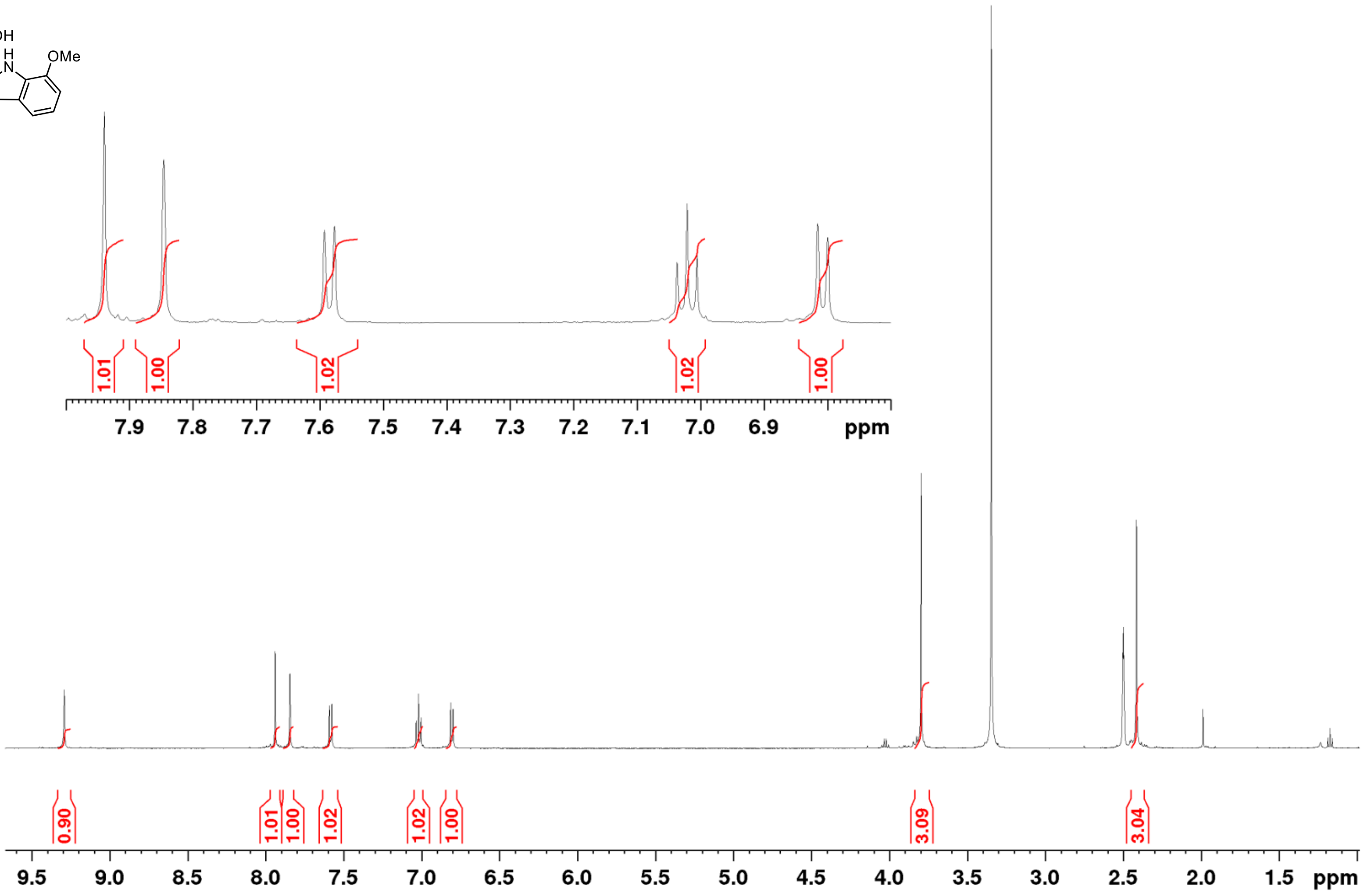

14b ^{13}C NMR, 125 MHz, [D$_6$]DMSO

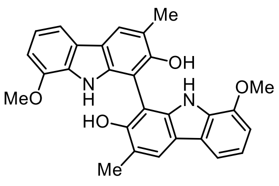

14b

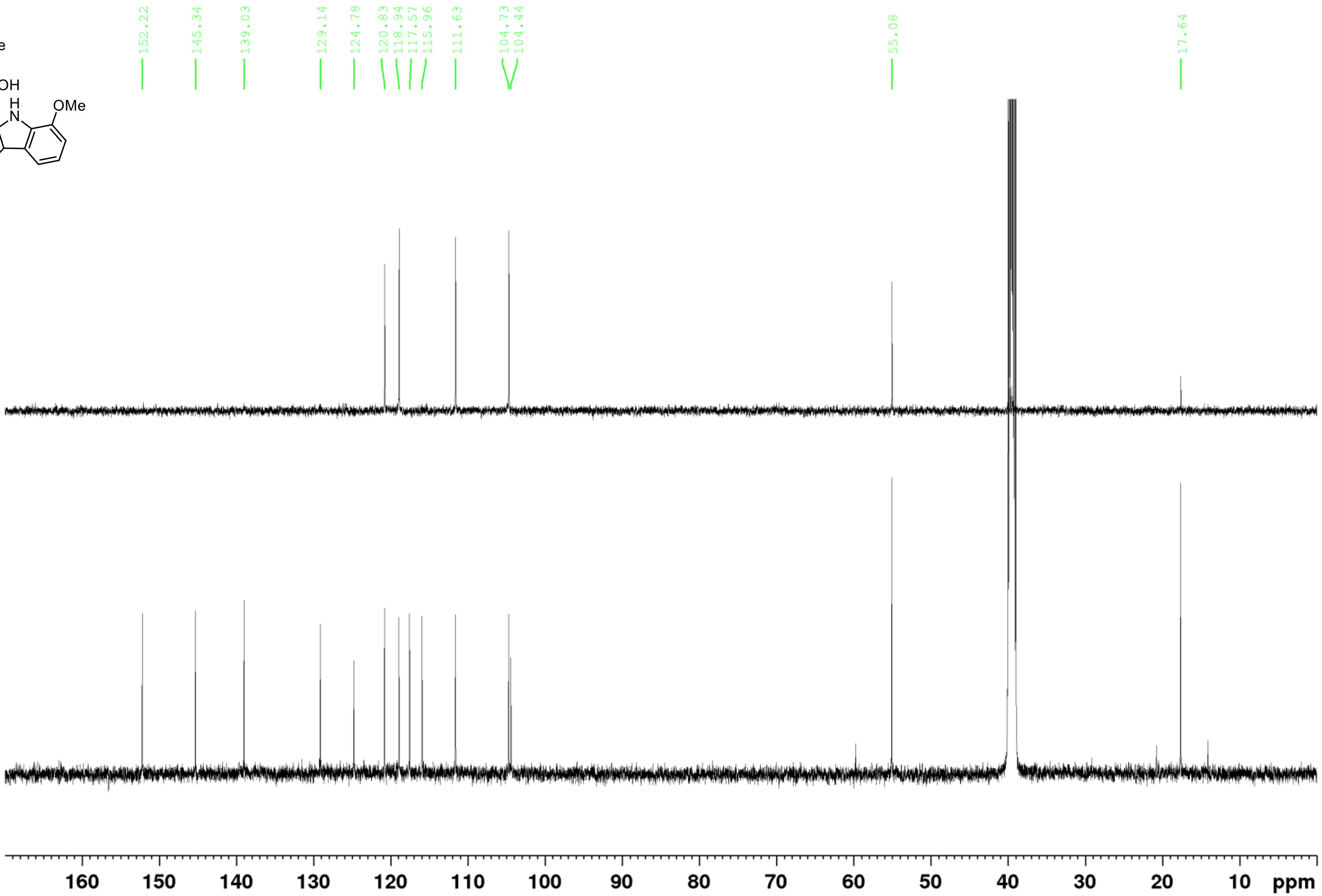

14c ^{1}H NMR, 600 MHz, [D$_6$]acetone

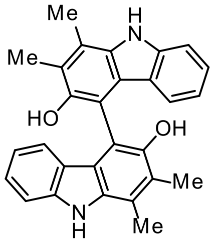

14c

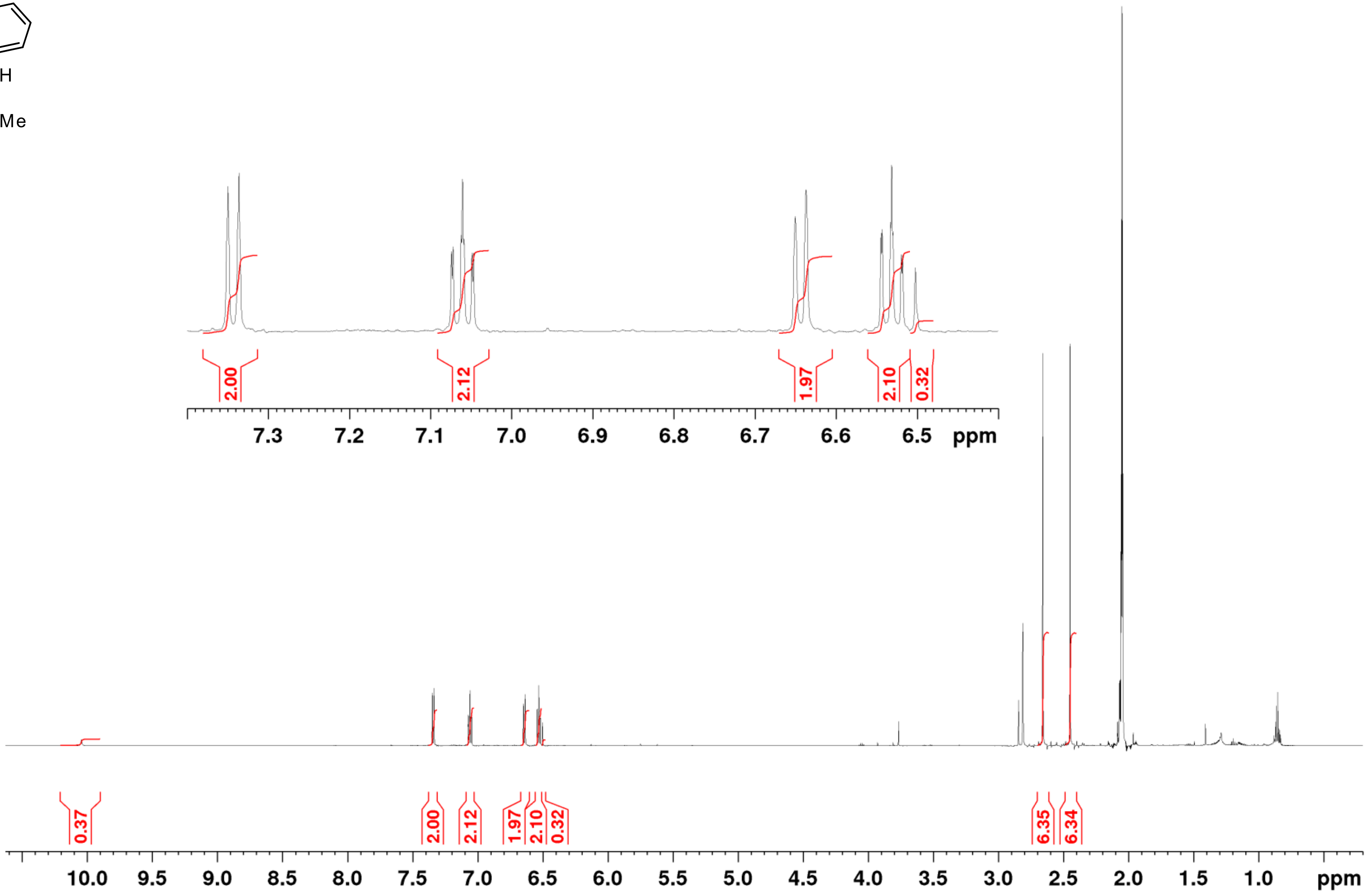

14c ^{13}C NMR, 151 MHz, [D6]acetone

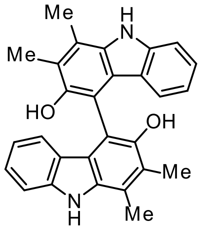

14c

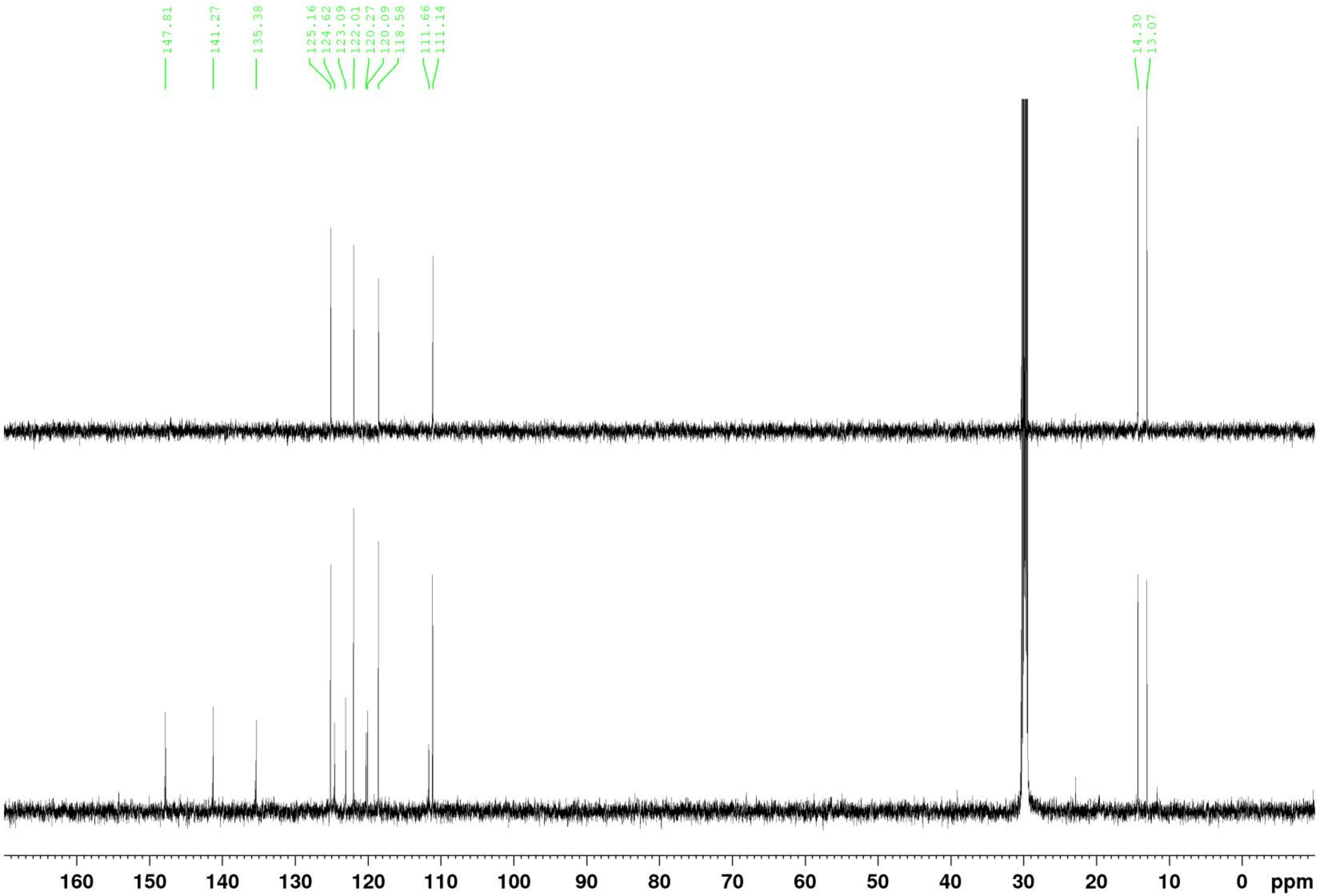

14d ^{1}H NMR, 600 MHz, [D$_6$]acetone

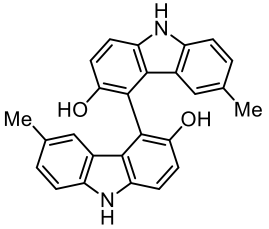

14d

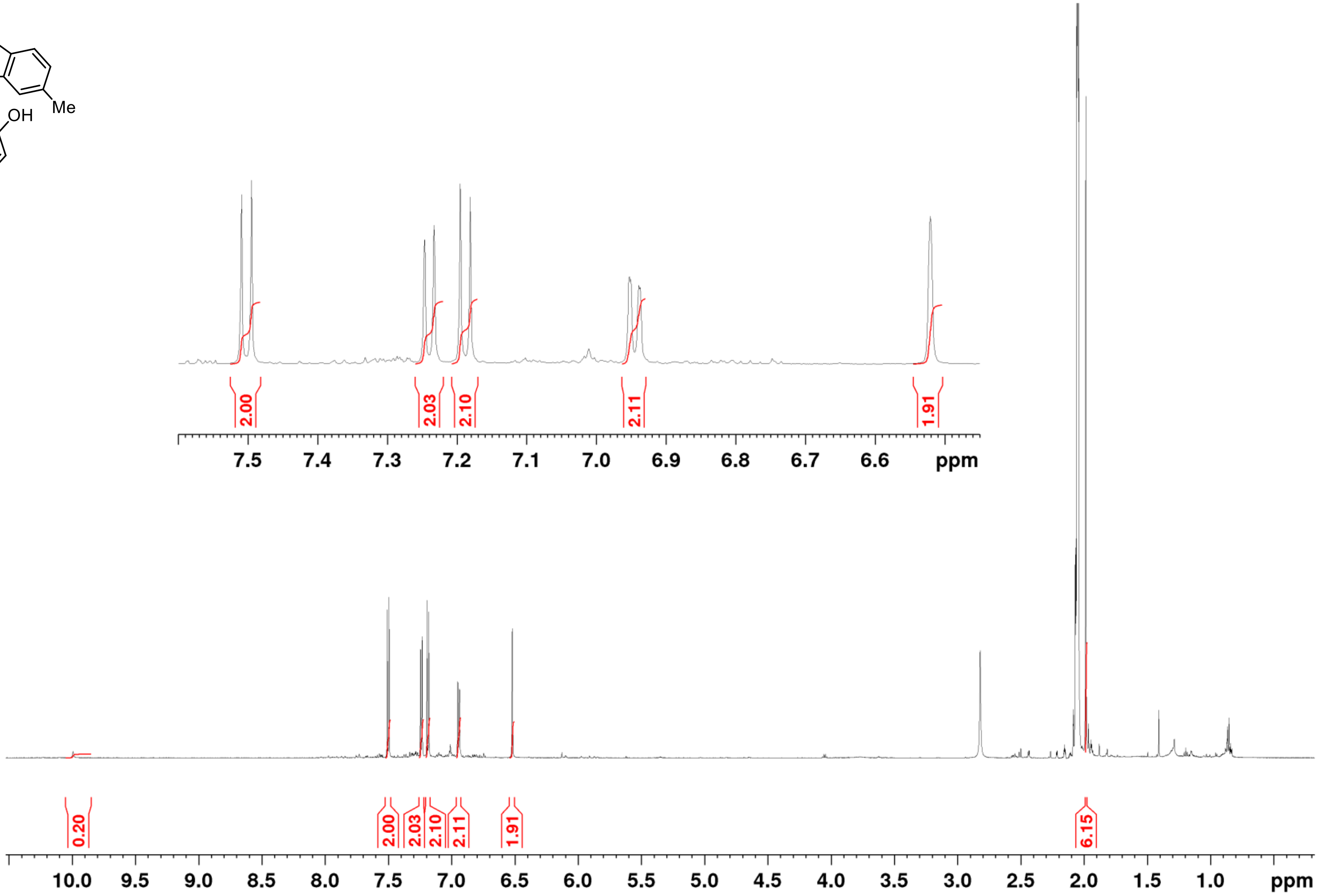

14d ^{13}C NMR, 151 MHz, [D$_6$]acetone

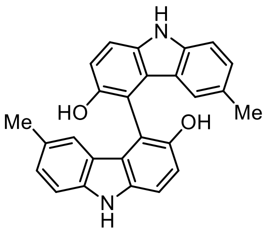

14d

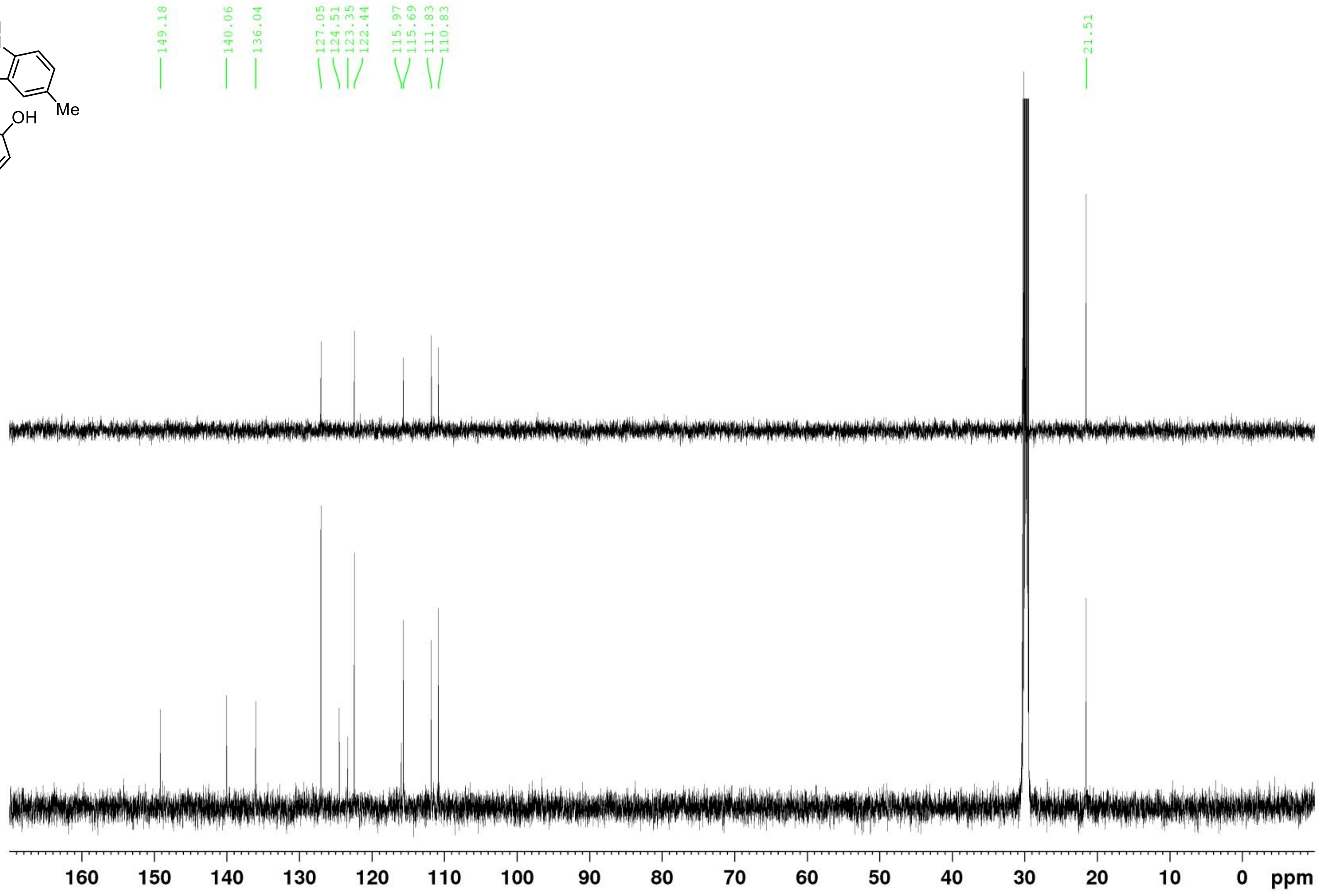

15a ^{1}H NMR, 500 MHz, $CDCl_3$

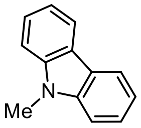

15a

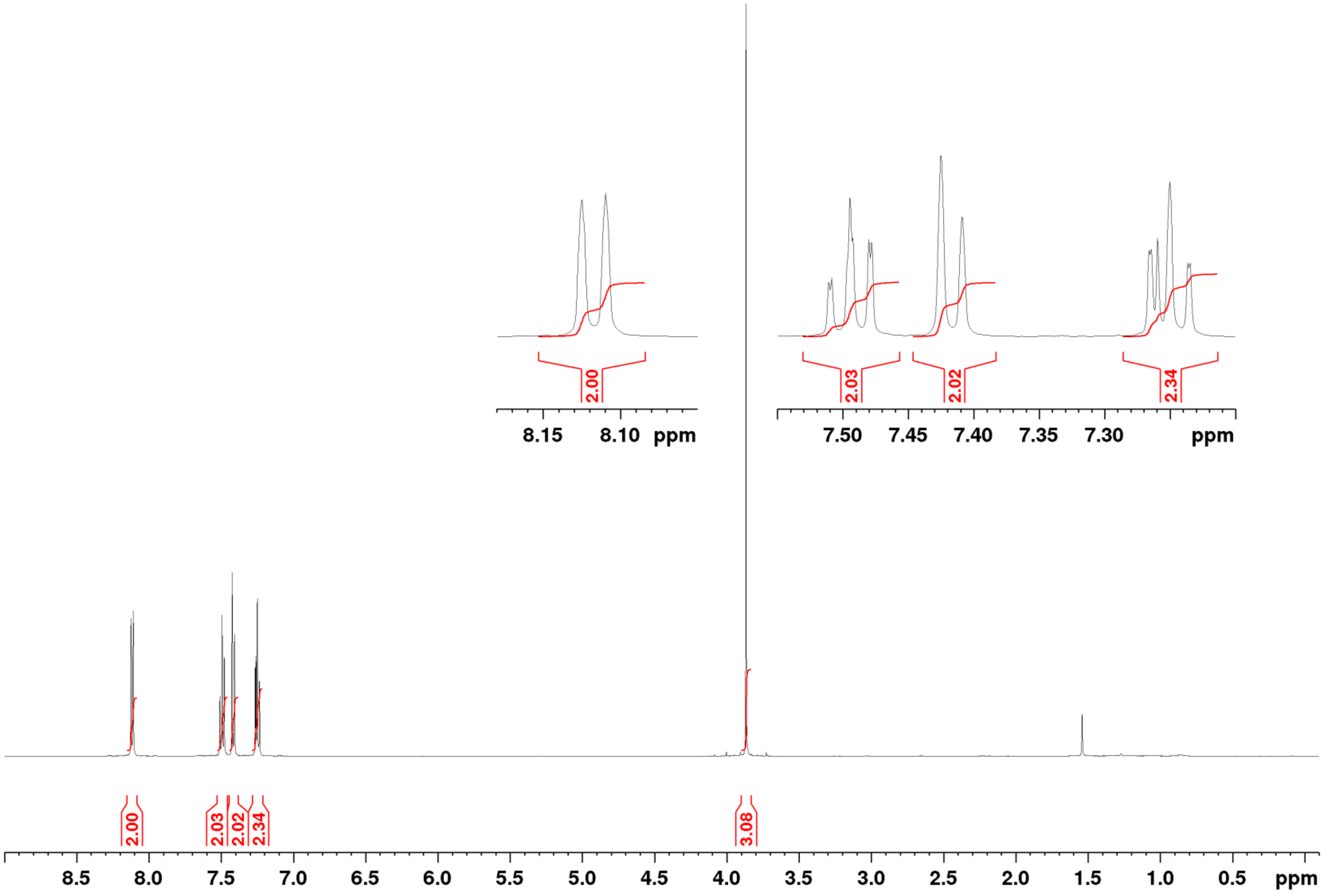

15a ^{13}C NMR, 125 MHz, $CDCl_3$

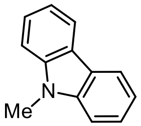

15a

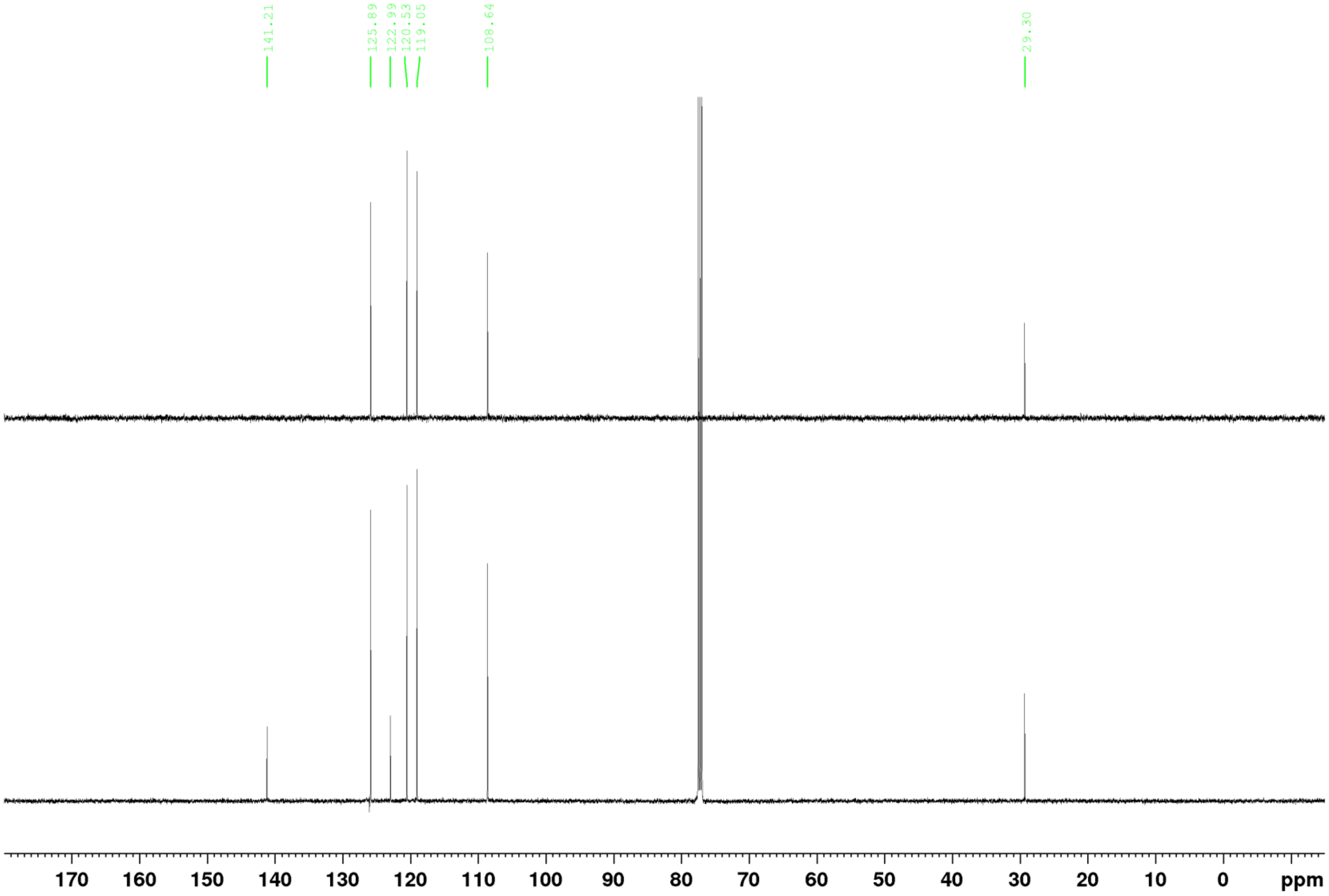

16a ^{1}H NMR, 600 MHz, [D$_6$]DMSO (traces of CH_2Cl_2)

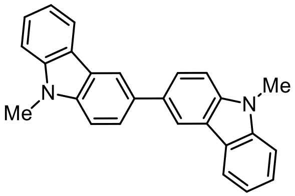

16a

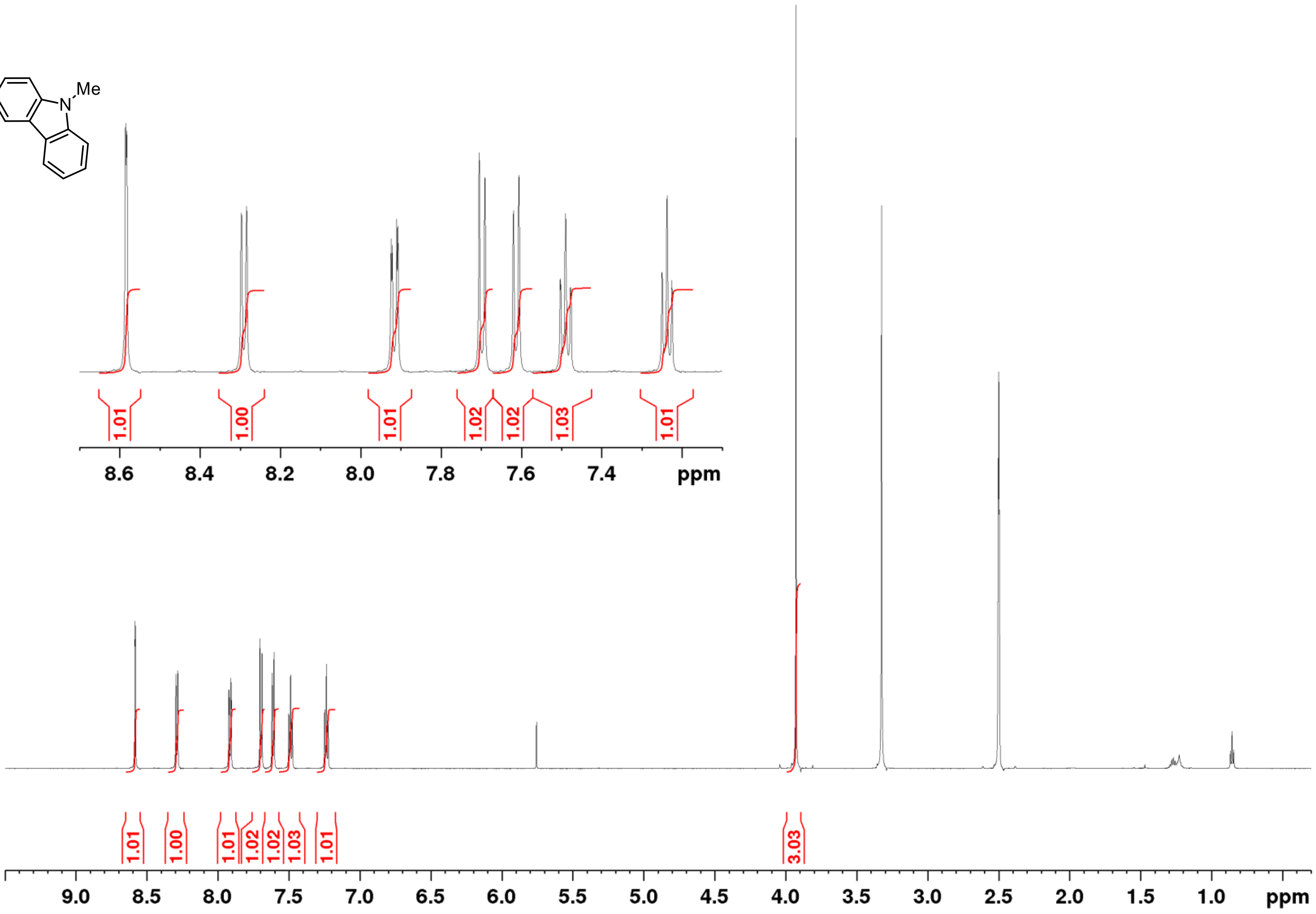

16a ^{13}C NMR, 151 MHz, [D6]DMSO

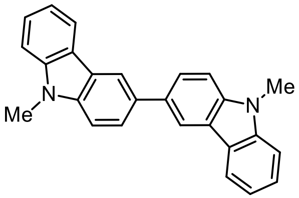

16a

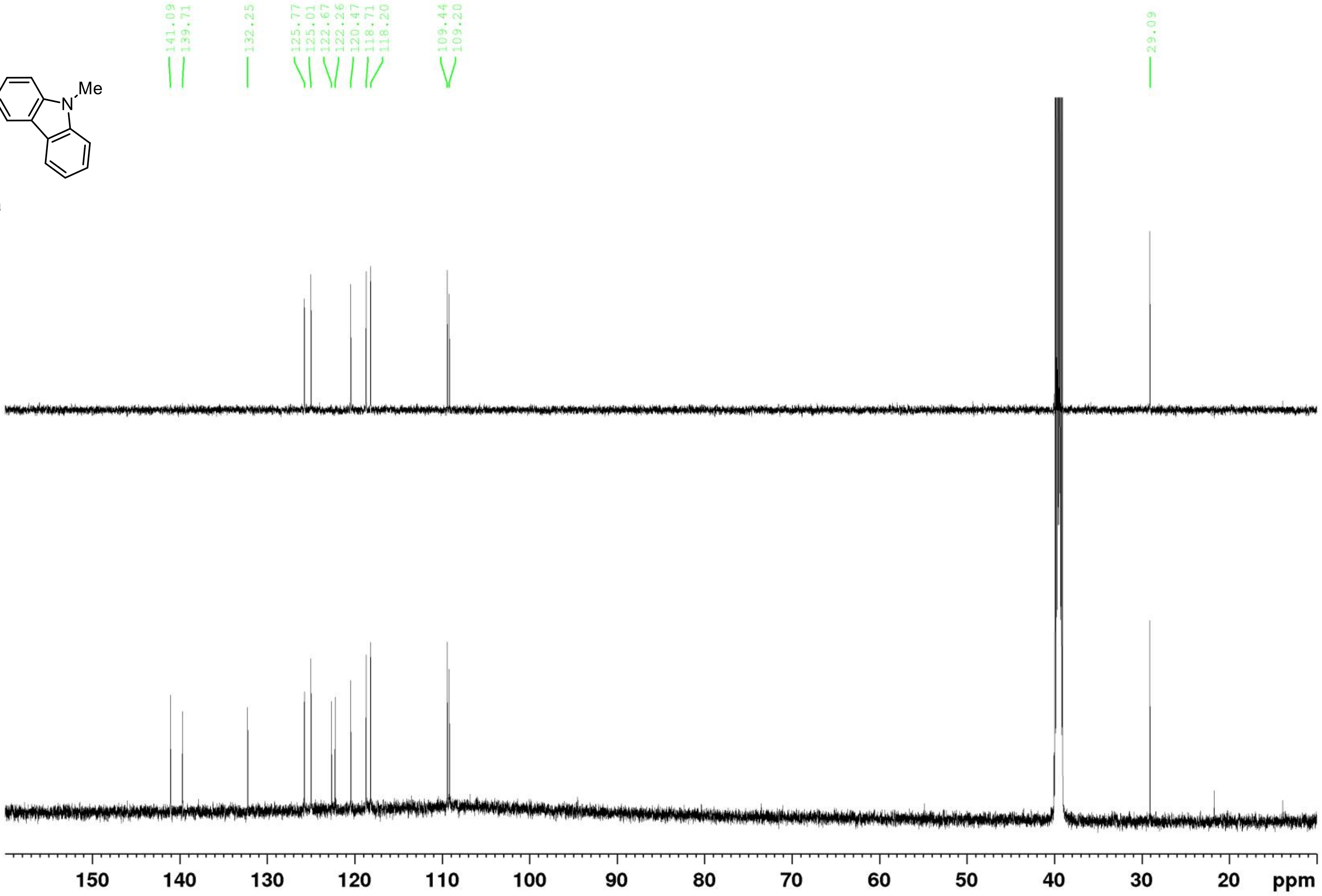

15b ^{1}H NMR, 600 MHz, $CDCl_3$

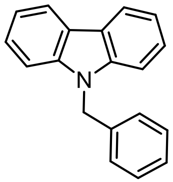
15b

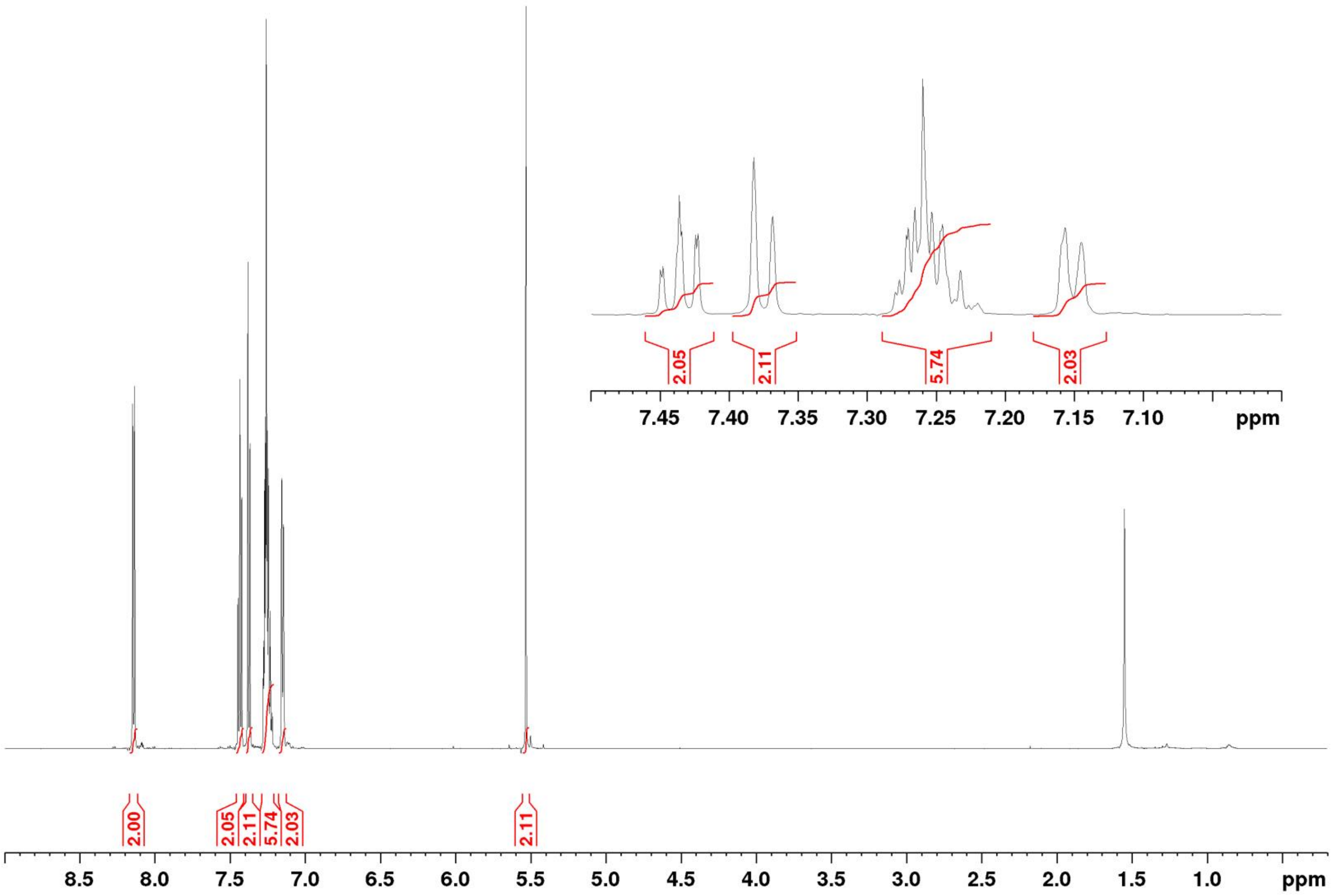

15b ^{13}C NMR, 151 MHz, $CDCl_3$

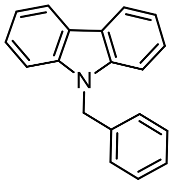

15b

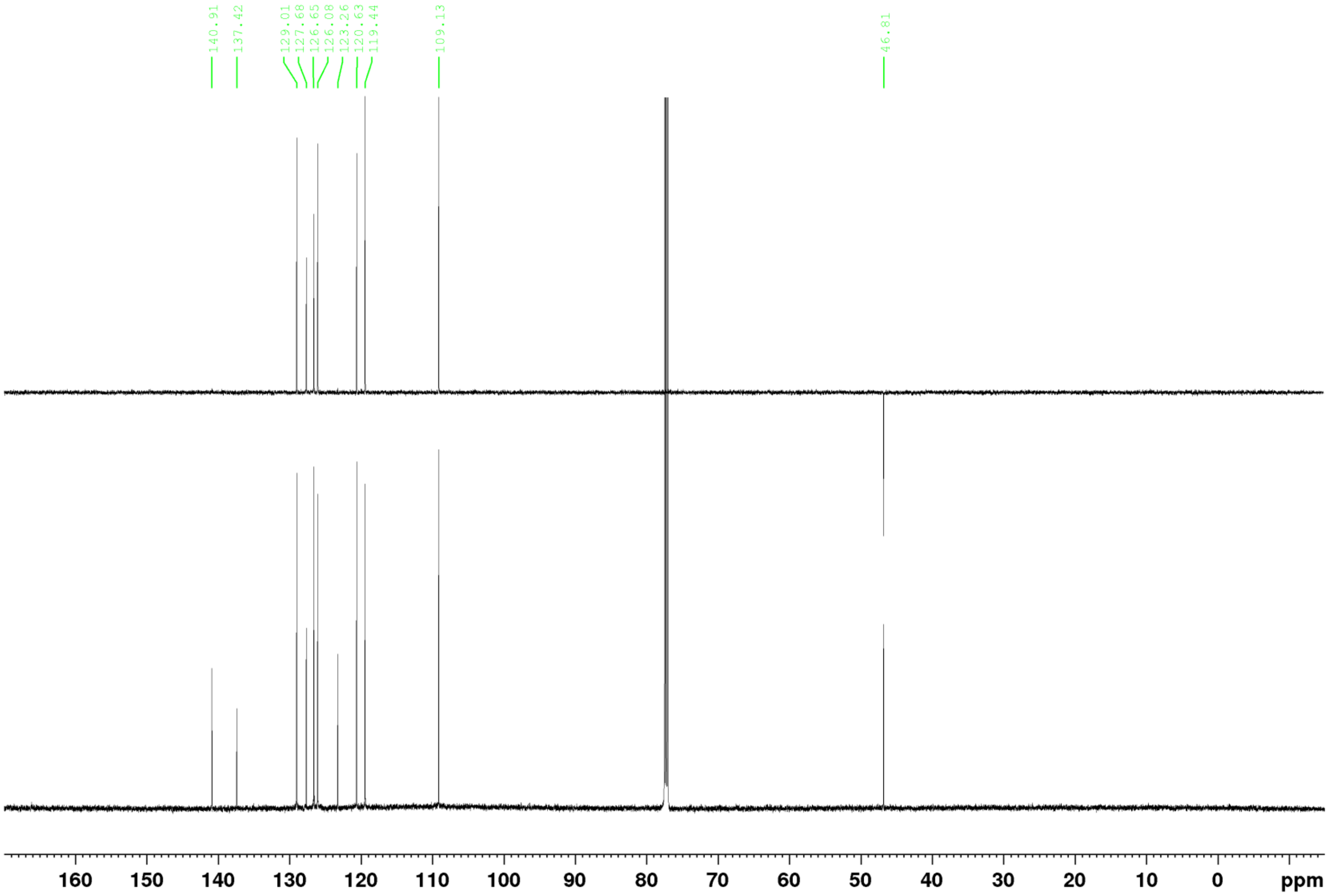

16b ^{1}H NMR, 600 MHz, [D$_6$]DMSO

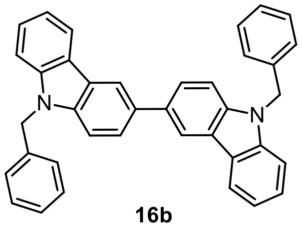

16b

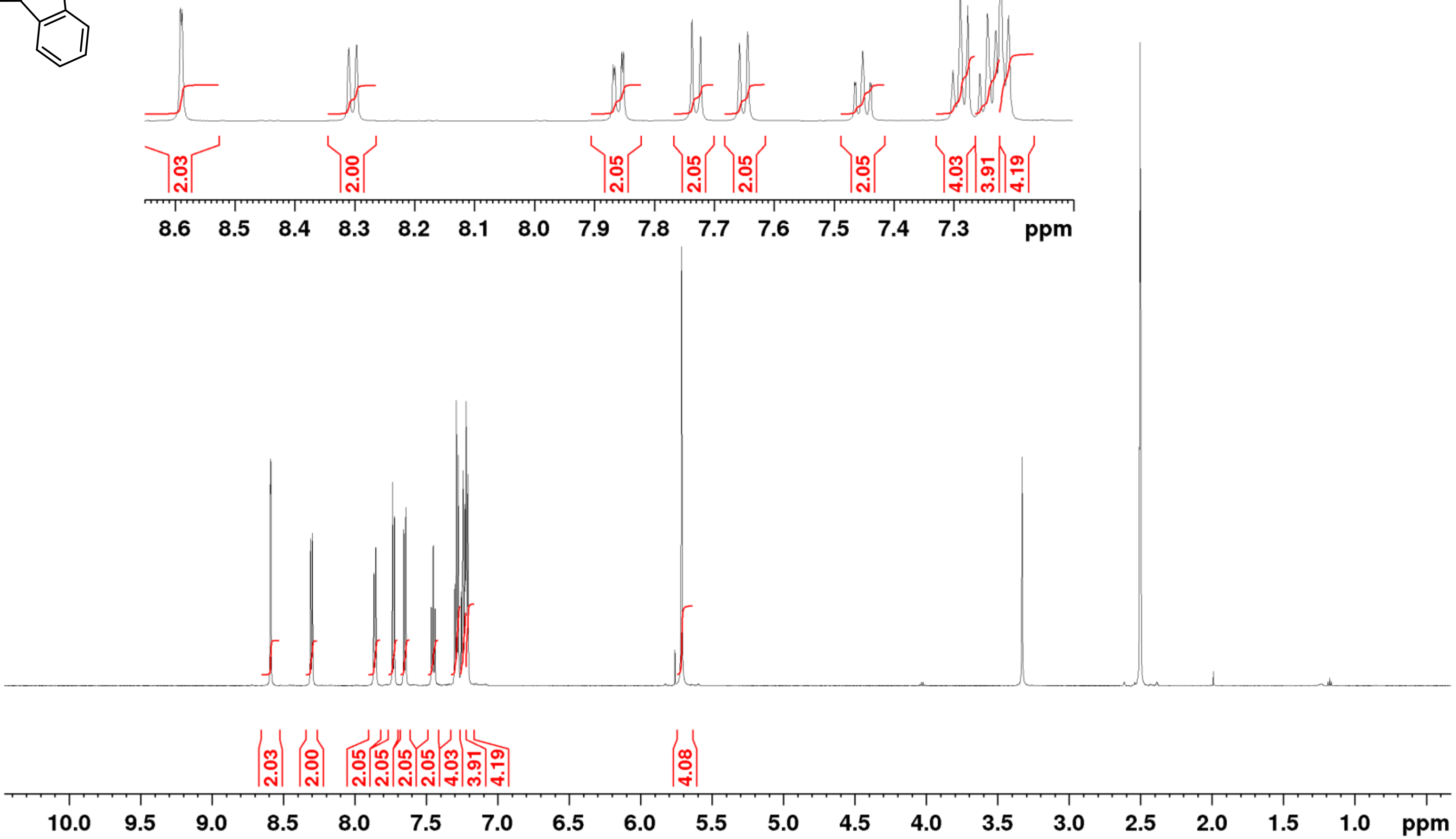

16b ^{13}C NMR, 151 MHz, [D$_6$]DMSO

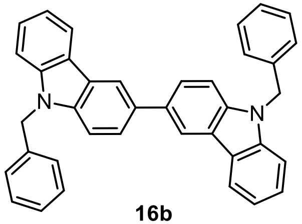

16b

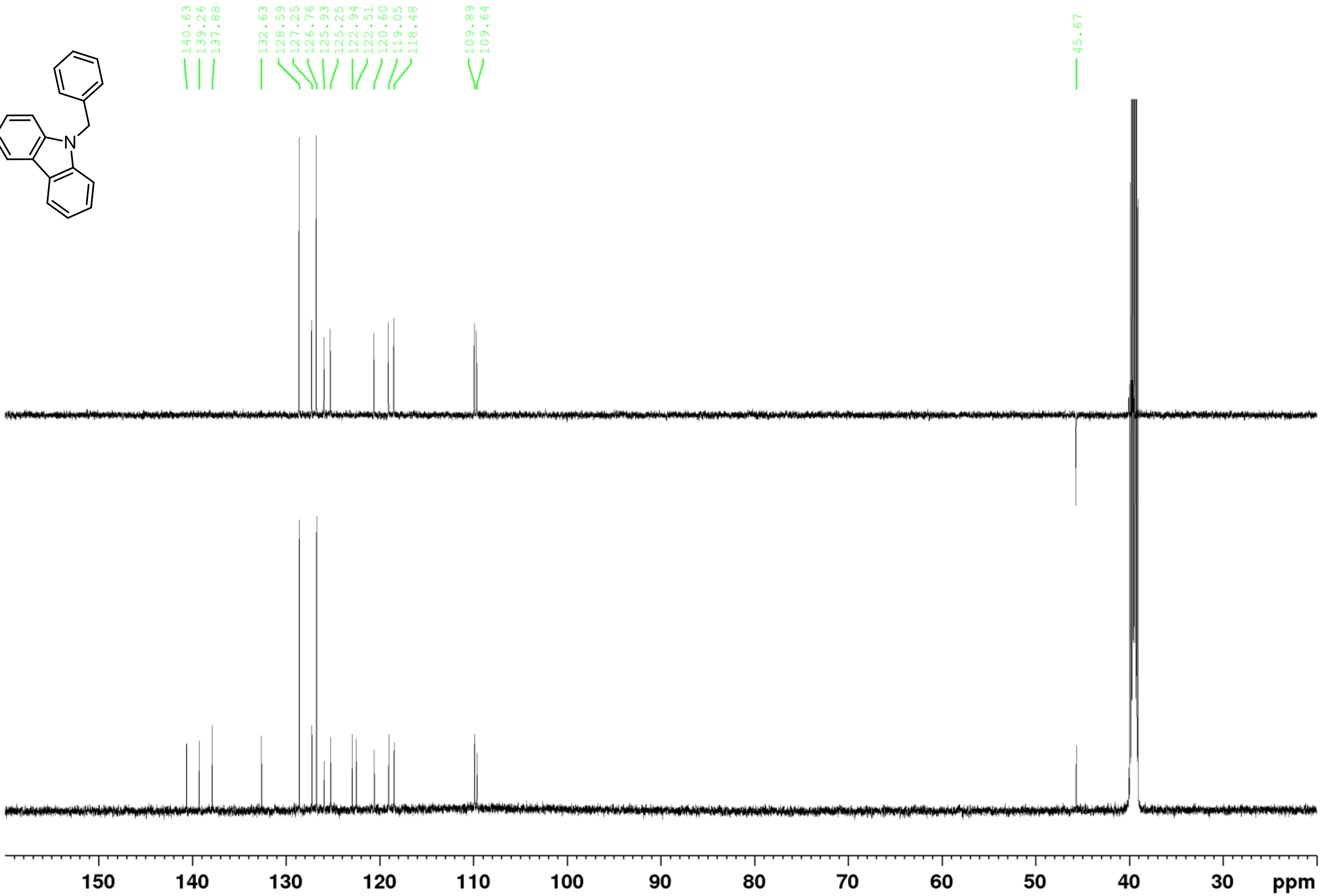

16c ^{1}H NMR, 600 MHz, [D$_6$]DMSO

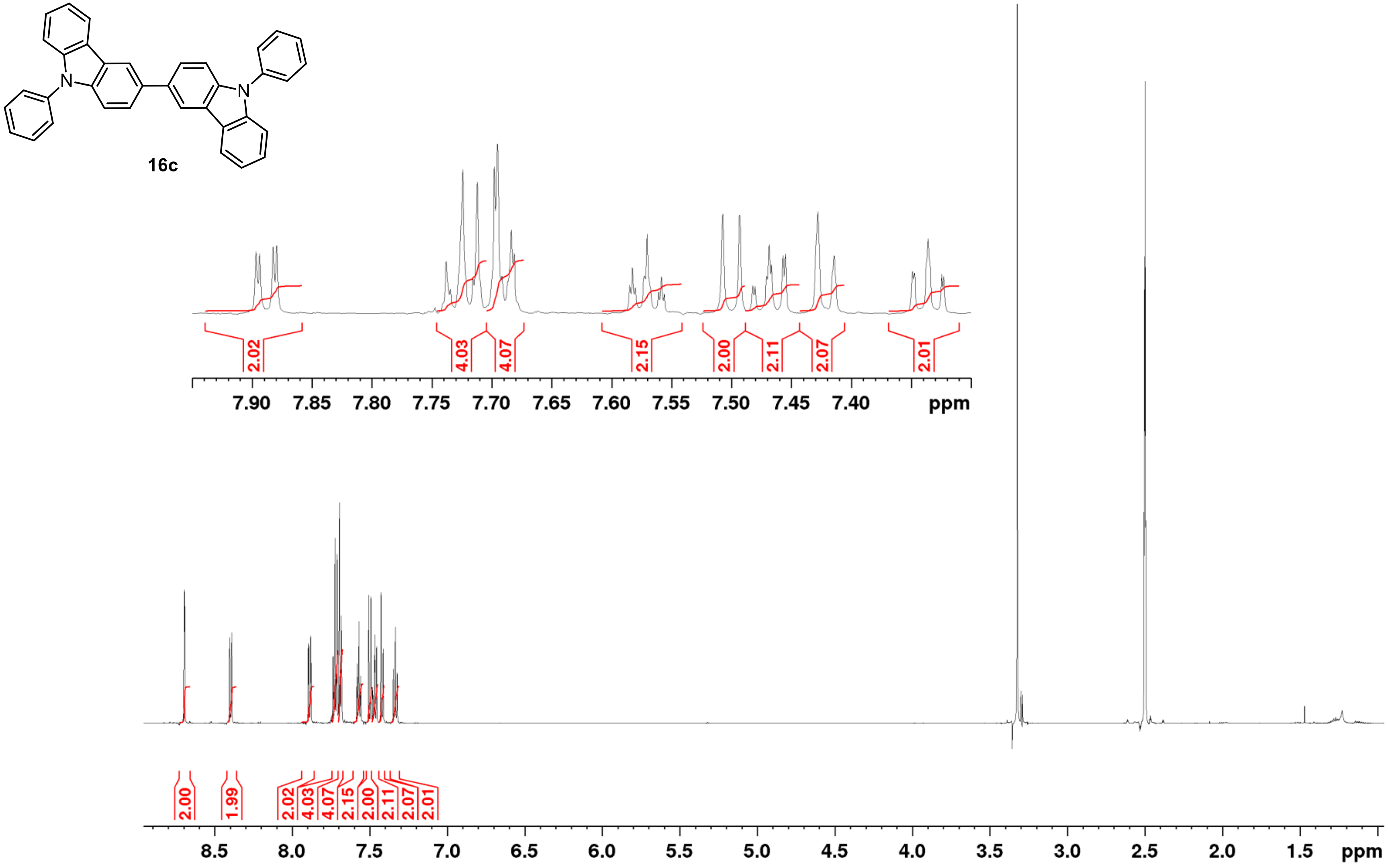

16c ^{13}C NMR, 151 MHz, [D6]DMSO

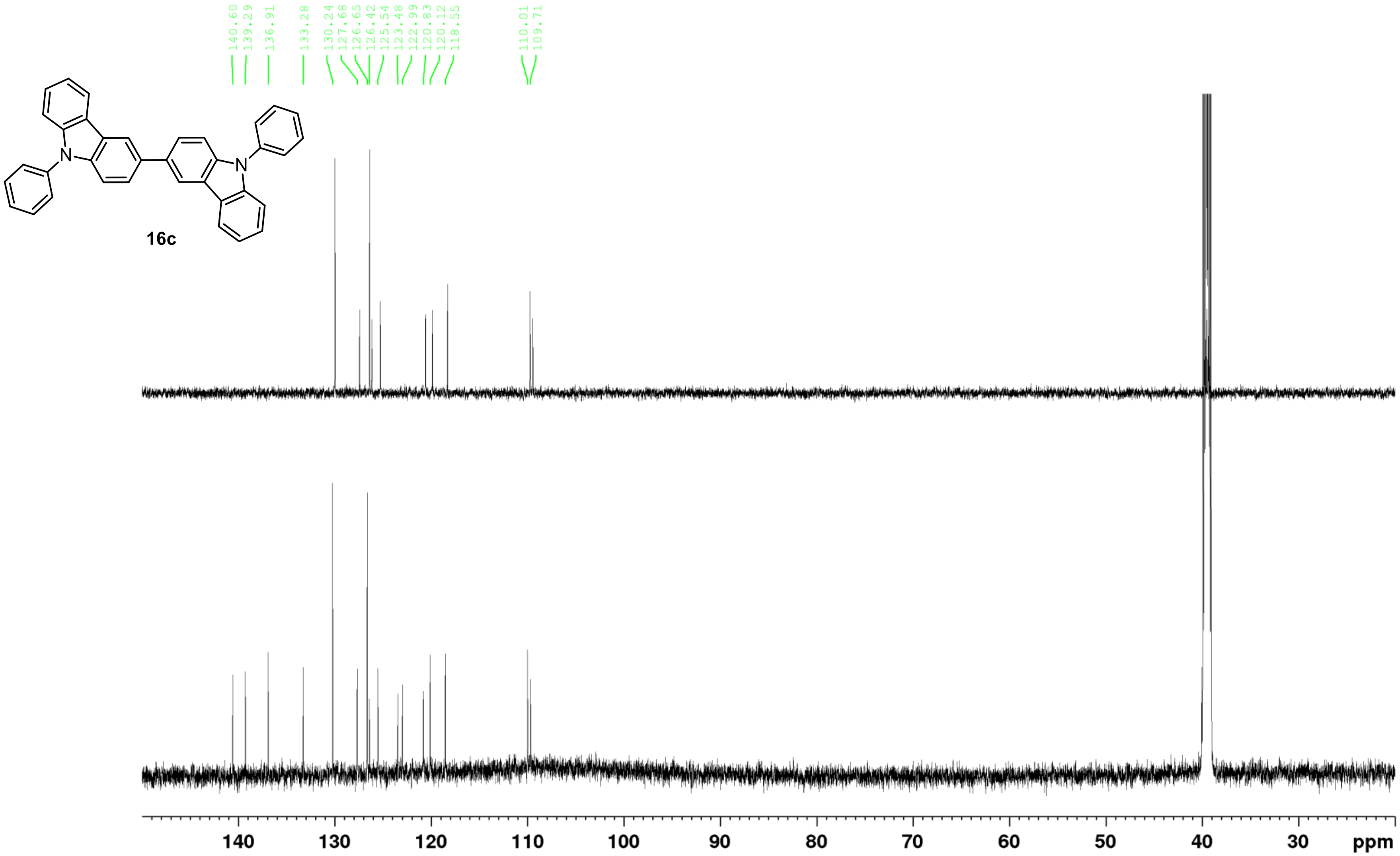

18a ^{1}H NMR, 500 MHz, $CDCl_3$

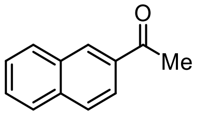

18a

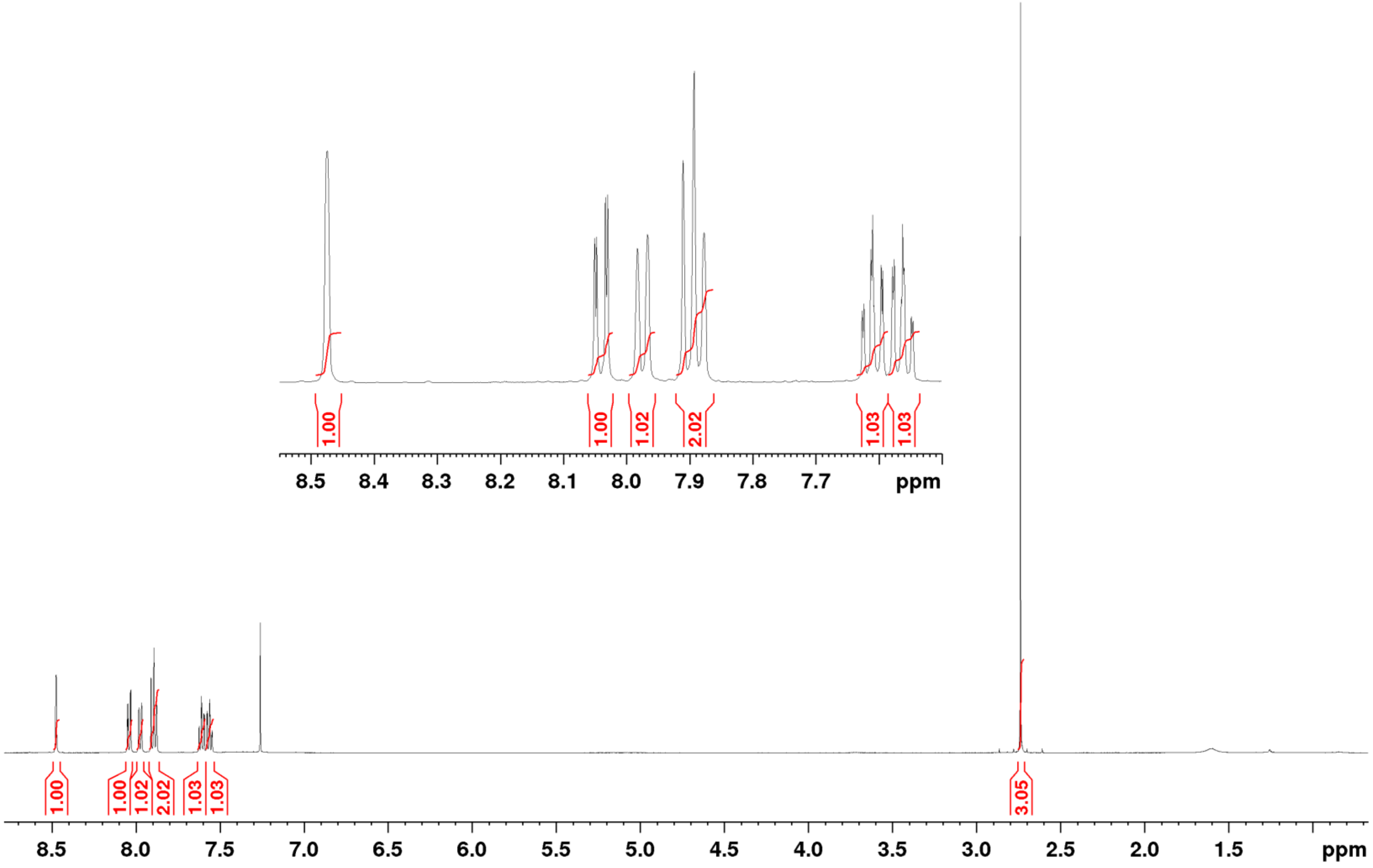

18a ^{13}C NMR, 125 MHz, $CDCl_3$

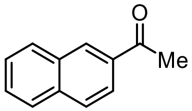

18a

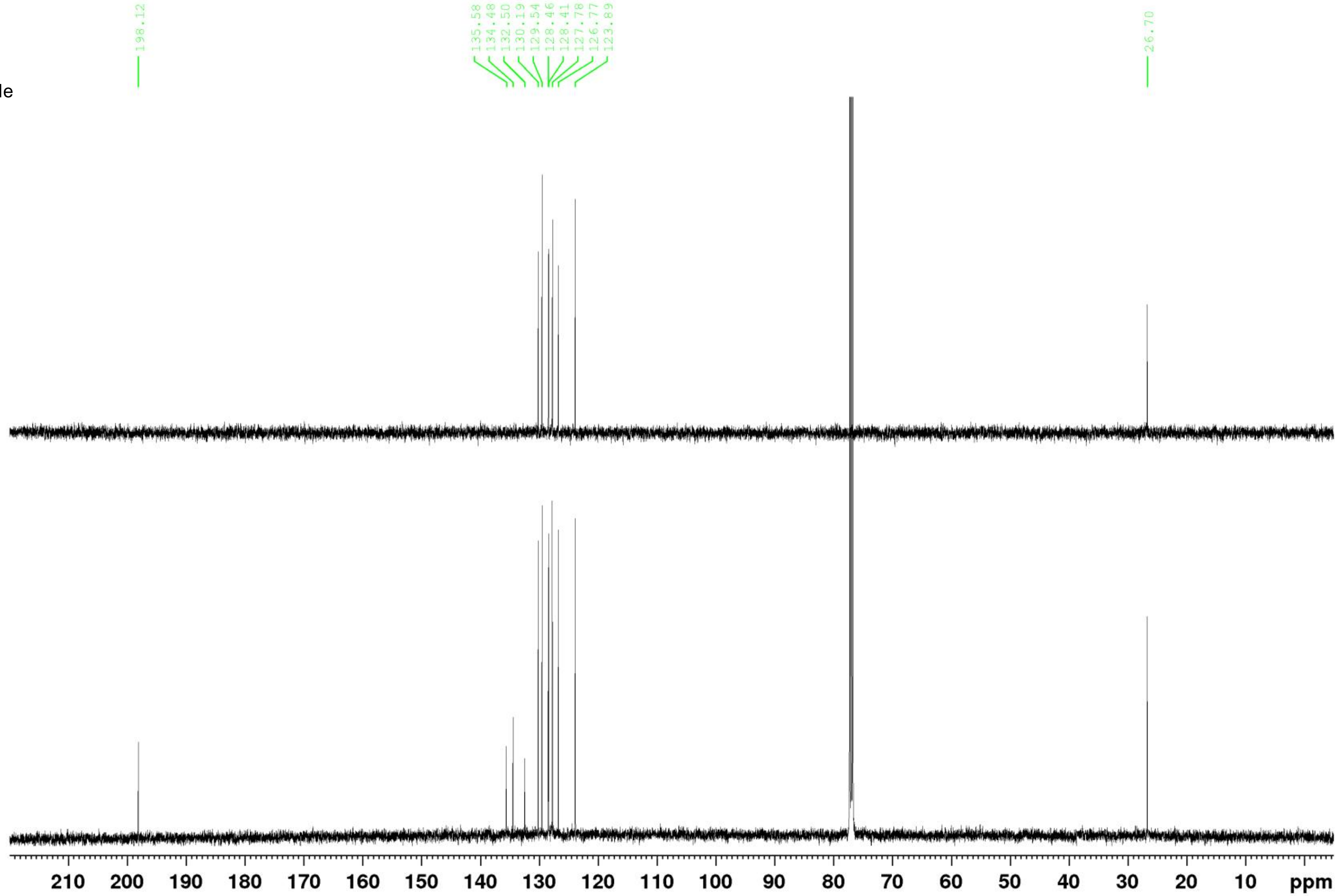

19a ^{1}H NMR, 600 MHz, $CDCl_3$

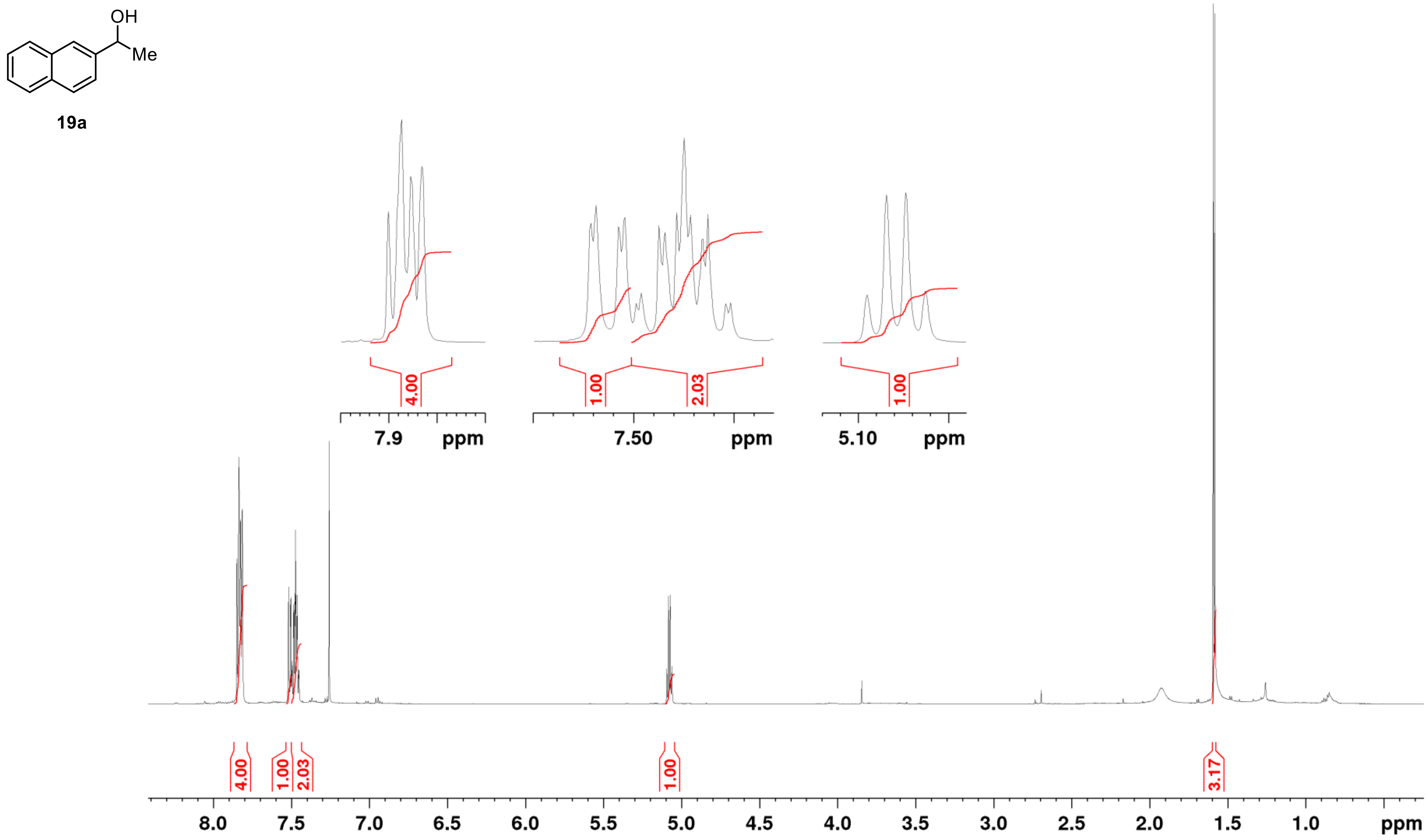

19a ^{13}C NMR, 151 MHz, $CDCl_3$

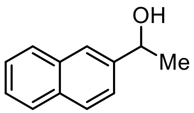

19a

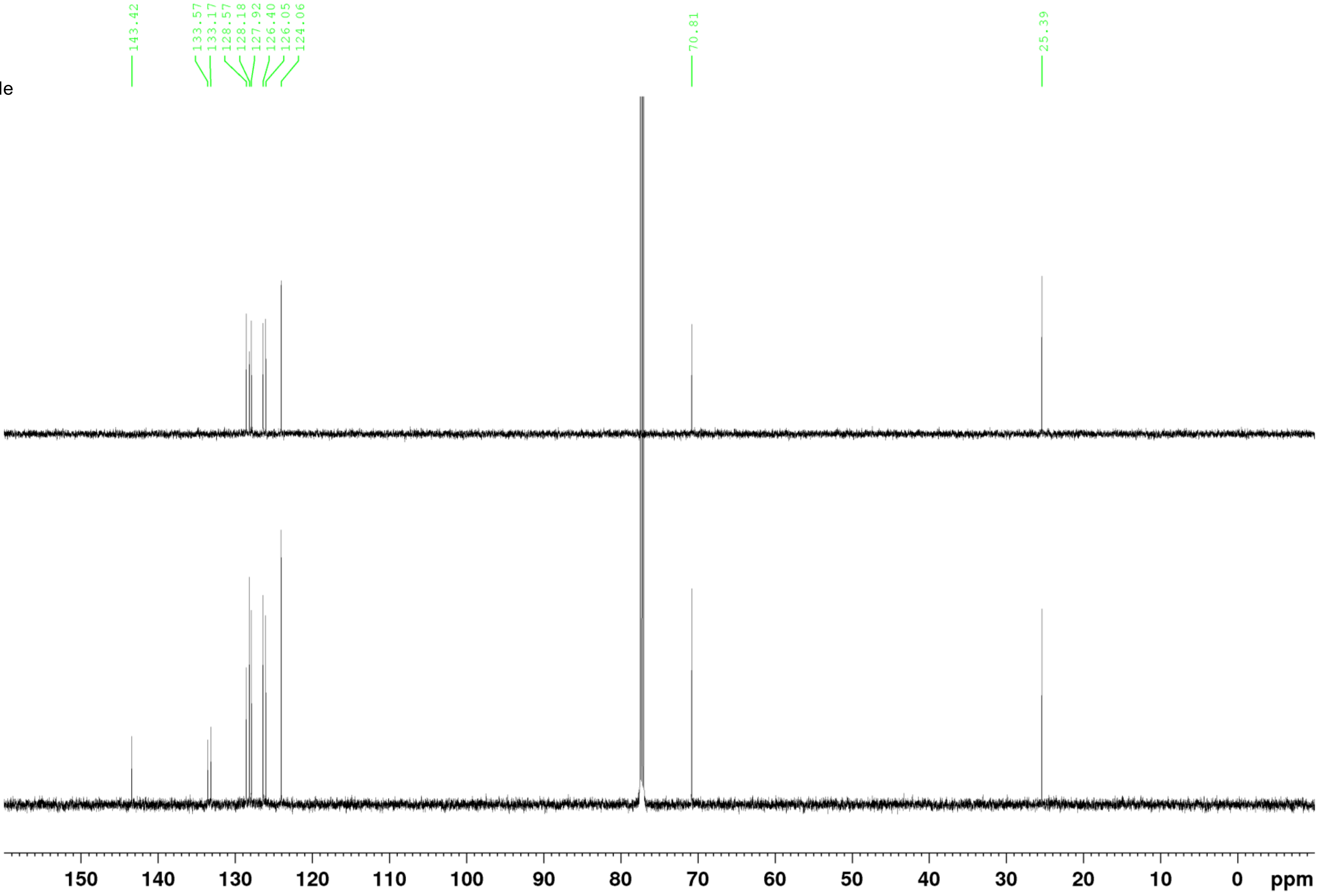

18b ^{1}H NMR, 600 MHz, $CDCl_3$

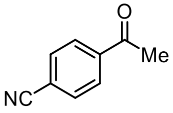

18b

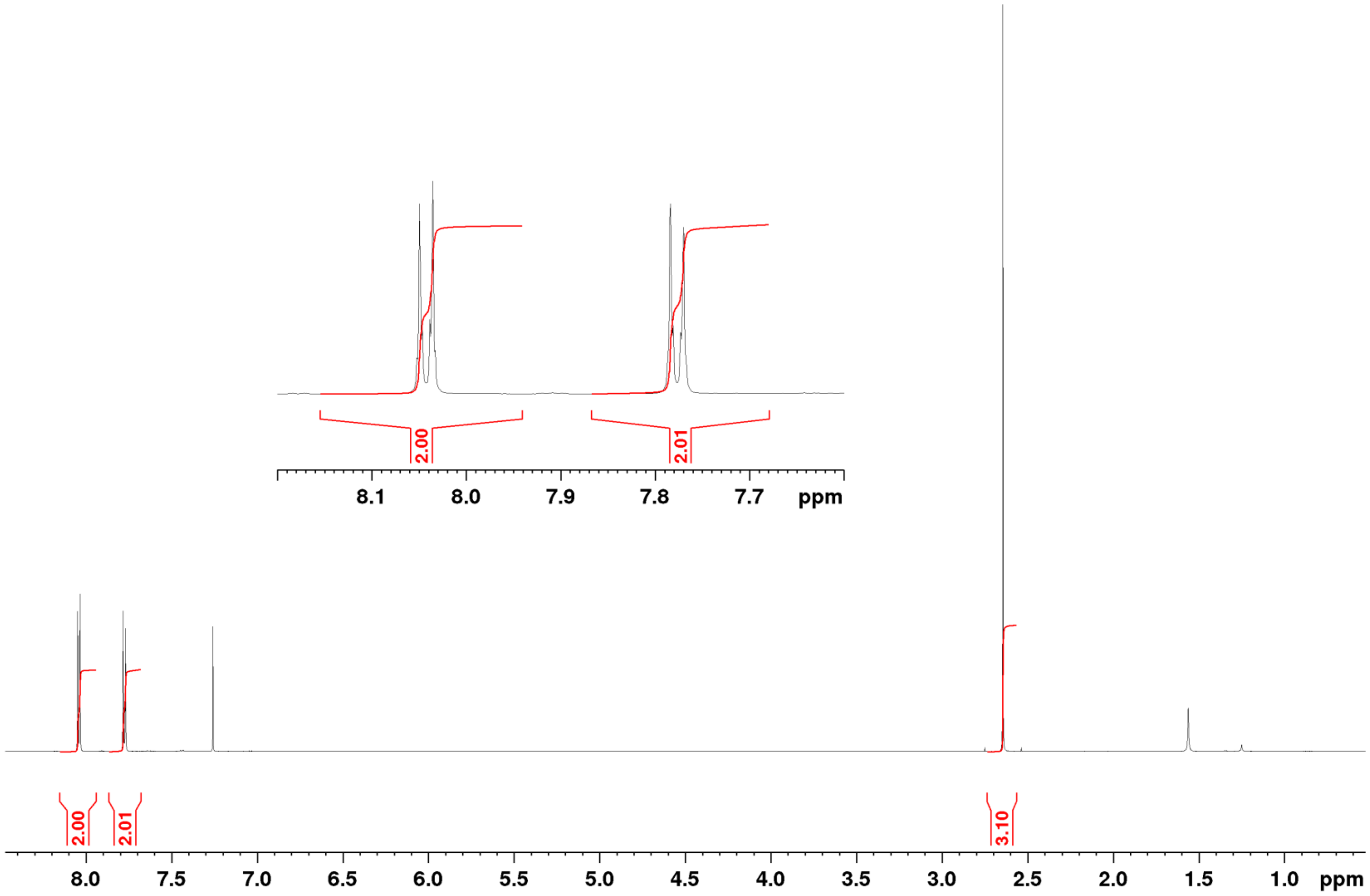

18b ^{13}C NMR, 151 MHz, $CDCl_3$

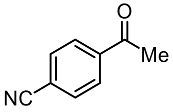

18b

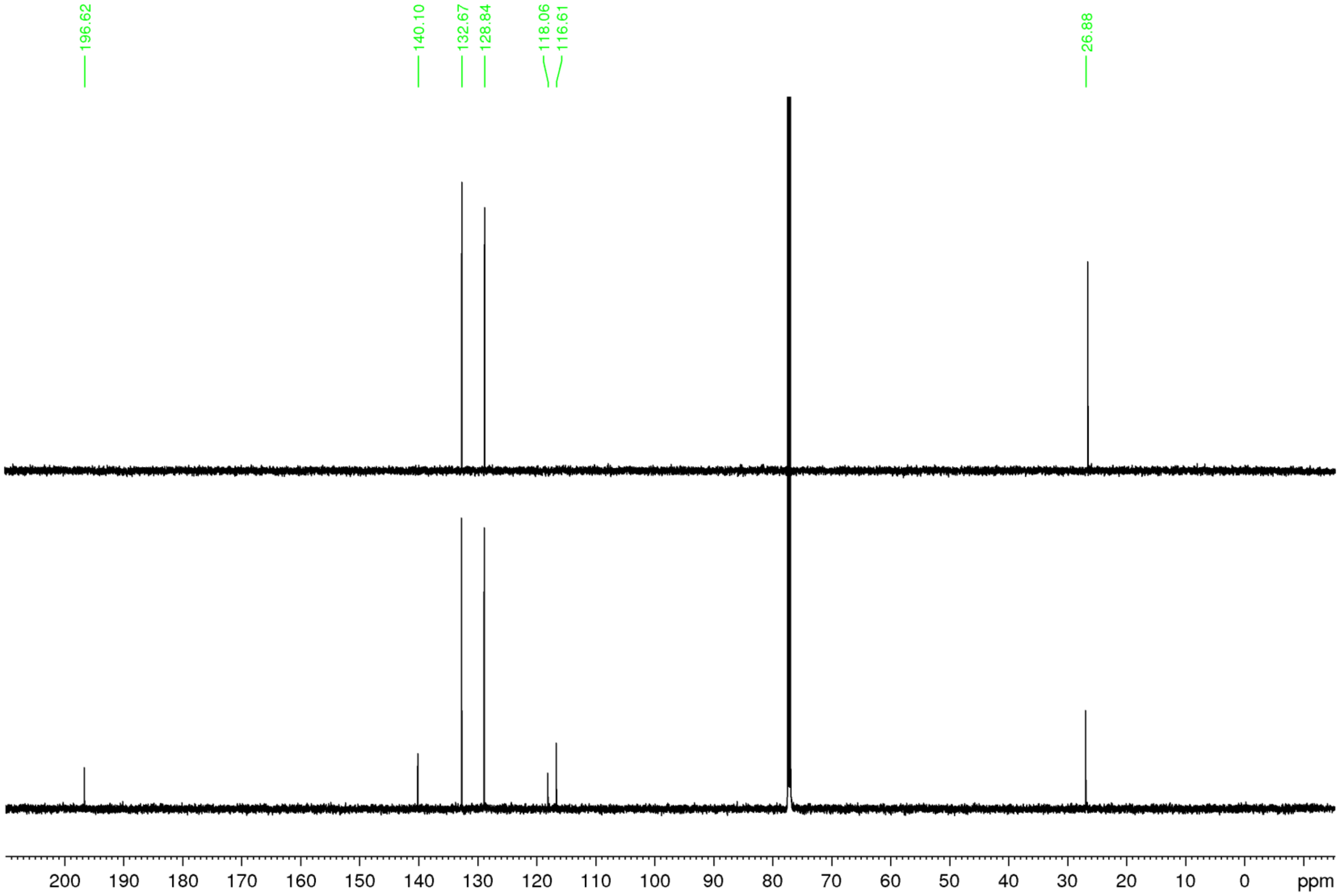

18c ^{1}H NMR, 500 MHz, $CDCl_3$

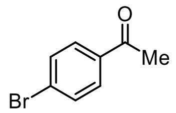

18c

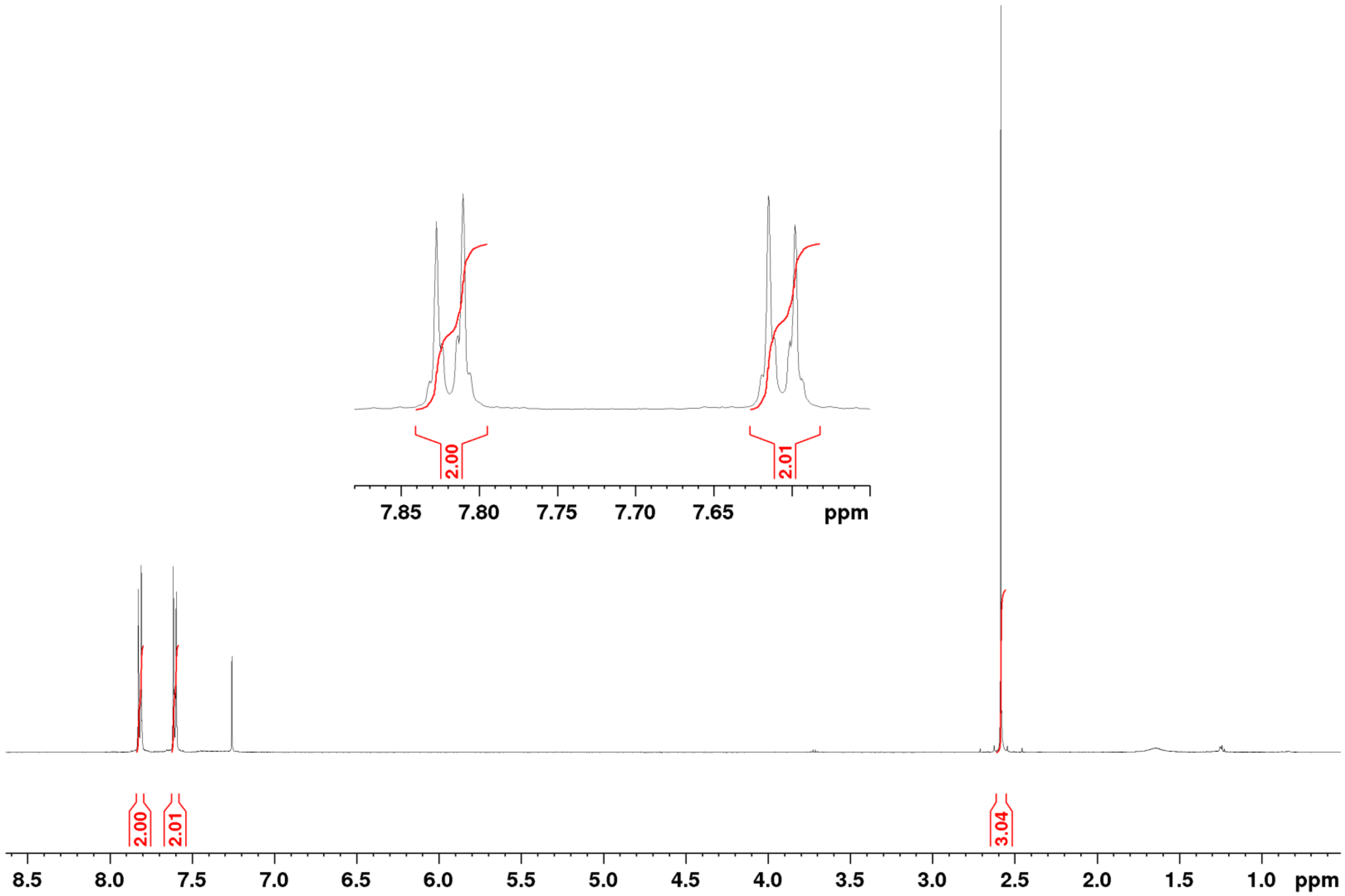

18c ^{13}C NMR,

125 MHz, $CDCl_3$

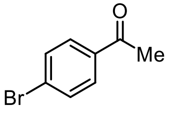

18c

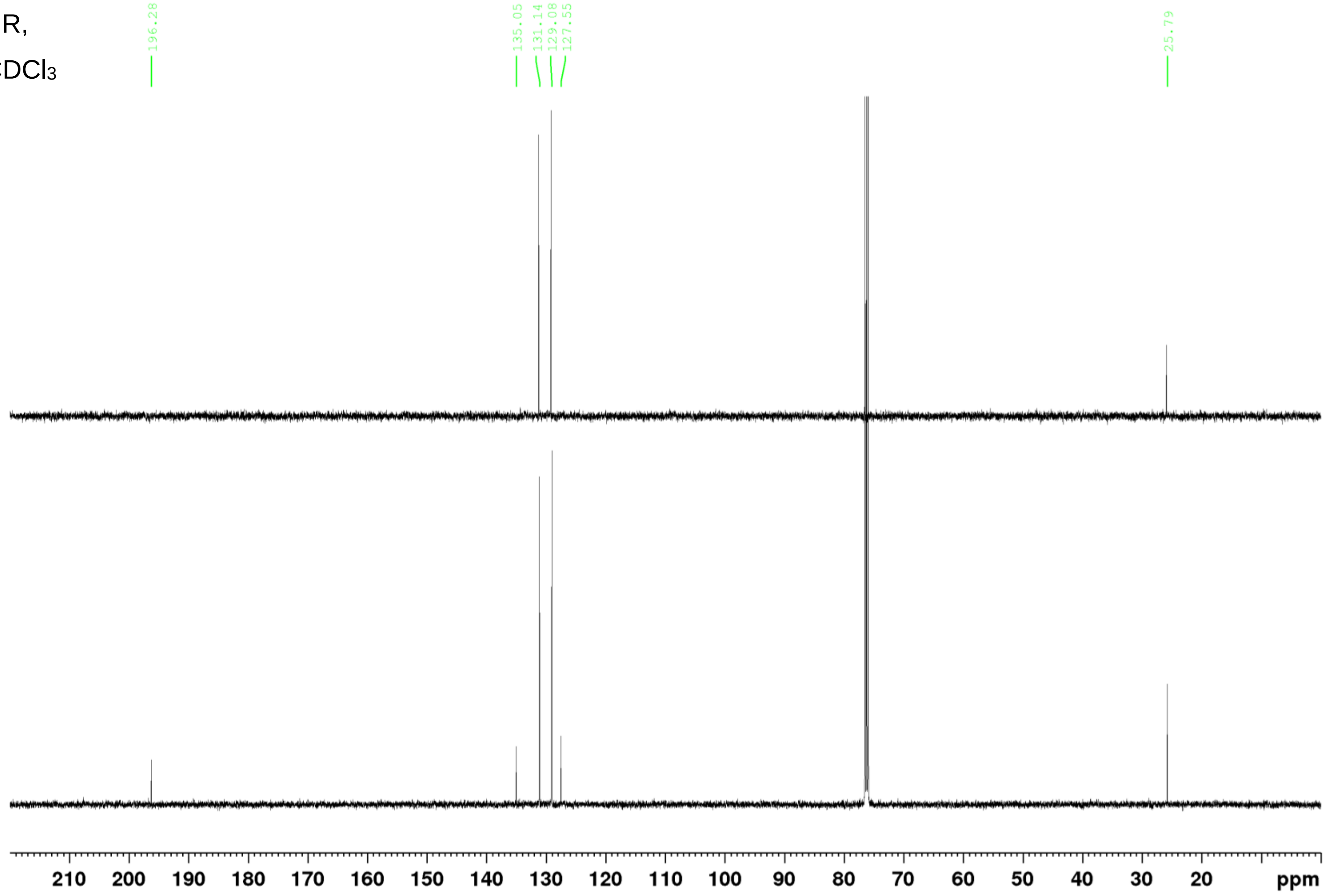

18d ^{1}H NMR, 600 MHz, $CDCl_3$

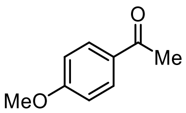

18d

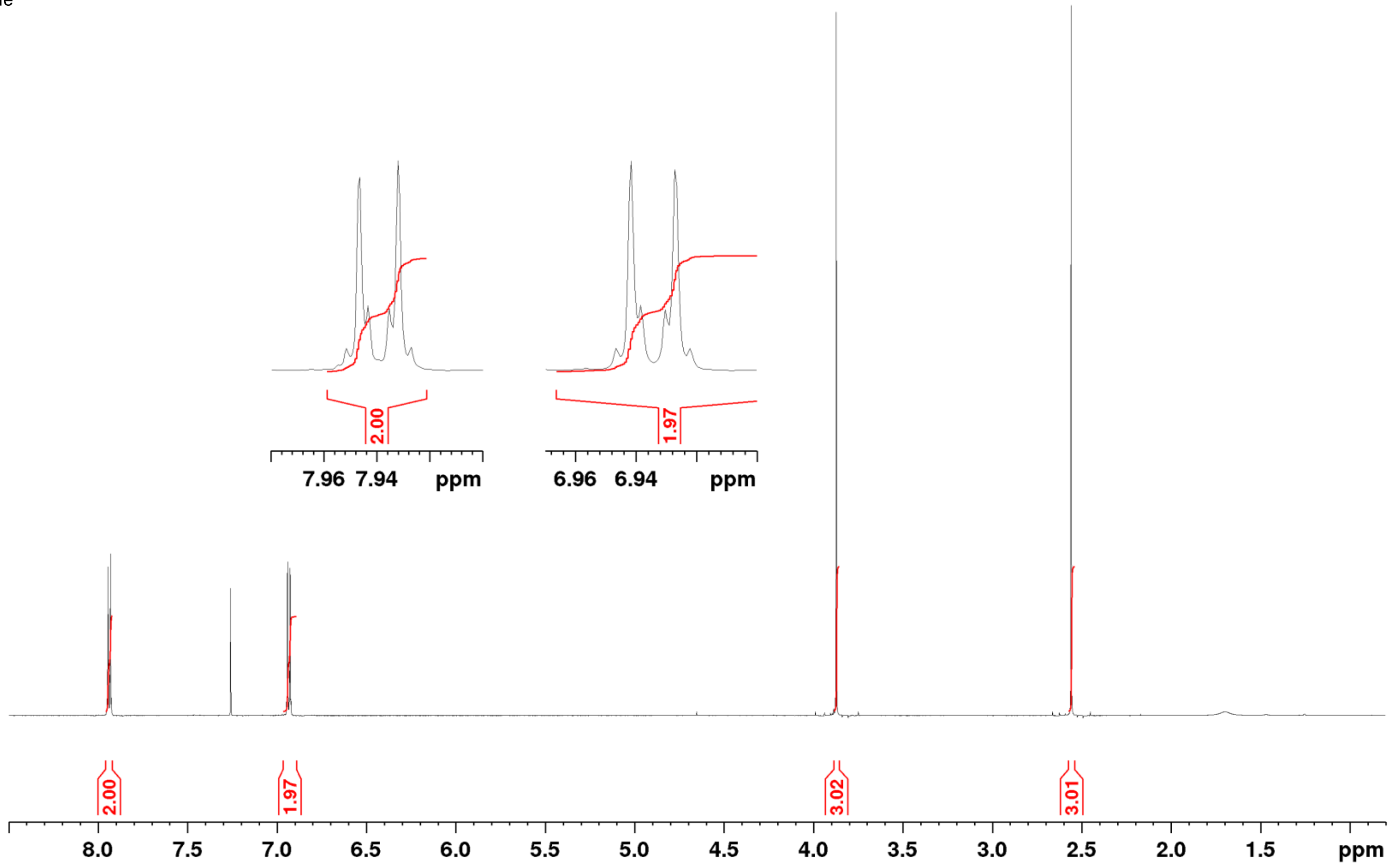

18d ^{13}C NMR, 151 MHz, $CDCl_3$

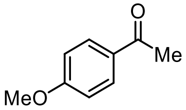

18d

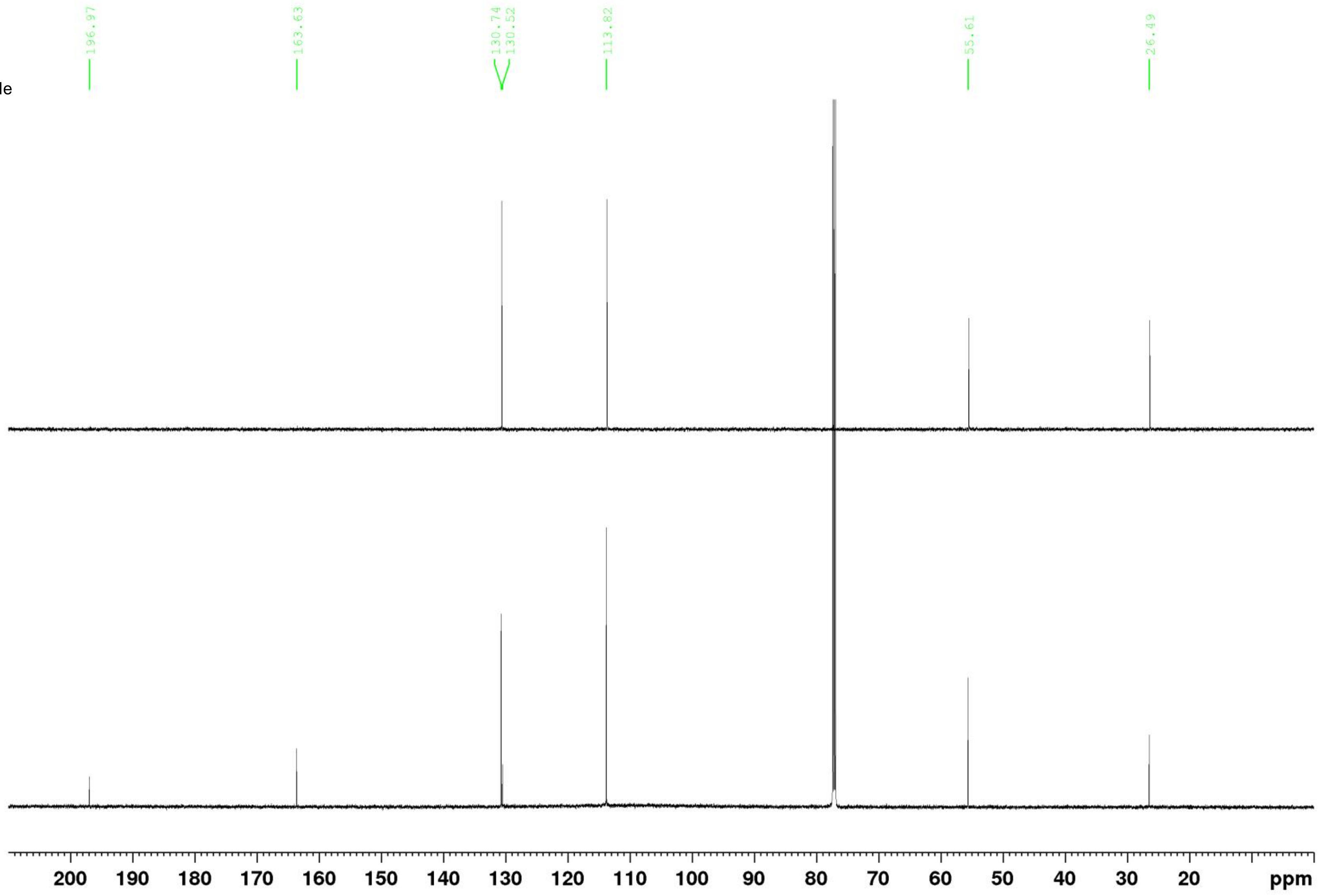

18e ^{1}H NMR, 500 MHz, $CDCl_3$

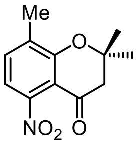

18e

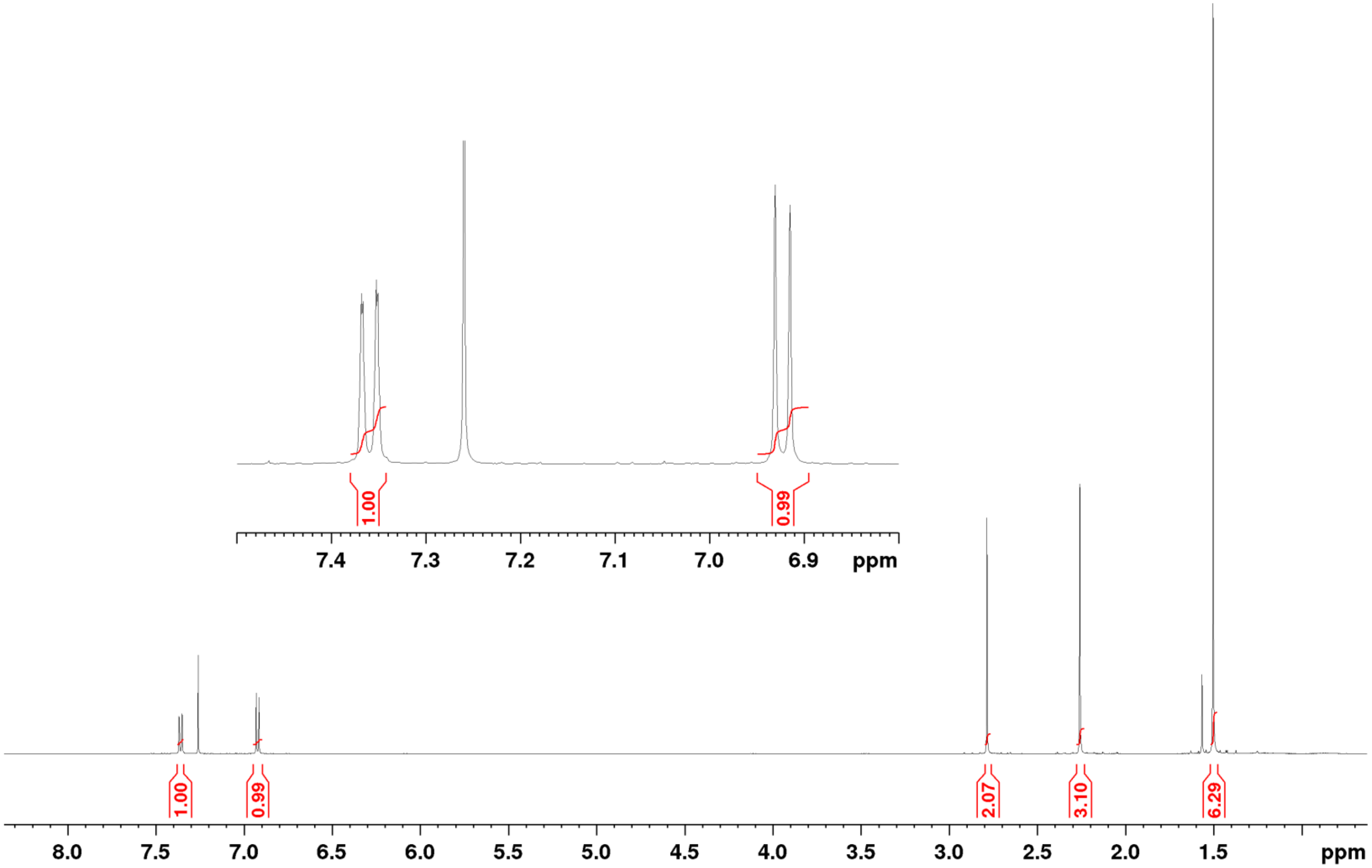

18e ^{13}C NMR, 125 MHz, $CDCl_3$

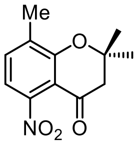

18e

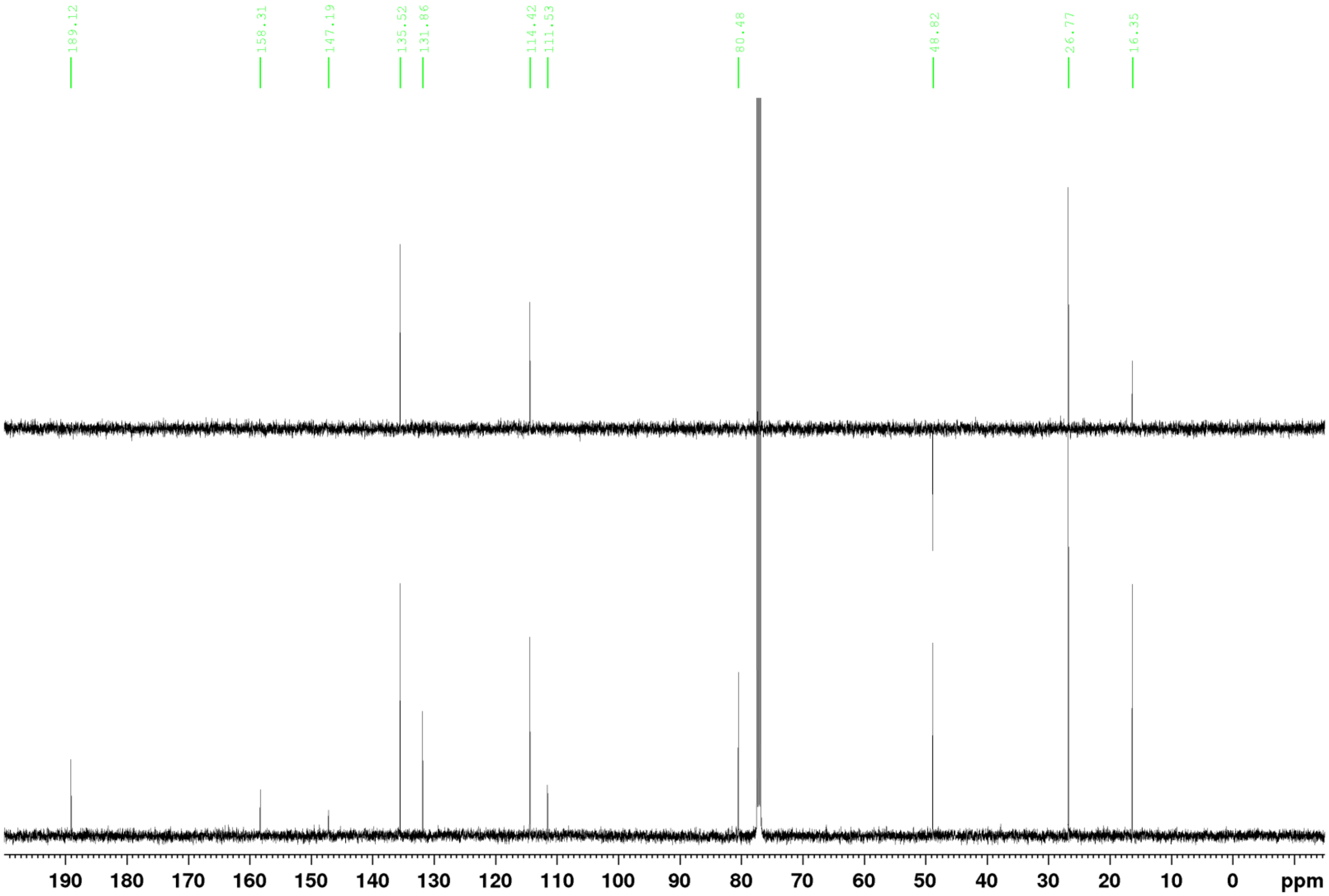

18f ^{1}H NMR, 500 MHz, $CDCl_3$

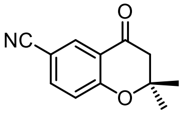

18f

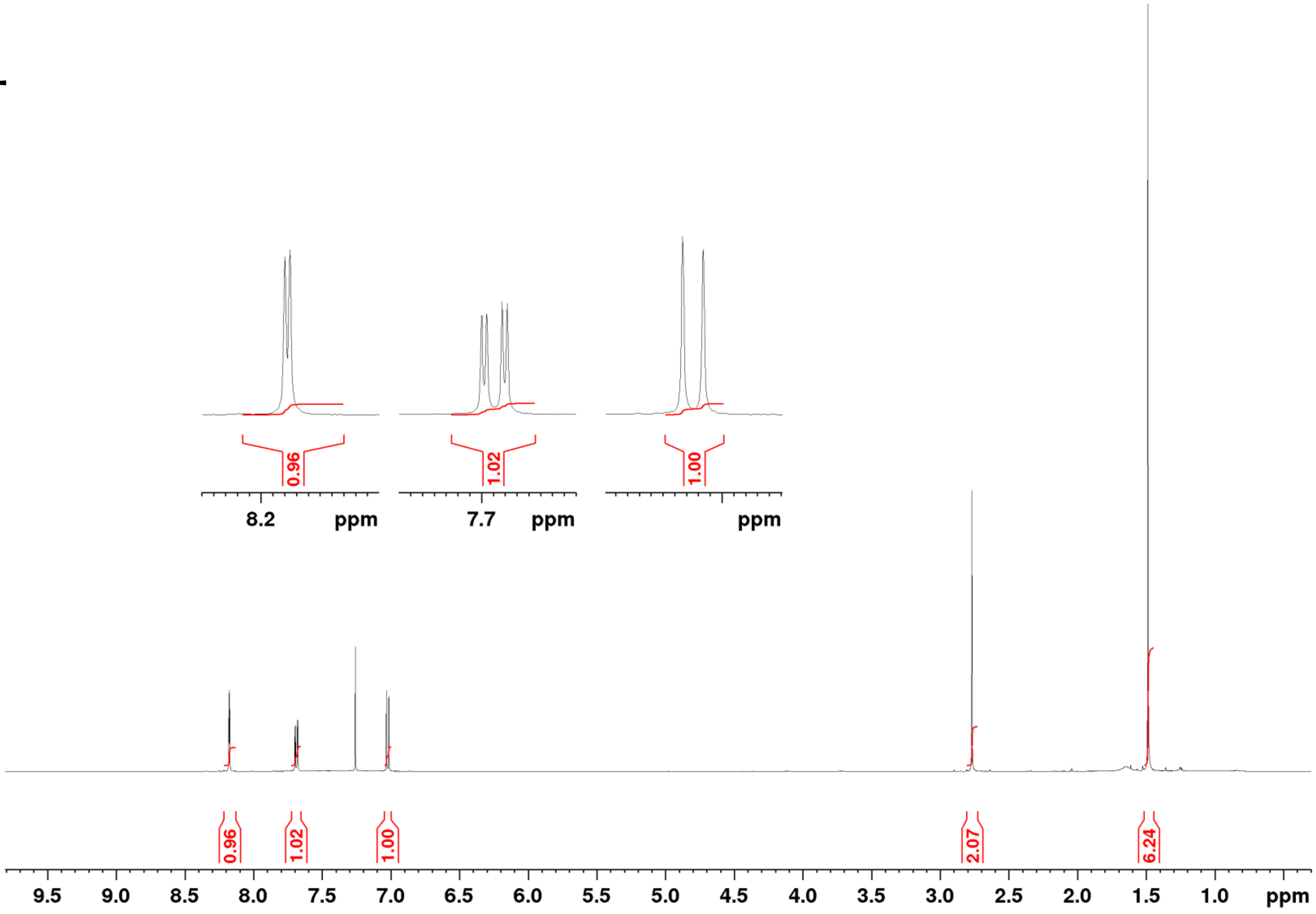

18f ^{13}C NMR, 125 MHz, $CDCl_3$

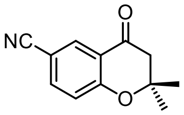

18f

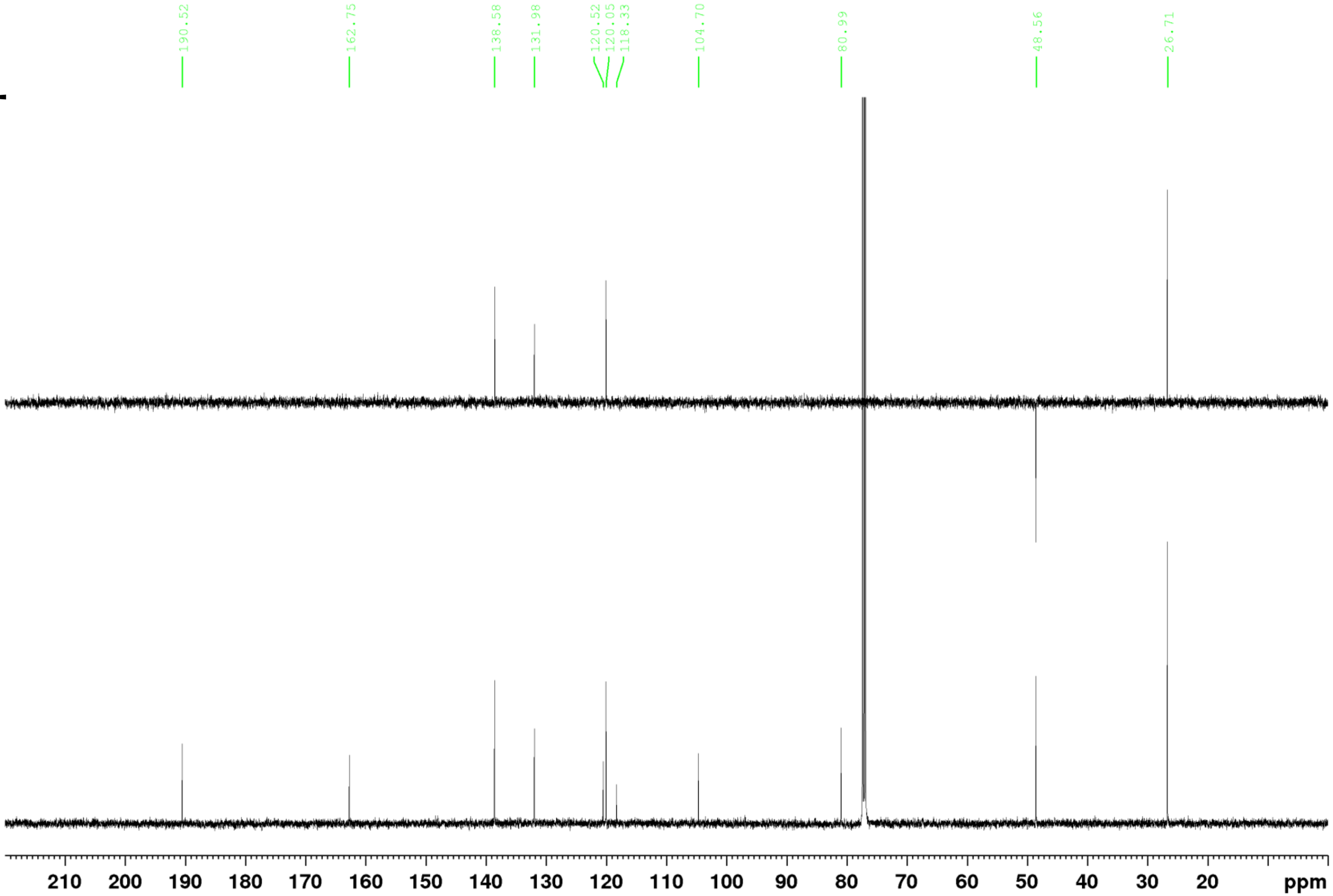

18g ^{1}H NMR, 500 MHz, $CDCl_3$

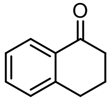

18g

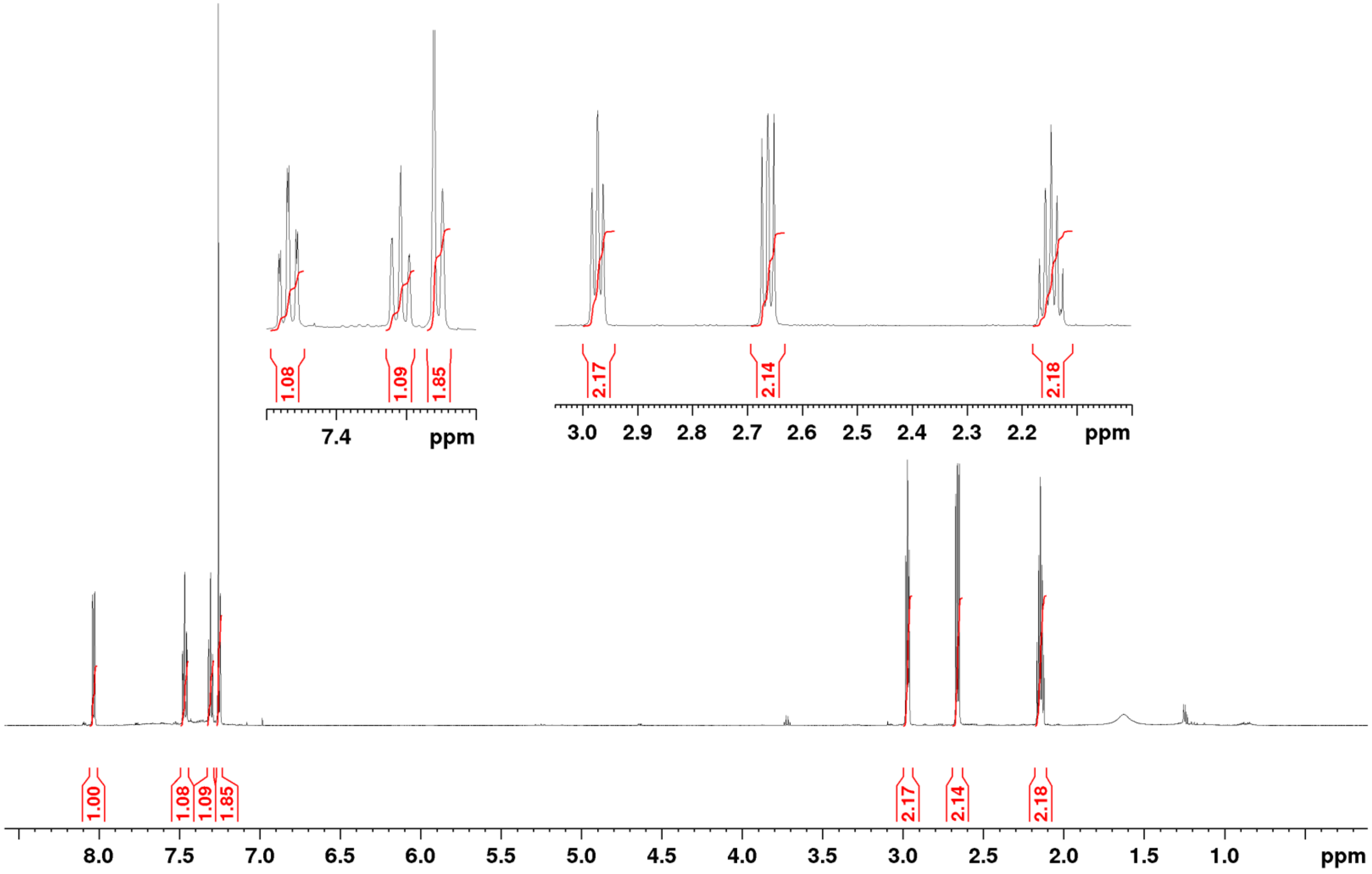

18g ^{1}H NMR, 500 MHz, $CDCl_3$

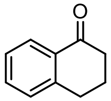
18g

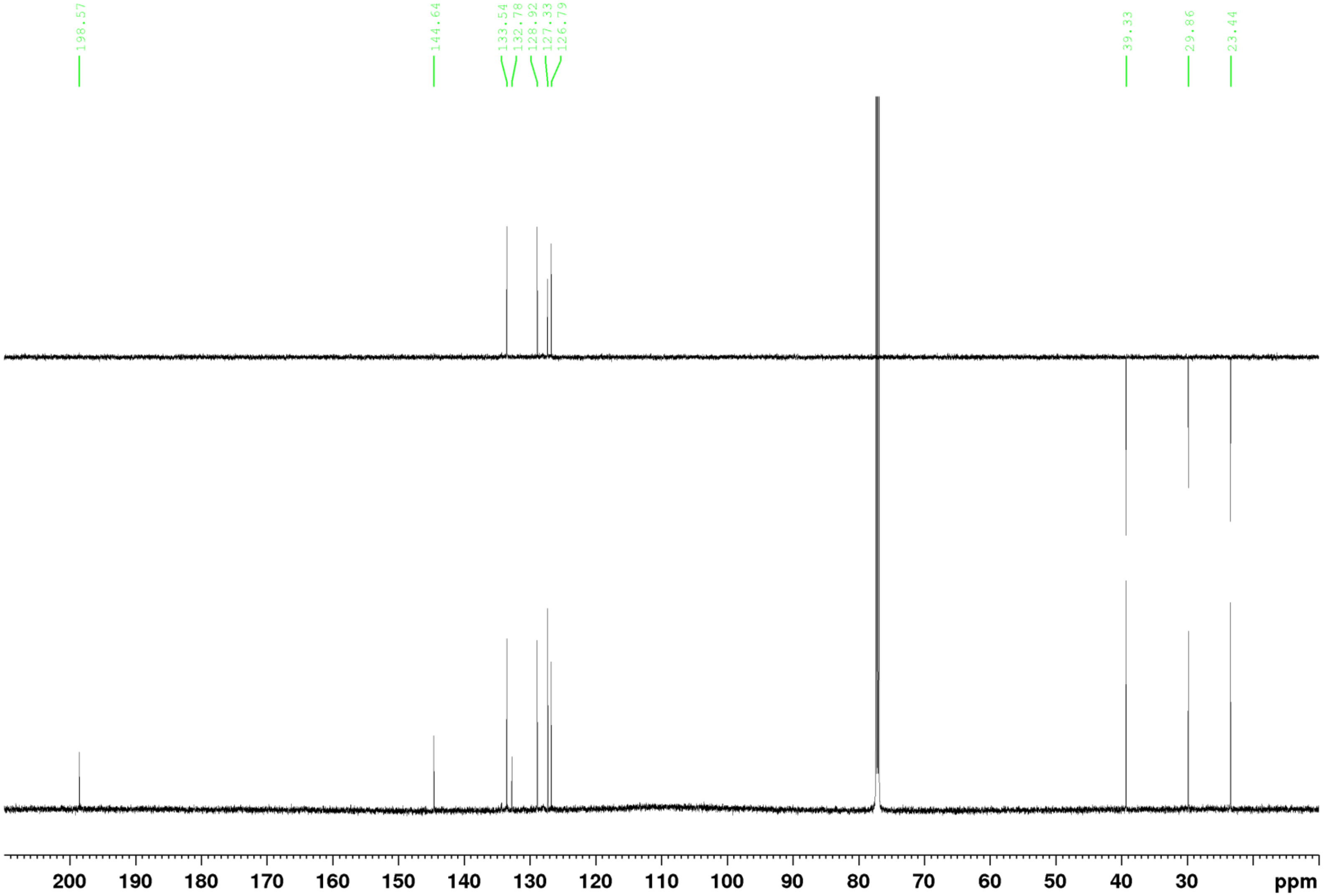

18h ^{1}H NMR, 600 MHz, $CDCl_3$

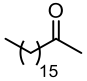

18h

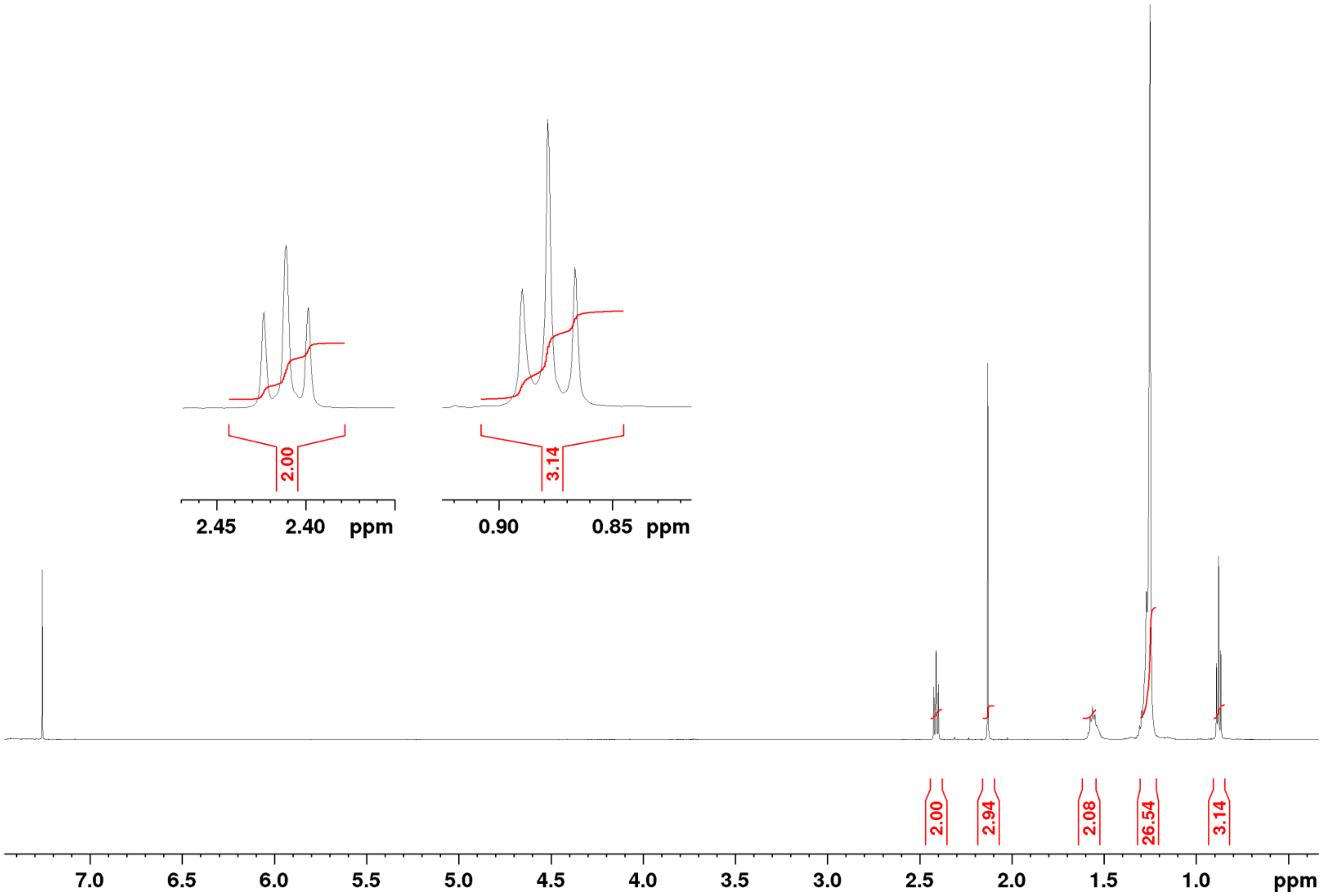

18h ^{13}C NMR, 151 MHz, $CDCl_3$

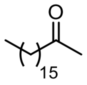

18h

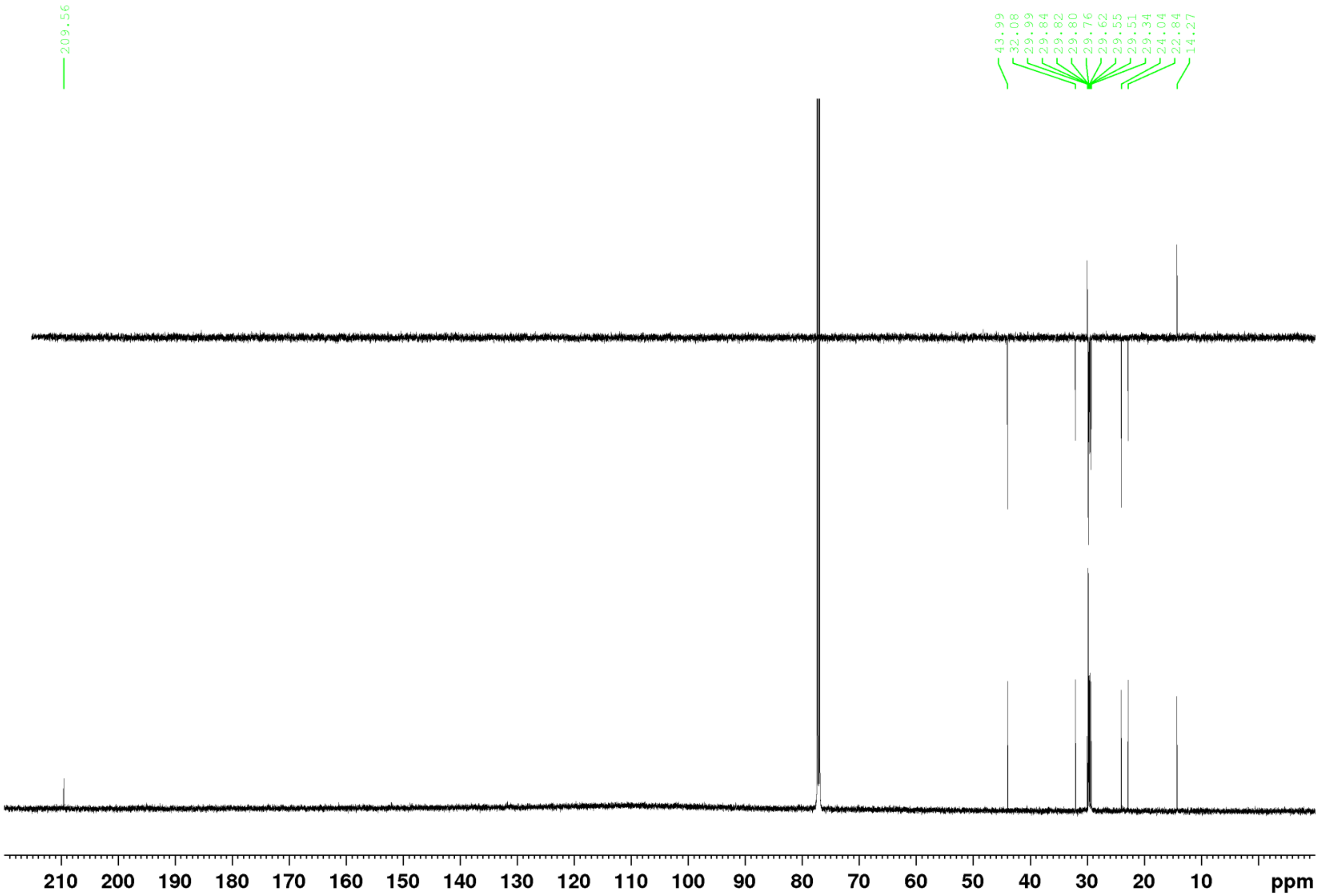

18h ^{13}C NMR, 151 MHz, $CDCl_3$, detail

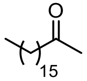

18h

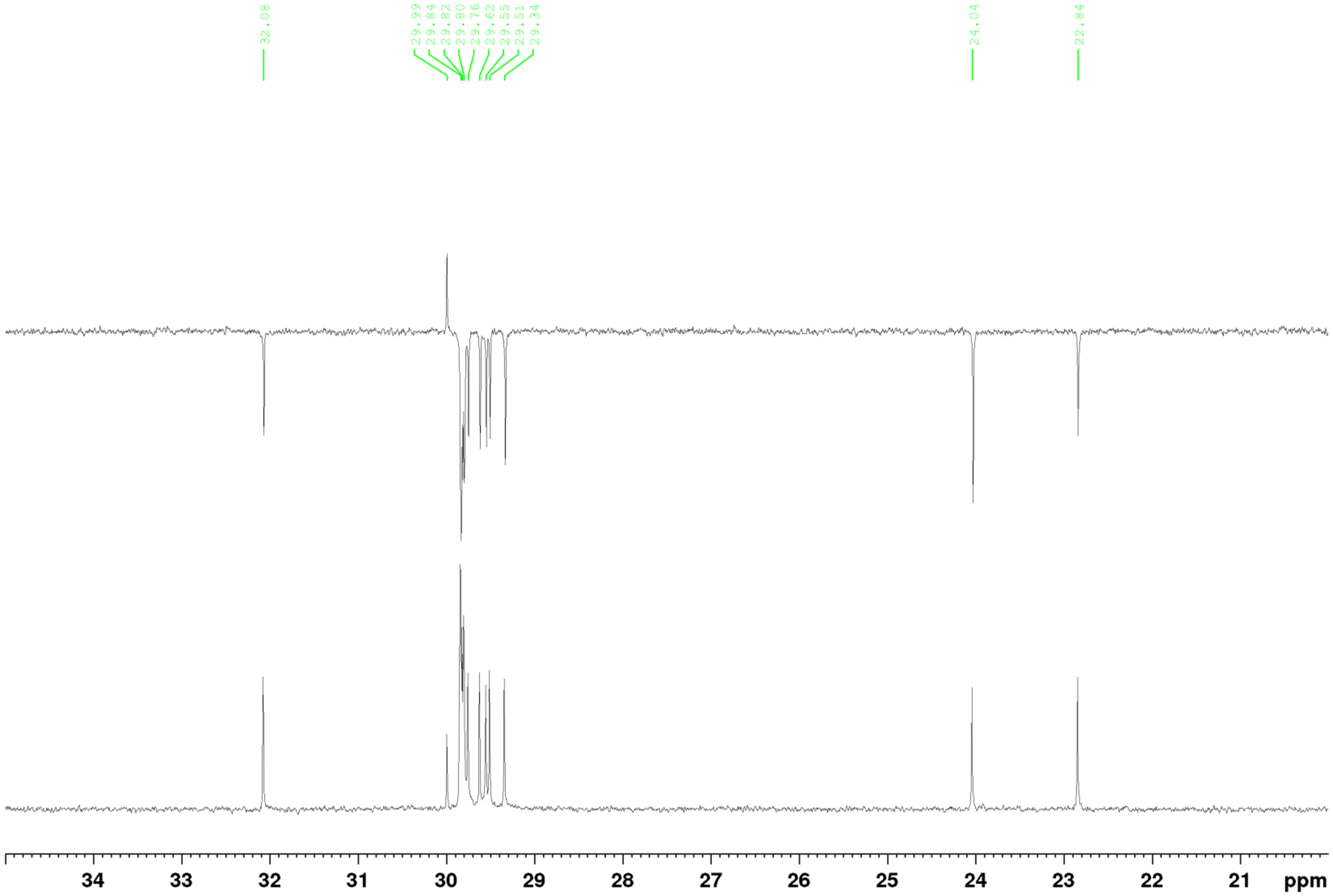

Supplement: SC-014-D2SC06083C-s001 [file SC-014-D2SC06083C-s001.pdf]
